# Supplementary material for: PTEN deficiency exposes a requirement for an ARF GTPase module for integrin‐dependent invasion in ovarian cancer
Source: EMBO J. 2023 Aug 14;42(18):e113987. doi: 10.15252/embj.2023113987 (PMC10505920; doi:10.15252/embj.2023113987)
Supplement: Supplementary file 1 — Appendix S1 [file EMBJ-42-e113987-s011.pdf]

PTEN deficiency exposes a requirement for an ARF GTPase module for integrin-dependent invasion in ovarian cancer.

Appendix

Table of Contents

Appendix Figure S1 2
Appendix Figure S2 5
Appendix Figure S3 8

Appendix Figure S1

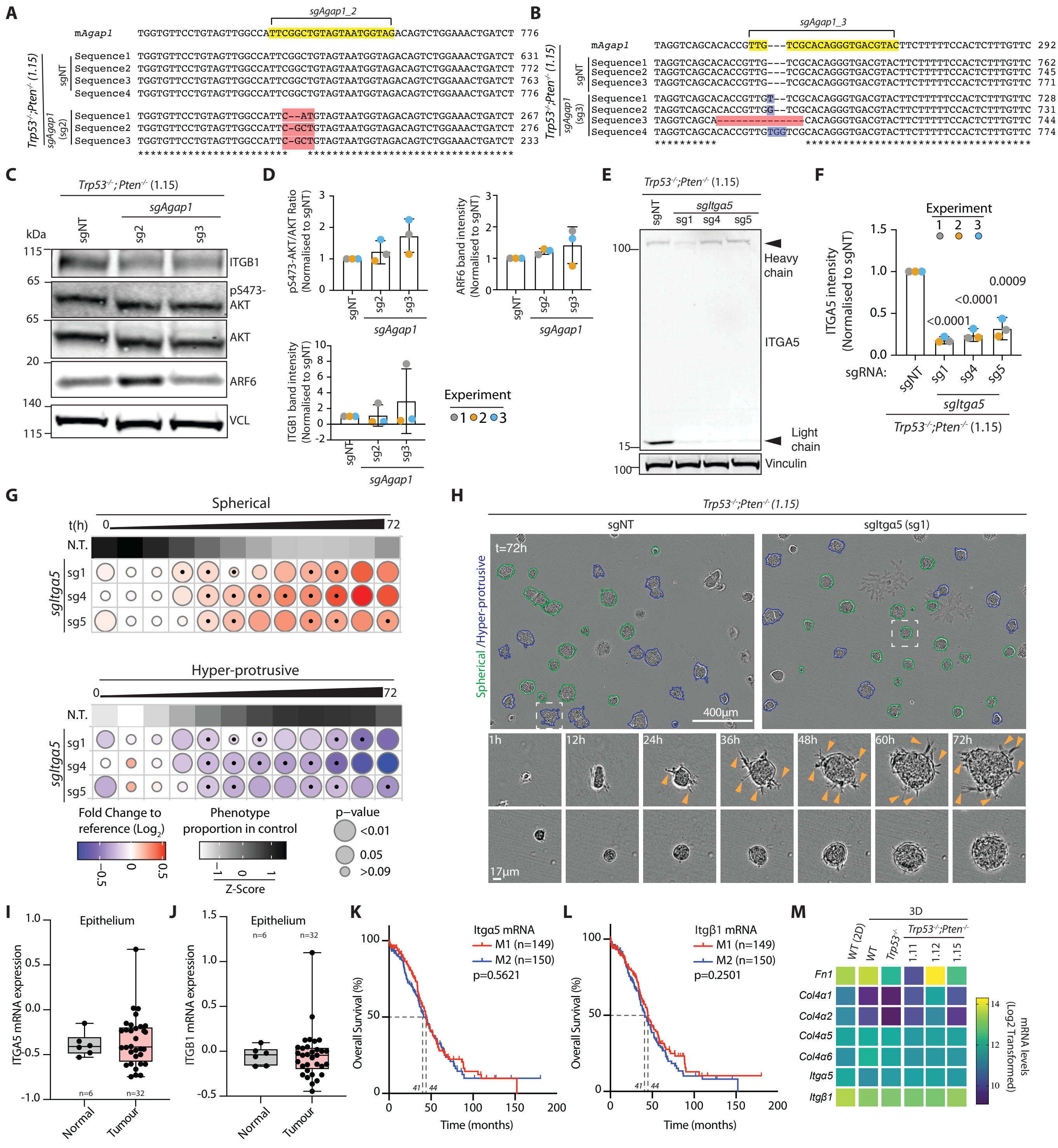

Appendix Figure S1. Itga5 contribution to collective invasion.

A-B. Sequencing of CRISPR editing of the murine *Agap1* locus with sg2 **(A)** or sg3 **(B)** compared to control (sgNT). Yellow, sgRNA sequence; red, deletions; blue, insertions.

C-D. Western blotting **(C)** and quantitation **(D)** of β1-integrin (ITGB1), pS473-AKT, AKT and ARF6 in lysates from *Agap1* KO cells (sgNT versus sg2 or sg3). VCL is loading control for AKT, pS473-AKT and ARF6 and sample integrity control for ITGB1. Representative blots of n=3 independent lysate preparations. **(D)** Data, mean ± SD for pS473-AKT:total AKT band intensity ratio, normalised to control (sgNT). Unpaired, two-tailed t-test, p values not significant (>0.05).

E-F. Western blot **(E)** and quantitation (**F**), α5 integrin (ITGA5) in *ITGA5* KO cells (sg1, sg4, sg5 versus sgNT). VCL, loading control. Representative blots of n=3 independent lysate preparations. **(F)** Data, mean ± SD sum intensity of ITGA5 heavy and light chain bands, normalised to ID8 Trp53$^{-/-}$;*Pten*$^{-/-}$ 1.15 cells treated with sgNT. Unpaired, two-tailed t-test, p values are annotated.

G. Frequency of Spherical and Hyper-protrusive phenotypes upon CRISPR-mediated KO of *Itga5,* 6 hr time intervals over 72 hrs. Heatmap (grayscale) - phenotype proportion (z-score) in control (sgNT). Heatmap (blue-red) - $\log_2$ fold change from control. P-values, bubble size (Cochran-Mantel-Haenszel test with Bonferroni adjustment). Black dot, homogenous effect across independent experiments (Breslow-Day test, Bonferroni adjustment, non-significant). N=3 independent experiments, 3-6 technical replicates/experiment. Total spheroid number per condition, Table EV 1.

H. Representative phase contrast images of spheroids described in **(G)**. Outlines pseudocoloured for classification (Spherical, green; Hyper-protrusive, blue) at indicated timepoints. Magnified individual spheroids from boxed regions. Arrowheads, protrusions into ECM. Scale bars, 400µm or 17µm, as indicated.

I-J. *Itgα5* **(I)** and *Itgβ1* **(J)** mRNA levels in LCM normal ovarian surface epithelium versus HGSOC epithelium. Dataset, GSE40595, sample size (n) and p-values (Mann-Whitney) annotated, whiskers Min-Max, line at median.

K-L. Overall survival (% patients, months; TCGA OV dataset), of patients grouped by low (M1) versus high (M2) mRNA levels, based on a median split, of **(K)** *ITGA5* or **(L)** *ITGB1*. Median survival, sample size (n) and p-value, Log-rank test (Mantel-Cox) annotated.

M. Heatmap, Log_2-transformed RNA-sequencing read counts of Collagen IV, Fibronectin *Itgα5* and *Itgβ1* in ID8 spheroids and 2D monolayers (Wild Type, WT (2D)) across n=4 independent RNA preparations.

Appendix Figure S2

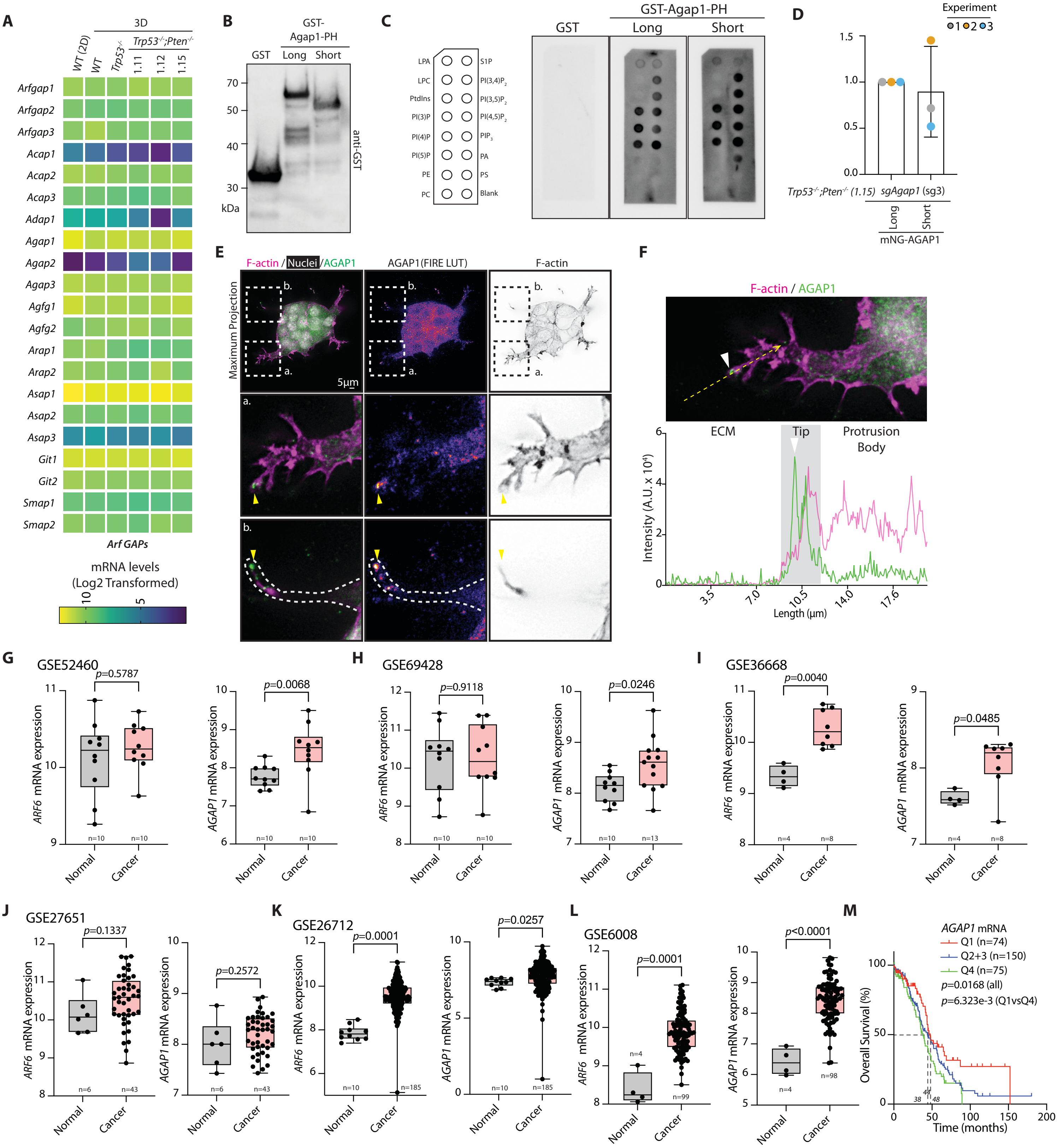

Appendix Figure S2. Further characterization of AGAP1 biological and clinical features.

A. Heatmap, log_2-transformed RNA-sequencing read counts of ARF GAPs in ID8 spheroids and 2D monolayers (Wild Type, WT (2D)) across n=4 independent RNA preparations.

B. Western blot for GST performed on purified recombinant GST alone, or GST-fusion of both isoforms of AGAP1 PH domains. Representative of n=2 blots performed.

C. Binding of the GST (Control), GST-AGAP1 PH Long or GST-AGAP1 PH Short fusion proteins to phospholipids immobilised on a cellulose membrane (cartoon on left denotes lipid position. Probed using a-GST antibody for visualization. Representative of n=3 independent experiments. LPA, Lysophosphatidic acid; LPC, Lysophosphocholine; PE, Phosphatidylethanolamine; PC, Phosphatidylcholine; S1P, Sphingosine-1-phosphate; PA, Phosphatidic Acid; PS, Phosphatidylserine.

D. Quantitation of **(Figure 6C).** Data, mean ± SD of mNG band intensity between mNG-AGAP1-Long and mNG-AGAP1-Short isoforms. Unpaired, two-tailed t-test, p values ns, non-significant (>0.05).

E. Immunofluorescence and confocal imaging of *Trp53*$^{-/-}$;*Pten*$^{-/-}$ 1.15 spheroids stained for AGAP1 (green) and F-actin (magenta). Magnified images from boxed regions, pseudocoloured in inverted grayscale (F-actin) or FIRE LUT (AGAP1). Arrowheads, labelling at protrusion tips. Scale bars = 5 μm. Representative of n=3 spheroids imaged.

F. Intensity profiles for AGAP1 (green) and F-actin (magenta) from spheroid in **(E)**. Tip measured is annotated, ECM to body, yellow arrow, tip, white arrowhead.

G-L. *ARF6 and AGAP1* mRNA levels in normal ovary versus tumour. Specific datasets, sample size (n) and p-values (Mann-Whitney) annotated, whiskers Min-Max, line at median.

M. Overall survival (% patients, months; TCGA OV dataset), of patients grouped by *AGAP1* mRNA levels based on quartile. Median survival, sample size (n) and p-value, Log-rank test (Mantel-Cox) annotated.

Appendix Figure S3

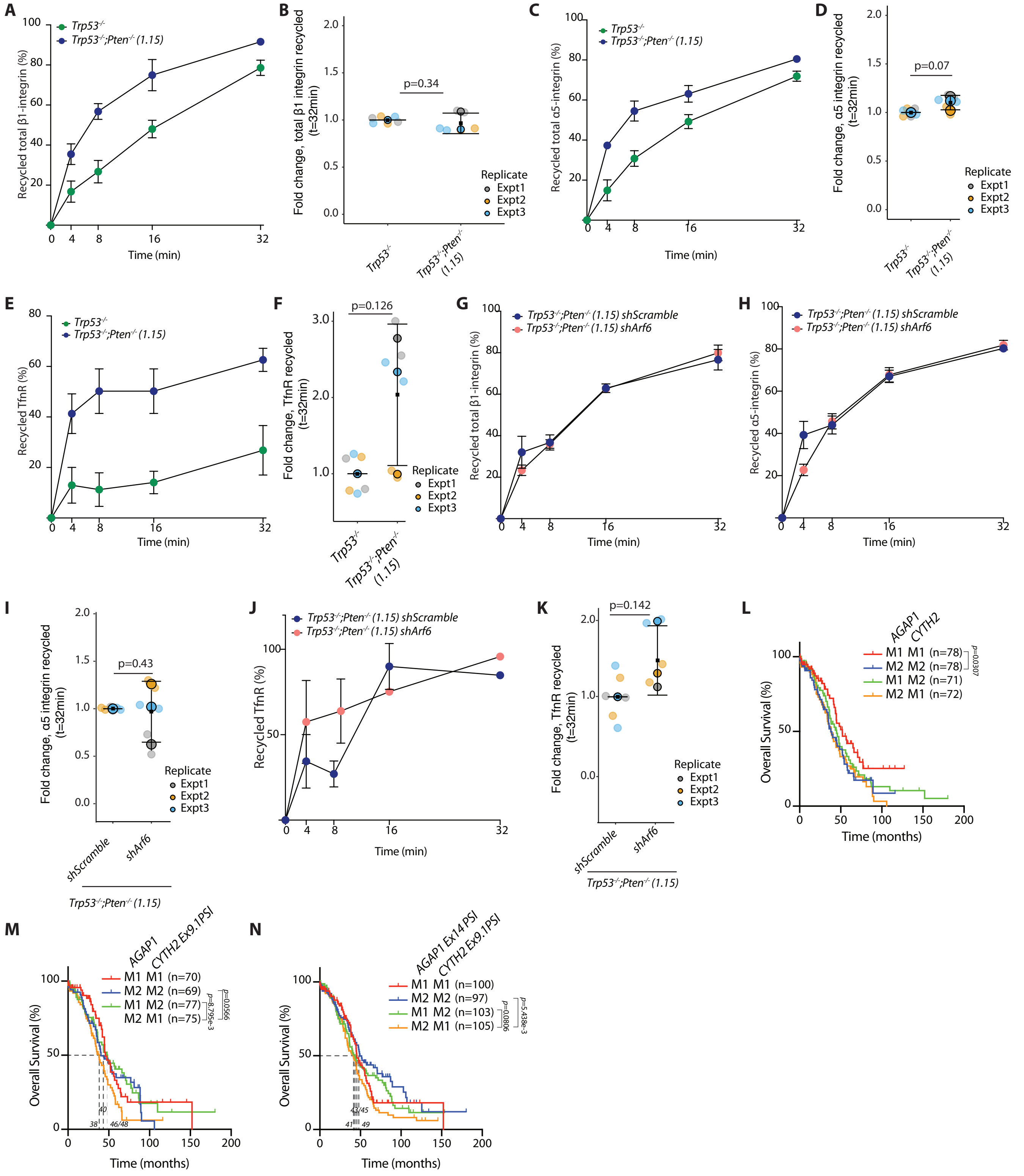

Appendix Figure S3. The ARF6 module does not affect recycling of other cargos.

A-K. Representative capture ELISA graphs (**A,C,E,G,H,J**) and associated quantitation (**B,D,F,I,K**) for recycling of internalized cargoes. *Trp53*$^{-/-}$ versus *Trp53*$^{-/-}$*;Pten*$^{-/-}$ cells (**A-F**) or *Trp53*$^{-/-}$*;Pten*$^{-/-}$ cells expressing sh*Scramble* versus sh*Arf6* (**G-K**). Graphs are shown for total β1 integrin (**A,B,G**), α5 integrin **(C,D,H,I)** or Transferrin receptor (TrnR) **(E,F,J,K)**. Graphs shown are representative of n=3 independent replicates apart from total β1 integrin in *Trp53*$^{-/-}$*;Pten*$^{-/-}$ cells expressing sh*Arf6,* for which n=1. Data, mean (black square) ± SD for 3 repeated experiments (large circles), 1-3 technical replicates/experiment/timepoint (small circles), two-tailed t-test, p-values are annotated.

L-N. Overall survival (% patients, months; TCGA OV dataset) of patients grouped in combinations of a median split of (**L**) *AGAP1* mRNA and *CYTH2* mRNA, (**M**) *AGAP1* mRNA and *CYTH2* Ex9.1 PSI, (**N**) *AGAP1* Ex14 PSI and *CYTH2* Ex9.1 PS. Median survival, sample size (n) and p-value, Log-rank test (Mantel-Cox) annotated.
