# Supplementary material for: PTEN deficiency exposes a requirement for an ARF GTPase module for integrin‐dependent invasion in ovarian cancer
Source: EMBO J. 2023 Aug 14;42(18):e113987. doi: 10.15252/embj.2023113987 (PMC10505920; doi:10.15252/embj.2023113987)
Supplement: Supplementary file 2 — Expanded View Figures PDF [file EMBJ-42-e113987-s024.pdf]

Expanded View Figures

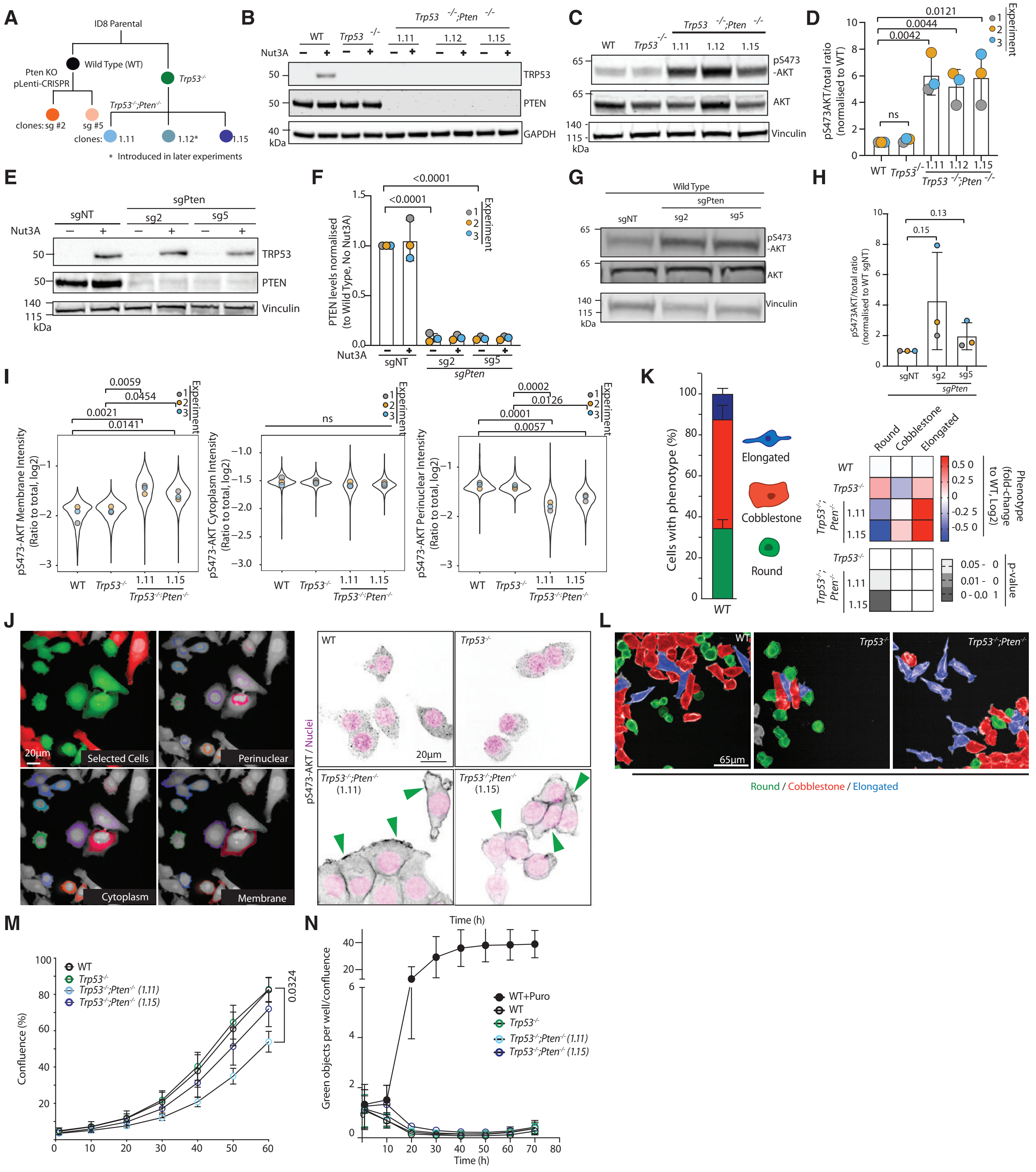

Figure EV1.

 © 2023 The Authors

Figure EV1. Characterisation of *Pten* loss effect on PI3K-AKT in 2D culture.

A Schema, derivation of *Pten* and *Trp53* alterations in ID8 sublines.
B, C Western blot in ID8 sublines. (B) TRP53, PTEN, GAPDH expression upon Nutlin-3A (MDM2 inhibitor) treatment to stabilise P53 or (C) pS473-AKT, AKT, Vinculin (VCL) expression. Each panel is representative of $n = 3$ lysate preparations for each subline. GAPDH and VCL are loading controls for each panel.
D Quantitation of (C). Data, mean ± SD of pS473-AKT/total AKT intensity ratio, normalised to WT. Unpaired, two-tailed *t*-test; *P*-values, annotated.
E Western blot, TRP53, PTEN, VCL in ID8 Wild Type cells expressing non-targeting (sgNT) or *Pten*-targeting sgRNA upon Nutlin-3A (MDM2 inhibitor) treatment. Representative of $n = 3$ lysate preparations for each subline. VCL is loading control.
F Quantitation of PTEN band intensity from (E). Data, mean ± SD of band intensity, normalised to ID8 Wild-Type sgNT. Unpaired, two-tailed *t*-test; *P*-values, annotated.
G Western blot, pAKT(S473), AKT pan, VCL in ID8 Wild Type cells expressing non-targeting (sgNT) or *Pten*-targeting sgRNA. Representative of $n = 3$ lysate preparations for each subline. VCL is loading control.
H Quantitation of (G). p:t AKT ratio. Data, mean ± SD of band intensity, normalised to ID8 Wild-Type sgNT. Unpaired, two-tailed *t*-test; *P*-values, annotated.
I Quantitation of (J). Data, ratio pS473-AKT signal at indicated regions to total area. Means, overlaid on plots of all data points (exact number per replicate provided in Table EV1) as distinctly coloured dots according to culture replicate number. *P*-values are annotated, ANOVA with Tukey's honest significant difference (HSD) test.
J ID8 cells plated in 2D, stained with pS473-AKT (grey) and Hoechst (Magenta) (bottom panels), segmented into indicated regions (perinuclear, cytoplasmic, membrane) (top panels). Colour in selected cells panel: red, excluded due to touching image edge, green, included for segmentation. Arrowheads, pS473-AKT at cell membrane. Scale bar, 20 µm. $N = 3$ independent experiments, four technical replicates/subline/experiment. Total cell number per condition, Table EV1.
K Percentage of cells classified as Round (green), Cobblestone (red) or Elongated (blue) in Wild Type ID8 cells. Classification of ID8 WT, *Trp53*$^{-/-}$ and *Trp53*$^{-/-}$;*Pten*$^{-/-}$ *1.15* cells grown in 2D as Round, Cobblestone, or Elongated. Heatmap, $\log_2$ fold change, mean proportion across indicated lines. Grayscale heatmap, *P*-values for each comparison. $N = 2$ independent experiments, four technical replicates/subline/experiment. Total cell number per condition, Table EV1.
L Representative images of cells quantified in (K). Cells classified by shape (Round, green; Cobblestone, red; Elongated, blue). Scale bar, 65 µm.
M Proliferation assay based on well confluence over time. $N = 3$ experiments set up with repeated cultures of each subline, 4–5 technical replicates/subline/experiment. Data are presented as mean ± SD. Unpaired, two-tailed *t*-test between WT and each of the sublines per time point. Significant *P*-values annotated.
N Cell death assay, green object (Sytox green fluorescence) confluence over time. $N = 2$ experiments set up with repeated cultures of each subline, 4 technical replicates/subline in each experiment. Kruskal–Wallis ANOVA was performed at $t = 10$ h and $t = 20$ h, all comparisons are nonsignificant (P-value > 0.05).

© 2023 The Authors

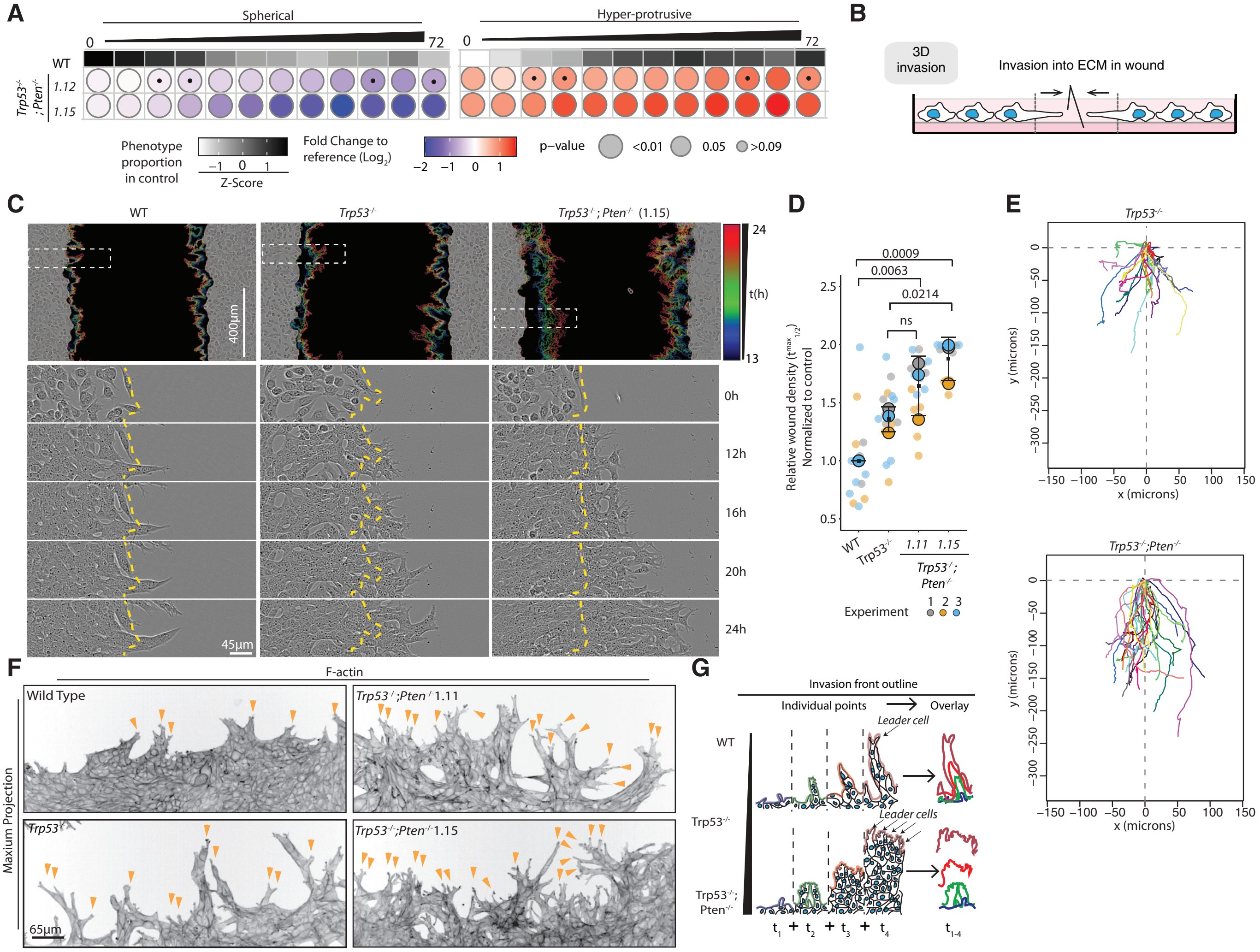

Figure EV2. Collective invasion into ECM in orthogonal assays.

A Frequency of Spherical and Hyper-protrusive phenotypes in ID8 WT, *Trp53*$^{-/-}$; *Trp53*$^{-/-}$;*Pten*$^{-/-}$ clones 1.12 and 1.15. spheroids, 6-h time intervals over 72 h. Heatmap (grayscale)—phenotype proportion (z-score) in control (WT). Heatmap (blue-red)—$\log_2$ fold change from control. *P*-values, bubble size (Cochran–Mantel–Haenszel test with Bonferroni adjustment). Black dot, homogenous effect across independent experiments (Breslow–Day test, Bonferroni adjustment, nonsignificant). $N = 3$ independent experiments, 3–4 technical replicates/experiment. Total spheroid number per condition, Table EV1.

B Schema, 3D invasion into ECM of wounded ID8 monolayer.

C Representative images of 3D invasion assay, monolayers plated onto ECM, wounded and then overlaid with 50% ECM (gel). Outlines of invasive front at different time points, pseudocoloured by time (rainbow legend), overlaid as concatenate on phase image of initial wound. Boxes, regions for different timepoints. Yellow lines, initial wound. Scale bar, 400 or 45 µm (indicated).

D Quantification of (C). Graph, Relative Wound Density (RWD) at $t_{1/2}$ max (time when WT 50% closed). Data, mean (black square) ± SD for 3 independent experiments (large circles) with 3–6 technical replicates/subline/experiment (small circles). ANOVA with Tukey's HSD test; exact *P*-values, annotated; ns, nonsignificant.

E Representative spider plots, leader cell movement in first 19 h of invasion of *Trp53*$^{-/-}$ and *Trp53*$^{-/-}$;*Pten*$^{-/-}$ 1.15 ID8 cells. $N = 2$ independent experiments, 10–25 leader cells tracked in each, across 4–6 technical replicates/experiment.

F Confocal images of wounded monolayer invasive fronts, stained for F-actin (Phalloidin). Arrowheads, protrusion tips. Scale bar, 65 µm. Representative of 7–12 fields imaged across $n = 3$ independent experiments, 4 technical replicates/experiment.

G Schema, loss of PTEN phenotype, a sheet-like mode of invasion with most ECM-abutting cells acting as "leader cells," compared with leader and follower cell chains in WT.

 © 2023 The Authors

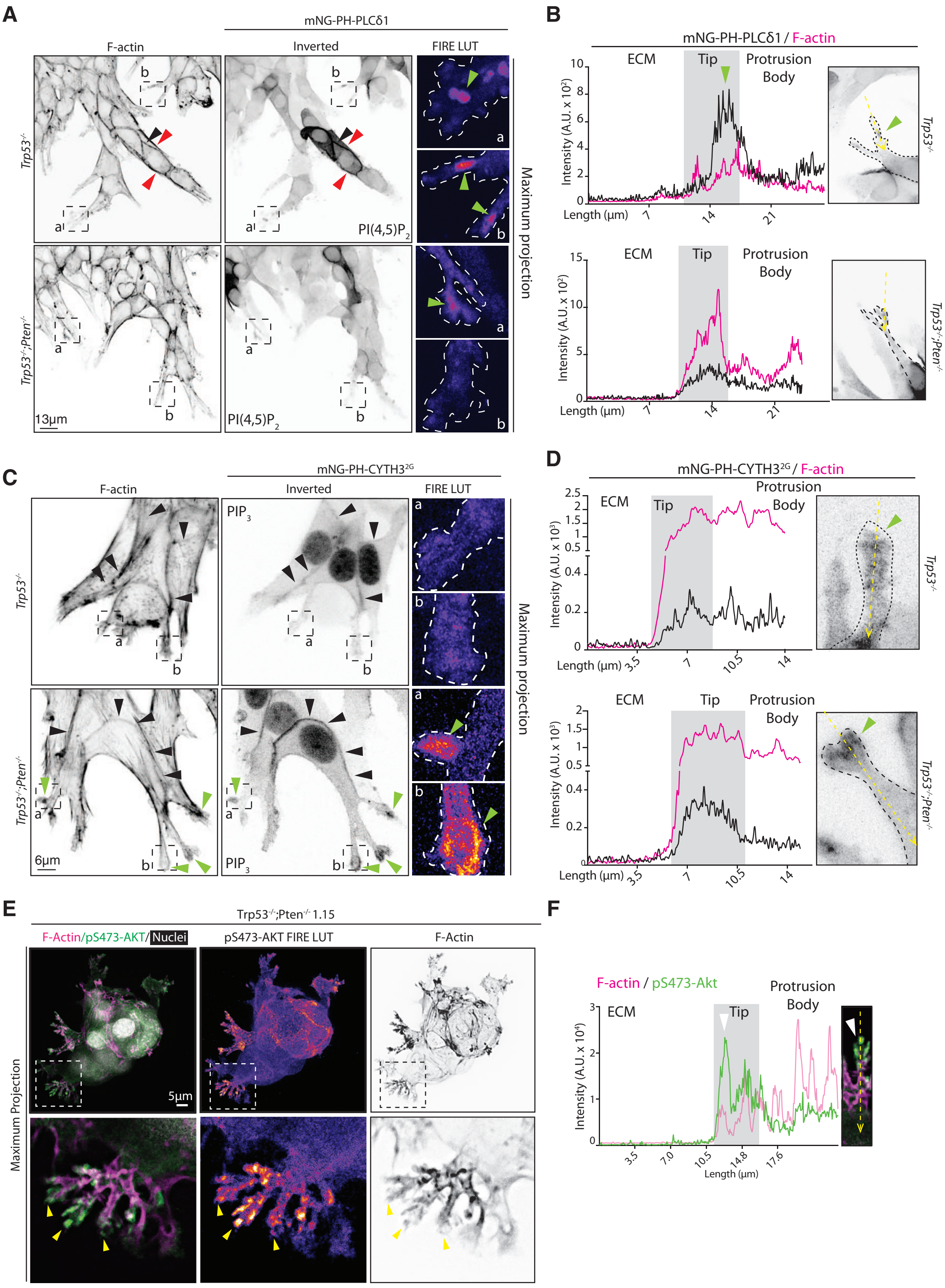

A
mNG-PH-PLCδ1
F-actin
Inverted
FIRE LUT
Trp53-/-
Trp53-/-;Pten-/-
PI(4,5)P2
13μm
Maximum projection
B
mNG-PH-PLCδ1 / F-actin
ECM
Tip
Protrusion Body
Intensity (A.U. x 10²)
Length (μm)
C
mNG-PH-CYTH3 2G
PIP3
6μm
D
mNG-PH-CYTH3 2G / F-actin
Intensity (A.U. x 10³)
E
Trp53-/-;Pten-/- 1.15
F-Actin/pS473-AKT/Nuclei
pS473-AKT FIRE LUT
F-Actin
Maximum Projection
5μm
F
F-actin / pS473-Akt
Intensity (A.U. x 10⁴)

Figure EV3.

Figure EV3. Characterisation of Phosphoinositide enrichment in Invasion assays.

A, C Confocal images, *Trp53*$^{-/-}$ or *Trp53*$^{-/-}$;*Pten*$^{-/-}$ invasive monolayer fronts with cells expressing mNeonGreen (mNG) tagged biosensors for (A) PI(4,5)P_2 (PH-PLCδ1) or (C) PIP_3 (CYTH3^{2G}). Representative of (A) 7 (*Trp53*$^{-/-}$) or 9 (*Trp53*$^{-/-}$;*Pten*$^{-/-}$) fields or (C) 8 (*Trp53*$^{-/-}$) or 9 (*Trp53*$^{-/-}$;*Pten*$^{-/-}$) fields imaged across n = 2 experiments set up with repeated cultures of each subline. Magnified boxed regions, pseudocoloured with FIRE LUT. Arrowheads: cell–cell contacts, black; protrusions, green; cell-ECM contacts, red. Scale bar, (A)13 μm, (C) 6 μm.

B, D Intensity profiles for mNG PH-PLCδ1 (B) or mNG PH- CYTH3^{2G} (D) from invasive monolayers on (A, C). Tips measured correspond to boxed, magnified regions on images in (A, C). Arrowhead, phosphoinositide-rich region.

E Immunofluorescence and confocal imaging of *Trp53*$^{-/-}$;*Pten*$^{-/-}$ 1.15 spheroid stained for pS473-AKT (green or FIRE LUT), F-actin (magenta or black) and Hoechst (grey). Magnified images from boxed regions. Arrowheads, labelling of pS473-AKT at protrusion tips. Scale bar, 5 μm. Representative of n = 5 spheroids.

F Intensity profile for pS473-AKT (green) and F-Actin (magenta) from spheroid in (A). Tip measured is annotated, ECM to body, yellow arrow; tip, white arrowhead.

Figure EV4. Further characterisation of ARF6 role upon *Trp53* and *Pten* loss.

A, B Western blot (A) and quantitation (B) of pS473-AKT, AKT, ARF5, ARF6, GAPDH in ID8 *Trp53*$^{-/-}$;*Pten*$^{-/-}$ 1.15 cell lines expressing shScramble, sh*Arf5* or sh*Arf6*. Representative blots of n = 3 independent lysate preparations. (B) Data, mean ± SD for ARF5, ARF6 and pS473-AKT band intensity ratio, normalised to shScramble. *P*-values, unpaired, two-tailed *t*-test; ns, not significant. GAPDH is loading control for all panels.

C Representative images, ID8 *Trp53*$^{-/-}$;*Pten*$^{-/-}$ 1.15 cell lines expressing sh*Scramble*, sh*Arf5* or sh*Arf6* in wounded monolayers invading ECM. Yellow lines, initial wound. Arrowheads, invasive protrusions. Outlines of invasive front pseudocoloured by time and overlaid as concatenate over phase image of initial wound. Scale bar, 45 μm. *N* = 3 independent experiments, 3–6 technical replicates/experiment.

D Quantitation of (C). Graph, Relative Wound Density (RWD) at $t_{1/2}$ max (time when shScramble 50% closed). Data, mean (black square) ± SD for 3 independent experiments (large circles), 3–6 technical replicates//experiment (small circles). *P*-values, ANOVA with Tukey's HSD test; annotated when significant.

E, F Western blot (E) and quantitation (F) from pS473-AKT, AKT, ARF6, VCL in ID8 *Trp53*$^{-/-}$;*Pten*$^{-/-}$ 1.15 cell lines expressing shScramble or sh*Arf6* (5 individual shRNA sequences). Representative blots of n = 3 (ARF6) or n = 2 (pS473-AKT and AKT) independent lysate preparations VCL is loading control for all panels. (F) Data, mean ± SD for ARF6 and pS473-AKT band intensity normalised to shScramble. *P*-values, unpaired, two-tailed *t*-tests; ns, not significant.

G Regression analysis. Scatter plot, mean Hyper-protrusive level across all time points versus ARF6 protein levels (determined by western blot). Solid black line, best linear fit and dotted cyan lines, 95% confidence interval. *P*-value and R^2, annotated.

H Heatmap, Log_2-transformed RNA-sequencing read counts of each ARF GTPase in ID8 spheroids and 2D monolayers (Wild-Type, WT [2D]) across n = 4 independent RNA preparations.

I Western blot and quantitation for ARF6 protein in ID8 sublines. VCL, loading control. Representative blots of n = 3 independent protein isolations. Quantitation, mean ± SD ARF6 intensity normalised to ID8 WT. *P*-values, unpaired, two-tailed *t*-tests; ns, not significant.

J ARF6-GTP levels in ID8 sublines. Normalised Optical Density (OD) of Arf6-GTP G-LISA. *N* = 3 independent lysate preparations, 3 technical replicates/experiment. Data, mean ± SD of independent replicates (large circles) with technical replicates shown (small circles). *P*-values annotated, student's *t*-test; ns, nonsignificant.

K Western blot, ARF6, T2A, V5, BFP and VCL from lysates extracted from TurboID (control) or ARF6-TurboID-expressing cell lines. VCL, loading control for T2A and BFP and sample integrity control for all other blots. *N* = 3 independent lysate preparations.

L Confocal images, ID8 *Trp53*$^{-/-}$;*Pten*$^{-/-}$ 1.15 cells expressing ARF6-TurboID or TurboID, stained with T2A. Red box, cell–cell contacts, shown in higher magnification and pseudocoloured with FIRE LUT. Black arrowheads, cell–cell contact; green arrowheads, cell periphery. Scale, 10 μm. Representative images from two fields (ARF6-TurboID) or three fields (TurboID alone) from one experiment.

M Western blot with Streptavidin HRP in ID8 *Trp53*$^{-/-}$;*Pten*$^{-/-}$ 1.15 cells treated with Biotin for at times and concentrations indicated. VCL was used as loading control. n = 1 lysate preparation.

N Gene Ontology Cell Compartment (GOCC) enrichment analysis of interactors identified in ID8 *Trp53*$^{-/-}$;*Pten*$^{-/-}$ 1.15 cells expressing ARF6-TurboID compared to TurboID alone. Data, *P*-value ($-Log_{10}$) of enrichment. *N* = 4 independent experiments. Red dotted line, significance threshold.

 © 2023 The Authors

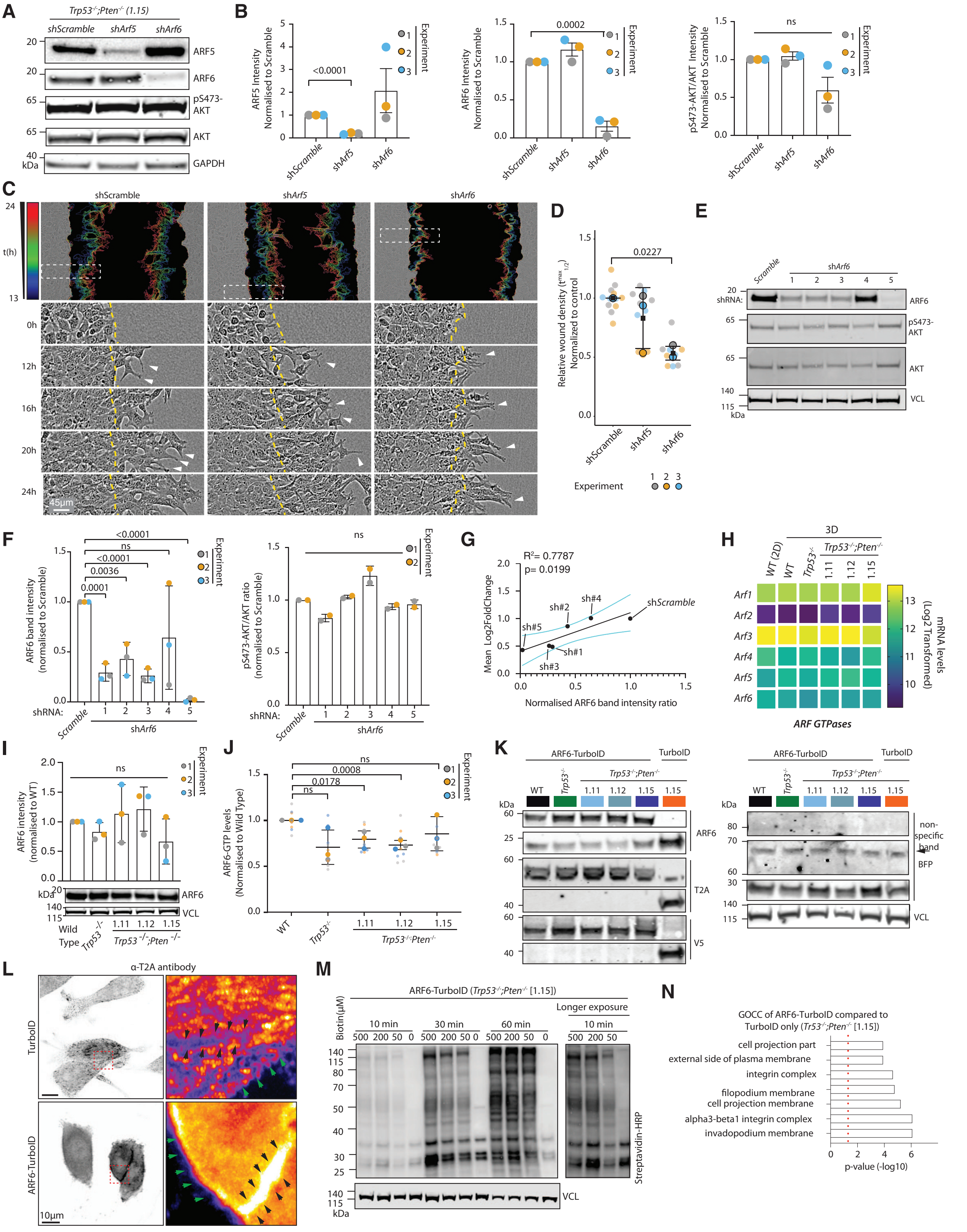

Figure EV4.

Figure EV5. Characterisation of Cytohesin and CYTH2 contribution to invasion.

A Heatmap, log_2-transformed RNA-sequencing read counts of ARF GEFs in ID8 spheroids and 2D monolayers (Wild Type, WT [2D]) across $n = 4$ independent RNA preparations.

B, C Quantitation of ID8 *Trp53*$^{-/-}$;*Pten*$^{-/-}$ spheroids treated with 20 μM SecinH3, 6-h time intervals over 72 h. (B) Heatmap (viridis)—area presented as mean of *Z*-score values, normalised to control (DMSO). (C) Frequency of Spherical and Hyper-protrusive phenotypes. Heatmap (grayscale)—phenotype proportion (*z*-score) in control. Heatmap (blue-red)—log_2 fold change from control. *P*-values, bubble size (Cochran–Mantel–Haenszel test with Bonferroni adjustment). Black dot, homogenous effect across independent experiments (Breslow–Day test, Bonferroni adjustment, non-significant). $N = 3$ independent experiments, 4–5 technical replicates/experiment. Total spheroid number per condition, Table EV1.

D Representative phase contrast images of spheroids described in (B, C). Outlines pseudocoloured for classification (Spherical, green; Hyper-protrusive, blue). Magnified individual spheroids from boxed regions at indicated timepoints. Arrowheads, protrusions into ECM. Scale bars, 400 or 17 μm, as indicated.

E Representative images of ID8 Trp53$^{-/-}$;Pten$^{-/-}$ 1.15 wounded monolayers treated with DMSO or SecinH3 (20 μM) in wounded monolayers invading ECM. Yellow lines, initial wound. Arrowheads, invasive protrusions. Outlines of invasive front pseudocoloured by time and overlaid as concatenate over phase image of initial wound. $N = 3$ independent experiments, 3–5 technical replicates/experiment. Scale bar, 200 or 45 μm.

F Quantitation of (E). Graph, Relative Wound Density (RWD) at $t_{1/2}$ max (time when DMSO 50% closed). Data, mean (black square) ± SD for 3 independent experiments (large circles), 3–6 technical replicates/experiment (small circles). Exact *P*-value annotated, ANOVA with Tukey's HSD test.

G Spider plots of leader cell movement in the first 19 h of invasion of *Trp53*$^{-/-}$;*Pten*$^{-/-}$ 1.15 ID8 cells treated with DMSO or SecinH3. 10–25 leader cells were tracked per experiment ($n = 2$ set-up using repeated cultures of each subline), across multiple technical replicates/experiment. Representative plots from cells tracked in one independent experiment shown.

H–P *CYTH2* mRNA levels in (H, I) LCM normal ovarian surface epithelium versus HGSOC epithelium or normal ovarian stroma versus ovarian cancer-associated stroma, or (J–P) bulk sequencing of normal ovary versus tumour. Specific data set, sample size (*n*) and *P*-values (Mann–Whitney) annotated, whiskers Min-Max, line at median.

Q–T Overall survival (% patients, months; TCGA OV data set), of patients grouped by low (M1) versus high (M2) levels based on a median split of (Q) *CYTH2* mRNA, (R) *CYTH2* exon 9.1 percentage spliced in ratio (PSI), (S) combination of *ARF6* and *CYTH2* mRNA, or (T) combination of A*RF6* mRNA and *CYTH2* Ex9.1 PSI. Median survival, sample size (*n*) and *P*-value, Log-rank test (Mantel-Cox) annotated.

 © 2023 The Authors

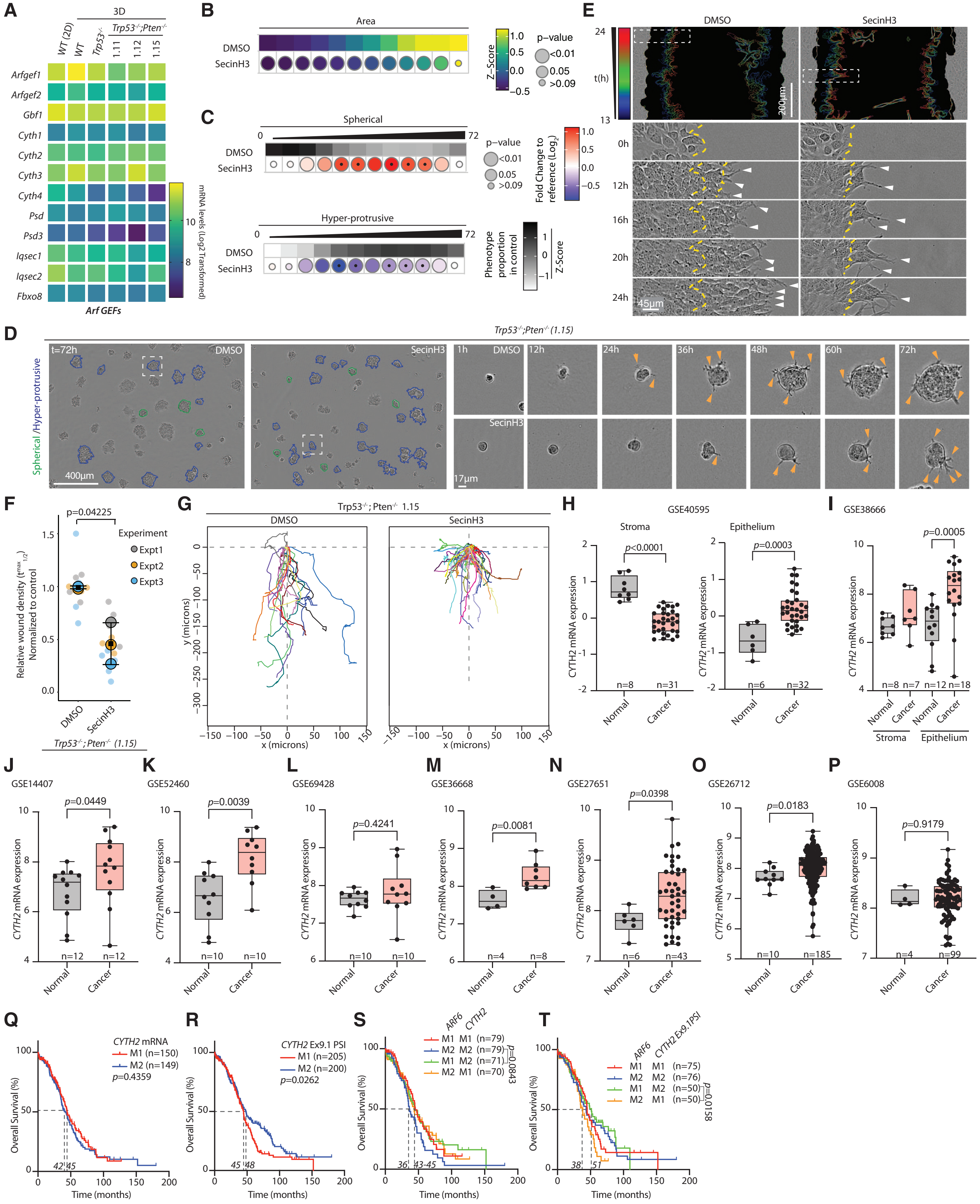

Figure EV5.
