# Supplementary material for: PTEN deficiency exposes a requirement for an ARF GTPase module for integrin‐dependent invasion in ovarian cancer
Source: EMBO J. 2023 Aug 14;42(18):e113987. doi: 10.15252/embj.2023113987 (PMC10505920; doi:10.15252/embj.2023113987)
Supplement: Supplementary file 3 — Table EV1 [file EMBJ-42-e113987-s010.pdf]

Table EV 1

The following is a list of the number of spheroids or cells analysed per experiment in each condition (live cyst imaging or 2D analysis of cell shape and pAKT intensity) using the Incucyte or the Opera Phenix systems repectively.

Figure EV1 Panel I-J	pAKT Intensity Expt1	pAKT Intensity Expt2	pAKT Intensity Expt3
Condition	Cell Number	Object number	Object number
Wild Type	1622	1622	1749
Trp53-/-	1471	1144	1891
Trp53-/-;Pten-/- 1.11	1274	1300	1749
Trp53-/-;Pten-/- 1.15	3150	1096	1468

Figure EV1 Panel K-L	2D Shape Expt1	2D Shape Expt2
Condition	Object number	Object number
Wild Type	7786	6594
Trp53-/-	6366	9840
Trp53-/-;Pten-/- 1.11	5972	4256
Trp53-/-;Pten-/- 1.15	7442	5933

Figure 2 Panels D-F			
	ID8 PTEN Expt1	ID8 PTEN Expt2	ID8 PTEN Expt3
Condition	Object number	Object number	Object number
Wild Type	365	312	1468
Trp53-/-	127	677	1168
Trp53-/-;Pten-/- 1.11	663	439	801

Trp53-/-;Pten-/- 1.15	270	293	872

Figure 2 Panels F-G			
	PTENKO on WT Expt1	PTENKO on WT Expt2	PTENKO on WT Expt3
Condition	Object number	Object number	Object number
PTEN_2	403	724	1617
PTEN_5	506	1333	1384
SCR2	490	1087	1786

Figure EV2 Panel A			
	ID8 PTEN 112 Expt1	ID8 PTEN 112 Expt2	ID8 PTEN 112 Expt3
Condition	Object number	Object number	Object number
PTEN 112	663	158	903
PTEN 115	712	762	829
Wild Type	1133	597	766

Figure 3 Panel E-G	ID8 PTEN 115 Inhibitors AKT/LY Expt1	ID8 PTEN 115 Inhibitors AKT/LY Expt2
Condition	Object number	Object number
10uM LY294002	701	1558
15uM AKT Inhibitor II	897	1824
DMSO	544	1217

Figure 3 Panel H-J	PI3K Isoforms Inhibiotrs Expt1	PI3K Isoforms Inhibiotrs Expt2

Condition	Object number	Object number
DMSO	415	620
PI3Kalpha	676	877
PI3Kbeta	926	689
PI3Kdelta	811	723
PI3Kgamma	305	762

Figure 4 Panel A-C	Arf6 KD Expt1	Arf6 KD Expt2	Arf6 KD Expt3
Condition	Object number	Object number	Object number
ARF5_3	1774	659	1361
ARF6_3	1497	1014	667
Scramble	1233	776	958

Figure 5 Panel B	CRISPR Screen Iteration 1 Expt1	CRISPR Screen Iteration 1 Expt2	CRISPR Screen Iteration 1 Expt3	CRISPR Screen Iteration 1 Expt4
Condition	Object number	Object number	Object number	Object number
EGFR	416	674	370	676
ITGA6	625	1239	711	913
ITGB1	586	1850	1650	860
LAMTOR5	342	1099	1230	1199
RAB14	514	847	763	987
SCR	607	1197	595	1037
SCRIB	373	1516	1459	1072
YWHAQ	564	1167	1242	916
	CRISPR Screen Iteration 2 Expt1	CRISPR Screen Iteration 2 Expt2	CRISPR Screen Iteration 2 Expt3	
Condition	Object number	Object number	Object number	
AGAP1	1327	1598	839	
ANO1	818	513	954	
CYTH2	1180	515	1228	
INPPL1	938	1602	1069	
ITGA3	1197	1468	929	

ITGA5	957	531	996	
SCR	1259	909	1182	
SPAG9	1157	1284	941	
	CRISPR Screen Iteration 3 Expt1	CRISPR Screen Iteration 3 Expt2	CRISPR Screen Iteration 3 Expt3	
Condition	Object number	Object number	Object number	
ARHGEF2	770	849	637	
FERMT2	1533	926	812	
FLOT2	1103	844	595	
KRT8	810	279	517	
LAMTOR3	1914	895	752	
LGR4	1039	1255	1135	
SCR	560	1084	916	
TPD52L2	1313	1423	982	
	CRISPR Screen Iteration 4 Expt1	CRISPR Screen Iteration 4 Expt2	CRISPR Screen Iteration 4 Expt3	
Condition	Object number	Object number	Object number	
FLRT2	399	436	862	
FMNL3	491	719	858	
IGF2R	440	676	1144	
MYOF	157	1120	1179	
PCBP2	433	1083	655	
RAB3B	261	848	716	
SCR	35	885	1114	

Figure 5 Panel E,G			
	ITGB1 Deco Expt1	ITGB1 Deco Expt2	ITGB1 Deco Expt3
Condition	Object number	Object number	Object number
ITGB1_2	617	599	1977
ITGB1_3	1075	504	649
ITGB1_4	764	665	1530
SCR2	587	751	378

Figure 5 Panel F,G			
	Agap1 Deco Expt1	Agap1 Deco Expt2	Agap1 Deco Expt3
Condition	Object number	Object number	Object number
AGAP1_2	1272	952	341
AGAP1_3	1786	1159	171
SCR2	1379	890	299

Figure EV5 Panel B-D			
	SecinH3 Expt1	SecinH3 Expt2	SecinH3 Expt3
Condition	Object number	Object number	Object number
115 DMSO	924	584	664
115 SecinH3	197	856	569

Figure 6 Panel E-G			
	mNG AGAP1 OE Expt1	mNG AGAP1 OE Expt2	mNG AGAP1 OE Expt3
Condition	Object number	Object number	Object number
sgNT+mNG	1494	1431	1264
sgAgap1+ mNG	1822	1542	1288
sgAgap1+ mNG AGAP1-L	2498	1685	1116
sgAgap1+ mNG AGAP1-S	2063	943	712

Figure EV5 Panel D			
	ID8 Itgr a5 KO Expt 1	ID8 Itgr a5 KO Expt 2	ID8 Itgr a5 KO Expt 3
Condition	Object number	Object number	Object number

**Itgr a5_1 KO**	**1129**	**1498**	**1178**
**Itgr a5_4 KO**	**977**	**932**	**951**
**Itgr a5_5 KO**	**1407**	**1422**	**749**
**Scr**	**1350**	**838**	**834**
