# Supplementary material for: PTEN deficiency exposes a requirement for an ARF GTPase module for integrin‐dependent invasion in ovarian cancer
Source: EMBO J. 2023 Aug 14;42(18):e113987. doi: 10.15252/embj.2023113987 (PMC10505920; doi:10.15252/embj.2023113987)
Supplement: Supplementary file 4 — Table EV2 [file EMBJ-42-e113987-s012.pdf]

Table EV 2
CRISPR gRNA sequences used in the CRISPR Screen (b is bottom oligo strand) and throughout the paper.

Sequence Name	Sequence (5'-3')
Non_Targeting_2	CAC CGC CGC GCC GTT AGG GAA CGA G
Non_Targeting_2b	AAA CCT CGT TCC CTA ACG GCG CGG C
Non_Targeting_3	CAC CGC GGG CGT CAC CTG CTA GTA A
Non_Targeting_3b	AAA CTT ACT AGC AGG TGA CGC CCG C
Non_Targeting_4	CAC CGT ACA GTT ATA CGT CGC GGT G
Non_Targeting_4b	AAA CCA CCG CGA CGT ATA ACT GTA C
Non_Targeting_6	CAC CGC TCG GGC TAT TCA GCG ATA G
Non_Targeting_6b	AAA CCT ATC GCT GAA TAG CCC GAG C
Pten_2	CAC CGG GTT TGA TAA GTT CTA GCT G
Pten_2b	AAA CCA GCT AGA ACT TAT CAA ACC
Pten_5	CAC CGT CAC CTG GAT TAC AGA CCC G
Pten_5b	AAA CCG GGT CTG TAA TCC AGG TGA
Itga6_1	CAC CGG AGT ATA TAT TTG ACG GAG A
Itga6_1b	AAA CTC TCC GTC AAA TAT ATA CTC C
Itga6_2	CAC CGT CAA ATT CAA TCC GTG TAC A
Itga6_2b	AAA CTG TAC ACG GAT TGA ATT TGA C
Itga6_3	CAC CGC TCC TGA GTA TAC TTT GGC T
Itga6_3b	AAA CAG CCA AAG TAT ACT CAG GAG C
Itga6_4	CAC CGC AAC ACG AAG CAG GAG TCG C
Itga6_4b	AAA CGC GAC TCC TGC TTC GTG TTG C
Itga6_5	CAC CGC AAT CCG TGT ACA AGG TCC C
Itga6_5b	AAA CGG GAC CTT GTA CAC GGA TTG C
Itgb1_1	CAC CGG TCA CAG GAT CGA CCT GCA C
Itgb1_1b	AAA CGT GCA GGT CGA TCC TGT GAC C
Itgb1_2	CAC CGA AGA CAT GGA CGC TTA CTG C
Itgb1_2b	AAA CGC AGT AAG CGT CCA TGT CTT C
Itgb1_3	CAC CGG AGG AAT GTA ACA CGA CTG C
Itgb1_3b	AAA CGC AGT CGT GTT ACA TTC CTC C
Itgb1_4	CAC CGG CGG AGA ATG TAT ACA AGC A
Itgb1_4b	AAA CTG CTT GTA TAC ATT CTC CGC C
Itgb1_5	CAC CGG AGG AAT GTA ACA CGA CTG C
Itgb1_5b	AAA CGC AGT CGT GTT ACA TTC CTC C
Krt8_1	CAC CGC CAG CTT CAA GGG GCT CAA C
Krt8_1b	AAA CGT TGA GCC CCT TGA AGC TGG C
Krt8_2	CAC CGG CTA CAT CAA CAA CCT CCG C
Krt8_2b	AAA CGC GGA GGT TGT TGA TGT AGC C

Krt8_3	CAC CGC CGA CGA GAT CAA CTT CCT C
Krt8_3b	AAA CGA GGA AGT TGA TCT CGT CGG C
Krt8_4	CAC CGT GAA GTT CGT GCC CAG TAC G
Krt8_4b	AAA CCG TAC TGG GCA CGA ACT TCA C
Krt8_5	CAC CGC CTC CGG CAG ATC CAT GAA G
Krt8_5b	AAA CCT TCA TGG ATC TGC CGG AGG C
Lamtor3_1	CAC CGA CAT TAC TCG TGA CCT CCG C
Lamtor3_1b	AAA CGC GGA GGT CAC GAG TAA TGT C
Lamtor3_2	CAC CGC ACA TTA CTC GTG ACC TCC G
Lamtor3_2b	AAA CCG GAG GTC ACG AGT AAT GTG C
Lamtor3_3	CAC CGG ACT GAA CTG CGG GAA CAC C
Lamtor3_3b	AAA CGG TGT TCC CGC AGT TCA GTC C
Lamtor3_4	CAC CGA CTG AAC TGC GGG AAC ACC G
Lamtor3_4b	AAA CCG GTG TTC CCG CAG TTC AGT C
Lamtor3_5	CAC CGA CGG AAT ATA CAG ACC TGG T
Lamtor3_5b	AAA CAC CAG GTC TGT ATA TTC CGT C
Lamtor5_1	CAC CGA GTC ACG TGA TCG AGG TTT G
Lamtor5_1b	AAA CCA AAC CTC GAT CAC GTG ACT C
Lamtor5_2	CAC CGA CCC TGT CGG ATG AGC ACG C
Lamtor5_2b	AAA CGC GTG CTC ATC CGA CAG GGT C
Lamtor5_3	CAC CGC ATA CCA CAG GGA TGT CGG T
Lamtor5_3b	AAA CAC CGA CAT CCC TGT GGT ATG C
Lamtor5_4	CAC CGT CGA TCA CGT GAC TTG ACC G
Lamtor5_4b	AAA CCG GTC AAG TCA CGT GAT CGA C
Lgr4_1	CAC CGT AAG TTG ATT GAG CCC ATT C
Lgr4_1b	AAA CGA ATG GGC TCA ATC AAC TTA C
Lgr4_2	CAC CGT TAA CCT CAT TTC CTA CGG A
Lgr4_2b	AAA CTC CGT AGG AAA TGA GGT TAA C
Lgr4_3	CAC CGA TTA AGT GTT TGC ACG ATA C
Lgr4_3b	AAA CGT ATC GTG CAA ACA CTT AAT C
Lgr4_4	CAC CGC AGG ATT TCT ACT ACG ACT G
Lgr4_4b	AAA CCA GTC GTA GTA GAA ATC CTG C
Lgr4_5	CAC CGC AAC CAT ATT ACC TCA GTC C
Lgr4_5b	AAA CGG ACT GAG GTA ATA TGG TTG C
Myof_1	CAC CGC GGA GTC CAC GCA GGC CCA C
Myof_1b	AAA CGT GGG CCT GCG TGG ACT CCG C
Myof_2	CAC CGT GGC TCG GAG GAT CAC CAA A
Myof_2b	AAA CTT TGG TGA TCC TCC GAG CCA C
Myof_3	CAC CGC TGG CTC GGA GGA TCA CCA A
Myof_3b	AAA CTT GGT GAT CCT CCG AGC CAG C
Myof_4	CAC CGA GAG GAC ACC TAT ACG GAT G
Myof_4b	AAA CCA TCC GTA TAG GTG TCC TCT C

Myof_5	CAC CGG TTT CTC TCC CAG ATA CAC G
Myof_5b	AAA CCG TGT ATC TGG GAG AGA AAC C
Pcbp2_1	CAC CGG ACA CCG GTG TGA TTG AAG G
Pcbp2_1b	AAA CCC TTC AAT CAC ACC GGT GTC C
Pcbp2_2	CAC CGA TGG ACA CCG GTG TGA TTG A
Pcbp2_2b	AAA CTC AAT CAC ACC GGT GTC CAT C
Pcbp2_3	CAC CGG CAC GTA TCA ACA TCT CAG A
Pcbp2_3b	AAA CTC TGA GAT GTT GAT ACG TGC C
Pcbp2_4	CAC CGA CTG AAT CCG GTG TTG CCA T
Pcbp2_4b	AAA CAT GGC AAC ACC GGA TTC AGT C
Rab14_1	CAC CGG TTA CAC GGA GCT ACT ATA G
Rab14_1b	AAA CCT ATA GTA GCT CCG TGT AAC C
Rab14_2	CAC CGC ATA TAA CCA CTT AAG CAG C
Rab14_2b	AAA CGC TGC TTA AGT GGT TAT ATG C
Rab14_3	CAC CGG GGC CGG ACC ACA AAA GAA G
Rab14_3b	AAA CCT TCT TTT GTG GTC CGG CCC C
Rab14_4	CAC CGT GGG CCG GAC CAC AAA AGA A
Rab14_4b	AAA CTT CTT TTG TGG TCC GGC CCA C
Rab14_5	CAC CGG CCG GAC CAC AAA AGA AGG G
Rab14_5b	AAA CCC CTT CTT TTG TGG TCC GGC C
Rab3b_1	CAC CGA GCA TTG AAG GAC TCC TCG T
Rab3b_1b	AAA CAC GAG GAG TCC TTC AAT GCT C
Rab3b_2	CAC CGC ATG TAT GAC ATC ACC AAC G
Rab3b_2b	AAA CCG TTG GTG ATG TCA TAC ATG C
Rab3b_3	CAC CGT CAG ATT AAG ACC TAC TCC T
Rab3b_3b	AAA CAG GAG TAG GTC TTA ATC TGA C
Rab3b_4	CAC CGC ATG TAT GAC ATC ACC AAC G
Rab3b_4b	AAA CCG TTG GTG ATG TCA TAC ATG C
Rab3b_5	CAC CGG TAG TCA AAG TTC TGG TCG G
Rab3b_5b	AAA CCC GAC CAG AAC TTT GAC TAC C
Scrib_1	CAC CGT ACA GAA GAC GAC TAT AAC G
Scrib_1b	AAA CCG TTA TAG TCG TCT TCT GTA C
Scrib_2	CAC CGC TTG GAA GGA CCA TAC CCT G
Scrib_2b	AAA CCA GGG TAT GGT CCT TCC AAG C
Scrib_3	CAC CGT TGT AAC CGG CAC GTG GAG T
Scrib_3b	AAA CAC TCC ACG TGC CGG TTA CAA C
Scrib_4	CAC CGA CGT GGA GTC GGT GGA TAA G
Scrib_4b	AAA CCT TAT CCA CCG ACT CCA CGT C
Scrib_5	CAC CGC CTG AAT GAC GTG TCC CTG C
Scrib_5b	AAA CGC AGG GAC ACG TCA TTC AGG C
Spag9_1	CAC CGG TCC ATA TCC AAT TCA GGA G
Spag9_1b	AAA CCT CCT GAA TTG GAT ATG GAC C

Spag9_2	CAC CGC ATT GAA TCC ACT CCT GAA T
Spag9_2b	AAA CAT TCA GGA GTG GAT TCA ATG C
Spag9_3	CAC CGT TGA ACC TTT ATA TCC ACT G
Spag9_3b	AAA CCA GTG GAT ATA AAG GTT CAA C
Spag9_4	CAC CGA AAG GAT TTA CAG ACG CGA G
Spag9_4b	AAA CCT CGC GTC TGT AAA TCC TTT C
Spag9_5	CAC CGA ATC ACA GGA GAA GCA CCG T
Spag9_5b	AAA CAC GGT GCT TCT CCT GTG ATT C
Tpd52l2_1	CAC CGC TTC TAC AAC TGG AGT CCG G
Tpd52l2_1b	AAA CCC GGA CTC CAG TTG TAG AAG C
Tpd52l2_2	CAC CGG AGT CCG GTG GAC CAC ACC T
Tpd52l2_2b	AAA CAG GTG TGG TCC ACC GGA CTC C
Tpd52l2_3	CAC CGG GAG TCC GGT GGA CCA CAC C
Tpd52l2_3b	AAA CGG TGT GGT CCA CCG GAC TCC C
Tpd52l2_4	CAC CGT TGA CCC AGG TGT GGT CCA C
Tpd52l2_4b	AAA CGT GGA CCA CAC CTG GGT CAA C
Tpd52l2_5	CAC CGT GGA CCA CAC CTG GGT CAA C
Tpd52l2_5b	AAA CGT TGA CCC AGG TGT GGT CCA C
Ywhaq_1	CAC CGA CGG GAA GGT GCG GCA CCA A
Ywhaq_1b	AAA CTT GGT GCC GCA CCT TCC CGT C
Ywhaq_2	CAC CGA CCA ACG GCC GCT TCC ATC T
Ywhaq_2b	AAA CAG ATG GAA GCG GCC GTT GGT C
Ywhaq_3	CAC CGC CCA AGA TGG AAG CGG CCG T
Ywhaq_3b	AAA CAC GGC CGC TTC CAT CTT GGG C
Ywhaq_4	CAC CGC CTC GTG CGC TGA GAC AAA G
Ywhaq_4b	AAA CCT TTG TCT CAG CGC ACG AGG C
Ywhaq_5	CAC CGC ACC CGG CCC AAG ATG GAA G
Ywhaq_5b	AAA CCT TCC ATC TTG GGC CGG GTG C
Agap1_1	CAC CGT GTA GCG TCA CTC CAC TGG T
Agap1_1b	AAA CAC CAG TGG AGT GAC GCT ACA C
Agap1_2	CAC CGC TAC CAT TAC TAC AGC CGA A
Agap1_2b	AAA CTT CGG CTG TAG TAA TGG TAG C
Agap1_3	CAC CGT ACG TCA CCC TGT GCG ACA A
Agap1_3b	AAA CTT GTC GCA CAG GGT GAC GTA C
Agap1_4	CAC CGC ATA AGT TCC ACC AAC CCG A
Agap1_4b	AAA CTC GGG TTG GTG GAA CTT ATG C
Agap1_5	CAC CGC ATA GTA CGT GCA CCT CTT C
Agap1_5b	AAA CGA AGA GGT GCA CGT ACT ATG C
Ano1_1	CAC CGC TGC CCT TCT AAG TAA ACG G
Ano1_1b	AAA CCC GTT TAC TTA GAA GGG CAG C
Ano1_2	CAC CGG ATG TTG GAC CGC ACA GAC G
Ano1_2b	AAA CCG TCT GTG CGG TCC AAC ATC C

Ano1_3	CAC CGG ATG AGG CTC AAC TAC CGA T
Ano1_3b	AAA CAT CGG TAG TTG AGC CTC ATC C
Ano1_4	CAC CGT GAC AAA CGC TTC AGA CGG G
Ano1_4b	AAA CCC CGT CTG AAG CGT TTG TCA C
Ano1_5	CAC CGA CGG AAG GTG GAC TAC ATC T
Ano1_5b	AAA CAG ATG TAG TCC ACC TTC CGT C
Arhgef2_1	CAC CGG CTC ACG CTG GAT TAT GTC T
Arhgef2_1b	AAA CAG ACA TAA TCC AGC GTG AGC C
Arhgef2_2	CAC CGC AAT AAG GGA TTC CAC GGA C
Arhgef2_2b	AAA CGT CCG TGG AAT CCC TTA TTG C
Arhgef2_3	CAC CGT GAG CAA GTC ACC CAA ACG A
Arhgef2_3b	AAA CTC GTT TGG GTG ACT TGC TCA C
Arhgef2_4	CAC CGT GGA TGG TCA CGT TAC ATG C
Arhgef2_4b	AAA CGC ATG TAA CGT GAC CAT CCA C
Arhgef2_5	CAC CGT GGC TAT GTC TCT TAC GAT G
Arhgef2_5b	AAA CCA TCG TAA GAG ACA TAG CCA C
Cyth2_1	CAC CGG TAG ACA CCG TCC TCC ATG G
Cyth2_1b	AAA CCC ATG GAG GAC GGT GTC TAC C
Cyth2_2	CAC CGG GCA AAG GAC AAC ACG TAG C
Cyth2_2b	AAA CGC TAC GTG TTG TCC TTT GCC C
Cyth2_3	CAC CGA TGA ACC GGG GCA TCA ACG A
Cyth2_3b	AAA CTC GTT GAT GCC CCG GTT CAT C
Cyth2_4	CAC CGC TGA GAT TCG GTA CAC CAT G
Cyth2_4b	AAA CCA TGG TGT ACC GAA TCT CAG C
Cyth2_5	CAC CGA GCC ATT GGG GAC TAC CTA G
Cyth2_5b	AAA CCT AGG TAG TCC CCA ATG GCT C
Egfr_1	CAC CGA CCA GAC AGT CAC TCT CTC G
Egfr_1b	AAA CCG AGA GAG TGA CTG TCT GGT C
Egfr_2	CAC CGC ATG AAT AGG CCA ATC CCA A
Egfr_2b	AAA CTT GGG ATT GGC CTA TTC ATG C
Egfr_3	CAC CGG AGA ACC TAG AAA TAA TAC G
Egfr_3b	AAA CCG TAT TAT TTC TAG GTT CTC C
Egfr_4	CAC CGT GGG CCT GAC TAC TAC GAA G
Egfr_4b	AAA CCT TCG TAG TAG TCA GGC CCA C
Egfr_5	CAC CGG ACC GCG AGA ACC ACA CTG C
Egfr_5b	AAA CGC AGT GTG GTT CTC GCG GTC C
Fermt2_1	CAC CGA TCA TGA GCG TCG TAA GTA G
Fermt2_1b	AAA CCT ACT TAC GAC GCT CAT GAT C
Fermt2_2	CAC CGA TTG AAG TAA TGT CAC CCT A
Fermt2_2b	AAA CTA GGG TGA CAT TAC TTC AAT C
Fermt2_3	CAC CGA ATC AAT CAG CTT TAC GAG C
Fermt2_3b	AAA CGC TCG TAA AGC TGA TTG ATT C

Fermt2_4	CAC CGC CAT CAT GAG CGT CGT AAG T
Fermt2_4b	AAA CAC TTA CGA CGC TCA TGA TGG C
Fermt2_5	CAC CGC TGA TGA GCA GGT GTA CCA C
Fermt2_5b	AAA CGT GGT ACA CCT GCT CAT CAG C
Flot2_1	CAC CGA TGG GCA ACT GCC ACA CGG T
Flot2_1b	AAA CAC CGT GTG GCA GTT GCC CAT C
Flot2_2	CAC CGA GTG TCC GAG ATA CAC CAC C
Flot2_2b	AAA CGG TGG TGT ATC TCG GAC ACT C
Flot2_3	CAC CGC CAC ACG GTG GGC CCC AAC G
Flot2_3b	AAA CCG TTG GGG CCC ACC GTG TGG C
Flot2_4	CAC CGC CGA GAC CAG TTT GCC AAG C
Flot2_4b	AAA CGC TTG GCA AAC TGG TCT CGG C
Flot2_5	CAC CGG TTC CTG GGC AAG AAC GTA C
Flot2_5b	AAA CGT ACG TTC TTG CCC AGG AAC C
Flrt2_1	CAC CGC ACC ACC AAG GAA TGT GCG C
Flrt2_1b	AAA CGC GCA CAT TCC TTG GTG GTG C
Flrt2_2	CAC CGA AGG TCC TGA GCA AGT CCG C
Flrt2_2b	AAA CGC GGA CTT GCT CAG GAC CTT C
Flrt2_3	CAC CGC ACA ATA GTC GTC TTT CCG T
Flrt2_3b	AAA CAC GGA AAG ACG ACT ATT GTG C
Flrt2_4	CAC CGA CAA CAT TCA GAC CAT CTC G
Flrt2_4b	AAA CCG AGA TGG TCT GAA TGT TGT C
Flrt2_5	CAC CGC AGC TTA CGG AGG TTT GCG A
Flrt2_5b	AAA CTC GCA AAC CTC CGT AAG CTG C
Fmnl3_1	CAC CGA GAC ATG GAC GTC ATC CTT C
Fmnl3_1b	AAA CGA AGG ATG ACG TCC ATG TCT C
Fmnl3_2	CAC CGC ACT AAA GTC CTA CGG GAG C
Fmnl3_2b	AAA CGC TCC CGT AGG ACT TTA GTG C
Fmnl3_3	CAC CGG GAG TCC ACT AAA GTC CTA C
Fmnl3_3b	AAA CGT AGG ACT TTA GTG GAC TCC C
Fmnl3_4	CAC CGC ATT TGA CAA GCT CCG GTC C
Fmnl3_4b	AAA CGG ACC GGA GCT TGT CAA ATG C
Fmnl3_5	CAC CGA CGG TGC ATT TGA CAA GCT C
Fmnl3_5b	AAA CGA GCT TGT CAA ATG CAC CGT C
Igf2r_1	CAC CGG TTT ATA AGA TCA ACG TCT G
Igf2r_1b	AAA CCA GAC GTT GAT CTT ATA AAC C
Igf2r_2	CAC CGA CGT TGG CCG ACC CAA GGA G
Igf2r_2b	AAA CCT CCT TGG GTC GGC CAA CGT C
Igf2r_3	CAC CGT GAC GTT GGC CGA CCC AAG G
Igf2r_3b	AAA CCC TTG GGT CGG CCA ACG TCA C
Igf2r_4	CAC CGA GGA CAT TGA CTC CAC ACG A
Igf2r_4b	AAA CTC GTG TGG AGT CAA TGT CCT C

Igf2r_5	CAC CGC CAC TGA TCA AGC TAA ATG G
Igf2r_5b	AAA CCC ATT TAG CTT GAT CAG TGG C
Itga3_1	CAC CGA TCG GTA CAC CAA GGT GCT G
Itga3_1b	AAA CCA GCA CCT TGG TGT ACC GAT C
Itga3_2	CAC CGA CCC AGA ACA CCG TAT ACT T
Itga3_2b	AAA CAA GTA TAC GGT GTT CTG GGT C
Itga3_3	CAC CGC TAC TAC TTC GAA CGG AAA G
Itga3_3b	AAA CCT TTC CGT TCG AAG TAG TAG C
Itga3_4	CAC CGA TCG GTG ACA TCA ACC AGG A
Itga3_4b	AAA CTC CTG GTT GAT GTC ACC GAT C
Itga3_5	CAC CGT AGT TCA TGG CAA TGA CGA T
Itga3_5b	AAA CAT CGT CAT TGC CAT GAA CTA C
Itga5_1	CAC CGG TCT TAC ATA GCC ATA GGT G
Itga5_1b	AAA CCA CCT ATG GCT ATG TAA GAC C
Itga5_2	CAC CGG TGT CAA GGA GCT GCC GAA T
Itga5_2b	AAA CAT TCG GCA GCT CCT TGA CAC C
Itga5_3	CAC CGG GGC TTC AAC CTA GAC GCG G
Itga5_3b	AAA CCC GCG TCT AGG TTG AAG CCC C
Itga5_4	CAC CGT GGG GGC TTC AAC CTA GAC G
Itga5_4b	AAA CCG TCT AGG TTG AAG CCC CCA C
Itga5_5	CAC CGC TTC GAG GTG GAA CTC CAA C
Itga5_5b	AAA CGT TGG AGT TCC ACC TCG AAG C
