# Supplementary material for: PTEN deficiency exposes a requirement for an ARF GTPase module for integrin‐dependent invasion in ovarian cancer
Source: EMBO J. 2023 Aug 14;42(18):e113987. doi: 10.15252/embj.2023113987 (PMC10505920; doi:10.15252/embj.2023113987)
Supplement: Supplementary file 5 — Table EV3 [file EMBJ-42-e113987-s025.pdf]

Table EV 3

shRNAs used, Target, species, target sequence and RNAi Consortium (TRC) clone ID provid

mRNA Target	Target Sequence	TRC ID
**Scramble**	CCGCAGGTATGCACGCGT	
**Arf6_3 (human and mouse)**	GCTCACATGGTTAACCTCTAA	TRCN0000048005
**Arf5_3 (human and mouse)**	TGCTGATGAACTCCAGAAGAT	TRCN0000381650
**mARF6_1 (Mouse)**	CCGGAAGGAGAGAAATCCAAA	TRCN0000100335
**mARF6_2 (Mouse)**	CGGCAAGACAACGATCCTGTA	TRCN0000100336
**mARF6_3 (Mouse)**	GCATTACTACACCGGGACCCA	TRCN0000100338
**mARF6_4 (Mouse)**	CAACGTGGAGACGGTGACTTA	TRCN0000100339
**mARF6_5 (Mouse)**	CAACGATCCTGTACAAGTTGA	TRCN0000381041
