# Supplementary material for: PTEN deficiency exposes a requirement for an ARF GTPase module for integrin‐dependent invasion in ovarian cancer
Source: EMBO J. 2023 Aug 14;42(18):e113987. doi: 10.15252/embj.2023113987 (PMC10505920; doi:10.15252/embj.2023113987)
Supplement: Supplementary file 18 — PDF+ [file EMBJ-42-e113987-s022.pdf]

Article

SOURCE DATA | TRANSPARENT PROCESS | OPEN ACCESS

THE EMBO JOURNAL

PTEN deficiency exposes a requirement for an ARF GTPase module for integrin-dependent invasion in ovarian cancer

Konstantina Nikolatou[1,2], Emma Sandilands[1,2], Alvaro Román-Fernández[1,2], Erin M Cumming[1,2], Eva Freckmann[1,2], Sergio Lilla[2], Lori Buetow[2], Lynn McGarry[2], Matthew Neilson[2], Robin Shaw[2], David Strachan[2], Crispin Miller[2], Danny T Huang[1,2], Iain A McNeish[3], James C Norman[1,2], Sara Zanivan[1,2] & David M Bryant[1,2,*]

Abstract

Dysregulation of the PI3K/AKT pathway is a common occurrence in high-grade serous ovarian carcinoma (HGSOC), with the loss of the tumour suppressor PTEN in HGSOC being associated with poor prognosis. The cellular mechanisms of how PTEN loss contributes to HGSOC are largely unknown. We here utilise time-lapse imaging of HGSOC spheroids coupled to a machine learning approach to classify the phenotype of PTEN loss. PTEN deficiency induces PI(3,4,5)P_3-rich and -dependent membrane protrusions into the extracellular matrix (ECM), resulting in a collective invasion phenotype. We identify the small GTPase ARF6 as a crucial vulnerability of HGSOC cells upon PTEN loss. Through a functional proteomic CRISPR screen of ARF6 interactors, we identify the ARF GTPase-activating protein (GAP) AGAP1 and the ECM receptor β1-integrin (ITGB1) as key ARF6 interactors in HGSOC regulating PTEN loss-associated invasion. ARF6 functions to promote invasion by controlling the recycling of internalised, active β1-integrin to maintain invasive activity into the ECM. The expression of the CYTH2-ARF6-AGAP1 complex in HGSOC patients is inversely associated with outcome, allowing the identification of patient groups with improved versus poor outcome. ARF6 may represent a therapeutic vulnerability in PTEN-depleted HGSOC.

Keywords 3D spheroids; ARF6; integrins; Ovarian Cancer; PTEN
Subject Categories Cancer; Cell Adhesion, Polarity & Cytoskeleton
DOI 10.15252/embj.2023113987 | Received 9 March 2023 | Revised 13 July 2023 | Accepted 19 July 2023 | Published online 14 August 2023
The EMBO Journal (2023) 42: e113987

Introduction

The tumour suppressor PTEN is a dual specificity phosphatase regulating both protein tyrosine dephosphorylation (Tamura *et al*, 1998) and dephosphorylation of the 3-positions of phosphatidyl-inositol-3,4,5-*tris*-phosphate (PI(3,4,5)P_3, PIP_3; Maehama & Dixon, 1998) and phosphatidyl-inositol-3,4-*bis*-phosphate (PI(3,4)P_2; Malek *et al*, 2017). In a classical view of lipid phosphatase function, PTEN acts as a buffer to oppose potential overproduction of PIP_3 or PI(3,4)P_2. This ensures the appropriate level of downstream pathway activation and homeostatic responses to PI3K signalling (Myers *et al*, 1998; Cantley & Neel, 1999). In addition to their well-documented roles in cell signalling, such as to the AKT and mTOR pathways (Alessi *et al*, 1996, 1997; Sarbassov *et al*, 2005), the spatial distribution of PIP_3 or PI(3,4)P_2 is integral to their contribution to cell behaviour. Specifically, the location of these two PTEN-regulated PIP species is asymmetric in polarised epithelial cells; PIP_3 is focally enriched at the basolateral surface (Gassama-Diagne *et al*, 2006) while PI(3,4)P_2 is located at the apical domain (Roman-Fernandez *et al*, 2018). PTEN is present at the apicolaterally localised tight junction, which is a boundary point between these asymmetric lipids (Martin-Belmonte *et al*, 2007).

PTEN gene deletion can be found in a number of cancers, particularly high-grade serous ovarian carcinoma (HGSOC) and prostate cancers (Taylor *et al*, 2010; Patch *et al*, 2015). Mutation of *PTEN* also occurs at a modest level in most cancers, with glioblastoma and uterine cancers presenting frequent *PTEN* mutation (The Cancer Genome Atlas [TCGA], cBioPortal; Cerami *et al*, 2012; Gao *et al*, 2013). Mutation of the PIP_3-producing *PIK3CA*, in contrast, is a frequent event in a number of cancers (Lawrence *et al*, 2014). This emphasises that dysregulation of the PI3K-PTEN axis is a common event in several cancer types (Hammond & Balla, 2015). Despite this, exactly how these lipid kinase and phosphatases enact the cellular changes that contribute to tumorigenesis remains largely unclear. For instance, given the polarised nature of these lipids, does the loss of *PTEN* allow for enhanced signalling function at the normal site of PIP_3 in the cell (the basolateral domain) or is PIP_3 produced at ectopic sites, allowing for *de novo* functions? Clarifying

1 School of Cancer Sciences, University of Glasgow, Glasgow, UK
2 The CRUK Beatson Institute, Glasgow, UK
3 Department of Surgery and Cancer, Ovarian Cancer Action Research Centre, Imperial College London, London, UK
*Corresponding author. Tel: +44 0141 330 8597; E-mail: david.bryant@glasgow.ac.uk

© 2023 The Authors. Published under the terms of the CC BY 4.0 license

such fundamental questions may inform whether targeting classical downstream targets of PI3K-PIP_3 signalling versus potential dependencies that manifest particularly when *PTEN* is lost, show therapeutic viability.

The spatial distribution of PIP species has been revealed by the use of domains of proteins that show preferential PIP affinity fused to fluorescent proteins as indirect reporters for PIP location (Watt *et al*, 2002; Kutateladze, 2010; Shewan *et al*, 2011; Hammond & Balla, 2015). For example, fusion to fluorescent proteins (e.g. GFP) of the pleckstrin homology (PH) domain from the cytohesin (CYTH) family of GTP exchange factors (GEFs) for ARF GTPases, such as ARNO/CYTH2, (e.g. GFP-PH-CYTH2) can be an exquisite sensor for PIP_3 location. Splicing of these PH-CYTH domains alters their lipid specificity, wherein a di-glycine splice variant of the PH domain (PH-$CYTH2^{2G}$) preferentially binds PIP_3, while a tri-glycine splice variant (PH-$CYTH2^{3G}$) associates with $PI(4,5)P_2$ (Klarlund *et al*, 2000). This illustrates how using lipid-preferential binding domains in such reporters allows the detection of PIP distribution.

Although the PH domains of CYTH-type ARF GEFs have been extensively used as probes for PIP_3 localisation, the extent to which they are required to enact PIP_3 downstream signalling has mostly been neglected. Recent work identifies that the PIP_3-specific variant of CYTH1 is required for signalling from c-Met to induce migration (Ratcliffe *et al*, 2019). Moreover, both PI4- and PI5-kinases are effectors of ARF GTPases themselves (Brown *et al*, 1993; Cockcroft *et al*, 1994; Honda *et al*, 1999; Tsai *et al*, 2017), highlighting that ARF GTPases are intimately involved in maintaining and effecting PIP homeostasis. ARF GTPases are evolutionarily conserved membrane trafficking regulators, controlling many aspects of this process, such as turnover and recycling of receptor tyrosine kinases, cell–cell and cell-matrix adhesion proteins (Palacios *et al*, 2002; Powelka *et al*, 2004; D'Souza-Schorey & Chavrier, 2006; Loskutov *et al*, 2015). ARF GTPases are therefore well-placed to respond to changes in phospholipid metabolism that occur frequently in cancer and enact the cellular alterations that lead to invasive activity.

Here, we used a murine-derived model of HGSOC (ID8 cells) to examine the cellular consequences of *Pten* loss on collective cancer cell behaviour, using machine learning to detect phenotypic changes across multiday time-lapse spheroid imaging. We identify that *Pten* loss induces PIP_3-rich and -driven invasive protrusions into the extracellular matrix (ECM), which leads to invasive activity. We uncover that ARF6 is essential for this process. Through CRISPR-mediated ARF6 interactor screening, we identify that ARF6 acts in concert with the ARFGAP protein AGAP1 to promote recycling of active integrin in protrusions and drive invasion. Levels of this ARF6 module predict clinical outcome in ovarian cancer patients. Our approach therefore uncovers an ARF6 vulnerability upon PTEN loss in collective cancer cell behaviour in ovarian cancer.

Results

PTEN loss in the tumour epithelium and association with poor patient survival

To understand how *PTEN* expression levels are altered in ovarian cancer (OC), we examined *PTEN* mRNA in tumour epithelium and stroma. In three independent data sets of laser capture microdissected (LCM), ovarian tumours separated into epithelium and stroma (Bowen *et al*, 2009a; Data ref: Bowen *et al*, 2009b; Lili *et al*, 2013a; Data ref: Lili *et al*, 2013b; Yeung *et al*, 2013a; Data ref: Yeung *et al*, 2013b). *PTEN* mRNA was significantly decreased in Tumour versus Normal ovarian epithelium, whereas stromal *PTEN* levels were inconsistently altered (Fig 1A–C). As such, an epithelial-specific downregulation of PTEN at the mRNA level was evident in those data sets. Across bulk Ovarian Cancer tumour data sets, which included epithelium and stroma, three of six independent data sets showed decreased *PTEN* mRNA in Tumour versus Normal samples, with nonsignificant data sets all possessing a low number of normal samples (n = 4–6; Fig 1D; Wu *et al*, 2007a; Data ref: Wu *et al*, 2007b; Bonome *et al*, 2008a; Data ref: Bonome *et al*, 2008b; King *et al*, 2011a; Data ref: King *et al*, 2011b; Elgaaen *et al*, 2012a; Data ref: Elgaaen *et al*, 2012b; Hill *et al*, 2014a; Data ref: Hill *et al*, 2014b; Yamamoto *et al*, 2016a; Data ref: Yamamoto *et al*, 2016b). In The Cancer Genome Atlas (TCGA) Ovarian Cancer data set (Cerami *et al*, 2012; Gao *et al*, 2013), 73% of samples possessed *TP53* mutation and consequently *PTEN* alteration occurred frequently with *TP53* alteration. Low *PTEN* mRNA was poorly associated with *PTEN* copy number changes and modestly associated with low PTEN protein levels (Fig 1E). Comparing high levels of *PTEN* mRNA (Quartile 4, Q4) to lower (Q1 + 2 + 3) levels did not distinguish overall survival in ovarian cancer patients (Fig 1F). Yet, an 11-month (P = 0.0019) increase in survival was observed in high (Q4) versus not (Q1 + 2 + 3) PTEN protein levels (Fig 1G). Accordingly, while low *PTEN* mRNA patients (Q1 vs. Q4) displayed significant, but modest AKT activation (pT308, pS473) (Fig 1H), similar comparisons using PTEN protein levels revealed a significant and robust PI3K-AKT signalling signature in low PTEN protein patients (Fig 1I). Therefore, low *PTEN* protein levels in ovarian cancer are associated with upregulated AKT signalling and poor overall survival.

Pten loss induces modest effects in 2D culture

We aimed to model how PTEN loss in the epithelium affects tumour cell behaviour. A mutant *TP53* is a defining feature of HGSOC and is therefore an almost universal characteristic of the disease. An approximate 30–35% of the observed *TP53* mutations are classified as null (Yemelyanova *et al*, 2011) with loss of wild-type P53 signalling observed regardless of mutation type (Hoadley *et al*, 2014). As such *TP53*-null models of HGSOC constitute good representations of the clinical situation. Based on the above and the fact that patient outcome is not stratified based on TP53 mutation type (Ahmed *et al*, 2010), we utilised ID8 ovarian cancer cells knocked out (KO) for *Trp53* and *Pten*, alone or in combination (Fig EV1A; including multiple clones of the double KO, dKO; Walton *et al*, 2016, 2017). As a control, we made use of a wild-type (WT) ID8 cell line, derived from Parental ID8 cells upon treatment with CRISPR plasmids containing the sgRNA sequence that produced the $Trp53^{-/-}$ subline but had in this specific case failed to introduce *Trp53* KO (Walton *et al*, 2016). While *Pten* KO alone resulted in a trend towards increased AKT activation (pS473), this was not statistically significant. However, *Pten* KO, in combination with *Trp53* KO, significantly increased AKT activation (Fig EV1B–H), indicating synergy between *Pten* and *Trp53* depletion in stabilising pAKT. By examining pS473-Akt staining on cells segmented into membrane, cytoplasm and perinuclear regions, PTEN loss was observed to

© 2023 The Authors

result in the activation of pAKT at the cell cortex in cells grown in two-dimensional (2D) contexts (Fig EV1I and J, arrowheads). We noted that ID8 cells in 2D displayed a mixed morphology that could be classified into three categories: Cobblestone, Round and Elongated. *Trp53* KO alone did not significantly affect cell shape compared with parental (WT) cells. In contrast, *Pten* co-depletion decreased the frequency of being Round and induced a general elevation in both other classes without a consistent increase in one or the other (Fig EV1K and L). Examination of proliferation or apoptosis, using puromycin treatment as a control for cell death, revealed that neither $Trp53^{-/-}$ or $Trp53^{-/-}$;$Pten^{-/-}$ dKO affected global growth or death in 2D culture (Fig EV1M and N). Together, this revealed that despite a robust activation of pAKT, p53 and PTEN loss do not manifest in major phenotypes in the examined conditions in cells in 2D culture.

PTEN loss induces ECM invasion

We next examined whether PTEN loss phenotypes may involve altered collective morphogenesis using multiday time-lapse imaging of single cells plated in ECM gels that developed into three-dimensional spheroids (Fig 2A). While parental ID8 spheroids (WT) underwent proliferation and organisation into spherical multicellular objects with infrequent protrusive activity into the ECM (Fig 2B,

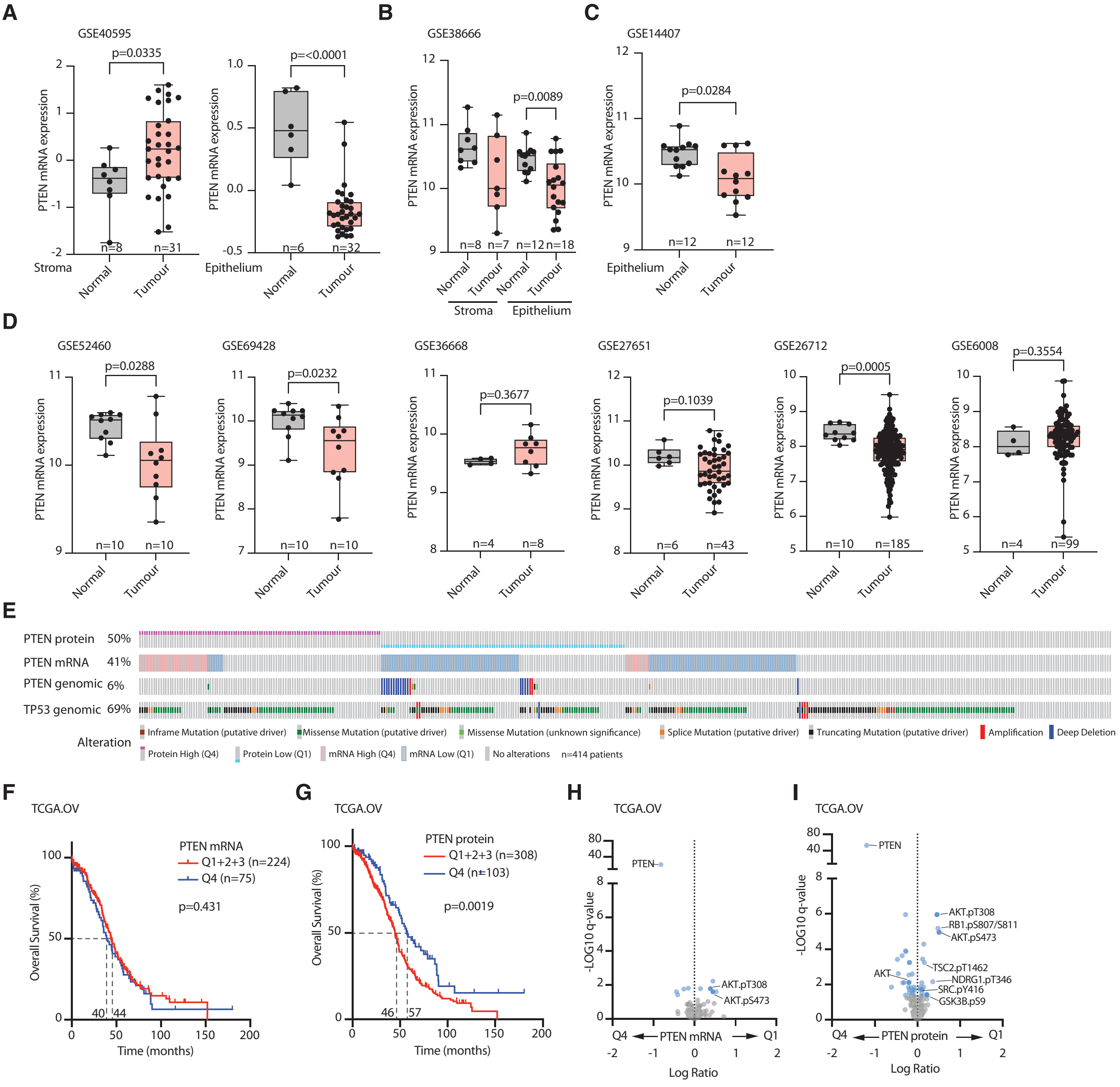

Figure 1.

Figure 1. Loss of *Pten* in HGSOC epithelium is associated with poor outcome.

A–C *PTEN* mRNA levels in LCM normal ovarian surface epithelium versus high-grade serous ovarian cancer (HGSOC) epithelium or normal ovarian stroma versus ovarian cancer-associated stroma. Data sets; (A) GSE40595, (B) GSE38666, (C) Epithelium only, GSE14407. Sample size (*n*) and *P*-values, (Mann–Whitney) annotated, whiskers Min–Max, line at median.

D *PTEN* mRNA levels in normal ovarian surface epithelium versus tumour. Data set ID, sample size (*n*) and *P*-values (Mann–Whitney) annotated, whiskers Min–Max, line at median.

E Copy Number, mRNA, protein level changes and mutations identified across *PTEN* and *TP53* in the TCGA data set of OC. Sample size (*n*) = 414 patients.

F Overall survival (% patients, months) of OC patients. Highest quartile (Q4) versus combination of quartiles 1–3 (Q1 + 2 + 3), *PTEN* mRNA (TCGA, OV). Median survival (40 and 44 months), sample size (*n*) and *P*-value, Log-rank test (Mantel–Cox) annotated.

G Overall survival (% patients, months) of OC patients. Highest quartile (Q4) versus combination of quartiles 1–3 (Q1 + 2 + 3), PTEN protein. Reverse Phase Protein Array Data, TCGA OV. Median survival (46 and 57 months), sample size (*n*) and *P*-value, Log-rank test (Mantel-Cox).

H Differential abundance (*x*, Log Ratio between conditions; *y*, $-\text{Log}_{10}$ *q*-values) of Reverse Phase Protein Array data (TCGA, OV) in patient grouped by *PTEN* mRNA, High (Q4) versus Low (Q1). Significant, blue ($-\text{Log}_{10}$ *q*-values > 1.3); AKT signalling pathway, labelled.

I Differential abundance (*x*, Log Ratio between conditions; *y*, $-\text{Log}_{10}$ *q*-values) of proteins in PTEN High (Q4) versus PTEN Low (Q1) protein samples. Reverse Phase Protein Array Data, TCGA OV. Significantly altered components in AKT signalling pathway labelled ($-\text{Log}_{10}$ *q*-value > 1.3).

Source data are available online for this figure.

arrowheads), *Trp53*$^{-/-}$ spheroids exhibited modest protrusive activity. In contrast, *Trp53*$^{-/-}$;*Pten*$^{-/-}$ spheroids displayed an enlarged, hyperprotrusive phenotype (Fig 2B; Movie EV1). This suggests that, in contrast to mild phenotypes in 2D culture, the phenotype of PTEN loss robustly manifests in 3D contexts where ECM is present.

To develop a quantitative measure of altered morphogenesis, we used a CellProfiler and CellProfiler Analyst-based *Fast Gentle Boosting* machine learning pipeline. Upon imaging, this pipeline could classify hundreds-to-thousands of spheroids per condition into Spherical and Hyper-protrusive (Freckmann *et al*, 2022). The steps involved were as follows: (i) phase-contrast images of segmented spheroids were measured for texture, granularity, shape, size and movement features in tracked objects over multiple days; (ii) a high-accuracy classifier was applied to determine in-focus objects; (iii) out-of-focus objects were removed; (iv) a second high-accuracy classification into Spherical and Hyper-protrusive spheroids was applied; and (v) the frequency of phenotypes over time across different manipulations were calculated (Fig 2C).

We used bubble heatmaps for size (Area) of the spheroids and the proportion of objects across genotypes classified as either Spherical or Hyper-protrusive. This allows simultaneous presentation of (i) the magnitude of change in phenotypes, (ii) the statistical significance of each comparison and (iii) whether the magnitude of the effect was reproducible across independent experiments (Freckmann *et al*, 2022). In the bubble heatmaps, experiments are presented in 6-h time chunks, representing the average value of phenotype proportion during each interval. The control condition is presented as *z*-scored normalised values at each time period (Fig 2D). For treatments, compared with the control condition, the colour of the circle corresponds to Log_2 fold-change to control and the circle is scaled according to the statistical significance of the comparison (Cochran–Mantel Haenszel test with Bonferroni adjustment), with larger circle sizes corresponding to smaller *P*-values. Additionally, the presence of a black dot in the centre corresponds to effect magnitude, demonstrating homogeneity across biological replicates as determined by a nonsignificant *P*-value using the Breslow–Day statistical test (with Bonferroni adjustment). Application of this approach revealed that KO of *Pten*, whether in combination with *Trp53* loss (Fig 2D and E) or alone (Fig 2F and G), and across multiple clones (Figs 2D and E and EV2A), results in the induction of a hyperprotrusive, invasive spheroid phenotype (Fig 2H, arrowheads; Movies EV2 and EV3).

Confirmation of this increased activity upon *Pten* KO occurred in orthogonal 3D invasion assays with monolayers plated on ECM, wounded and then further overlaid with more ECM (Fig EV2B–D; Movie EV4). Tracking of the directionality of invasive front of the wound edge revealed an increase in additional depth and persistence occurred upon co-loss of *Pten* compared with *Trp53* alone (Fig EV2E). Notably, while invasion of parental cells into ECM occurred via infrequent chains of cells following a leader cell, upon *Pten* KO most cells at the leading edge displayed leader cell behaviours (Fig EV2C and F, arrowheads; Fig EV2G). Therefore, loss of *Pten* is associated with desynchronised leader cell activity into the ECM, leading to a hyperprotrusive, persistently invasive phenotype.

Pten loss-induced invasion is associated with PIP_3 enrichment at invasive protrusion tips

Class I PI3-kinases (PI3Ks) add a 3-phosphate group to PI(4,5)P_2, generating PIP_3. PTEN reverses PI3K activity by removing this 3-phosphate group. We thus examined how *Pten* loss controls PI(4,5)P_2 and PIP_3 distribution in 3D contexts. In poorly protrusive *Trp53*$^{-/-}$ spheroids, probes for PI(4,5)P_2 (mNeonGreen [mNG]-tagged PH-PLCδ1) and PIP_3 (mNG-PH-CYTH3^{2G}) localised cortically, as well as in the nucleus in the case of PIP_3 (Fig 3A). In wounded invasive monolayers of *Trp53*$^{-/-}$ cells, PI(4,5)P_2 and PIP_3 were not obviously enriched at protrusion tips (Fig EV3A–D, arrowheads). However, in *Trp53*$^{-/-}$;*Pten*$^{-/-}$ dKO cells, a pool of PIP_3 was prominently located to the tips of protrusions in both spheroids and invasive monolayers (Figs 3A and B, and EV3C and D, arrowheads). Accordingly, the tips of the invasive protrusions in the *Trp53*$^{-/-}$;*Pten*$^{-/-}$ spheroids were highly enriched for the PIP_3 effector pAKT (S473), prior to F-actin enrichment (Fig EV3E and F, arrowheads). This suggest that the elevated protrusive activity upon PTEN loss is associated with an elevation of PIP_3 and pAKT (S473) at the tip of protrusions.

As low PTEN protein patient tumours displayed a PI3K-AKT substrate phosphorylation activation signature (Fig 1I), we examined the requirement for PI3K-AKT signalling in the hyperprotrusive PTEN KO phenotype. PIP_3 can be generated from PI(4,5)P_2 through four Class-I PI3Ks (α, β, γ, δ) (Fig 3C). Pan inhibition of these PI3Ks (pan-PI3K-i; LY294002) or AKT (AKT-I; AKT Inhibitor II) (Fig 3D) abolished protrusion formation, resulting in smaller spheroids with

 © 2023 The Authors

upregulation of the Spherical phenotype and loss of Hyper-protrusive classification (Fig 3E–G; Movie EV5). Deconvolution of class-I PI3K contribution using isoform-preferential inhibitors revealed a major contribution of PI3Kβ to invasion and growth across the entire imaging period, and a more modest effect of PI3Kα at earlier timepoints (1–36 h; Fig 3D and H–J; Movie EV6). Interestingly, PI3Kδ inhibition resulted in a transient elevation of spheroid size that nonetheless did not change the Hyper-protrusive behaviour, suggesting an uncoupling between proliferation and invasiveness under these conditions. Notably, PI3Kβ was found localised at the tips of the invasive protrusions (white/black arrowheads), prior to F-actin (yellow arrowheads), in *Trp53*$^{-/-}$;*Pten*$^{-/-}$ spheroids (Fig 3K and L). This mirrors pAKT (S473) and PIP_3 localisation to the immediate tip of protrusions (Figs 3A and B, and EV3E and F). Therefore, in this system, and similar to Ovarian Cancer patients with low *PTEN* (Fig 1I), loss of *Pten* is associated with PI3Kβ-AKT signalling, which is localised to the tip of, and required for, invasive protrusions.

The small GTPase ARF6 is required for *Pten* loss-mediated ECM invasion

We reported that in prostate cancer cells, the small GTPases ARF5 and ARF6 are required to maintain invasive protrusion formation in 3D culture (Nacke *et al*, 2021). In ID8 *Trp53*$^{-/-}$;*Pten*$^{-/-}$ dKO cells, stable lentiviral shRNA to *Arf5* or *Arf6* (Fig EV4A and B) revealed a moderate effect of *Arf5* depletion on Hyper-protrusiveness and spheroid size (Fig 4A–C), but no effect on invasion for ECM-

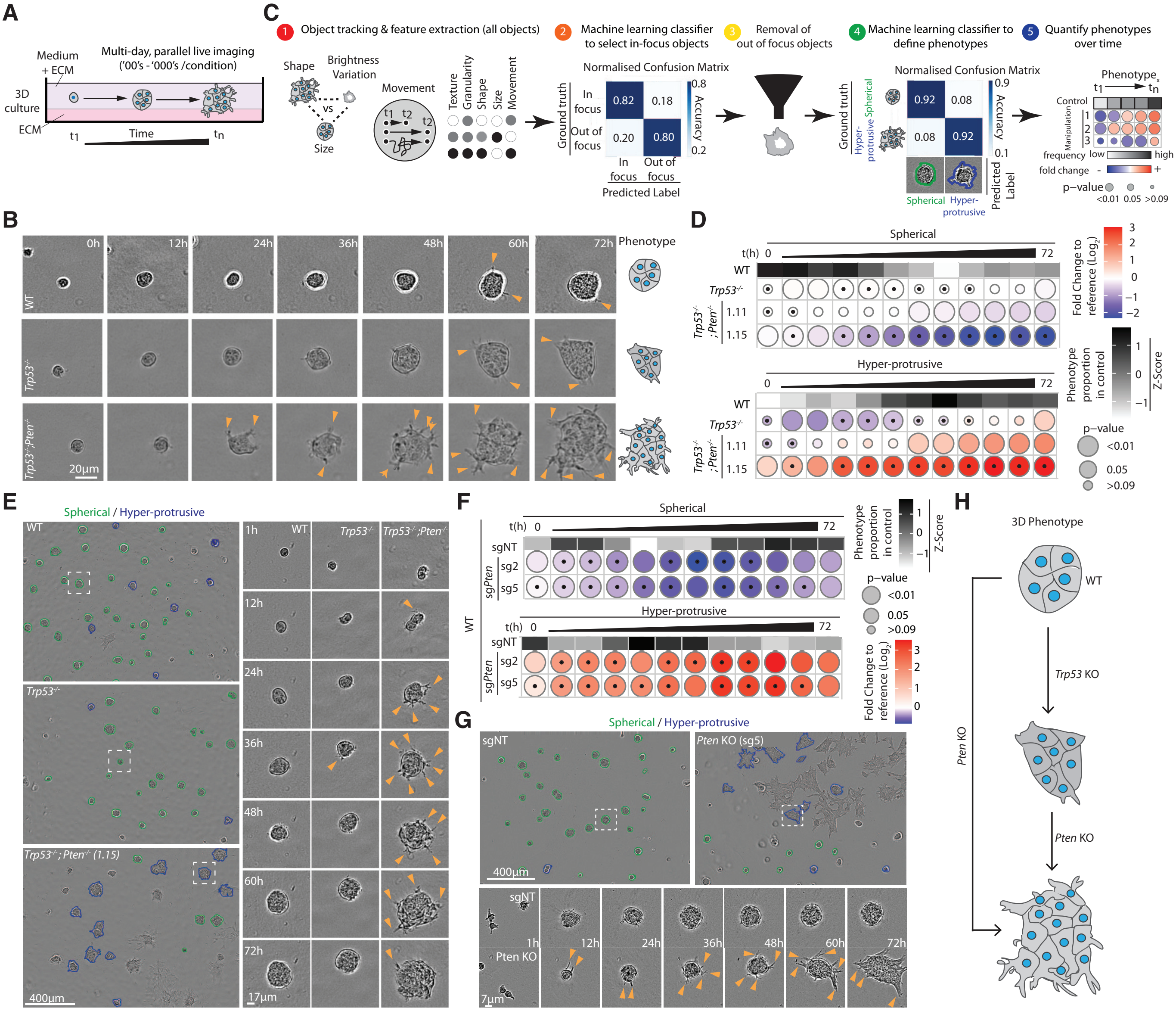

Figure 2.

Figure 2. Loss of *Pten* is associated with collective invasion into ECM in a spheroid assay.

A Schema, imaging of ID8 spheroids in three-dimensional (3D) culture over time. Single cell suspensions were seeded onto and overlaid with ECM diluted in medium and then live-imaged.

B Time series, showing a representative spheroid for each genotype, 12 h intervals. Arrowheads, protrusions into ECM. Scale bar, 20 μm. Right, cartoon of phenotype.

C Schema, analysis pathway to classify ID8 3D phenotypes. (1) Phase contrast images were segmented using CellProfiler. Shape, size, movement, texture, granularity and brightness measurements were extracted for each object. (2) Based on the measurements obtained from live imaging for each individual spheroid, we used CellProfiler Analyst and user-supervised machine learning (FastGentle Boosting algorithm) to construct rules based on which the objects would be classified as "In Focus" or "Out-of-focus". (3) The later were filtered out of the data set. (4) Additional machine learning was used to classify remaining 'In-focus' objects as "Hyper-protrusive" or "Spherical," with high accuracy. (5) Data analysis pipeline was used to quantify the $\log_2$ fold-change of each phenotype relative to control for each subline.

D Frequency of Spherical and Hyper-protrusive phenotypes in ID8 sublines, 6 h time intervals over 72 h. Heatmap (grayscale)—phenotype proportion (*z*-score) in control (Wild-type [WT]). Heatmap (blue-red)—$\log_2$ fold change from control. *P*-values, bubble size (Cochran–Mantel–Haenszel test with Bonferroni adjustment). Black dot, homogenous effect across independent experiments (Breslow-Day test, Bonferroni adjustment, non-significant). *N* = 3 independent experiments, 3–5 technical replicates/experiment. Total spheroid number per condition, Table EV1.

E Representative phase contrast images of spheroids described in (D). Outlines pseudocoloured for classification (Spherical, green; Hyper-protrusive, blue). Scale bars, 400 or 17 μm, as indicated. Magnified individual spheroids from boxed regions at indicated timepoints. Arrowheads, protrusions into ECM.

F Frequency of Spherical and Hyper-protrusive phenotypes in ID8 parental spheroids expressing sgNT, sg2 *Pten* or sg5 *Pten*, 6-h time intervals over 72 h. Heatmap (grayscale)—phenotype proportion (*z*-score) in control (sgNT). Heatmap (blue-red)—$\log_2$ fold change from control. *P*-values, bubble size (Cochran–Mantel–Haenszel test with Bonferroni adjustment). Black dot, homogenous effect across independent experiments (Breslow–Day test, Bonferroni adjustment, non-significant). *N* = 3 independent experiments, 2–6 technical replicates/experiment. Total spheroid number per condition, Table EV1.

G Representative phase contrast images of spheroids described in (F). Outlines pseudocoloured for classification (Spherical, green; Hyper-protrusive, blue). Magnified individual spheroids from boxed regions at indicated timepoints. Arrowheads, protrusions into ECM. Scale bars, 400 or 17 μm, as indicated.

H Schema, phenotypes of ID8 spheroids with analysed genotypes.

Source data are available online for this figure.

embedded, wounded monolayers (Fig EV4C and D). In contrast, *Arf6* stable depletion phenocopied PI3Kβ inhibition, resulting in reduced Area, a near-complete loss of Hyper-protrusiveness in spheroids (Fig 4A–C), and strongly attenuated invasion (Fig EV4C and D, arrowheads, invading cells; Movie EV7). *Arf5* or *Arf6* depletion did not affect AKT activation (pS473) (Fig EV4A and B), suggesting that these GTPases act downstream of PIP_3 generation. Validation of the *Arf6* depletion effect across five additional *Arf6*-targeting shRNAs revealed that the Hyper-protrusive activity of *Trp53*$^{-/-}$;*Pten*$^{-/-}$ dKO spheroids highly correlated with ARF6 levels (R^2 = 0.7787, *P* = 0.0199; Fig EV4E–G).

We examined whether ARF6 localisation was modulated by PTEN loss. In *Trp53*$^{-/-}$ leader cells of invasive, ECM-embedded monolayers, ARF6-mNeonGreen (mNG) localised prominently at cell–cell contacts (white arrowheads), cell-ECM contacts (blue arrowheads), as well as to intracellular pools (green arrowheads) (Fig 4D). In contrast, in *Trp53*$^{-/-}$;*Pten*$^{-/-}$ dKO invading monolayers, while the cell–cell labelling (white arrowheads) of ARF6-mNG was still present, a new pool of ARF6-mNG could be observed at invasive protrusion tips (yellow arrowheads) (Fig 4D and E). The same pattern was evident in *Trp53*$^{-/-}$;*Pten*$^{-/-}$ dKO spheroids (Fig 4F and G) mirroring PIP_3-Akt location upon *Pten* loss (Figs 3A and B, and EV3E and F). Collectively, this suggests a role for ARF6 in regulating invasive protrusion tip formation upon PTEN loss.

Identification of ARF6-proximal protein networks

We examined how ARF6 is a vulnerability in *Pten*-null cells. We observed no consistent alteration in global levels of *Arf6* mRNA (Fig EV4H), protein (Fig EV4I), or GTP-loading (Fig EV4J) in *Trp53*$^{-/-}$ or *Trp53*$^{-/-}$;*Pten*$^{-/-}$ cells compared with parental cell (WT), including multiple clones of the latter genotype. We therefore examined whether, rather than ARF6 activation or levels being altered, ARF6 interaction partners may change upon *Trp53* and *Pten* loss.

We identified ARF6-proximal proteins through ARF6 fusion to the promiscuous biotin ligase TurboID (Branon *et al*, 2018) (Figs 4H and EV4K), in WT, *Trp53*$^{-/-}$ and *Trp53*$^{-/-}$;*Pten*$^{-/-}$ cells, including three clones of the latter genotype and across four independently repeated experiments. This allowed robust statistical support of identified ARF6-proximal proteins by mass spectrometry (MS) proteomic analysis. ARF6-TurboID localisation mirrored that of ARF6-mNG, occurring at cell–cell and cell-ECM contacts in 2D cells (Fig EV4L, black and green arrowheads, respectively) and allowed rapid labelling of ARF6-proximal proteins upon biotin addition (Fig EV4M). Gene Ontology Cell Compartment (GOCC) analysis of ARF6-proximal proteins in *Trp53*$^{-/-}$;*Pten*$^{-/-}$ cells compared with TurboID alone in the same cells, identified significant enrichment for proteins involved in cell projections, filopodia and ECM interactions (Fig EV4N). Cytoscape and STRING database analysis identified a highly interconnected network of ARF6-proximal proteins (Fig 4I–L), including a singular ARF GEF, the PIP_3-regulated CYTH2/ARNO protein, and a singular ARF GAP, AGAP1 (Nie *et al*, 2002). In addition, networks centred around proteins with known functions of Rho GTPases, cell-ECM adhesion, cell–cell adhesion, endocytosis and endosomal system, and cytoskeleton and migration, as well as others with less reported connections.

We examined how ARF6-proximal proteins changed upon *Trp53* and *Pten* loss, dividing interactors into three categories: those that were largely unchanged, strong interactors across examined genotypes (Fig 4J), changing strong interactors (Label-Free Quantitation [LFQ] intensity difference > 1.2) altered in *Trp53*$^{-/-}$ and/or *Trp53*$^{-/-}$;*Pten*$^{-/-}$ cells compared to the parental (WT) genotype (Fig 4K), and weak interactors (LFQ intensity difference < 1.2) altered in *Trp53*$^{-/-}$ and/or *Trp53*$^{-/-}$;*Pten*$^{-/-}$ cells compared with the parental (WT) genotype (Fig 4L). We include this third category to entertain interactors that may only bind in the *Trp53* and/or *Pten*

 © 2023 The Authors

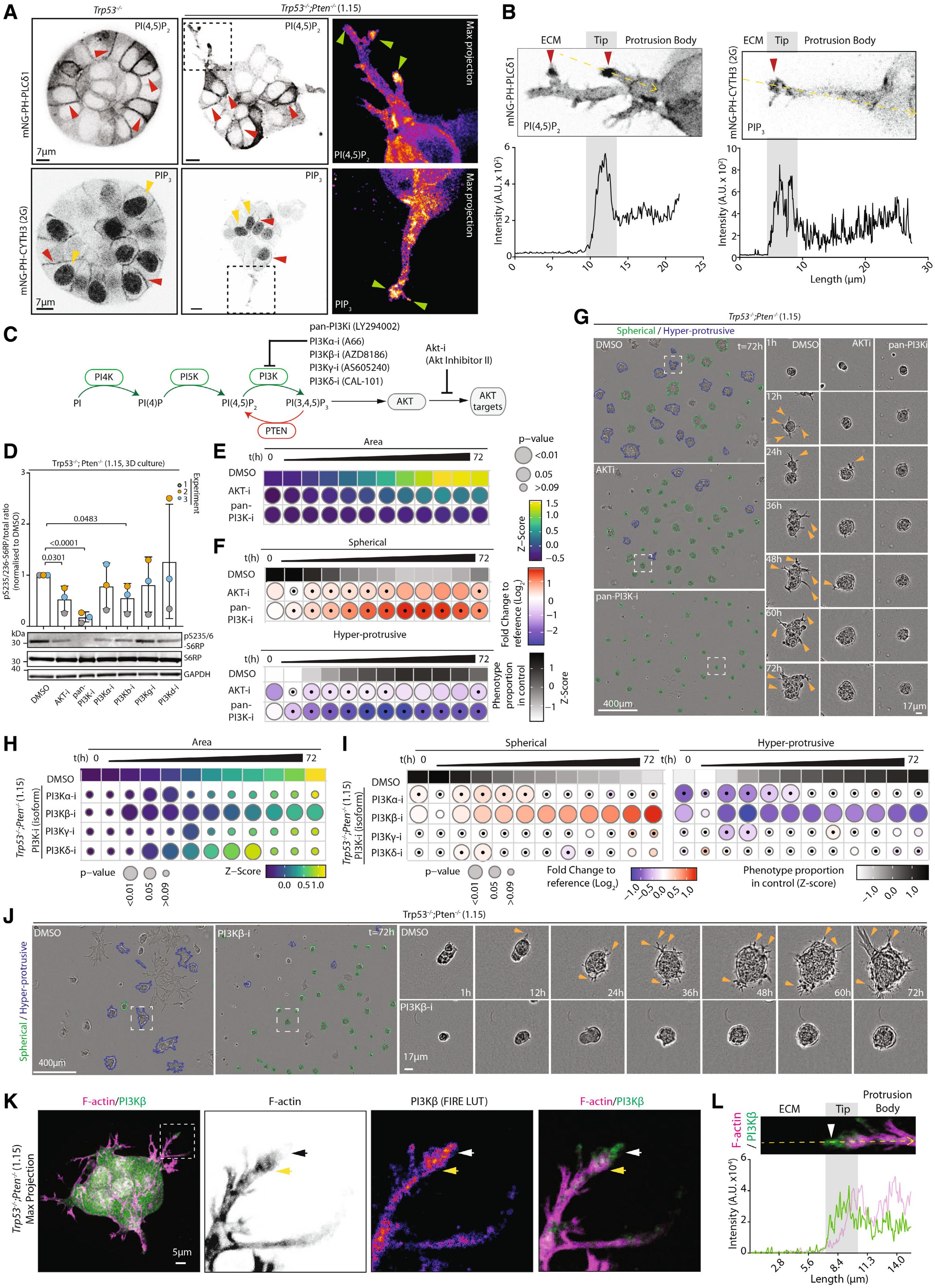
A
Trp53-/-
Trp53-/-;Pten-/- (1.15)
mNG-PH-PLCδ1
mNG-PH-CYTH3 (2G)
PI(4,5)P2
PIP3
Max projection
7µm
B
ECM
Tip
Protrusion Body
Intensity (A.U. x 10²)
Length (µm)
C
pan-PI3Ki (LY294002)
PI3Kα-i (A66)
PI3Kβ-i (AZD8186)
PI3Kγ-i (AS605240)
PI3Kδ-i (CAL-101)
Akt-i
(Akt Inhibitor II)
PI4K
PI5K
PI3K
PI
PI(4)P
PI(4,5)P2
PI(3,4,5)P3
AKT
AKT targets
PTEN
D
Trp53-/-; Pten-/- (1.15, 3D culture)
pS235/236-S6RP/total ratio (normalised to DMSO)
Experiment
0.0483
<0.0001
0.0301
pS235/6 -S6RP
S6RP
GAPDH
kDa
DMSO
AKT-i
pan-PI3K-i
PI3Kα-i
PI3Kb-i
PI3Kg-i
PI3Kd-i
E
Area
t(h)
p-value
<0.01
0.05
>0.09
Z-Score
F
Spherical
Hyper-protrusive
Fold Change to reference (Log2)
Phenotype proportion in control
G
Trp53-/-;Pten-/- (1.15)
Spherical / Hyper-protrusive
t=72h
AKTi
pan-PI3K-i
pan-PI3Ki
400µm
17µm
H
Trp53-/-;Pten-/- (1.15) PI3K-i (isoform)
PI3Kα-i
PI3Kβ-i
PI3Kγ-i
PI3Kδ-i
I
Phenotype proportion in control (Z-score)
J
PI3Kβ-i
K
F-actin/PI3Kβ
F-actin
PI3Kβ (FIRE LUT)
Max Projection
5µm
L
F-actin / PI3Kβ
Intensity (A.U. x10⁴)

Figure 3.

Figure 3. PI3K-AKT dependence of collective invasion.

A Confocal images (single slice) of *Trp53*$^{-/-}$ or *Trp53*$^{-/-}$;*Pten*$^{-/-}$ (1.15) spheroids expressing mNeonGreen-tagged (mNG) biosensors for PI(4,5)P_2 (PH-PLCδ1) or PIP_3 (CYTH3^{2G}/GRP1). Magnified images from boxed regions, max projection of 8 (PH-PLCδ1) or 3 (CYTH3^{2G}/GRP1) z-slices, pseudocoloured in FIRE LUT. Arrowheads: red, cell–cell contact; yellow, nucleus; green, protrusion tip. Scale bar, 7 μm. Representative of 8 (*Trp53*$^{-/-}$) or 10 (*Trp53*$^{-/-}$;*Pten*$^{-/-}$) spheroids imaged across $n = 2$ independent experiments (PH-PLCδ1) and 22 (*Trp53*$^{-/-}$) or 23 (*Trp53*$^{-/-}$;*Pten*$^{-/-}$) spheroids imaged across $n = 4$ independent experiments (CYTH3^{2G}/GRP1).

B Intensity profiles for PH-PLCδ1 and PH-CYTH3 from spheroids shown in (A). Protrusions measured are annotated on images in upper panels, yellow lines. Arrowheads: red, protrusion tips.

C Schema, select PI-kinases and phosphatases and their inhibitors participating in PIP_3 production and downstream AKT phosphorylation.

D Western blotting and quantitation for S6RP pS235/236, S6RP, GAPDH (sample integrity control) in *Trp53*$^{-/-}$;*Pten*$^{-/-}$ 1.15 spheroids treated with DMSO or inhibitors annotated in (B) for 2 days. Representative of $n = 3$ independent lysate preparations. Data, mean ± SD of pS235/236:total S6RP ratio, normalised to DMSO. *P*-values, unpaired, two-tailed *t*-tests, as annotated.

E, F Quantitation of *Trp53*$^{-/-}$;*Pten*$^{-/-}$ 1.15 spheroids treated with DMSO, AKTi (AKT inhibitor II) or pan-PI3Ki (LY294002), 6-h time intervals over 72 h. (E) Heatmap (viridis)—area presented as mean of *Z*-score values, normalised to control (DMSO). (F) Frequency of Spherical and Hyper-protrusive phenotypes. Heatmap (grayscale)—phenotype proportion (*z*-score) in control. Heatmap (blue-red)—$\log_2$ fold change from control. *P*-values, bubble size (Cochran–Mantel–Haenszel test with Bonferroni adjustment). Black dot, homogenous effect across independent experiments (Breslow–Day test, Bonferroni adjustment, non-significant). $N = 2$ independent experiments, 4–5 technical replicates/experiment. Total spheroid number per condition, Table EV1.

G Representative phase contrast images of spheroids described in (E). Outlines pseudocoloured for classification (Spherical, green; Hyper-protrusive, blue). Magnified individual spheroids from boxed regions at indicated timepoints. Arrowheads, protrusions into ECM. Scale bar, 400 or 17 μm (indicated).

H, I Quantitation of ID8 *Trp53*$^{-/-}$;*Pten*$^{-/-}$ spheroids treated with PI3K isoform specific inhibitors: A66 (PI3Kα), AZD8186 (PI3Kβ), AS605240 (PI3Kγ) or CAL-101 (PI3Kδ), 6-h time intervals over 72 h. (H) Heatmap (viridis)—area presented as mean of *Z*-score values, normalised to control (DMSO). (I) Frequency of Spherical and Hyper-protrusive phenotypes. Heatmap (grayscale)—phenotype proportion (*z*-score) in control. Heatmap (blue-red)—$\log_2$ fold change from control. *P*-values, bubble size (Cochran–Mantel–Haenszel test with Bonferroni adjustment). Black dot, homogenous effect across independent experiments (Breslow–Day test, Bonferroni adjustment, non-significant). $N = 2$ independent experiments, 3–5 technical replicates/experiment. Total spheroid number per condition, Table EV1.

J Representative phase contrast images of spheroids described in (H, I). Outlines pseudocoloured for classification (Spherical, green; Hyper-protrusive, blue). Magnified individual spheroids from boxed regions at indicated timepoints. Arrowheads, protrusions into ECM. Scale bars, 400 or 17 μm, as indicated.

K Confocal image of *Trp53*$^{-/-}$;*Pten*$^{-/-}$ (1.15) spheroids stained for PI3Kβ (green), F-actin (magenta) and Hoechst (grey). Magnified images from boxed regions, pseudocoloured in inverted grayscale (F-actin) or FIRE LUT (PI3Kβ). Yellow or white/black arrowheads, enrichment of F-actin or PI3Kβ at protrusion tips respectively. Scale bar, 5 μm. Representative of $n = 5$ spheroids.

L Intensity profiles for PI3Kβ (green) and F-Actin (magenta) from spheroid in (K). Tip measured is annotated, ECM to body, yellow arrow, tip, arrowhead.

Source data are available online for this figure.

loss conditions, but do not display significant binding in the WT condition.

The majority of prominent ARF6 interactors, such as β1-integrin/*Itgb1* and α3-integrin/*Itga3*, or AGAP1, did not change upon *Trp53* or *Pten* loss (Fig 4J, colour scheme on left corresponds to grouping from 4I). When compared to WT ID8 cells, only a subset of ARF6 interactors were altered upon *Trp53* loss or when *Pten* was lost (Fig 4K and L), such as CYTH2 interaction increasing upon *Trp53* loss irrespective of *Pten* status, or α5-integrin/*Itga5* interaction specifically induced upon *Pten* loss. This suggests that rather than large-scale alteration to ARF6 networks, loss of *Pten* may change a small number of key network members or render cells dependent on constitutive ARF6 network members.

Cytohesin-2 function in invasion and contribution to ovarian cancer

The majority of known ARF GEFs were expressed in ID8 cells, and their expression was not consistently altered upon *Trp53* or *Pten* loss (Fig EV5A). However, only a single ARF GEF, Cytohesin-2 (CYTH2), was identified as interacting with ARF6 (Fig 4K). We therefore investigated chemical inhibition of Cytohesin-class GEFs using SecinH3 (Benabdi *et al*, 2017). SecinH3 treatment of *Trp53*$^{-/-}$;*Pten*$^{-/-}$ cells resulted in modestly smaller spheroids (Fig EV5B) that displayed less protrusive activity (Fig EV5C and D, arrowheads, Movie EV8). Accordingly, multiple leader cell formation was strongly reduced upon SecinH3 treatment (Fig EV5E, arrowheads) and consequently invasive activity and invasion distance (Fig EV5F and G). This suggests that CYTH2 may function with ARF6 to regulate invasion.

In ovarian cancer patients, *CYTH2* mRNA was increased in the tumour compared with normal epithelium in both independent data sets of LCM tumours, whereas stromal *CYTH2* levels were inconsistent across data sets (Fig EV5H and I). In bulk tumour sequencing, five of seven data sets indicate increased *CYTH2* mRNA levels in tumours (Fig EV5J–P) (Data refs: Wu *et al*, 2007b; Bonome *et al*, 2008b; Bowen *et al*, 2009b; King *et al*, 2011b; Elgaaen *et al*, 2012b; Lili *et al*, 2013b; Yeung *et al*, 2013b; Hill *et al*, 2014b; Yamamoto *et al*, 2016b). Comparison of *CYTH2* mRNA levels, based on median split comparing high (M2) versus low (M1), showed no significant difference in survival (Fig EV5Q). *CYTH2*, however, can be produced as two alternate transcripts based on alternate inclusion of exon 9.1, which encodes for a single additional glycine residue in the PH domain. Exclusion of exon 9.1 results in the CYTH2^{2G} isoform, which is preferential for PIP_3 binding, whereas inclusion of exon 9.1 results in the PI(4,5)P_2-binding CYTH2^{3G} isoform (Klarlund *et al*, 2000; Cronin *et al*, 2004; Oh & Santy, 2012). Therefore, the exon 9.1 Percentage Spliced In (Ex9.1 PSI) ratio can be used to distinguish such alternate PIP-associating CYTH2 isoforms. A modest but significant (3-month, $P = 0.0262$) decrease in overall survival was observed in patients displaying low Ex9.1 PSI (e.g. predominantly the PIP_3-associating CYTH2^{2G} isoform; Fig EV5R). Combination of *CYTH2* expression and splicing with *ARF6* expression levels revealed a significant (7–9 month) decrease in overall survival in *ARF6*^{HI}/*CYTH2*^{HI} patient subgroups (M2/M2) (Fig EV5S), which become more pronounced (13 months) only when *CYTH2* Ex9.1 PSI was low (i.e. when PIP_3-binding CYTH2^{2G} is predominant;

 © 2023 The Authors

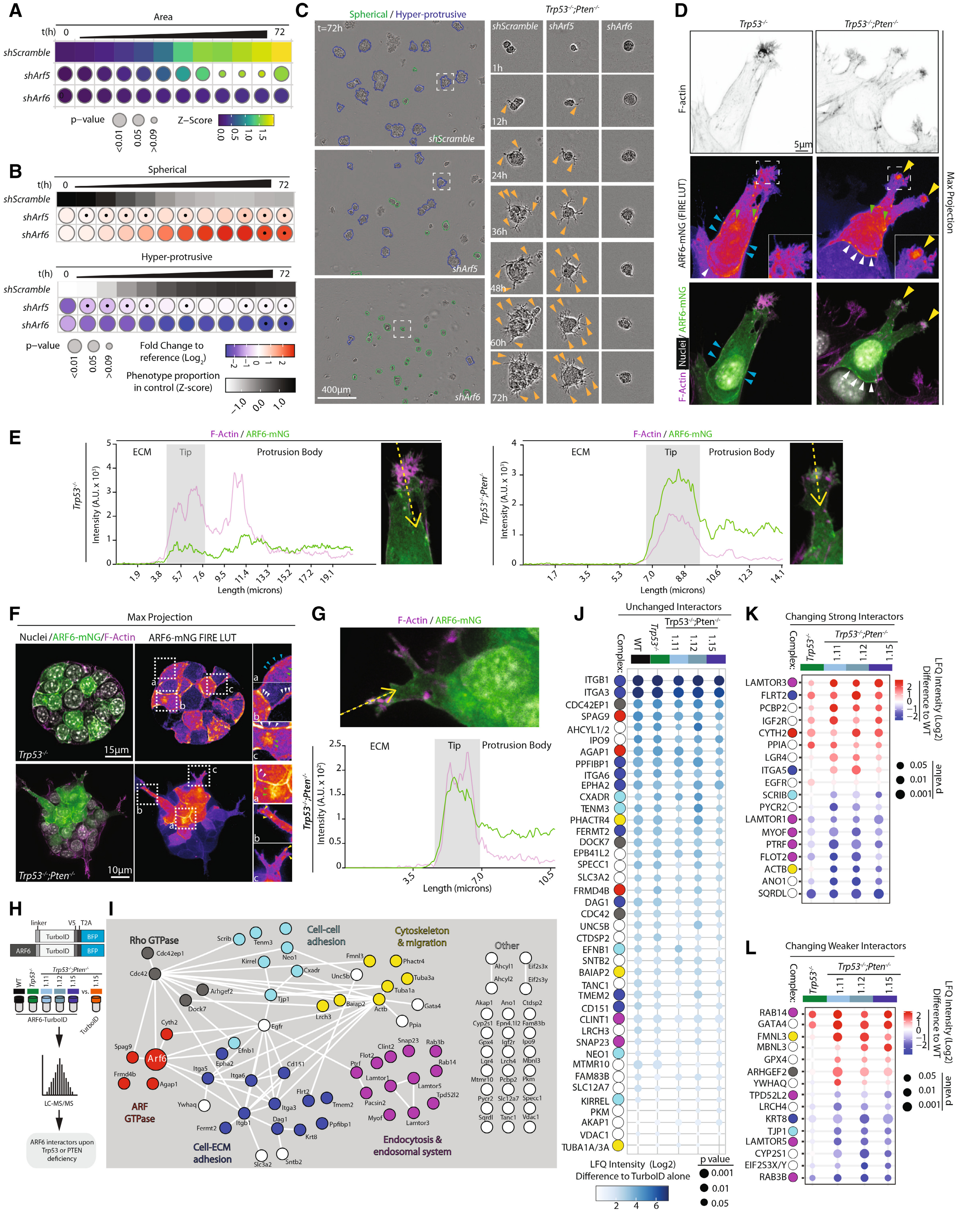

Figure 4.

Figure 4. The small GTPase ARF6 is required for *Pten*-loss mediated ECM invasion.

A, B Quantitation of ID8 *Trp53*$^{-/-}$;*Pten*$^{-/-}$ 1.15 spheroids expressing shScramble, sh*Arf5* or sh*Arf6*, 6 h time intervals over 72 h. (A) Heatmap (viridis)—area presented as mean of *Z*-score values, normalised to control (shScramble). (B) Frequency of Spherical and Hyper-protrusive phenotypes. Heatmap (grayscale)—phenotype proportion (*z*-score) in control. Heatmap (blue-red)—$\log_2$ fold change from control. *P*-values, bubble size (Cochran–Mantel–Haenszel test with Bonferroni adjustment). Black dot, homogenous effect across independent experiments (Breslow–Day test, Bonferroni adjustment, non-significant). *N* = 3 independent experiments, 4–5 technical replicates/experiment. Total spheroid number per condition, Table EV1.

C Representative phase contrast images of spheroids described in (A, B). Outlines pseudocoloured for classification (Spherical, green; Hyper-protrusive, blue). Magnified individual spheroids from boxed regions at indicated timepoints. Arrowheads, protrusions into ECM. Scale bars, 400 or 17 μm, as indicated.

D–G Representative confocal images and intensity profiles of ID8 *Trp53*$^{-/-}$ and *Trp53*$^{-/-}$;*Pten*$^{-/-}$ 1.15 cells expressing mNeonGreen (mNG)-tagged ARF6 (green) and stained with Hoechst (grey) and F-actin (magenta) at the invasive front of wounded monolayers (D, E) or in spheroids (max. projection ~ 10 Z-slices) (F, G). Pseudo colour is FIRE LUT. Magnified images of boxed regions are shown. Arrowheads: cell–cell contacts, white; cell-ECM contacts, blue; endosomes, grey; protrusion tips, yellow; intracellular pool, green. Scale bar, 5 μm. (E, G) Tips for which ARF6-mNG and F-Actin intensity profiles were measured are annotated, ECM to body, yellow arrow. (D) *n* = 2 independent experiments, 3–7 fields imaged/subline/experiment. (F) *n* = 3 independent experiments, 4–8 fields imaged/subline/experiment.

H Schema, mass spectrometry (MS) proteomic-based TurboID approach for detecting ARF6-proximal proteins.

I STRING network analysis of ARF6 interactions visualised using Cytoscape. Nodes manually annotated for known protein complexes. *N* = 4 independent lysate preparations from each subline.

J–L Heatmap, (J) unchanging, (K) Strong changing or (L) Weaker changing ARF6 interactors across genotypes. White to blue colour or blue to red, ARF6 interaction score, Log_2Fold Student's *t*-test Difference in LFQ intensity compared to control ID8 *Trp53*$^{-/-}$;*Pten*$^{-/-}$ 1.15 TurboID alone. Interactors, sorted, descending order of mean interaction. Circle size, *t*-test *P*-value, coloured spots underneath denote the protein complex that each interactor belongs (in I), manual annotation. *N* = 4 independent lysate preparations from each subline.

Source data are available online for this figure.

Fig EV5T). These data suggest that the PIP_3-binding *CYTH2* isoform is associated with poor survival when co-expressed with high levels of *ARF6*.

Identification of ARF6 interactors required for invasive activity

To identify additional ARF6 network proteins required for invasion, we performed a functional proteomic screen of 26 select interactors that represented constitutive ARF6 network members or those altered upon *Trp53* and *Pten* KO compared to WT (Fig 5A). In this approach, *Trp53*$^{-/-}$;*Pten*$^{-/-}$ ID8 cells were transduced with a lentiviral pool of 5× sgRNAs/gene and Cas9, for each of the 26 interactors. Each transduced and selected cell pool was then plated as 3D cultures, and Spherical and Hyper-protrusive phenotypes calculated from multiday time-lapse imaging. To ensure accuracy of plating in 3D culture, sgRNAs were broken into four iterations containing distinct gene targets (Screen Iteration 1–4) with a control (sgNon-targeting, sgNT) per iteration (Fig 5A). Each iteration contained multiple technical replicates and was performed three independent times. The effect of each pooled sgRNA was calculated as fold-change to control classification.

All pooled sgRNAs decreased Hyper-protrusiveness and increased Spherical phenotype to varying degrees, except for 14-3-3theta/*Ywhaq*, which showed a modest increase in Hyper-protrusive activity (Fig 5B). Notably, several constitutive ARF6 interactors (Fig 4J), such as ITGB1 and AGAP1, showed robust reduction in Hyper-protrusive activity when depleted (Fig 5B), while reduction in Hyper-protrusiveness could also be seen for sgRNAs against *Trp53* or *Pten* loss-induced interactors, such as *Cyth2* or *Itga5*, respectively (Figs 4K and L and 5B).

Deconvolution of sgRNAs to *Itgβ1*, *Agap1*, and *Itga5* revealed efficient CRISPR editing to each target across multiple independent sgRNAs (Fig 5C and D; Movies EV9 and EV10; Appendix Fig S1A–F). This occurred without consistent alterations to pS473-AKT levels in the *Itgβ1* and *Agap1*-depleted cell lines (Fig 5C and D, Appendix Fig S1C and D), suggesting that these effects are downstream of PI3K signalling. Each of *Itgb1*, *Agap1* (Fig 5E–G) and *Itga5* (Appendix Fig S1G and H) depletions resulted in spheroids that lacked Hyper-protrusive activity (arrowheads denote protrusions), confirming the pooled screen results (Fig 5B). This revealed that α5β1-integrin may be a major cargo of ARF6 that regulates interactions with the ECM to promote invasion, in conjunction with the GEF, CYTH2, and the GAP, AGAP1. This is particularly notable as although ITGB1 and AGAP1 association occurred across all genotypes (Fig 4J), ARF6 association with ITGA5 increased specifically in *Pten*-null conditions (Fig 4K). Notably, there was no change in the mRNA levels of either integrins in LCM HGSOC patient samples, while the comparison of either *ITGA5 or ITGB1* mRNA levels based on median split comparing high (M2) versus low (M1) showed no significant different in survival (Appendix Fig S1I–L; Data ref: Yeung *et al*, 2013b). Consistently, neither *Itga5 nor Itgb1* mRNA levels, or those of their ligand, Fibronectin (*Fn1*) changed across the ID8 sublines (Appendix Fig S1M). This suggests that the total expression of these integrins alone does not stratify patient survival.

To test whether altered interaction with the ECM underpins the *Pten*-null invasive phenotype, we examined basement membrane formation around spheroids by staining for Collagen IV (COL4), as the expression levels of *Col4* did not change upon loss of *Trp53* or *Pten* (Appendix Fig S1M). The pattern of Collagen IV surrounding ID8 spheroids could be classified as Fragmented, Defined or Absent (Fig 5H and I). In *Trp53*$^{-/-}$ spheroids, Collagen IV staining was well-defined (92.16% of spheroids, green arrowheads). By contrast, in *Trp53*$^{-/-}$;*Pten*$^{-/-}$ spheroids (expressing a nontargeting sgRNA), the majority of spheroids (73.1%) displayed a fragmented basement membrane, representing clear regions of presence (green arrowheads) and absence (yellow arrowheads) of Collagen IV. Continuous basement membrane formation could be restored in *Trp53*$^{-/-}$;*Pten*$^{-/-}$ spheroids by KO of *Agap1* (85.5%). Notably, basement membrane was largely absent upon *Itgb1* KO (80.1%). This suggests that disrupted basement membrane is associated with invasion and may contribute to hyperprotrusive activity upon *Pten* loss, but that invasion requires β1- integrin-dependent function in conjunction with the ARF6 interactor, AGAP1.

© 2023 The Authors

AGAP1 regulates collective invasion and is associated with poor survival

Although the majority of ARF GAPs are co-expressed in ID8 cells, and this expression is unaltered across the examined genotypes (Appendix Fig S2A), AGAP1 was the singular ARF GAP identified in the ARF6 interactome (Fig 4J). *Agap1* isoforms can differ by alternate inclusion of Exon 14, encoding part of the PH domain (Fig 6A), and resulting in AGAP1 Long (AGAP1-L) and AGAP1 Short (AGAP1-S) isoforms. The consequence of such splicing on AGAP1 is unknown.

Association of purified recombinant AGAP1-L and AGAP1-S PH domains identified that the major difference in lipid binding between isoforms is in phosphatidylserine (PS) association, while broad binding to phosphoinositides and phosphatidic acid (PA) was indistinguishable (Fig 6B; Appendix Fig S2B and C). We performed reconstitution of sgRNA-resistant mNG-tagged AGAP1 isoforms into AGAP1 KO *Trp53*$^{-/-}$;*Pten*$^{-/-}$ cells (Fig 6A and C), which were equally expressed (Fig 6C; Appendix Fig S2D). The AGAP1-S isoform modestly decreased ARF6 levels (~ 30% reduction) and AKT activation (~ 10% reduction) (Fig 6C and D). Accordingly, mNG-AGAP1-S-expressing spheroids were initially modestly smaller and strongly deficient in protrusive activity, but this was restored to control levels by later time points (Fig 6E–G). In contrast, mNG-AGAP1-L-expressing spheroids showed increased size and while initially less Hyper-protrusive than control (sgNT) spheroids, AGAP1-L spheroids became more protrusive than control cells in the second half of the imaging period (Fig 6E–G, arrowheads). Staining for endogenous AGAP1 in Hyperprotrusive *Trp53*$^{-/-}$;*Pten*$^{-/-}$ spheroids showed, in addition to a generalised cytoplasmic localisation, a prominent pool at the tip of invasive protrusions (Appendix Fig S2E and F). This suggests that both AGAP1 isoforms can support protrusive activity to varying degrees, though this occurs robustly for the weakly PS-associating AGAP1-L isoform.

In five of seven bulk tumour data sets, *AGAP1* mRNA expression was elevated in tumour compared with normal ovarian tissue, which occurred in the epithelium in independent LCM tumour data sets, but not consistently in the stroma. In contrast, *ARF6* showed a less consistent alteration across data sets, with *ARF6* mRNA elevated in only three of seven bulk tumour data sets, and *ARF6* mRNA elevation occurring in the stroma in LCM data sets (Fig 6H–J; Appendix Fig S2G–L) (Data refs: Wu *et al*, 2007b, Bonome *et al*, 2008b, Bowen *et al*, 2009b, King *et al*, 2011b, Elgaaen *et al*, 2012b, Lili *et al*, 2013b, Yeung *et al*, 2013b, Hill *et al*, 2014b, Yamamoto *et al*, 2016b). While comparison of *ARF6* mRNA (Fig 6K) or *AGAP1* Exon 14 PSI (Fig 6L, High vs. Low levels based on median split) did not affect overall survival, *AGAP1* mRNA levels strongly segregated survival groups, whether based on median split (Fig 6M) or comparing Quartile 1 to Quartile 4 (Appendix Fig S2M). In both cases, a difference of 10-month survival was observed. Combining *ARF6* and *AGAP1* mRNA levels, but not *AGAP1* Exon 14 PSI, even further separated overall survival, with a robust 17-month increase in overall survival of *ARF6*^{LO}-*AGAP1*^{LO} patients (red line), compared with the poor survival of *ARF6*^{HI}-*AGAP1*^{HI} patients (blue line) (Fig 6N). This same effect could not be found when examining splicing of *AGAP1* at Exon 14 (Fig 6O). Together, these data indicate that AGAP1 is required for invasion in *Pten*-null cells and that ovarian cancer patients with high *ARF6* and *AGAP1* levels, irrespective of the isoform of the *AGAP1*, have a poor clinical outlook.

ARF6 regulates active integrin pools to produce invasive protrusions

Our data thus far indicate that a CYTH2-ARF6-AGAP1 module is required for invasion in *Pten*-null cells and that α5-integrin and β1-integrin are two ARF6-promixal proteins essential for this phenotype. Although the mRNA levels of these two integrins are not altered in normal versus tumour epithelium, nor do they stratify patient survival based on a medium split (Appendix Fig S1K and L), we explored whether the ARF6 module may act by regulating distribution of ECM-adhesion complexes to the tips of protrusions to drive invasion. In addition to cortical localisation, both α5-integrin and β1-integrin showed localisation to the extreme tips of invasive protrusions in *Trp53*$^{-/-}$;*Pten*$^{-/-}$ spheroids, occurring prior to F-actin enrichment and mirroring the localisation observed for both pAKT (S473) and PIP_3 (Fig 7A and B). Two markers of ECM-signalling hubs, pY397-FAK and pY416-Src family kinases (SFK), also localised prominently to the tips of protrusions (Fig 7C and D), in addition to the cell-ECM interface. This observation suggests a pool of integrin signalling complexes localise to protrusion tips.

In ovarian cancer patients with low levels of PTEN protein, pY416-SFK protein levels were elevated (Fig 1I). To interrogate whether loss of PTEN may be associated with alterations in membrane trafficking of these integrins, we used a captured-based ELISA approach (Roberts *et al*, 2001). In *Trp53*$^{-/-}$;*Pten*$^{-/-}$ cells, the recycling of internalised total α5- or β1-integrin, the active form of β1-integrin, or a control cargo of Transferrin Receptor (TfnR), was increased at all time points examined compared with *Trp53*$^{-/-}$ cells. This increase reached statistical significance ($P < 0.05$) at $t = 32$ min for active β1 integrin (Fig 7E and F; Appendix Fig S3A–F).

To determine whether ARF6 regulates this enhanced recycling in *Trp53*$^{-/-}$;*Pten*$^{-/-}$ cells, we depleted *Arf6*. *Arf6* depletion in *Trp53*$^{-/-}$;*Pten*$^{-/-}$ cells specifically blunted recycling of active form of β1-integrin, but not of total α5-integrin, β1-integrin or TfnR (Fig 7G and H; Appendix Fig S3G–K). This suggests that the CYTH2-ARF6-AGAP1 module specifically regulates recycling of the active β1-integrin, while trafficking of inactive β1-integrins and TfnR is controlled by other signalling modules downstream of PIP_3.

Combined analysis of CYTH2-ARF6-AGAP1 module mRNA levels in ovarian cancer patients indicated that high levels of all three components (blue line; upper grouping based on median split of each gene's expression, M2) showed a significant, 17-month decrease in survival compared to low levels (red line, M1; $P = 3.264e-3$; Fig 7I). This effect could be recapitulated only when considering the PIP_3-binding *CYTH2*^{2G} isoform (i.e. low levels of Ex9.1PSI, blue line; $P = 0.0159$, Fig 7J). More modest effects could be observed in pairwise comparisons of *CYTH2-ARF6-AGAP1*, and splicing variants of *AGAP1* and *CYTH2* (Appendix Fig S3L–N). Crucially, the poor outcome associated with patients with low PTEN protein levels (Fig 1G) requires simultaneous high mRNA levels of each of the three ARF module components (CYTH2-ARF6-AGAP1) as patients with low PTEN protein levels and low mRNA levels of each of CYTH2-ARF6-AGAP1 had indistinguishable outcome to high PTEN protein patients (Fig 7K). This resulted in a 23-month difference in

© 2023 The Authors

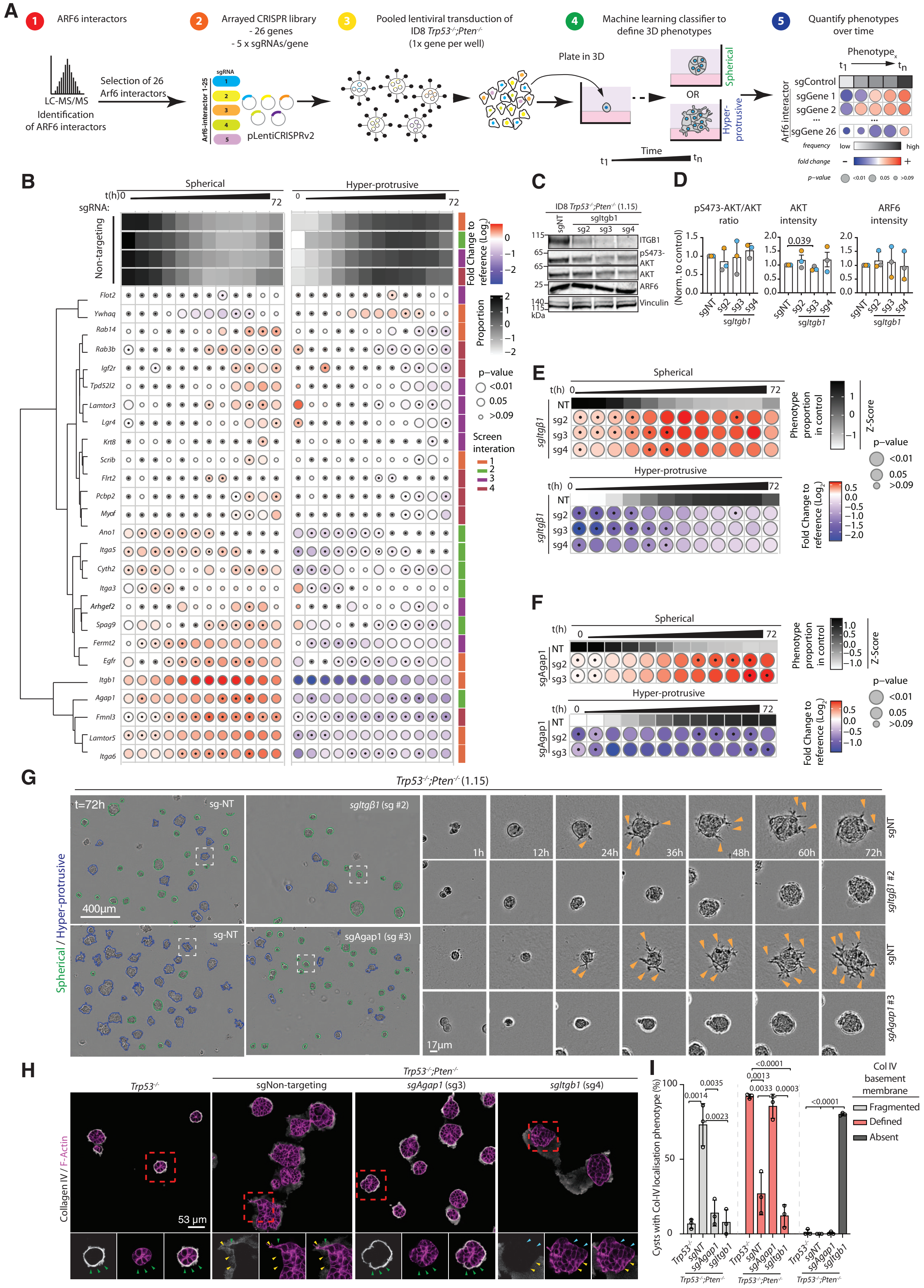
A
1 ARF6 interactors
2 Arrayed CRISPR library - 26 genes - 5 x sgRNAs/gene
3 Pooled lentiviral transduction of ID8 Trp53-/-;Pten-/- (1x gene per well)
4 Machine learning classifier to define 3D phenotypes
5 Quantify phenotypes over time
LC-MS/MS Identification of ARF6 interactors
Selection of 26 Arf6 interactors
pLentiCRISPRv2
Plate in 3D
OR
Spherical
Hyper-protrusive
Time
sgControl
sgGene 1
sgGene 2
sgGene 26
Arf6 interactor
B
Spherical
Hyper-protrusive
sgRNA:
Non-targeting
Fold Change to reference (Log2)
Proportion
p-value
Screen interaction
Flot2
Ywhaq
Rab14
Rab3b
Igf2r
Tpd52l2
Lamtor3
Lgr4
Krt8
Scrib
Flrt2
Pcbp2
Myof
Ano1
Itga5
Cyth2
Itga3
Arhgef2
Spag9
Fermt2
Egfr
Itgb1
Agap1
Fmnl3
Lamtor5
Itga6
C
ID8 Trp53-/-;Pten-/- (1.15)
sgNT
sgItgb1
ITGB1
pS473-AKT
AKT
ARF6
Vinculin
kDa
D
pS473-AKT/AKT ratio
AKT intensity
ARF6 intensity
(Norm. to control)
0.039
E
Spherical
Hyper-protrusive
sgItgβ1
Phenotype proportion in control
Z-Score
p-value
Fold Change to reference (Log2)
F
Spherical
Hyper-protrusive
sgAgap1
G
Trp53-/-;Pten-/- (1.15)
t=72h
sg-NT
sgItgβ1 (sg #2)
sgAgap1 (sg #3)
400µm
17µm
Spherical / Hyper-protrusive
1h 12h 24h 36h 48h 60h 72h
sgNT
sgItgβ1 #2
sgAgap1 #3
H
Trp53-/-
Trp53-/-;Pten-/-
sgNon-targeting
sgAgap1 (sg3)
sgItgb1 (sg4)
Collagen IV / F-Actin
53 µm
I
Cysts with Col-IV localisation phenotype (%)
Col IV basement membrane
Fragmented
Defined
Absent
0.0014
0.0035
0.0023
<0.0001
0.0013
0.0033
0.003
<0.0001

Figure 5.

Figure 5. A functional proteomic CRISPR screen for ARF6-proximal proteins controlling collective invasion.

A Schema, (1) CRISPR screen. 26 ARF6-proximal proteins from TurboID studies were investigated for their contribution to ARF6-mediated invasion of ID8 Trp53$^{-/-}$; *Pten*$^{-/-}$ spheroids. (2) For each interactor, 5 sgRNAs were cloned into lentiviral CRISPR vectors. (3) A pooled approach was used, generating a KO cell line with all 5 sgRNAs (4) Live imaging performed. (5) Phenotype of each KO compared with nontargeting sgRNA.

B Frequency of Spherical and Hyper-protrusive phenotypes upon pooled gRNA CRISPR of indicated targets (sorted based on hierarchical clustering) in ID8 *Trp53*$^{-/-}$; *Pten*$^{-/-}$ clone 1.15 cells, performed in four parts (Iterations indicated). Heatmap (grayscale)—phenotype proportion (*z*-score) in control (sgNT). Heatmap (blue-red)—$\log_2$ fold change from control. *P*-values, bubble size (Cochran–Mantel–Haenszel test with Bonferroni adjustment). Black dot, homogenous effect across independent experiments (Breslow–Day test, Bonferroni adjustment, nonsignificant). $N = 3$–4 independent experiments, 3–6 technical replicates/experiment. Total spheroid number per condition, Table EV1.

C Western blot, β1-integrin (ITGB1), pS473-AKT, AKT, ARF6 from deconvolved ITGB1 sgRNA-expressing cells. VCL, loading control for ITGB1, sample integrity control for other blots. Representative blots of $n = 3$ independent lysate preparations.

D Quantitation of (C). Data, mean ± SD for pS473-AKT:total AKT band intensity ratio, total AKT or ARF6 intensity, normalised to control (sgNT ID8 *Trp53*$^{-/-}$;*Pten*$^{-/-}$ clone 1.15) cells. *P*-values, unpaired, two-tailed *t*-test.

E, F Frequency of Spherical and Hyper-protrusive phenotypes in ID8 *Trp53*$^{-/-}$;*Pten*$^{-/-}$ 1.15spheroids upon CRISPR-mediated KO of (E) *Itgβ1* or (F) *Agap1*, 6 h time intervals over 72 h. Heatmap (grayscale)—phenotype proportion (*z*-score) in control (sgNT). Heatmap (blue-red)—$\log_2$ fold change from control. *P*-values, bubble size (Cochran–Mantel–Haenszel test with Bonferroni adjustment). Black dot, homogenous effect across independent experiments (Breslow–Day test, Bonferroni adjustment, non-significant). $N = 3$ independent experiments, 1–5 technical replicates/experiment. Total spheroid number per condition, Table EV1.

G Representative phase contrast images of spheroids described in (E, F). Outlines pseudocoloured for classification (Spherical, green; Hyper-protrusive, blue) at indicated timepoints. Magnified individual spheroids from boxed regions. Arrowheads, protrusions into ECM. Scale bars, 400 or 17 μm, as indicated.

H Representative confocal images of *Trp53*$^{-/-}$ and *Trp53*$^{-/-}$;*Pten*$^{-/-}$ *clone* 1.15 spheroids expressing sgNT, sg*Agap1* (sg3) or sg*Itgb1* (sg4), stained for collagen IV (grayscale) and F-Actin (magenta). Boxed areas, basement membrane region in higher magnification. Arrowheads, Collagen IV labelling that is: well-defined, green; fragmented, yellow; absent, navy. Scale bar, 53 μm.

I Quantitation of (H). Collagen IV basement membrane staining as Defined, Fragmented, or Absent in spheroids set up across $n = 3$ independent experiments, 1 technical replicate/experiment, 5–9 fields imaged per technical replicate, 365 spheroids scored in total. Data, mean ± SD of % of spheroids in each phenotype for independent experiments, with circles representing technical replicates. Unpaired *t*-test, *P*-values annotated.

Source data are available online for this figure.

survival in patients with low PTEN protein and high ARF module (blue line) compared with high PTEN levels (green line; Fig 7K). Accordingly, a robust protein phosphorylation signature for PI3K-AKT signalling was observed in Ovarian Cancer patients with high levels of *ARF6-AGAP1-CYTH2* (specifically the PIP_3-binding *CYTH2*^{2G}, but not the PI(4,5)P_2-binding *CYTH2*^{3G} variant; Fig 7L). Collectively, this indicates a role for the potentially PIP_3-responsive CYTH2-ARF6-AGAP1 module in regulating survival in ovarian cancer through controlling recycling of a5β1-integrin complexes to invasive protrusion tips.

Discussion

Here, we propose a model of how loss of *Pten* can drive invasive behaviours, central to which is PTEN's function as a phosphatase controlling PIP_3 levels and localisation (Fig 7M). In *Pten*-expressing cells, PIP_3 localises to cell–cell contacts. In *Pten* KO cells, while cell–cell contact PIP_3 is not lost, a prominent pool of PIP_3 appears in ECM-invading protrusion tips. The small GTPase ARF6 likely acts directly in PIP_3-elevated areas through activation by the PIP_3-associating variant of its cognate GEF, CYTH2^{2G}, and via its GAP AGAP1. This ARF6 module functions in the recycling of internalised active pools of integrin, thereby driving invasive protrusions enriched for the adhesion signal-transducing FAK and SFKs. This suggests a model wherein PTEN loss elevates recycling of the invasion-promoting cargoes α5β1-integrins. The cellular consequence of this is altered interaction with the ECM.

It is notable that this CYTH2-ARF6-AGAP1 module was not specifically and only induced in *Pten*-null contexts, but rather that *Pten*-null cells became dependent on the module for enacting the invasive phenotype. Indeed, with the exception of α5-integrin, the majority of ARF6-proximal protein network was largely unchanged across *Trp53* or *Pten* knockout cells. This suggests that ARF6 and interactors likely have a steady-state function in recycling active integrins. It may be that this function is enhanced in *Pten* KO cells, as in our functional proteomic CRISPR screen of ARF6-proximal proteins we identified KINDLIN-2/FERMT2, a PIP_3-binding regulator of integrin activation. When PIP_3 levels are high, it is possible that, in addition to selectively supporting recycling of previously internalised active integrin cargoes, ARF6 may collaborate with KINDLIN-2 to promote or maintain activation of recycled integrins at the plasma membrane, although this remains to be tested. In addition, a number of additional hits in the screen, such as EGFR, FMNL3, LAMTOR5 and ITGA6, gave strong reductions in Hyper-protrusiveness and may act as additional ARF6 cargoes or effectors in regulating collective invasion.

It should be highlighted that our observations herein and the model we are suggesting do not imply that ARF6 is required for invasion initiation, but rather for invasion maturation and persistence. Indeed, upon ARF6 depletion, PTEN-null spheroids often exhibited the formation of fine, transient protrusions. In most cases, however, these were not enough to lead to the formation of a stable invasion structure. Similarly, ECM-embedded *Trp53*$^{-/-}$;*Pten*$^{-/-}$ ARF6 KD monolayers were still able to invade and eventually close the monolayer wound albeit with reduced efficiency compared with their ARF6-proficient counterparts. This mirrored the behaviour of both WT and *Trp53*$^{-/-}$ spheroids and monolayers. We reported a similar function of ARF6 regulating protrusion maturation rather than initiation in conjunction with the ARF GEF protein IQSEC1 in invading 3D cultures of prostate cancer cells (PC3) (Nacke *et al*, 2021).

It is notable that the effects of *Pten* or *Trp53* loss were most prominent in 3D culture. This suggests that the phenotypes of loss of these central tumour suppressors may only manifest when cells are embedded in extracellular matrices and/or when multicellularity is considered. Indeed, the basement membrane around *Trp53*$^{-/-}$;

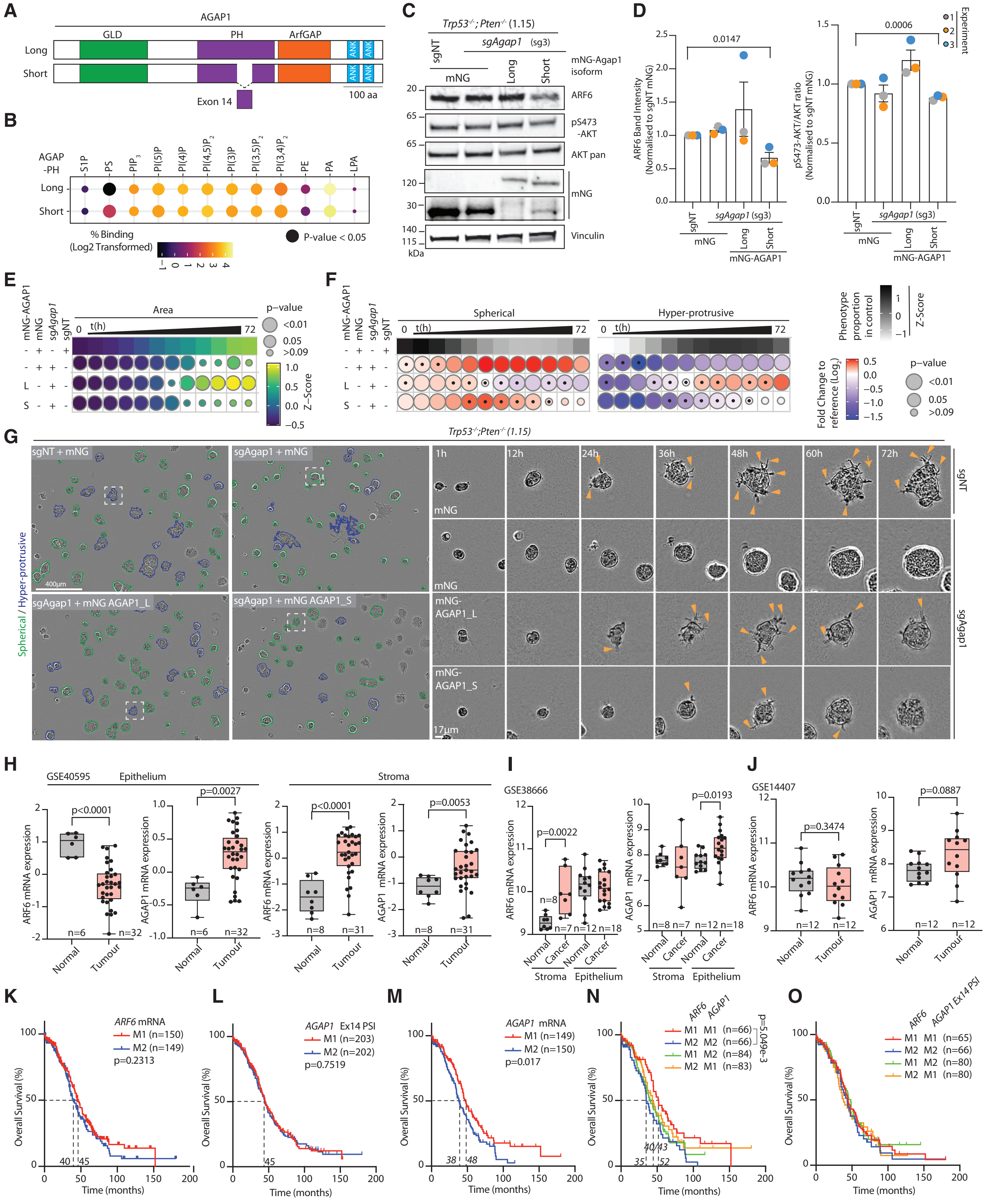

Figure 6.

 © 2023 The Authors

Figure 6. The ARFGAP AGAP1 controls invasion and stratifies survival.

A Schema, AGAP1 isoform domains. GLD, GTP binding-like domain; PH, Pleckstrin homology; ANK, Ankyrin; ARF GAP, ARF GTPase-activating Protein. Based on information found in www.ensembl.org (Cunningham *et al*, 2022; 'Long' isoform, Transcript ID: ENST00000304032.13 for the human genome, and ENSMUST00000027521.15 for the mouse genome) or 804 amino acids ('Short' isoform, Transcript ID: ENST00000336665.9 for the human and ENSMUST00000190096.7 for the mouse genome) and previously described annotations of AGAP1 domains (Nie *et al*, 2002).

B Heatmap, differential association of isoforms with phospholipids. Data, Log_2-transformed % of total signal between AGAP1-S versus AGAP1-L GST-tagged PH domain association with each phospholipid. *P*-value, circles size (unpaired *t*-test). $n = 3$ blots per condition.

C Western blots of ID8 Trp53$^{-/-}$;*Pten*$^{-/-}$ 1.15 cells expressing either sgNT or sgAgap1 (sg3) and either mNeonGreen (mNG) or CRISPR-resistant mNG-Agap1_S or -L isoforms. Blotted for ARF6, pS473-AKT, AKT, mNG, and VCL. VCL is loading control for AKT, pS473-AKT and ARF6 and sample integrity control for others. $n = 3$ independent lysate preparations.

D Quantitation of (C). Data, mean ± SD for ARF6 and pS473/AKT band intensity ratio, normalised to sgNT. *P*-values, unpaired two-tailed *t*-test, annotated when significant.

E, F Quantitation of ID8 *Trp53*$^{-/-}$;*Pten*$^{-/-}$ 1.15 spheroids treated with sgNT or AGAP1-targeting sg3 and expressing either mNG or mNG-fusion with either isoform of AGAP1, 6 h time intervals over 72 h. (E) Heatmap (viridis)—area presented as mean of *Z*-score values, normalised to control (sgNT). (F) Frequency of Spherical and Hyper-protrusive phenotypes. Heatmap (grayscale)—phenotype proportion (*z*-score) in control. Heatmap (blue-red)—log_2 fold change from control. *P*-values, bubble size (Cochran–Mantel–Haenszel test with Bonferroni adjustment). Black dot, homogenous effect across independent experiments (Breslow-Day test, Bonferroni adjustment, nonsignificant). $N = 3$ independent experiments, 5–6 technical replicates/experiment. Total spheroid number per condition, Table EV1.

G Representative phase contrast images of spheroids described in (E, F). Outlines pseudocoloured for classification (Spherical, green; Hyper-protrusive, blue). Magnified individual spheroids from boxed regions at indicated timepoints. Arrowheads, protrusions into ECM. Scale bars, 400 or 17 μm, as indicated.

H–J *ARF6 and AGAP1* mRNA levels in LCM normal ovarian surface epithelium versus HGSOC epithelium or normal ovarian stroma versus OC-associated stroma. Specific data sets, sample size (*n*) and *P*-values (Mann–Whitney) annotated, whiskers Min–Max, line at median.

K–O Overall survival (% patients, months; TCGA OV data set), of patients grouped by low (M1) versus high (M2) levels, based on a median split, of (K) *ARF6* mRNA, (L) *AGAP1* mRNA, (M) *AGAP1* Exon 14 percentage spliced in ratio (PSI), (N) combination of *ARF6* and *AGAP1* mRNA, or (O) combination of *ARF6* mRNA and *AGAP1* Ex14 PSI. Median survival, sample size (*n*) and *P*-value, Log-rank test (Mantel-Cox) annotated.

Source data are available online for this figure.

Pten$^{-/-}$ spheroids was fragmented. This may explain how *Pten* loss resulted in the hyperactivation of leader-cell function in most cells at the ECM interface, rather than co-ordination of follower cells behind a singular leader cell. The tumour suppressor function of PTEN therefore may normally function to co-ordinate polarisation and cellular position in multicellularity by controlling basement membrane assembly through integrins, structurally influencing where invasive protrusions can occur.

The application of machine-learning approaches to live imaging allowed us to classify hundreds-to-thousands of spheroids tracked over time into robustly statistically supported categories, Spherical and Hyper-protrusive. While these labels were pivotal in identifying molecular perturbation that essentially turn on or off invasive behaviours, they are broad categories. It may be that subtle and important differences occur between perturbations, which could be further segregated with additional phenotype classifications. Indeed, while Hyper-protrusive *Trp53*$^{-/-}$;*Pten*$^{-/-}$ spheroids have fragmented basement membranes, this could be reversed to a fully defined basement membrane upon *Agap1* KO, thereby preventing protrusions. *Itgβ1* KO spheroids, however, largely lacked an assembled basement membrane but also the ability to interact with the ECM to form protrusions. Both *Agap1* and *Itgβ1* KO in *Trp53*$^{-/-}$;*Pten*$^{-/-}$ spheroids lack invasive protrusions, suggesting different alterations can result in similar morphogenetic consequences.

For consistency and clarity, we excluded from our analyses objects that were either out of focus during imaging or had completely invaded to the bottom of the dish. This ensured that objects that could not be imaged properly would not be improperly segmented and thus erroneously classified as either Spherical or Hyper-protrusive. Furthermore, the highly invasive cells that sometimes invaded from early time points were almost exclusively found in the *Trp53*$^{-/-}$;*Pten*$^{-/-}$ spheroids (across all clones). While we cannot exclude the possibility that this exclusion may lead to some underestimation on the magnitude of the effect of PTEN loss, we felt that since the effect was clear even upon exclusion, this was a more honest way of performing our analyses as it allowed for more accurate classification of 3D structures. More refined subcategorisations may help to detect additional phenotypic variations.

Pten knockout alone was sufficient to drive *in vitro* invasion in the absence of *Trp53* depletion. The exact contribution of *Trp53* loss to the invasive phenotypes we examined is unclear. We observed modest alterations to phenotypes upon *Trp53* KO alone, such as increased protrusive tip formation or invasive capacity, but not sufficiently outside the range of normal variation to reach significance. Dissecting the role of *Trp53* is likely more complicated than we have examined as although *TP53* alteration is near-universal in ovarian cancer, many of these are distinct mutation, including some hotspots. Intraperitoneal injection of *Trp53*-null ID8 cells increases tumour growth rate and decreases survival compared with parental cells (Walton *et al*, 2016), showing that *Trp53* loss alone does cause *in vivo* functional differences to tumorigenesis. This tumorigenesis effect is accelerated *in vivo* by *Pten* co-knockout (Walton *et al*, 2017). Whether *Trp53* mutation versus loss differently contributes to *Pten*-depleted phenotypes remains to be examined.

The intraperitoneal injection of ID8 cells is an excellent system for *in vivo* examination of tumorigenesis in an immune-competent host. However, the rapid progression to clinical endpoint due to excess ascites production and spread of cells within the peritoneal fluid, rather than *bona fide* invasion, renders it poorly suited to determine contributions to metastasis, particularly in the case of *Pten*-null tumours due to rapid progression (*Trp53*$^{-/-}$;*Pten*$^{-/-}$, 34 days; *Trp53*$^{-/-}$, 47 days; Parental ID8, ~ 100 days) (Walton *et al*, 2016, 2017). Validation of the *in vivo* contribution of PTEN loss to metastasis requires the use of approaches that allow metastasis to occur before clinical endpoint is reached. Introduction of such additional models is beyond the mechanistic cell biological studies provided here.

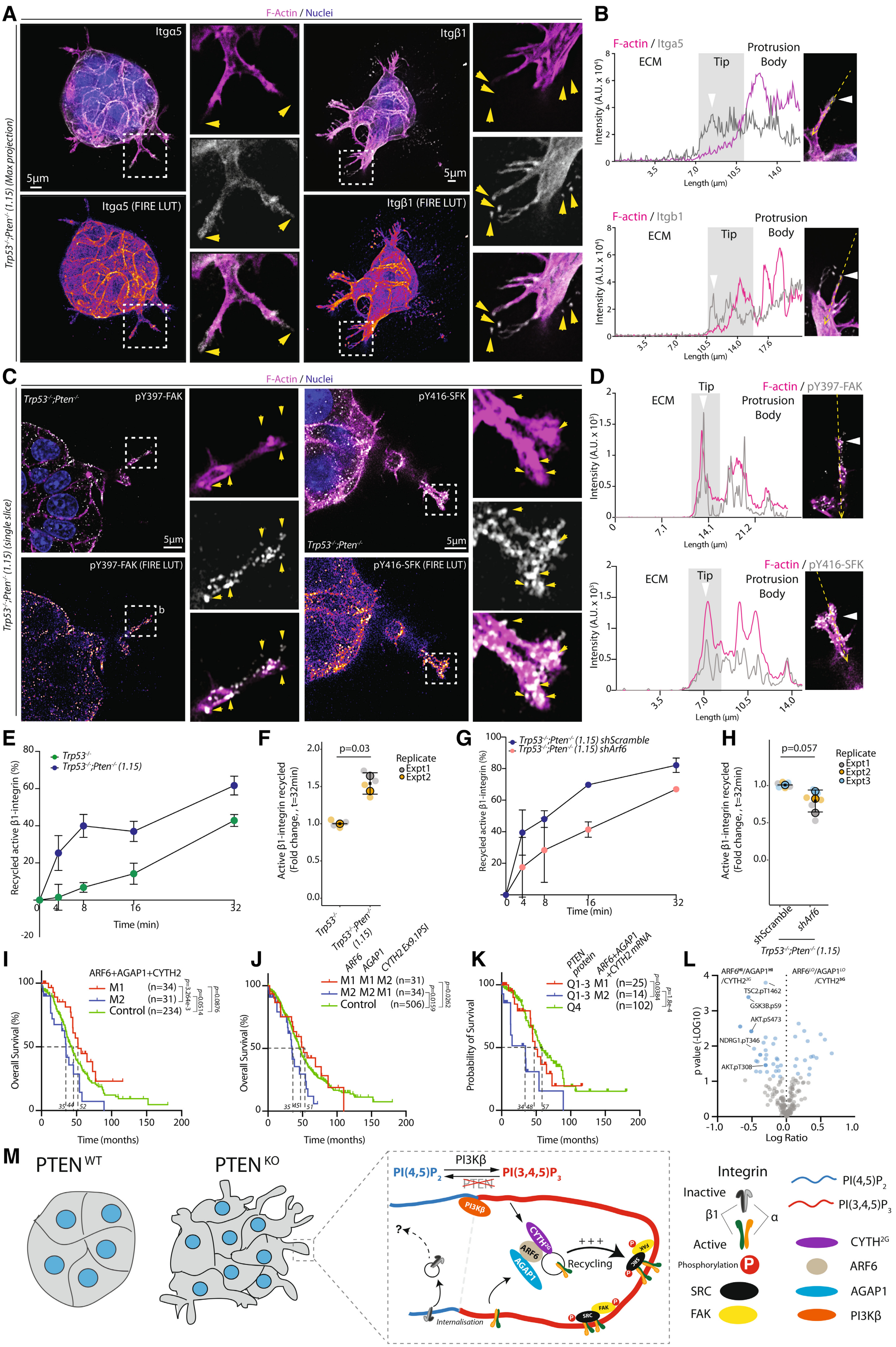

Figure 7.

 © 2023 The Authors

◀ **Figure 7. ARF6 controls invasion by regulating recycling of active integrins.**

A, B Immunofluorescence and confocal imaging of *Trp53*$^{-/-}$;*Pten*$^{-/-}$ 1.15 spheroids stained for α5-integrin or β1-integrin (grey or FIRE LUT), Hoechst (blue) and F-actin (magenta). Magnified images from boxed regions shown. Arrowheads, labelling at protrusion tips. Scale bars, 5 μm. Representative of n = 3 spheroids imaged. (B) Intensity profiles for integrins (grey) and F-actin (magenta) from spheroids in (A). Tip measured is annotated, ECM to body, yellow arrow, tip, white arrowhead.

C, D Immunofluorescence and confocal imaging of *Trp53*$^{-/-}$;*Pten*$^{-/-}$ 1.15 spheroids stained for pFAK (Y379) or pSRC Family Kinases (SFK pY416) (grey or FIRE LUT), Hoechst (blue) and F-actin (magenta). Magnified images from boxed regions shown. Arrowheads, positive staining. Scale bars, 5 μm. Representative of n = 5 spheroids imaged. (D) Intensity profiles for active FAK and Src (grey) and F-actin (magenta) from spheroids in (C). Tip measured is annotated, ECM to body, yellow arrow, tip, white arrowhead.

E–H Representative capture ELISA graphs (E, G) and associated quantitation (F, H) for recycling of internalised cargoes between *Trp53*$^{-/-}$ versus *Trp53*$^{-/-}$;*Pten*$^{-/-}$ cells or *Trp53*$^{-/-}$;*Pten*$^{-/-}$ cells expressing sh*Scramble* versus sh*Arf6* for active β1-integrin. Graphs shown are representative of n = 2 (E) or n = 3 (G) independent replicates. Data, mean (black square) ± SD for repeated experiments (large circles), 1–3 technical replicates/experiment/timepoint (small circles), two-tailed t-test, P-values are annotated.

I–K Overall survival (% patients, months; TCGA OV data set) of patients grouped into combined expression based on median mRNA split. (I) Low (red line, M1) or high (blue line, M2) expression for all mRNA, control, remaining patients (green line), (J), same as (I), but *CYTH2* Ex9 PSI, rather than total CYTH2. (K), as for (I), but PTEN protein levels split by quantiles (red and blue, Q1 + Q2, Q3, low PTEN, green Q4, high PTEN). Median survival, sample size (n) and P-value, Log-rank test (Mantel-Cox) annotated.

L Differential abundance (x, Log Ratio between conditions; y, Log_{10} q-values) of proteins in PIP_3-responsive module (ARF6^{HI}-AGAP1^{HI}-CYTH2^{2G}) versus PI(4,5)P_2-responsive ARF module (ARF6^{HI}-AGAP1^{HI}-CYTH2^{3G}) protein samples. Reverse Phase Protein Array Data, TCGA OV. Significantly altered components in AKT signalling pathway labelled ($-\mathrm{Log}_{10}$ q-value > 1.3).

M Schema, molecular model for ARF GTPase regulation of integrin-dependent invasion.

Source data are available online for this figure.

In ovarian cancer patient cohorts, *PTEN* loss is frequent and PTEN protein loss is associated with poor outcome. *ARF6* mRNA levels themselves were not consistently altered across independent data sets, making *ARF6* mRNA alone a likely unsuitable potential biomarker of poor outcome. Both the *CYTH2* GEF and *AGAP1* GAP mRNAs were elevated in tumour tissue in a number of data sets; however, CYTH2 contribution is complicated by poor outcome being specifically conferred by the PIP_3-associating CYTH2^{2G} isoform. While the effect of CYTH2^{2G} (PIP_3-binding variant) on survival is modest; (3-month decrease when CYTH2^{2G} is high), combining this with high ARF6 levels allows identification of a 10-month decrease in survival. Due to this isoform lacking a single amino acid difference to the PI(4,5)P_2-binding CYTH2^{3G} isoform, this complexity renders CYTH2 analysis alone a poor biomarker candidate. *AGAP1* mRNA levels, in contrast, strongly stratified patient outcome. Combined high versus low mRNA levels of *CYTH2-ARF6-AGAP1* provided the most robust 17-month different in survival of ovarian cancer patients, which occurred in patients with an PI3K-AKT signature. This emphasises the capacity of *in vitro* 3D cell biology to identify mechanistic insight into tumour suppressor contribution to cancer that can be used to clinically stratify poor and superior patient survival groups.

Materials and Methods

Cell culture

All ID8 sublines were maintained in Dulbecco's modified Eagle medium (DMEM) supplemented with 4% heat-inactivated Fetal Bovine Serum (FBS), 2 mM L-Glutamine, 1× Insulin-Transferrin-Selenium (0.01 mg/ml Insulin, 5.5 μg/ml Transferrin, 6.7 ng/ml Selenium) and 10 U Penicillin–Streptomycin (all Gibco). HEK293-FT cells were maintained in DMEM with 10% FBS, 2 mM L-Glutamine and 0.1 mM Non-Essential Amino Acids (NEAA) (all Gibco). Cells were incubated at 37°C, 5% CO_2 and routinely tested for mycoplasma contamination. Inhibitors were added at the following concentrations: 10 μM Nutlin3A (Merck, SML0580), 10 μM pan-PI3Ki (LY294002, Merck, 440204), 200 nM PI3Kα-i (A66, Selleckchem, S2636), 200 nM PI3Kβ-i (AZD8186, AstraZeneca), 200 nM PI3Kγ-i (AS605240, Stratech, S1410), 200 nM PI3Kδ-i (Cal-101, Stratech, S2226), 20 μM SecinH3 (Tocris, 2849).

Generation of stable cell lines

Lentiviral delivery systems were used for the generation of stable knock down (KD) lines (pLKO.1 puro), CRISPR knock out (KO) (pLentiCRISPR v2 Neo, Addgene #98292) or for overexpression of mNG protein fusions and the TurboID construct (pLX303/304 blast). A list of all sgRNAs is provided in Table EV2, a list of shRNAs used is provided in Table EV3. A list of all constructs used is shown in Table EV4, which will be made available on Addgene. HEK293-FT cells at 70% confluence were co-transfected with the plasmid of interest (0.50 μg DNA/reaction) and lentiviral packaging vectors (pMD2.G, Addgene, #12259, 0.05 μg/reaction; and psPAX2, Addgene, #12260, 0.50 μg/reaction) using 6 μl Lipofectamine 2000 (Thermo Fisher Scientific, 11668019) in 500 μl Opti-MEM (Gibco)/reaction. Viral supernatants were centrifuged at 300 g for 5 min, filtered through 0.45 μm syringe filters (Starlab) and concentrated using 1/3 volume of Lenti-X concentrator (Clontech) as per the manufacturer's instructions. ID8 cells were transduced with lentivirus for 3 days before selection (7.5 μg/ml blasticidin, 2.5 μg/ml puromycin, 1.5 mg/ml G418) or FACS sorting.

Live 3D imaging and analysis

In a 96-well plate (Corning, 3595), 30 μl of 50% Growth Factor Reduced Matrigel (GFRM; Corning, 354230) was used to precoat each well. The plate was centrifuged for 3 min at 1,500 g at 4°C. Cells were seeded as single-cell suspensions supplemented with 2% GFRM (2,000 cells/well). The plate was centrifuged at 200 g for 2 min at room temperature (RT), incubated at 37°C for 4 h and scanned every hr for a total of 72 h using the Spheroid module of the IncuCyte S3 system (Sartorius) with a ×10 objective (1 field

imaged/well). Images were extracted and aligned using the Fiji plugin "Image stabiliser" and a custom-made Fiji macro. Custom pipelines in CellProfiler (v4.2.0) identified and tracked individual spheroids at each time point, while extracting information on their size, shape, movement and brightness variation (Freckmann *et al*, 2022). The generated data set was used in CellProfiler Analyst (v2.2.0) to apply user-supervised machine learning (FastGentle Boosting algorithm) and classify spheroids as "Out of Focus" or "In Focus" (accuracy > 80% according to confusion matrix). The shape, size and movement measurements of only "In Focus" spheroids were used again in CellProfiler Analyst to construct rules (Table EV5) and classify them based on their morphology as "Hyper-protrusive" or "Spherical" (Accuracy 92% according to confusion matrix). These rules were exported as .txt files and incorporated in a CellProfiler pipeline that would perform prospective classification of new data sets without the need for retraining. A custom KNIME Data Analytics Platform (v3.3.1) pipeline was used to collate data, $\log_2$ transform and normalise the proportion of phenotypes across conditions and time points, perform statistical analyses and generate heatmaps. Statistical tests are described in figure legends, and P-values are annotated on figures. Heatmaps were generated using ggplot2 (v3.3.0; Wicham, 2009) in the R environment (v3.6.2). Statistical comparison was performed in R using the Cochran–Mantel–Haenszel test wherein a comparison is only statistically significant where the effect was present across all biological replicates. Using the DescTools (v0.99.31; Andri *et al*, 2022) R package, the Breslow–Day statistic was used to test the assumption that the magnitude of effect of a condition is homogeneous across all strata (biological replicates): a nonsignificant *P*-value indicates homogeneity. In both statistical tests, a Bonferroni adjustment was applied to correct for multiple testing.

Cloning

Molecular cloning was performed using either classical ligation or In-Fusion technology. Restriction reactions were performed using High-Fidelity Restriction enzymes from New England Biolabs (NEB), by incubating 2 µg of DNA with 2 U of each enzyme in the presence of 10X NEB CutSmart buffer, diluted to the appropriate concentration in nuclease-free water. The restriction reaction was performed at 37°C (or other appropriate incubation temperatures) for 1 h. The digested products were stained with 6× DNA loading dye and resolved at 110 V for 1 h in 1% agarose in TAE buffer supplemented with Midori green (Nippon Genetics, MG04). The desired DNA was purified using the QIAquick Gel Extraction Kit (Qiagen, 28706X24) as per the manufacturer's instructions. For ligations using the Rapid DNA Ligation Kit (Roche, 11635379001), vector and insert were mixed in a 1:3 molar ratio, supplemented with 1× Dilution buffer, 1× Ligation buffer and 1 µl Ligase in a total volume of 10 µl, and incubated for 5 min at RT. For ligation reactions using the T4 DNA Ligase (NEB, M0202), the same molar ratio was used, supplemented with 2 µl of 10× T4 DNA Ligase buffer, 1 µl T4 DNA in a total of 20 µl. The reaction was performed at RT for 10 min and the ligase was subsequently heat-inactivated at 65°C for a further 10 min. For In-Fusion Cloning, a 1:3 vector to insert molar ratio was combined with 2 µl of 5× In-Fusion Reagent in a total volume of 10 µl. In-Fusion reaction was performed at 50°C for 15 min. Bacterial transformation was performed using either Stbl3 (Thermo Fisher Scientific, C737303) or Stellar (Takara, 636766) chemically competent cells using the bacteria:DNA ratio as per the manufacturer's instructions. A 10-min incubation on ice was followed by heat-shock of 45 s at 42°C. Transformed bacteria were plated on suitable agar plates and incubated overnight at 37°C.

CRISPR-Knock out (KO) screen

All gRNAs used in the screen were generated against the mouse genome (Ensemble v.100) using an online tool (https://portals.broadinstitute.org/gppx/crispick/public). The top 5 sgRNAs (as determined by the sgRNA Designer tool) were further interrogated at the Integrated DNA Technologies (IDT) website (https://eu.idtdna.com/site/order/designtool/index/CRISPR_SEQUENCE). Only those with high on-target potential and low off-target risk were retained. (all sgRNA sequences available in Table EV2). The pLentiCRISPRv2 Neo vector was used as a backbone and the cloning procedure followed the steps as described by the Zhang Lab (Sanjana *et al*, 2014; Shalem *et al*, 2014). From each oligo pair, 2 µl were combined with 1 µl 10× T4 Ligation Buffer (NEB, M0202), 6.5 µl Nuclease-free H2O and 0.5 µl T4 Polynucleotide Kinase (PNK) (NEB, M0201). The oligos were annealed in a thermocycler with gradual T reduction from 95 to 25°C at a rate of 5°C/min and subsequently diluted 1:20 into Nuclease-free water (Thermo Fisher Scientific, AM9938). The pLentiCRISPRv2 plasmid was digested for 1 h at 55°C with 1 U per µg of DNA BsmBI-v2 (NEB, R0580), in 5 µl Buffer 3.1 and diluted to a final volume of 50 µl. The digested backbone was dephosphorylated with 1 U/mg FastAP Thermosensitive Alkaline Phosphatase (Thermo Fisher Scientific, EF0651) for 10 min at 37°C. FastAP was inactivated at 75°C for 5 min. For ligation, 50 ng of digested plasmid was combined with 1 µl diluted oligo duplex, 1× Rapid DNA Ligation buffer, 1× Dilution buffer, nuclease-free water to a final volume of 10 µl and 1 µl Ligase (Roche, 11635379001). The mixture was incubated at RT for 5 min. Bacterial transformation was performed as described above.

The screen was performed in two phases. The first phase was performed in four iterations. The 4–5 gRNAs targeting each gene were pooled together and used to transfect HEK-293FT cells as described above. The viruses produced were then used to transduce *Trp53*$^{-/-}$;*Pten*$^{-/-}$ 1.15 cells and generate a single, stable cell line (Pooled KO) for each gene. The Pooled KO cell lines were imaged with the IncuCyte system as described above and compared with a Pooled sgNT cell line. Processing of images and data analysis was performed independently for each iteration as described above. The results are presented as fold change to the iteration's sgNT cell line and each iteration has been colour-coded to allow for easier comparison. Heatmaps were generated using ggplot2 (v3.3.0; Wicham, 2009) in the R environment (v3.6.2). Statistical comparison was performed in R using the Cochran–Mantel–Haenszel test wherein a comparison is only statistically significant where the effect was present across all biological replicates. Using the DescTools (0.99.31?) (Andri *et al*, 2022) R package, the Breslow–Day statistic was used to test the assumption that the magnitude of effect of a condition is homogeneous across all strata (biological replicates): a nonsignificant *P*-value indicates homogeneity. In both statistical tests, a Bonferroni adjustment was applied to correct for multiple testing. Select interactors were deconvoluted in Phase 2, where 4–5 distinct KO cell lines were

© 2023 The Authors

generated using each individual sgRNA and compared against a single sgNT cell line.

Fixed 3D and 2D imaging and analysis

For 2D samples, ID8 cells were seeded on a black-bottom 96-well plate (Greiner, 655090) with 2,000 cells per well and incubated for 24 h at 37°C. For 3D samples, ID8 spheroids were set up in eight-well chamber slides coated with 60 μl of 50% GFRM. 4,000 cells were seeded per well as single-cell suspensions supplemented with 2% GFRM and then incubated for 48 h. Spheroids or cells were washed once with PBS and fixed with 4% PFA for 15 min at RT. Blocking was achieved with PFS (0.7% fish skin gelatine and 0.025% saponin in PBS). The following antibodies were added at 1:200 dilution in PFS and incubated overnight at 4°C with gentle shaking: Collagen IV (Abcam, ab19808), pAKT pS473 (CST, 4060, D9E), pFAK pY397 (CST, 3283), pSRC Family pY416 (CST, 2101), V5-Tag (ABM, G189), PI3Kβ (Proteintech, 21739-1-AP), AGAP1 (TFS, 50542), ITGB1 (Merck, MAB1997), ITGA5 (BD Bioscience, 553319). Following three PFS washes, secondary antibodies Alexa Fluor 488 Donkey Anti-Mouse IgG (H + L) and/or Alexa Fluor 647 Donkey Anti- Mouse IgG (H + L) (Thermo Fisher Scientific, A21202 and A31571, respectively) were added in PFS (1:1,000) together with Alexa Fluor® 568 Phalloidin (Thermo Fisher Scientific, A12380, 1:200 dilution), HCS CellMask Deep Red Stain (1:50,000) and Hoechst 34580 (Thermo Fisher Scientific, H21486) (1:1,000) and incubated at RT for 45 min. Samples were further washed with PFS (twice) and with PBS (thrice). Invading monolayers and spheroids were imaged using a Zeiss 880 Laser Scanning Microscope with Airyscan using either confocal or super resolution functions. Images taken in super resolution mode were processed using the Zeiss proprietary ZEN 3.2 software, exported as TIFF files and processed in Fiji. Line scan intensity analysis on tips of invading protrusions was performed using Fiji. Invading monolayers and cells on 96-well plates were also imaged using an Opera Phenix high content analysis system (×20 or ×63) and the Columbus High-Content Imaging and Analysis Software (PerkinElmer, Version 2.9.1) was used to generate custom pipelines and perform object segmentation, intensity measurements and machine learning. For 2D morphology assays, cells were identified based on nuclear staining (Hoechst) and the shape of each cell was defined by CellMask staining. Machine learning and manual training was used to classify cells as either “elongated,” “cobblestone” or “round.” Each cell was imaged in 1 plane. Cells in contact with the image border were excluded. For measurement of pAKT enrichment, cells were identified based on nuclear staining (Hoechst), and the total cell area was defined by CellMask staining and cells in contact with the image border were excluded. The cell area was split into three Regions: Ring Region, or “Perinuclear,” resized to 35% Outer Border Shift (OBS) and 50% Inner Border Shift (IBS), “Membrane,” resized to −10% OBS and 10% IBS and “Cytoplasm,” resized to 10% OBS and 35% IBS. The cells were imaged in three planes with 1 μm distance between planes and processed as a maximum projection. The staining intensity of pAKT was measured in each cell, for each individual area and was expressed as a proportion of the total (sum of all areas). The $\log_2$-transformed values were plotted using a custom R pipeline. Due to the large number of values measured, only the means of each experimental replicate are shown as dot-plots overlaid on violin plots depicting the distribution of the normalised pAKT intensity values of all cells measured. Statistical tests are described in figure legends, and *P*-values are annotated on figures.

Invasion assay

Cell invasion was examined using the Scratch Wound assay method on the IncuCyte System (Zoom 1 or S3, Satorius). The wells of a 96-well IncuCyte Image Lock plate were coated with 20 μl of 1% GFRM (Corning, 354230) overnight and incubated at 37°C. The GFRM was removed and 6.5×10^4 cells were added per well and incubated at 37°C for 4 h to facilitate attachment. The IncuCyte Scratch Wound Tool was used as per the manufacturer’s instructions to create the wound. PBS was used to wash cell debris from the wells and 50 μl of 50% GFRM diluted in cell culture medium was placed on top of the cells and then incubated for 1 h at 37°C. If inhibitors /drugs were used; an appropriate volume was added in the GFRM to achieve the desired concentration. After incubation, 100 μl of cell culture medium (supplemented with inhibitors/drugs when required) was placed on top of the GFRM and the plate was imaged using the Scratch Wound module. Images were taken every 1 h using the ×10 objective and from a single field per well. Any wells where the wound did not form properly were not included in the analysis. Images were analysed using the dedicated IncuCyte analysis tool. For each time point, the relative wound density (RWD) was measured. Statistical analyses were performed, and graphs were generated using Microsoft Excel and RStudio (v1.4.1717). Data are presented for $t = 1/2$ max of Control condition as bee swarm “super-plots” (Lord *et al*, 2020). Statistical tests are annotated on figure legends, and *P*-values on Figs A similar approach was used for tracking of the leader cells, using the ×20 objective. A 1:2,000 dilution of IncuCyte NucLight Red dye (Sartorius, 4717) was added to stain nuclei and images were obtained every 15 min. Produced stacks were aligned using the Fiji plugin “Image stabiliser” and a custom Fiji macro. Leader cell tracking was performed using the MTrackJ plugin on Fiji. Spider plots were generated using RStudio (v1.4.1717). For scratch wound assays, fixed and stained for immunofluorescence, the same procedure was followed to set up cells as monolayers on black-bottom 96-well plates (Greiner, 655090) and a 20 μl pipette tip was used to manually form the wound. Following an incubation period of 19 h at 37°C, the invading monolayers were fixed and stained as described above.

RNA extraction and sequencing

RNA extractions were performed using the RNeasy kit (Qiagen, 74106) and the QIAshredder spin columns (Qiagen, 79656). For 2D samples, cells at 70–80% confluence were washed twice with PBS and lysed in 600 μl of buffer RLT with 6 μl β-mercaptoethanol for 2 min. Cells were scraped and homogenised using a QIAshredder spin column, centrifuged for 2 min at > 8,000 *g*. A 1:1 ratio of flow-through to 70% EtOH was mixed well and transferred onto a RNeasy Mini spin and the RNA isolated following the manufacturer’s instructions. The eluted RNA was stored at −80°C. For 3D spheroids, ID8 cells were passaged so they were sparse. The next day, six-well plates (Falcon, 353046) were coated using 180 μl of 50% GFRM per well and left to set for 60–75 min in an incubator at 37°C. Cells were washed, trypsinised, centrifuged, resuspended in

fresh media, counted and adjusted to 8 × 10^4 cells/ml. In each well, 1.6 ml of cell suspension supplemented with 2% GFRM was added per well, and spheroids allowed to develop for 2 days in an incubator at 37°C with 5% CO_2. For RNA extraction, cells were washed twice and the protocol for lysis was as described above for 2D samples. Adjustments were made to support the disruption of the ECM, by passing the lysates through a 25-27G needle slowly 10× before homogenisation using a QiaShredder. Lysis was performed using 350 µl of RLT buffer per well. For subsequent RNA sequencing of both 2D and 3D samples, extracted RNA underwent DNase treatment. An aliquot corresponding to 1.3 µg of RNA was obtained and combined with 1 µl 10× DNase I Reaction Buffer and 1.3 µl DNase 1 (1 U/µl) (Thermo Fisher Scientific, 18068015) to a final volume of 10 µl with RNase-free water. The RNA/DNase mix was incubated at RT for 15 min and the reaction was stopped with addition of 10% v/v EDTA and heat-inactivation at 65°C for 10 min. The DNase treated RNA was placed on ice. 300 ng of RNA was taken and diluted to 50–100 ng/µl and used for TapeStation quality control of samples with a RNA*I*ntegrity *S*core (RIN) of > 6 considered acceptable. The leftover 1 µg of RNA was brought to 50 µl volume with RNase-free water.

RNA sequencing and analysis

Sequencing was performed at the CRUK Beatson Institute using the Illumina polyAselection (2x36 PE Sequencing) kit without long reads. Quality checks and trimming on the raw fastq RNA-Seq data files were performed using FastQC version 0.11.9 (Andrews, 2010), FastP version 0.20.1 (Chen *et al*, 2018) and FastQ Screen version 0.14 (Wingett & Andrews, 2018). RNA-Seq paired-end reads were aligned to the GRCm38.101 version of the mouse genome and annotation (Yates *et al*, 2020), using HiSat2 version 2.2.1 (Kim *et al*, 2019) and sorted using Samtools version 1.7 (Li *et al*, 2009). Aligned genes were identified using Feature Counts from the SubRead package version 2.0.1 (Liao *et al*, 2014). Expression levels were determined and statistically analysed using the R environment version 4.0.3 (R Core Team, 2020) and utilising packages from the Bioconductor data analysis suite (Huber *et al*, 2015). Differential gene expression was analysed based on the negative binomial distribution using the DESeq2 package version 1.28.1 (Love *et al*, 2014) and adaptive shrinkage using Ashr (Stephens *et al*, 2020). Computational analysis was documented at each stage using MultiQC (Ewels *et al*, 2016), Jupyter Notebooks (Kluyver *et al*, 2016) and R Notebooks (RStudio Team, 2019). Log_2 Transformation of counts and heatmap generation was performed using PRISM.

Protein Domain-GST fusion purification

The PH domain sequences corresponding to the two isoforms of AGAP1 were ordered as GeneArt String DNA Fragments (Thermo Fisher Scientific) and cloned by In-Fusion as GST Fusions in pGEX-4T1 vector. Plasmids encoding GST-Control, GST-hAgap1_PH_L and GST-hAgap1_PH_S were transformed into Rosetta 2(DE3)pLysS (Novagen) and proteins were expressed in Luria Broth based auto-induction medium including trace elements (Formedium) at 37°C for 6.5 h followed by 18°C for 12 h. Cells were harvested by centrifugation and the resulting pellets were resuspended in 200 mM NaCl, 50 mM Tris–HCl, pH 7.6, 1 mM DTT, 2 mM PMSF prior to lysis with a microfluidizer at ~ 15,000 psi. Lysate was clarified by centrifugation, incubated with glutathione agarose resin (Agarose Bead Technologies), washed with resuspension buffer without PMSF and eluted with wash buffer containing 10 mM glutathione and 5 mM DTT. The glutathione agarose eluate was diluted to a concentration of 50 mM NaCl, applied to a 5 ml HiFliQ Q ion exchange FPLC column (Neo Biotech) and eluted with a linear gradient ranging from 50 to 600 mM NaCl in 50 mM Tris–HCl, pH 8.5. Selected fractions were combined and applied to a HiLoad 26/60 Superdex 75 (manufactured by GE Healthcare, now produced by Cytiva Life Sciences) equilibrated in 150 mM NaCl, 25 mM Tris–HCl, pH 7.6, 1 mM DTT. Protein concentration was based on the measured absorbance at 280 nm and calculated molar extinction coefficients (Wilkins *et al*, 1999) of 44,350, 73,800 and 66,810 M^{-1} cm^{-1} for GST-control, GST-hAGAP1_PH_L and GST-hAGAP1_PH_S, respectively.

BioID mass spectrometry proteomics and data analysis

An improved version of the promiscuous ligase BirA* (TurboID; Branon *et al*, 2018), was fused to the C terminus of ARF6, followed by a V5 Tag, a cleavable T2A peptide and BFP and cloned into a lentiviral vector. The construct was stably expressed in ID8 cells as described above. A construct lacking ARF6 but containing BirA*, V5, T2A and BFP was used as a negative control for nonspecific labelling. Cells at ~ 70–80% confluence were labelled for 30 min at 37°C by adding 50 µM of Biotin in full medium (Merck, S4501). Cells in Biotin-free medium were used as negative control. Cells were washed five times in ice-cold PBS and lysates were obtained by adding 800 µl of Lysis Buffer (50 mM Tris–HCl pH 7.4, 100 mM NaCl, 5 mM in MS-grade water) supplemented with one each of cOmplete™, Mini Protease Inhibitor (Roche, 05892970001) and PhosSTOP™ Phosphatase Inhibitor tablets (Roche, 04906837001). The lysates were scraped, incubated on ice for 30 min, sonicated and centrifuged at 13,600 *g* for 30 min at 4°C. Protein concentration was determined by performing a BCA assay (Pierce™ BCA Protein Assay Kit, Thermo Fisher Scientific, 23225, following the manufacturer's instructions). 350 µg of proteins was used per condition. 200 µl of streptavidin sepharose beads (Streptavidin Sepharose High Performance, Merck, GHC-17-5113-01) was washed thrice in 50 mM Tris–HCl pH 7.4. All samples were incubated with 25 µl prewashed beads in each at 4°C for 2 h with rotation. The beads were washed four times with 400 µl Washing Buffer (50 mM Tris pH 7.4, 100 mM NaCl, 5 mM EDTA) and each time centrifuged at 1,200 *g* for 1 min at 4°C. Samples were resuspended in 2 M urea in 100 mM ammonium bicarbonate buffer and stored at −20°C until further processing. On-bead digestion was performed from the supernatants. Quadruplicate biological replicates were digested with Lys-C (Alpha Laboratories) and trypsin (Promega) on beads as previously described (Hubner *et al*, 2010). Following trypsin digestion, peptides were separated by means of nanoscale C18 reverse-phase Liquid Chromatography (LC) using an EASY-nLC II 1200 (Thermo Fisher Scientific) system directly coupled to a mass spectrometer (Orbitrap Fusion Lumos, Thermo Fisher Scientific). Elution was performed using a 50-cm fused silica emitter (New Objective) packed in-house with ReproSil-Pur C18-AQ, 1.9 µm resin (Dr Maisch, GmbH). Separation was carried out using a 135 min binary gradient at flow rate of 300 nl/min. The packed emitter was maintained at 50°C by

© 2023 The Authors

means of a column oven (Sonation) integrated into the nanoelectrospray ion source (Thermo Fisher Scientific). Air contaminants signal levels were decreased using an Active Background Ion Reduction Device (ABIRD ESI Source Solutions). Data acquisition was performed using the Xcalibur software. A full scan was acquired over a mass range of 350–1,400 m/z at 60,000 resolution at 200 m/z. The 15 most intense ions underwent higher energy collisional dissociation fragmentation and the fragments generated were analysed in the Orbitrap (15,000 resolution). MaxQuant 1.6.14.0 was used for data processing. Data were processed with the MaxQuant software (Cox & Mann, 2008; Cox *et al*, 2011) querying SwissProt (UniProt, 2019) Mus musculus (25,198 entries). First and main searches were performed with precursor mass tolerances of 20 ppm and 4.5 ppm, respectively, and MS/MS tolerance of 20 ppm. The minimum peptide length was set to six amino acids and specificity for trypsin cleavage was required. Cysteine carbamidomethylation was set as fixed modification, whereas Methionine oxidation, Phosphorylation on Serine–Threonine-Tyrosine, and N-terminal acetylation were specified as variable modifications. The peptide, protein and site false discovery rate (FDR) was set to 1%. All MaxQuant outputs were analysed with the Perseus software version 1.6.2.3 (Tyanova *et al*, 2016). Protein abundance was measured using label-free quantification (LFQ) intensities, which were calculated according to the label-free quantification algorithm available in MaxQuant (Cox *et al*, 2014), reported in the ProteinGroups.txt file. Only proteins quantified in all three replicates in at least one group were used for further analysis. Missing values were inputted separately for each column (width 0.3, down shift 1.8), and significantly enriched proteins were selected using a permutation-based *t*-test with FDR set at 5% and s0 = 0. Processed data were filtered using Microsoft Excel to select the hits likely representing true interactions. Typically, proteins with Student's *t*-test difference in their LFQ value of > 1.2, when compared to ID8 *Trp53*$^{-/-}$;*Pten*$^{-/-}$ 1.15 TurboID, were considered as true interactors. Protein networks were visualised using Cytoscape (v3.9.1) and bubble heatmaps were generated using RStudio (v1.4.1717).

Integrin recycling assay

96-well ELISA plates were coated with 50 µl of integrin antibody at the optimised concentration diluted in 0.05 M Na_2CO_3 pH 9.6 at 4°C overnight and blocked with 5% BSA in TBS-T. Cells at 80% confluence were washed with cold PBS and surface labelling was achieved with 0.13 mg/ml sulfo-NHS-SS-Biotin for 1 h. For internalisation, cells were washed with cold PBS and treated with 12–14°C cell medium for 30 min at 37°C. Medium was removed, cells were washed with pH 8.6 buffer (50 mM Tris pH7.5, 100 mM NaCl, adjust pH with 10 M NaOH) and MesNa (95 mM of MesNa in pH 8.6 Buffer) was added to achieve thiol reduction at 4°C for 30 min. Cells were washed with PBS and prewarmed medium was added to induce recycling at 37°C for the annotated time points. Cells were washed with PBS and pH 8.6 Buffer, followed by another round of thiol reduction. The reaction was quenched with the addition of 1 ml 20 mM iodoacetamide at 4°C. Cells were lysed using 280 µl of lysis buffer (200 mM NaCl, 75 mM Tris, 15 mM NaF, 1,5 mM Na_3VO_4, 7.5 mM EDTA, 7.5 mM EGTA, 1.5% Triton X-100, 0.75% Igepal CA-630, 50 µg/ml leupeptin, 50 µg/ml aprotinin and 1 mM AEBSF). Lysates were scraped and syringed once through a 30 G needle, centrifuged at 13,000 *g* for 10 min at 4°C, added in the ELISA 96-well plates and incubated overnight at 4°C. The ELISA plate was extensively washed with PBS-T to remove the unbound material. Streptavidin-conjugated horseradish peroxidase in PBS-T (1:6.666) containing 0.1% BSA was added to each well for 1 h at 4°C. The plate was extensively washed with PBS-T and then with PBS to remove the Tween. For detection, 50 µl of Citrate/PO4 buffer (4 mM o-Phenylenediamine dihydrochloride corrected to pH 5.5 with H_2O_2) were added per well until colour in the total pool was well developed. The reaction was stopped with 50 µl of 8 M H_2SO_4 and absorbance was read at 490 nm.

Proliferation and cell death assays

ID8 cells were plated in a 96-well plate (2,000/well), 24 h either alone or in the presence of 1:1,000 dilution Sytox Green (Thermo Fisher Scientific, S7020). Imaging was carried out on IncuCyte ZOOM or S3 every hour for 48 h. Cell area (confluence) and the number of green objects over confluence were measured using the IncuCyte analysis software.

PCR genotyping for AGAP1 KO cell lines

AGAP1 KO cell lines (ID8 *Trp53*$^{-/-}$;*Pten*$^{-/-}$ 1.15 sg*Agap1*_2 and sg*Agap1*_3) and a control cell line (ID8 *Trp53*$^{-/-}$;*Pten*$^{-/-}$ sgNT) were allowed to reach 80% confluence. Genomic DNA isolation was performed using 500 µl Lysis Buffer per well of 6-well plate (100 mM Tris–HCl pH 8.5, 5 mM EDTA, 0.2% SDS, 200 mM NaCl supplemented with 10 µg/ml Proteinase K) and overnight incubation at 55°C. Extraction was performed using Phenol:Chloroform: Isoamyl Alcohol (25:24:1 v/v) The upper layer was retained, and the extraction repeated twice on the supernatants using 450 and 400 µl of chloroform. The final 450 µl were precipitated by adding 35 µl of 4 M Sodium Acetate pH 5.2 and 770 µl of 100% EtOH. The precipitate was spun at 14,000 rpm for 1 min, the supernatant removed and the DNA pellet washed twice with 1 ml of 70% EtOH. Following the removal of EtOH, the DNA pellet was left to air-dry for 5 min, resuspended in 250 µl TE buffer and incubated at 55°C with gentle shaking for 5 min to dissolve. An empty pUC19 vector was linearised by PCR (pUC 5′: 5′-TCTAGAGGATCCCCGGGTAC-3′, pUC3′: 5-CTGCAGGCATGCAAGCTTGG-3′). The NCB1 Blastn tool was used to find the Mus musculus *Agap1* gene and identify a 500 bp region with the target sequence in the middle for each of the gRNAs. Primers that would amplify the specified genomic regions were designed, including 20 bp complementary edges to the linearised pUC19 backbone. PCR was performed using the Q5® Hot Start Master mix (NEB M0491), supplemented with 10 µM of each primer and 20 ng of template in a final volume of 25 µl in the following conditions: initial denaturation: 98°C, 30 s, denaturation: 98°C, 30 s, annealing: 3–5°C lower than the Tm of the least stable primer in the reaction, 20 s, extension: 72°C, 20–30 s per kb; repeat Steps 1–3 for 30 cycle, final extension: 72°C, 2 min.

The PCR products were purified using a QIAquick PCR purification kit (Qiagen, 28104) and inserted into the linearised pUC19 backbone using the In-fusion reagent as described above. Single colonies were selected, the DNA was extracted (Qiagen, 27106X4) and the PCR products sequenced to identify CRISPR-derived Insertions

or Deletions (INDELs). The PCR primers used to amplify the genomic DNA were the following:
sg*Agap1*_2: Forward: 5′-CGGGGATCCTCTAGAGCACAGGTAGAGCCTTGCAT-3′, Reverse: 5’-CTTGCATGCCTGCAGGTGGCAGATGTCTGTCTGAG-3′
sg*Agap1*_3: Forward: 5′- CGGGGATCCTCTAGATGCAGAGTTCAAATTTCAAG-3′, Reverse: 5′- CTTGCATGCCTGCAGGCTCACCCCCCTTTGCCACTC-3′

Immunoblotting

For 2D samples, cells were plated for 48 h and allowed to reach 80% confluence. In the case of Nutlin3A treatment, the inhibitor was added 2 h before cell harvesting in fresh medium. For 3D samples, ID8 spheroids were generated in six-well plates as described in the RNA extraction protocol, and inhibitors were added at the time of plating. Plates/wells were washed with ice-cold PBS and lysed using RIPA Buffer (50 mM Tris, 150 mM NaCl, 1% NP-40 and 0.25% Na deoxycholate with cOmplete protease inhibitor cocktail and PhosSTOP tablets). Cells were scraped and lysates incubated on ice for 15 min and clarified by centrifugation at 216 *g* at 4°C for 15 min. For 3D samples, the lysates were also passed through 25–27 G needle. BCA Protein Assay kit (Pierce) was used to determine protein concentration in 2D samples while a control immunoblot using samples of known concentration was used in the case of 3D samples. SDS–PAGE was performed in MES buffer at 160 V for 1 h, using 10 or 12-well Bolt™ 4–12% Bis-Tris Plus Gels (Thermo Fisher Scientific, NW04122BOX or NW0412BOX) and proteins were transferred to PVDF membranes using the iBlot 2 transfer system (Thermo Fisher Scientific). Membranes were incubated for 1 h in Rockland blocking buffer (Rockland) and primary antibodies added overnight at 4°C (1:1,000 unless stated otherwise). Antibodies used were: anti-2A peptide (Merck, MABS2005, 3H4), anti-AKT pan (CST, 2920, 40D4), anti-ARF5 (Novus Biologicals, H0000281-M01, IB4), anti-ARF6 (Merck, A5230), anti-GAPDH (CST 2118, 14C10, 1:5,000), anti-α5 integrin (Abcam, ab150361), anti-β1 integrin (Merck, clone MB1.2), anti-AKT phospho S473 (CST, 4060, D9E), anti-S6RP phospo S235/236 (CST, 2217, 5G10), anti-S6RP (CST, 2217, 5G10), PTEN (CST, 9552), anti-RFP (used to detect BFP; Thermo Fisher Scientific, R10367), anti-Streptavidin-Horseradish Peroxidase (HRP) Conjugated (Thermo Fisher Scientific, SA10001), anti-TP53 (Abcam, ab26, diluted in 5% milk in TBS-T), anti-V5 Tag (ABM, G189), anti-Vinculin (Merck, V9131, 1:2,000). Secondary antibodies were added for 45 min, membranes were washed three times in TBST and imaged using a ChemiDoc Imager (BioRad) or Odyssey Imaging System (LI-COR Biosciences). Bands were quantified using Image Lab 6.1 (BioRad) or Image Studio Software 6.0 (LI-COR Biosciences). GAPDH or vinculin were used as loading controls for each immunoblot (representative sample integrity controls are shown in the figures). Statistics were performed using two-tailed *t*-test between a treatment and the control sample and all significant *P*-values are annotated on figures.

PIP strips

PIP strips (Tebu-bio, 117P-6001) were used as per the manufacturer’s instructions. The membranes were blocked for 1 h in PBS-T (0.1%) with 3% BSA, at RT. Each strip was incubated with 1 µg of purified GST or GST-PH Domain fusion in PBS-T with gentle agitation. Strips were washed thrice in PBS-T for 5 min. Anti-GST antibody (Merck, 06-332) was added diluted 1:1,000 in PBS-T with 3% BSA and incubated with gentle agitation in RT for 1 h. The strips were washed thrice with PBS-T and secondary HRP-conjugated antibody was added (1:5,000 in PBS-T 3% BSA) for 1 h at RT. Supersignal West Pico Plus Chemiluminescent Substrate (Thermo Fisher Scientific, 34580) was added for 3 min and the strips were scanned using the Bio-Rad ChemiDoc Imaging system.

Patient cohort analyses

Patient data were accessed, analysed and downloaded using in-platform tools from either cBioportal.org (Cerami *et al*, 2012; Gao *et al*, 2013; The Cancer Genome Atlas, https://www.cancer.gov/tcga, TCGA Ovarian Cancer Data set) or the Gene Expression for Normal and Tumour database (GENT2, http://gent2.appex.kr/gent2/). Graphs and statistical analyses were generated in PRISM 9 (GraphPad). Data sets can be accessed at cBioportal (TCGA, OV) or the Gene Expression Omnibus (GSE40595, GSE38666, GSE14407, GSE52460, GSE69428, GSE36668, GSE27651, GSE26712, GSE6008).

Statistical analysis

Sample size was not predetermined, and the data were not randomised prior to analysis. The number of biological and technical replicates are described in the figure legends. Where appropriate, the exact number of objects analysed is provided in Table EV1.

Data availability

The raw files and the MaxQuant search results files of the Mass Spectrometry experiment have been deposited to the ProteomeXchange Consortium (Deutsch *et al*, 2020) via the PRIDE partner repository (Perez-Riverol *et al*, 2022) with the data set identifier PXD038305 (http://www.ebi.ac.uk/pride/archive/projects/PXD038305). The RNA seq data have been deposited on the Sequence Read Archive (SRA), with ID Number PRJNA904679 (https://www.ncbi.nlm.nih.gov/bioproject/PRJNA904679).

Expanded View for this article is available online.

Acknowledgements
This work was supported by the following grants: DMB National Institute of Health (NIH) K99CA163535, Cancer Research UK (C596/A19481) ES, AR-F, DS and LM, Cancer Research UK C596/A17196 and A31287. KN was supported by a Cancer Research UK Glasgow Centre PhD studentship (C7932/A25170). EMC was supported by a University of Glasgow MVLS Doctoral Training Programme PhD Studentship. EF was supported by an industrial PhD studentship from Essen Bioscience and the University of Glasgow. SZ and SL were supported by Cancer Research UK (A29800 to SZ). LB and DTH, were supported by Cancer Research UK core funding to DTH (A23278). MN, RS and CM were supported by Cancer Research UK core funding to CM (A29801). IAM was supported by the Rivkin Center for Ovarian Cancer Research (574546) and Ovarian Cancer Action (P76567). JCN was supported by Cancer Research UK Core funding (A28291). We would like to thank the Core Services and Advanced Technologies at the Cancer Research UK Beatson

 © 2023 The Authors

Institute, with particular thanks to the Beatson Advanced Imaging Resource and Molecular Technologies. We would also like to thank Professor Stephen Tait, for provision of the pLenti CRISPR v2 Neo vector and Dr David Stevenson for provision of the pUC19 vector.

Author contributions

Konstantina Nikolatou: Data curation; formal analysis; validation; investigation; visualization; writing – original draft; writing – review and editing. **Emma Sandilands:** Data curation; formal analysis; validation; investigation; visualization; writing – original draft; writing – review and editing. **Alvaro Román-Fernández:** Data curation; formal analysis; validation; investigation; visualization. **Erin M Cumming:** Investigation. **Eva Freckmann:** Software; formal analysis; visualization; methodology. **Sergio Lilla:** Formal analysis; validation; investigation; methodology. **Lori Buetow:** Validation; investigation. **Lynn McGarry:** Formal analysis; validation; investigation. **Matthew Neilson:** Software. **Robin Shaw:** Formal analysis. **David Strachan:** Software. **Crispin Miller:** Supervision. **Danny T Huang:** Supervision. **Iain A McNeish:** Resources; methodology. **James C Norman:** Data curation; formal analysis; validation; investigation; visualization; methodology. **Sara Zanivan:** Supervision; methodology. **David M Bryant:** Conceptualization; data curation; formal analysis; supervision; funding acquisition; investigation; visualization; methodology; writing – original draft; project administration; writing – review and editing.

Disclosure and competing interests statement

EF was supported by a University of Glasgow Industrial Partnership Ph.D. scheme co-funded by Essen Bioscience, Sartorius Group. All other authors have no competing interests.

References

Ahmed AA, Etemadmoghadam D, Temple J, Lynch AG, Riad M, Sharma R, Stewart C, Fereday S, Caldas C, Defazio A *et al* (2010) Driver mutations in TP53 are ubiquitous in high grade serous carcinoma of the ovary. *J Pathol* 221: 49 – 56

Alessi DR, Andjelkovic M, Caudwell B, Cron P, Morrice N, Cohen P, Hemmings BA (1996) Mechanism of activation of protein kinase B by insulin and IGF-1. *EMBO J* 15: 6541 – 6551

Alessi DR, James SR, Downes CP, Holmes AB, Gaffney PR, Reese CB, Cohen P (1997) Characterization of a 3-phosphoinositide-dependent protein kinase which phosphorylates and activates protein kinase Balpha. *Curr Biol* 7: 261 – 269

Andrews S (2010) FastQC: a quality control tool for high throughput sequence data https://www.bioinformatics.babraham.ac.uk/projects/fastqc/

Andri S, Aho K, Alfons A, Anderegg N, Aragon T, Arachchige C, Arppe A, Baddeley A, Barton K, Bolker B *et al* (2022) DescTools: tools for descriptive statistics. R package version 0.99.46 https://cran.r-project.org/web/packages/DescTools/index.html

Benabdi S, Peurois F, Nawrotek A, Chikireddy J, Caneque T, Yamori T, Shiina I, Ohashi Y, Dan S, Rodriguez R *et al* (2017) Family-wide analysis of the inhibition of Arf guanine nucleotide exchange factors with small molecules: evidence of unique inhibitory profiles. *Biochemistry* 56: 5125 – 5133

Bonome T, Levine DA, Shih J, Randonovich M, Pise-Masison CA, Bogomolniy F, Ozbun L, Brady J, Barrett JC, Boyd J *et al* (2008a) A gene signature predicting for survival in suboptimally debulked patients with ovarian cancer. *Cancer Res* 68: 5478 – 5486

Bonome T, Levine DA, Shih J, Randonovich M, Pise-Masison CA, Bogomolniy F, Ozbun L, Brady J, Barrett JC, Boyd J *et al* (2008b) Gene Expression Omnibus GSE26712 (https://www.ncbi.nlm.nih.gov/geo/query/acc.cgi?acc=GSE26712). [DATASET]

Bowen NJ, Walker LD, Matyunina LV, Logani S, Totten KA, Benigno BB, McDonald JF (2009a) Gene expression profiling supports the hypothesis that human ovarian surface epithelia are multipotent and capable of serving as ovarian cancer initiating cells. *BMC Med Genomics* 2: 71

Bowen NJ, Walker LD, Matyunina LV, Logani S, Totten KA, Benigno BB, McDonald JF (2009b) Gene Expression Omnibus GSE14407 (https://www.ncbi.nlm.nih.gov/geo/query/acc.cgi?acc=GSE14407) [DATASET]

Branon TC, Bosch JA, Sanchez AD, Udeshi ND, Svinkina T, Carr SA, Feldman JL, Perrimon N, Ting AY (2018) Efficient proximity labeling in living cells and organisms with TurboID. *Nat Biotechnol* 36: 880 – 887

Brown HA, Gutowski S, Moomaw CR, Slaughter C, Sternweis PC (1993) ADP-ribosylation factor, a small GTP-dependent regulatory protein, stimulates phospholipase D activity. *Cell* 75: 1137 – 1144

Cantley LC, Neel BG (1999) New insights into tumor suppression: PTEN suppresses tumor formation by restraining the phosphoinositide 3-kinase/AKT pathway. *Proc Natl Acad Sci USA* 96: 4240 – 4245

Cerami E, Gao J, Dogrusoz U, Gross BE, Sumer SO, Aksoy BA, Jacobsen A, Byrne CJ, Heuer ML, Larsson E *et al* (2012) The cBio cancer genomics portal: an open platform for exploring multidimensional cancer genomics data. *Cancer Discov* 2: 401 – 404

Chen S, Zhou Y, Chen Y, Gu J (2018) fastp: an ultra-fast all-in-one FASTQ preprocessor. *Bioinformatics* 34: i884 – i890

Cockcroft S, Thomas GM, Fensome A, Geny B, Cunningham E, Gout I, Hiles I, Totty NF, Truong O, Hsuan JJ (1994) Phospholipase D: a downstream effector of ARF in granulocytes. *Science* 263: 523 – 526

Cox J, Mann M (2008) MaxQuant enables high peptide identification rates, individualized p.p.b.-range mass accuracies and proteome-wide protein quantification. *Nat Biotechnol* 26: 1367 – 1372

Cox J, Neuhauser N, Michalski A, Scheltema RA, Olsen JV, Mann M (2011) Andromeda: a peptide search engine integrated into the MaxQuant environment. *J Proteome Res* 10: 1794 – 1805

Cox J, Hein MY, Luber CA, Paron I, Nagaraj N, Mann M (2014) Accurate proteome-wide label-free quantification by delayed normalization and maximal peptide ratio extraction, termed MaxLFQ. *Mol Cell Proteomics* 13: 2513 – 2526

Cronin TC, DiNitto JP, Czech MP, Lambright DG (2004) Structural determinants of phosphoinositide selectivity in splice variants of Grp1 family PH domains. *EMBO J* 23: 3711 – 3720

Cunningham F, Allen JE, Allen J, Alvarez-Jarreta J, Amode MR, Armean IM, Austine-Orimoloye O, Azov AG, Barnes I, Bennett R *et al* (2022) Ensembl 2022. *Nucleic Acids Res* 50: D988 – D995

Deutsch EW, Bandeira N, Sharma V, Perez-Riverol Y, Carver JJ, Kundu DJ, Garcia-Seisdedos D, Jarnuczak AF, Hewapathirana S, Pullman BS *et al* (2020) The ProteomeXchange consortium in 2020: enabling 'big data' approaches in proteomics. *Nucleic Acids Res* 48: D1145 – D1152

D'Souza-Schorey C, Chavrier P (2006) ARF proteins: roles in membrane traffic and beyond. *Nat Rev Mol Cell Biol* 7: 347 – 358

Elgaaen BV, Olstad OK, Sandvik L, Odegaard E, Sauer T, Staff AC, Gautvik KM (2012a) ZNF385B and VEGFA are strongly differentially expressed in serous ovarian carcinomas and correlate with survival. *PloS One* 7: e46317

Elgaaen BV, Olstad OK, Sandvik L, Odegaard E, Sauer T, Staff AC, Gautvik KM (2012b) Gene Expression Omnibus GSE36668 (https://www.ncbi.nlm.nih.gov/geo/query/acc.cgi?acc=GSE36668) [DATASET]

Ewels P, Magnusson M, Lundin S, Kaller M (2016) MultiQC: summarize analysis results for multiple tools and samples in a single report. *Bioinformatics* 32: 3047 – 3048

Freckmann EC, Sandilands E, Cumming E, Neilson M, Roman-Fernandez A, Nikolatou K, Nacke M, Lannagan TRM, Hedley A, Strachan D *et al* (2022) Traject3d allows label-free identification of distinct co-occurring phenotypes within 3D culture by live imaging. *Nat Commun* 13: 5317

Gao J, Aksoy BA, Dogrusoz U, Dresdner G, Gross B, Sumer SO, Sun Y, Jacobsen A, Sinha R, Larsson E *et al* (2013) Integrative analysis of complex cancer genomics and clinical profiles using the cBioPortal. *Sci Signal* 6: pl1

Gassama-Diagne A, Yu W, ter Beest M, Martin-Belmonte F, Kierbel A, Engel J, Mostov K (2006) Phosphatidylinositol-3,4,5-trisphosphate regulates the formation of the basolateral plasma membrane in epithelial cells. *Nat Cell Biol* 8: 963 – 970

Hammond GR, Balla T (2015) Polyphosphoinositide binding domains: Key to inositol lipid biology. *Biochim Biophys Acta* 1851: 746 – 758

Hill CG, Matyunina LV, Walker D, Benigno BB, McDonald JF (2014a) Transcriptional override: a regulatory network model of indirect responses to modulations in microRNA expression. *BMC Syst Biol* 8: 36

Hill CG, Matyunina LV, Walker D, Benigno BB, McDonald JF (2014b) Gene Expression Omnibus GSE52460 (https://www.ncbi.nlm.nih.gov/geo/query/acc.cgi?acc=GSE52460) [DATASET]

Hoadley KA, Yau C, Wolf DM, Cherniack AD, Tamborero D, Ng S, Leiserson MDM, Niu B, McLellan MD, Uzunangelov V *et al* (2014) Multiplatform analysis of 12 cancer types reveals molecular classification within and across tissues of origin. *Cell* 158: 929 – 944

Honda A, Nogami M, Yokozeki T, Yamazaki M, Nakamura H, Watanabe H, Kawamoto K, Nakayama K, Morris AJ, Frohman MA *et al* (1999) Phosphatidylinositol 4-phosphate 5-kinase alpha is a downstream effector of the small G protein ARF6 in membrane ruffle formation. *Cell* 99: 521 – 532

Huber W, Carey VJ, Gentleman R, Anders S, Carlson M, Carvalho BS, Bravo HC, Davis S, Gatto L, Girke T *et al* (2015) Orchestrating high-throughput genomic analysis with bioconductor. *Nat Methods* 12: 115 – 121

Hubner NC, Bird AW, Cox J, Splettstoesser B, Bandilla P, Poser I, Hyman A, Mann M (2010) Quantitative proteomics combined with BAC TransgeneOmics reveals *in vivo* protein interactions. *J Cell Biol* 189: 739 – 754

Kim D, Paggi JM, Park C, Bennett C, Salzberg SL (2019) Graph-based genome alignment and genotyping with HISAT2 and HISAT-genotype. *Nat Biotechnol* 37: 907 – 915

King ER, Tung CS, Tsang YT, Zu Z, Lok GT, Deavers MT, Malpica A, Wolf JK, Lu KH, Birrer MJ *et al* (2011a) The anterior gradient homolog 3 (AGR3) gene is associated with differentiation and survival in ovarian cancer. *Am J Surg Pathol* 35: 904 – 912

King ER, Tung CS, Tsang YT, Zu Z, Lok GT, Deavers MT, Malpica A, Wolf JK, Lu KH, Birrer MJ *et al* (2011b) Gene Expression Omnibus GSE27651 (https://www.ncbi.nlm.nih.gov/geo/query/acc.cgi?acc=GSE27651) [DATASET]

Klarlund JK, Tsiaras W, Holik JJ, Chawla A, Czech MP (2000) Distinct polyphosphoinositide binding selectivities for pleckstrin homology domains of GRP1-like proteins based on diglycine versus triglycine motifs. *J Biol Chem* 275: 32816 – 32821

Kluyver T, Ragan-Kelley B, Pérez F, Granger B, Bussonnier M, Frederic J, Kelley K, Hamrick J, Grout J, Corlay S *et al* (2016) Jupyter Notebooks – a publishing format for reproducible computational workflows. In *Positioning and power in academic publishing: players, agents and agendas*, Loizides F, Scmidt B (eds), pp 87 – 90. Amsterdam, The Netherlands: IOS Press

Kutateladze TG (2010) Translation of the phosphoinositide code by PI effectors. *Nat Chem Biol* 6: 507 – 513

Lawrence MS, Stojanov P, Mermel CH, Robinson JT, Garraway LA, Golub TR, Meyerson M, Gabriel SB, Lander ES, Getz G (2014) Discovery and saturation analysis of cancer genes across 21 tumour types. *Nature* 505: 495 – 501

Li H, Handsaker B, Wysoker A, Fennell T, Ruan J, Homer N, Marth G, Abecasis G, Durbin R, Genome Project Data Processing S (2009) The Sequence Alignment/Map format and SAMtools. *Bioinformatics* 25: 2078 – 2079

Liao Y, Smyth GK, Shi W (2014) featureCounts: an efficient general purpose program for assigning sequence reads to genomic features. *Bioinformatics* 30: 923 – 930

Lili LN, Matyunina LV, Walker LD, Benigno BB, McDonald JF (2013a) Molecular profiling predicts the existence of two functionally distinct classes of ovarian cancer stroma. *Biomed Res Int* 2013: 846387

Lili LN, Matyunina LV, Walker LD, Benigno BB, McDonald JF (2013b) Gene Expression Omnibus GSE38666 (https://www.ncbi.nlm.nih.gov/geo/query/acc.cgi?acc=GSE38666) [DATASET]

Lord SJ, Velle KB, Mullins RD, Fritz-Laylin LK (2020) SuperPlots: communicating reproducibility and variability in cell biology. *J Cell Biol* 219: e202001064

Loskutov YV, Kozyulina PY, Kozyreva VK, Ice RJ, Jones BC, Roston TJ, Smolkin MB, Ivanov AV, Wysolmerski RB, Pugacheva EN (2015) NEDD9/Arf6-dependent endocytic trafficking of matrix metalloproteinase 14: a novel mechanism for blocking mesenchymal cell invasion and metastasis of breast cancer. *Oncogene* 34: 3662 – 3675

Love MI, Huber W, Anders S (2014) Moderated estimation of fold change and dispersion for RNA-seq data with DESeq2. *Genome Biol* 15: 550

Maehama T, Dixon JE (1998) The tumor suppressor, PTEN/MMAC1, dephosphorylates the lipid second messenger, phosphatidylinositol 3,4,5-trisphosphate. *J Biol Chem* 273: 13375 – 13378

Malek M, Kielkowska A, Chessa T, Anderson KE, Barneda D, Pir P, Nakanishi H, Eguchi S, Koizumi A, Sasaki J *et al* (2017) PTEN regulates PI(3,4)P2 signaling downstream of class I PI3K. *Mol Cell* 68: 566 – 580.e10

Martin-Belmonte F, Gassama A, Datta A, Yu W, Rescher U, Gerke V, Mostov K (2007) PTEN-mediated apical segregation of phosphoinositides controls epithelial morphogenesis through Cdc42. *Cell* 128: 383 – 397

Myers MP, Pass I, Batty IH, Van der Kaay J, Stolarov JP, Hemmings BA, Wigler MH, Downes CP, Tonks NK (1998) The lipid phosphatase activity of PTEN is critical for its tumor supressor function. *Proc Natl Acad Sci USA* 95: 13513 – 13518

Nacke M, Sandilands E, Nikolatou K, Roman-Fernandez A, Mason S, Patel R, Lilla S, Yelland T, Galbraith LCA, Freckmann EC *et al* (2021) An ARF GTPase module promoting invasion and metastasis through regulating phosphoinositide metabolism. *Nat Commun* 12: 1623

Nie Z, Stanley KT, Stauffer S, Jacques KM, Hirsch DS, Takei J, Randazzo PA (2002) AGAP1, an endosome-associated, phosphoinositide-dependent ADP-ribosylation factor GTPase-activating protein that affects actin cytoskeleton. *J Biol Chem* 277: 48965 – 48975

Oh SJ, Santy LC (2012) Phosphoinositide specificity determines which cytohesins regulate beta1 integrin recycling. *J Cell Sci* 125: 3195 – 3201

Palacios F, Schweitzer JK, Boshans RL, D'Souza-Schorey C (2002) ARF6-GTP recruits Nm23-H1 to facilitate dynamin-mediated endocytosis during adherens junctions disassembly. *Nat Cell Biol* 4: 929 – 936

Patch A-M, Christie EL, Etemadmoghadam D, Garsed DW, George J, Fereday S, Nones K, Cowin P, Alsop K, Bailey PJ *et al* (2015) Whole–genome characterization of chemoresistant ovarian cancer. *Nature* 521: 489 – 494

Perez-Riverol Y, Bai J, Bandla C, Garcia-Seisdedos D, Hewapathirana S, Kamatchinathan S, Kundu DJ, Prakash A, Frericks-Zipper A, Eisenacher M *et al* (2022) The PRIDE database resources in 2022: a hub for mass

 © 2023 The Authors

spectrometry-based proteomics evidences. *Nucleic Acids Res* 50: D543 – D552
Powelka AM, Sun J, Li J, Gao M, Shaw LM, Sonnenberg A, Hsu VW (2004) Stimulation-dependent recycling of integrin beta1 regulated by ARF6 and Rab11. *Traffic* 5: 20 – 36
R Core Team (2020) *R: a language and environment for statistical computing*. Vienna, Austria: R Foundation for Statistical Computing
Ratcliffe CDH, Siddiqui N, Coelho PP, Laterreur N, Cookey TN, Sonenberg N, Park M (2019) HGF-induced migration depends on the PI(3,4,5)P3-binding microexon-spliced variant of the Arf6 exchange factor cytohesin-1. *J Cell Biol* 218: 285 – 298
Roberts M, Barry S, Woods A, van der Sluijs P, Norman J (2001) PDGF-regulated rab4-dependent recycling of alphavbeta3 integrin from early endosomes is necessary for cell adhesion and spreading. *Curr Biol* 11: 1392 – 1402
Roman-Fernandez A, Roignot J, Sandilands E, Nacke M, Mansour MA, McGarry L, Shanks E, Mostov KE, Bryant DM (2018) The phospholipid PI (3,4)P2 is an apical identity determinant. *Nat Commun* 9: 5041
RStudio Team (2019) *RStudio: integrated development environment for R*. Boston, MA: RStudio, Inc.
Sanjana NE, Shalem O, Zhang F (2014) Improved vectors and genome-wide libraries for CRISPR screening. *Nat Methods* 11: 783 – 784
Sarbassov DD, Guertin DA, Ali SM, Sabatini DM (2005) Phosphorylation and regulation of Akt/PKB by the rictor-mTOR complex. *Science* 307: 1098 – 1101
Shalem O, Sanjana NE, Hartenian E, Shi X, Scott DA, Mikkelson T, Heckl D, Ebert BL, Root DE, Doench JG *et al* (2014) Genome-scale CRISPR-Cas9 knockout screening in human cells. *Science* 343: 84 – 87
Shewan A, Eastburn DJ, Mostov K (2011) Phosphoinositides in cell architecture. *Cold Spring Harb Perspect Biol* 3: a004796
Stephens M, Carbonetto P, Gerard D, Lu M, Sun L, Willwerscheid J, Xiao N (2020) Methods for adaptive shrinkage, using empirical Bayes. R package ashr version 2.2-47 https://cran.r-project.org/web/packages/ashr/index.html
Tamura M, Gu J, Matsumoto K, Aota S, Parsons R, Yamada KM (1998) Inhibition of cell migration, spreading, and focal adhesions by tumor suppressor PTEN. *Science* 280: 1614 – 1617
Taylor BS, Schultz N, Hieronymus H, Gopalan A, Xiao Y, Carver BS, Arora VK, Kaushik P, Cerami E, Reva B *et al* (2010) Integrative genomic profiling of human prostate cancer. *Cancer Cell* 18: 11 – 22
Tsai MT, Katagiri N, Ohbayashi N, Iwasaki K, Ohkohchi N, Ding ST, Kanaho Y, Funakoshi Y (2017) Regulation of HGF-induced hepatocyte proliferation by the small GTPase Arf6 through the PIP2-producing enzyme PIP5K1A. *Sci Rep* 7: 9438
Tyanova S, Temu T, Sinitcyn P, Carlson A, Hein MY, Geiger T, Mann M, Cox J (2016) The Perseus computational platform for comprehensive analysis of (prote)omics data. *Nat Methods* 13: 731 – 740
Walton J, Blagih J, Ennis D, Leung E, Dowson S, Farquharson M, Tookman LA, Orange C, Athineos D, Mason S *et al* (2016) CRISPR/Cas9-mediated Trp53 and Brca2 knockout to generate improved murine models of ovarian high-grade serous carcinoma. *Cancer Res* 76: 6118 – 6129
Walton JB, Farquharson M, Mason S, Port J, Kruspig B, Dowson S, Stevenson D, Murphy D, Matzuk M, Kim J *et al* (2017) CRISPR/Cas9-derived models of ovarian high grade serous carcinoma targeting Brca1, Pten and Nf1, and correlation with platinum sensitivity. *Sci Rep* 7: 16827
Watt SA, Kular G, Fleming IN, Downes CP, Lucocq JM (2002) Subcellular localization of phosphatidylinositol 4,5-bisphosphate using the pleckstrin homology domain of phospholipase C delta1. *Biochem J* 363: 657 – 666
Wicham H (2009) *Ggplot2: elegant graphics for data analysis*. New York, NY: Springer
Wilkins MR, Gasteiger E, Bairoch A, Sanchez JC, Williams KL, Appel RD, Hochstrasser DF (1999) Protein identification and analysis tools in the ExPASy server. *Methods Mol Biol* 112: 531 – 552
Wingett SW, Andrews S (2018) FastQ Screen: a tool for multi-genome mapping and quality control. *F1000Res* 7: 1338
Wu R, Hendrix-Lucas N, Kuick R, Zhai Y, Schwartz DR, Akyol A, Hanash S, Misek DE, Katabuchi H, Williams BO *et al* (2007a) Mouse model of human ovarian endometrioid adenocarcinoma based on somatic defects in the Wnt/beta-catenin and PI3K/Pten signaling pathways. *Cancer Cell* 11: 321 – 333
Wu R, Hendrix-Lucas N, Kuick R, Zhai Y, Schwartz DR, Akyol A, Hanash S, Misek DE, Katabuchi H, Williams BO *et al* (2007b) Gene Expression Omnibus GSE6008 (https://www.ncbi.nlm.nih.gov/geo/query/acc.cgi?acc=GSE6008) [DATASET]
Yamamoto Y, Ning G, Howitt BE, Mehra K, Wu L, Wang X, Hong Y, Kern F, Wei TS, Zhang T *et al* (2016a) *In vitro* and *in vivo* correlates of physiological and neoplastic human Fallopian tube stem cells. *J Pathol* 238: 519 – 530
Yamamoto Y, Ning G, Howitt BE, Mehra K, Wu L, Wang X, Hong Y, Kern F, Wei TS, Zhang T *et al* (2016b) Gene Expression Omnibus GSE69428 (https://www.ncbi.nlm.nih.gov/geo/query/acc.cgi?acc=GSE69428) [DATASET]
Yates AD, Achuthan P, Akanni W, Allen J, Allen J, Alvarez-Jarreta J, Amode MR, Armean IM, Azov AG, Bennett R *et al* (2020) Ensembl 2020. *Nucleic Acids Res* 48: D682 – D688
Yemelyanova A, Vang R, Kshirsagar M, Lu D, Marks MA, Shih Ie M, Kurman RJ (2011) Immunohistochemical staining patterns of p53 can serve as a surrogate marker for TP53 mutations in ovarian carcinoma: an immunohistochemical and nucleotide sequencing analysis. *Mod Pathol* 24: 1248 – 1253
Yeung TL, Leung CS, Wong KK, Samimi G, Thompson MS, Liu J, Zaid TM, Ghosh S, Birrer MJ, Mok SC (2013a) TGF-beta modulates ovarian cancer invasion by upregulating CAF-derived versican in the tumor microenvironment. *Cancer Res* 73: 5016 – 5028
Yeung TL, Leung CS, Wong KK, Samimi G, Thompson MS, Liu J, Zaid TM, Ghosh S, Birrer MJ, Mok SC (2013b) Gene Expression Omnibus GSE40595 (https://www.ncbi.nlm.nih.gov/geo/query/acc.cgi?acc=GSE40595) [DATASET]

License: This is an open access article under the terms of the Creative Commons Attribution License, which permits use, distribution and reproduction in any medium, provided the original work is properly cited.

Expanded View Figures

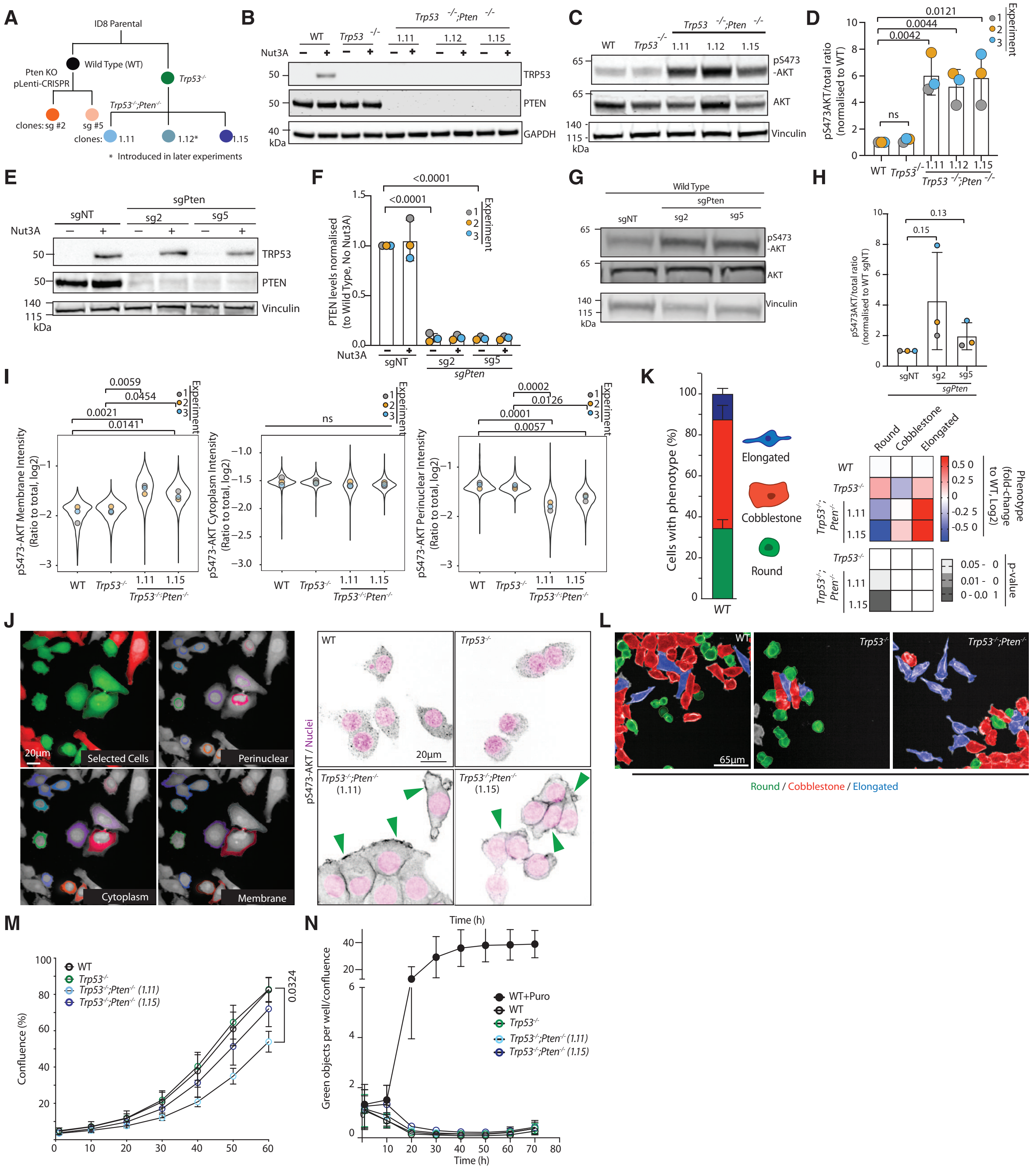

Figure EV1.

 © 2023 The Authors

◀ **Figure EV1. Characterisation of *Pten* loss effect on PI3K-AKT in 2D culture.**

A Schema, derivation of *Pten* and *Trp53* alterations in ID8 sublines.

B, C Western blot in ID8 sublines. (B) TRP53, PTEN, GAPDH expression upon Nutlin-3A (MDM2 inhibitor) treatment to stabilise P53 or (C) pS473-AKT, AKT, Vinculin (VCL) expression. Each panel is representative of $n = 3$ lysate preparations for each subline. GAPDH and VCL are loading controls for each panel.

D Quantitation of (C). Data, mean ± SD of pS473-AKT/total AKT intensity ratio, normalised to WT. Unpaired, two-tailed *t*-test; *P*-values, annotated.

E Western blot, TRP53, PTEN, VCL in ID8 Wild Type cells expressing non-targeting (sgNT) or *Pten*-targeting sgRNA upon Nutlin-3A (MDM2 inhibitor) treatment. Representative of $n = 3$ lysate preparations for each subline. VCL is loading control.

F Quantitation of PTEN band intensity from (E). Data, mean ± SD of band intensity, normalised to ID8 Wild-Type sgNT. Unpaired, two-tailed *t*-test; *P*-values, annotated.

G Western blot, pAKT(S473), AKT pan, VCL in ID8 Wild Type cells expressing non-targeting (sgNT) or *Pten*-targeting sgRNA. Representative of $n = 3$ lysate preparations for each subline. VCL is loading control.

H Quantitation of (G). p:t AKT ratio. Data, mean ± SD of band intensity, normalised to ID8 Wild-Type sgNT. Unpaired, two-tailed *t*-test; *P*-values, annotated.

I Quantitation of (J). Data, ratio pS473-AKT signal at indicated regions to total area. Means, overlaid on plots of all data points (exact number per replicate provided in Table EV1) as distinctly coloured dots according to culture replicate number. *P*-values are annotated, ANOVA with Tukey's honest significant difference (HSD) test.

J ID8 cells plated in 2D, stained with pS473-AKT (grey) and Hoechst (Magenta) (bottom panels), segmented into indicated regions (perinuclear, cytoplasmic, membrane) (top panels). Colour in selected cells panel: red, excluded due to touching image edge, green, included for segmentation. Arrowheads, pS473-AKT at cell membrane. Scale bar, 20 μm. $N = 3$ independent experiments, four technical replicates/subline/experiment. Total cell number per condition, Table EV1.

K Percentage of cells classified as Round (green), Cobblestone (red) or Elongated (blue) in Wild Type ID8 cells. Classification of ID8 WT, *Trp53*$^{-/-}$ and *Trp53*$^{-/-}$;*Pten*$^{-/-}$ *1.15* cells grown in 2D as Round, Cobblestone, or Elongated. Heatmap, $\log_2$ fold change, mean proportion across indicated lines. Grayscale heatmap, *P*-values for each comparison. $N = 2$ independent experiments, four technical replicates/subline/experiment. Total cell number per condition, Table EV1.

L Representative images of cells quantified in (K). Cells classified by shape (Round, green; Cobblestone, red; Elongated, blue). Scale bar, 65 μm.

M Proliferation assay based on well confluence over time. $N = 3$ experiments set up with repeated cultures of each subline, 4–5 technical replicates/subline/experiment. Data are presented as mean ± SD. Unpaired, two-tailed *t*-test between WT and each of the sublines per time point. Significant *P*-values annotated.

N Cell death assay, green object (Sytox green fluorescence) confluence over time. $N = 2$ experiments set up with repeated cultures of each subline, 4 technical replicates/subline in each experiment. Kruskal–Wallis ANOVA was performed at $t = 10$ h and $t = 20$ h, all comparisons are nonsignificant (P-value > 0.05).

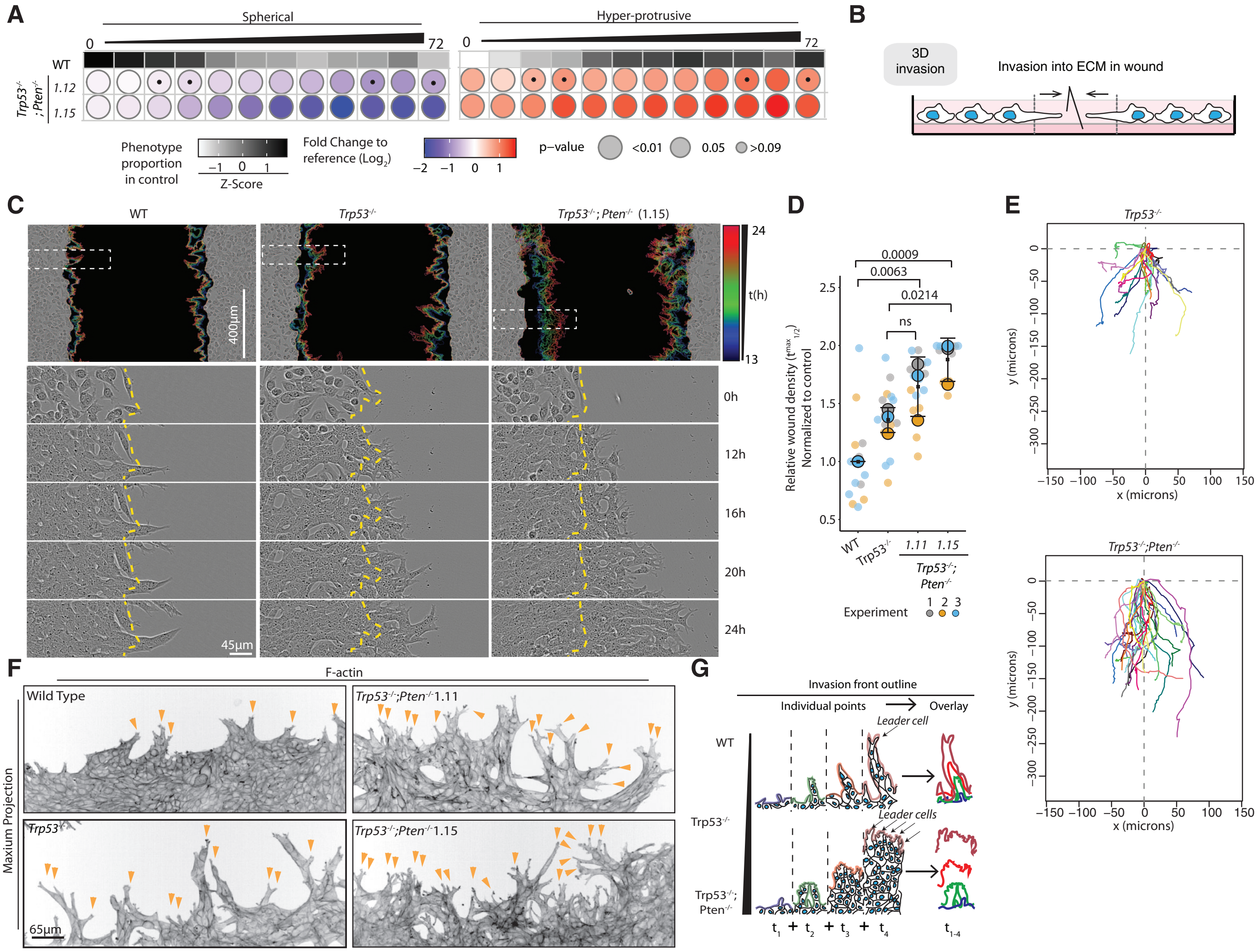

Figure EV2. Collective invasion into ECM in orthogonal assays.

A Frequency of Spherical and Hyper-protrusive phenotypes in ID8 WT, *Trp53*$^{-/-}$; *Trp53*$^{-/-}$;*Pten*$^{-/-}$ clones 1.12 and 1.15. spheroids, 6-h time intervals over 72 h. Heatmap (grayscale)—phenotype proportion (*z*-score) in control (WT). Heatmap (blue-red)—$\log_2$ fold change from control. *P*-values, bubble size (Cochran–Mantel–Haenszel test with Bonferroni adjustment). Black dot, homogenous effect across independent experiments (Breslow–Day test, Bonferroni adjustment, nonsignificant). *N* = 3 independent experiments, 3–4 technical replicates/experiment. Total spheroid number per condition, Table EV1.

B Schema, 3D invasion into ECM of wounded ID8 monolayer.

C Representative images of 3D invasion assay, monolayers plated onto ECM, wounded and then overlaid with 50% ECM (gel). Outlines of invasive front at different time points, pseudocoloured by time (rainbow legend), overlaid as concatenate on phase image of initial wound. Boxes, regions for different timepoints. Yellow lines, initial wound. Scale bar, 400 or 45 μm (indicated).

D Quantification of (C). Graph, Relative Wound Density (RWD) at $t_{1/2}$ max (time when WT 50% closed). Data, mean (black square) ± SD for 3 independent experiments (large circles) with 3–6 technical replicates/subline/experiment (small circles). ANOVA with Tukey's HSD test; exact *P*-values, annotated; ns, nonsignificant.

E Representative spider plots, leader cell movement in first 19 h of invasion of *Trp53*$^{-/-}$ and *Trp53*$^{-/-}$;*Pten*$^{-/-}$ 1.15 ID8 cells. *N* = 2 independent experiments, 10–25 leader cells tracked in each, across 4–6 technical replicates/experiment.

F Confocal images of wounded monolayer invasive fronts, stained for F-actin (Phalloidin). Arrowheads, protrusion tips. Scale bar, 65 μm. Representative of 7–12 fields imaged across *n* = 3 independent experiments, 4 technical replicates/experiment.

G Schema, loss of PTEN phenotype, a sheet-like mode of invasion with most ECM-abutting cells acting as "leader cells," compared with leader and follower cell chains in WT.

 © 2023 The Authors

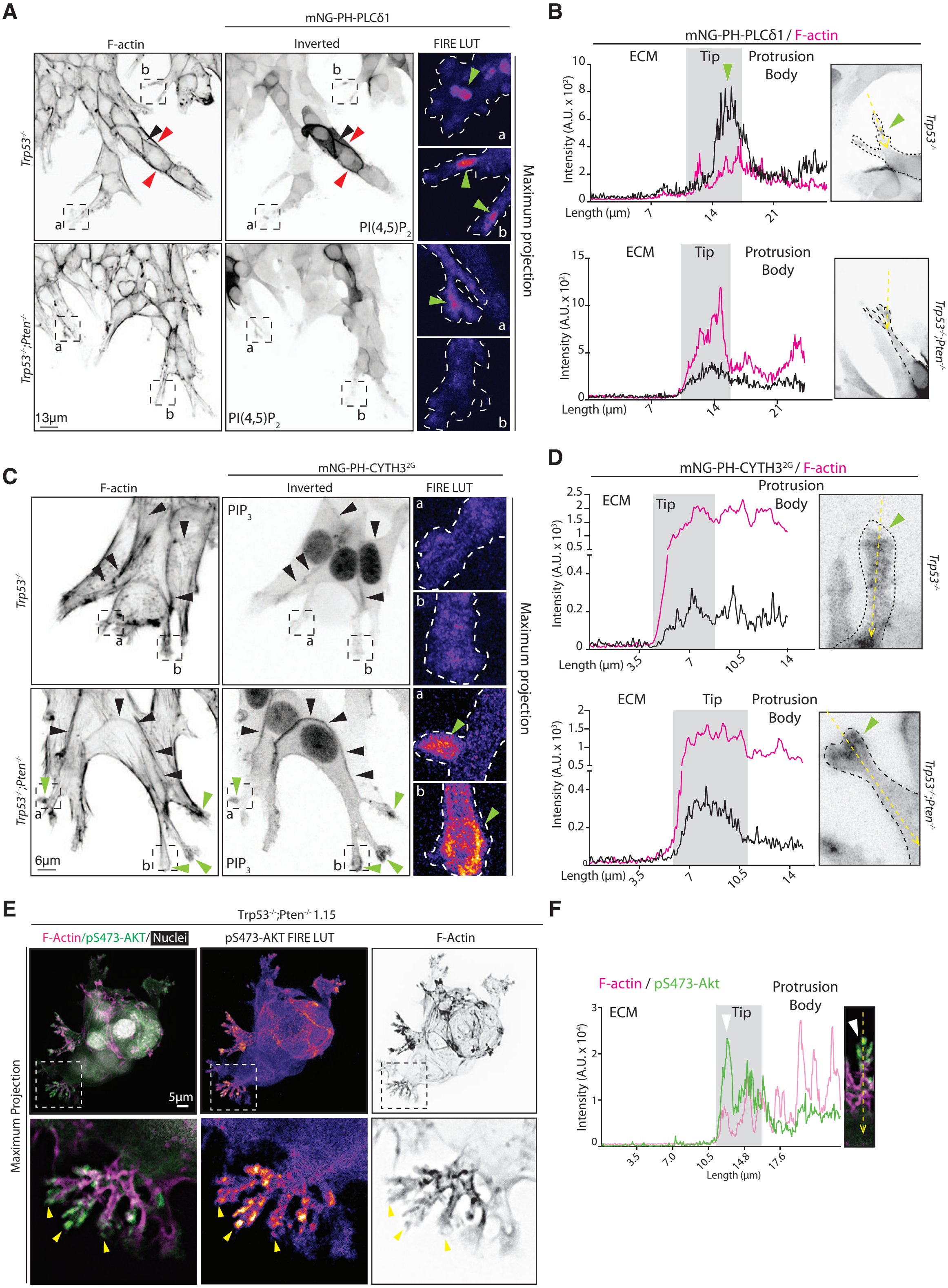
A
mNG-PH-PLCδ1
F-actin
Inverted
FIRE LUT
Trp53-/-
Trp53-/-;Pten-/-
PI(4,5)P2
13µm
Maximum projection
B
mNG-PH-PLCδ1 / F-actin
ECM
Tip
Protrusion Body
Intensity (A.U. x 10²)
Length (µm)
C
mNG-PH-CYTH3 2G
PIP3
6µm
D
mNG-PH-CYTH3 2G / F-actin
Intensity (A.U. x 10³)
E
Trp53-/-;Pten-/- 1.15
F-Actin/pS473-AKT/Nuclei
pS473-AKT FIRE LUT
F-Actin
Maximum Projection
5µm
F
F-actin / pS473-Akt
Intensity (A.U. x 10⁴)
Length (µm)

Figure EV3.

Figure EV3. Characterisation of Phosphoinositide enrichment in Invasion assays.

A, C Confocal images, *Trp53*$^{-/-}$ or *Trp53*$^{-/-}$;*Pten*$^{-/-}$ invasive monolayer fronts with cells expressing mNeonGreen (mNG) tagged biosensors for (A) PI(4,5)P_2 (PH-PLCδ1) or (C) PIP_3 (CYTH3^{2G}). Representative of (A) 7 (*Trp53*$^{-/-}$) or 9 (*Trp53*$^{-/-}$;*Pten*$^{-/-}$) fields or (C) 8 (*Trp53*$^{-/-}$) or 9 (*Trp53*$^{-/-}$;*Pten*$^{-/-}$) fields imaged across n = 2 experiments set up with repeated cultures of each subline. Magnified boxed regions, pseudocoloured with FIRE LUT. Arrowheads: cell–cell contacts, black; protrusions, green; cell-ECM contacts, red. Scale bar, (A)13 μm, (C) 6 μm.

B, D Intensity profiles for mNG PH-PLCδ1 (B) or mNG PH- CYTH3^{2G} (D) from invasive monolayers on (A, C). Tips measured correspond to boxed, magnified regions on images in (A, C). Arrowhead, phosphoinositide-rich region.

E Immunofluorescence and confocal imaging of *Trp53*$^{-/-}$;*Pten*$^{-/-}$ 1.15 spheroid stained for pS473-AKT (green or FIRE LUT), F-actin (magenta or black) and Hoechst (grey). Magnified images from boxed regions. Arrowheads, labelling of pS473-AKT at protrusion tips. Scale bar, 5 μm. Representative of n = 5 spheroids.

F Intensity profile for pS473-AKT (green) and F-Actin (magenta) from spheroid in (A). Tip measured is annotated, ECM to body, yellow arrow; tip, white arrowhead.

Figure EV4. Further characterisation of ARF6 role upon *Trp53* and *Pten* loss.

A, B Western blot (A) and quantitation (B) of pS473-AKT, AKT, ARF5, ARF6, GAPDH in ID8 *Trp53*$^{-/-}$;*Pten*$^{-/-}$ 1.15 cell lines expressing shScramble, sh*Arf5* or sh*Arf6*. Representative blots of n = 3 independent lysate preparations. (B) Data, mean ± SD for ARF5, ARF6 and pS473-AKT band intensity ratio, normalised to shScramble. *P*-values, unpaired, two-tailed *t*-test; ns, not significant. GAPDH is loading control for all panels.

C Representative images, ID8 *Trp53*$^{-/-}$;*Pten*$^{-/-}$ 1.15 cell lines expressing sh*Scramble*, sh*Arf5* or sh*Arf6* in wounded monolayers invading ECM. Yellow lines, initial wound. Arrowheads, invasive protrusions. Outlines of invasive front pseudocoloured by time and overlaid as concatenate over phase image of initial wound. Scale bar, 45 μm. N = 3 independent experiments, 3–6 technical replicates/experiment.

D Quantitation of (C). Graph, Relative Wound Density (RWD) at $t_{1/2}$ max (time when shScramble 50% closed). Data, mean (black square) ± SD for 3 independent experiments (large circles), 3–6 technical replicates//experiment (small circles). *P*-values, ANOVA with Tukey's HSD test; annotated when significant.

E, F Western blot (E) and quantitation (F) from pS473-AKT, AKT, ARF6, VCL in ID8 *Trp53*$^{-/-}$;*Pten*$^{-/-}$ 1.15 cell lines expressing shScramble or sh*Arf6* (5 individual shRNA sequences). Representative blots of n = 3 (ARF6) or n = 2 (pS473-AKT and AKT) independent lysate preparations VCL is loading control for all panels. (F) Data, mean ± SD for ARF6 and pS473-AKT band intensity normalised to shScramble. *P*-values, unpaired, two-tailed *t*-tests; ns, not significant.

G Regression analysis. Scatter plot, mean Hyper-protrusive level across all time points versus ARF6 protein levels (determined by western blot). Solid black line, best linear fit and dotted cyan lines, 95% confidence interval. *P*-value and R^2, annotated.

H Heatmap, Log_2-transformed RNA-sequencing read counts of each ARF GTPase in ID8 spheroids and 2D monolayers (Wild-Type, WT [2D]) across n = 4 independent RNA preparations.

I Western blot and quantitation for ARF6 protein in ID8 sublines. VCL, loading control. Representative blots of n = 3 independent protein isolations. Quantitation, mean ± SD ARF6 intensity normalised to ID8 WT. *P*-values, unpaired, two-tailed *t*-tests; ns, not significant.

J ARF6-GTP levels in ID8 sublines. Normalised Optical Density (OD) of Arf6-GTP G-LISA. N = 3 independent lysate preparations, 3 technical replicates/experiment. Data, mean ± SD of independent replicates (large circles) with technical replicates shown (small circles). *P*-values annotated, student's *t*-test; ns, nonsignificant.

K Western blot, ARF6, T2A, V5, BFP and VCL from lysates extracted from TurboID (control) or ARF6-TurboID-expressing cell lines. VCL, loading control for T2A and BFP and sample integrity control for all other blots. N = 3 independent lysate preparations.

L Confocal images, ID8 *Trp53*$^{-/-}$;*Pten*$^{-/-}$ 1.15 cells expressing ARF6-TurboID or TurboID, stained with T2A. Red box, cell–cell contacts, shown in higher magnification and pseudocoloured with FIRE LUT. Black arrowheads, cell–cell contact; green arrowheads, cell periphery. Scale, 10 μm. Representative images from two fields (ARF6-TurboID) or three fields (TurboID alone) from one experiment.

M Western blot with Streptavidin HRP in ID8 *Trp53*$^{-/-}$;*Pten*$^{-/-}$ 1.15 cells treated with Biotin for at times and concentrations indicated. VCL was used as loading control. n = 1 lysate preparation.

N Gene Ontology Cell Compartment (GOCC) enrichment analysis of interactors identified in ID8 *Trp53*$^{-/-}$;*Pten*$^{-/-}$ 1.15 cells expressing ARF6-TurboID compared to TurboID alone. Data, *P*-value ($-Log_{10}$) of enrichment. N = 4 independent experiments. Red dotted line, significance threshold.

 © 2023 The Authors

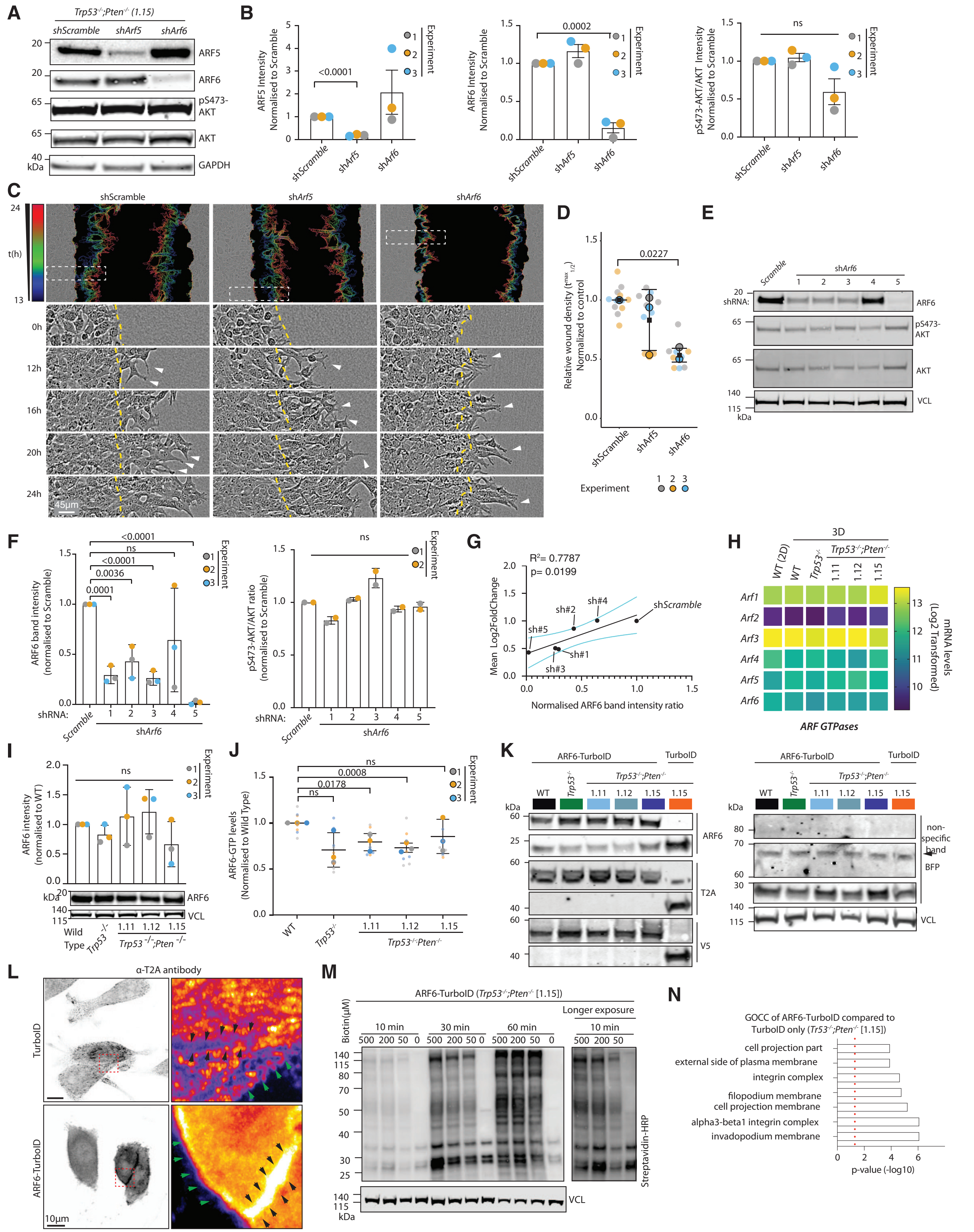
A
B
C
D
E
F
G
H
I
J
K
L
M
N
shScramble
shArf5
shArf6
ARF5
ARF6
pS473-AKT
AKT
GAPDH
VCL
Experiment
<0.0001
0.0002
ns
0.0227
Relative wound density
Normalized to control
R²= 0.7787
p= 0.0199
Normalised ARF6 band intensity ratio
Mean Log2FoldChange
ARF GTPases
mRNA levels (Log2 Transformed)
Arf1
Arf2
Arf3
Arf4
Arf5
Arf6
ARF6-TurboID
TurboID
α-T2A antibody
10µm
45µm
Streptavidin-HRP
Longer exposure
GOCC of ARF6-TurboID compared to TurboID only
cell projection part
external side of plasma membrane
integrin complex
filopodium membrane
cell projection membrane
alpha3-beta1 integrin complex
invadopodium membrane
p-value (-log10)

Figure EV4.

Figure EV5. Characterisation of Cytohesin and CYTH2 contribution to invasion.

A Heatmap, log_2-transformed RNA-sequencing read counts of ARF GEFs in ID8 spheroids and 2D monolayers (Wild Type, WT [2D]) across $n = 4$ independent RNA preparations.

B, C Quantitation of ID8 *Trp53*$^{-/-}$;*Pten*$^{-/-}$ spheroids treated with 20 μM SecinH3, 6-h time intervals over 72 h. (B) Heatmap (viridis)—area presented as mean of *Z*-score values, normalised to control (DMSO). (C) Frequency of Spherical and Hyper-protrusive phenotypes. Heatmap (grayscale)—phenotype proportion (*z*-score) in control. Heatmap (blue-red)—log_2 fold change from control. *P*-values, bubble size (Cochran–Mantel–Haenszel test with Bonferroni adjustment). Black dot, homogenous effect across independent experiments (Breslow–Day test, Bonferroni adjustment, non-significant). $N = 3$ independent experiments, 4–5 technical replicates/experiment. Total spheroid number per condition, Table EV1.

D Representative phase contrast images of spheroids described in (B, C). Outlines pseudocoloured for classification (Spherical, green; Hyper-protrusive, blue). Magnified individual spheroids from boxed regions at indicated timepoints. Arrowheads, protrusions into ECM. Scale bars, 400 or 17 μm, as indicated.

E Representative images of ID8 Trp53$^{-/-}$;Pten$^{-/-}$ 1.15 wounded monolayers treated with DMSO or SecinH3 (20 μM) in wounded monolayers invading ECM. Yellow lines, initial wound. Arrowheads, invasive protrusions. Outlines of invasive front pseudocoloured by time and overlaid as concatenate over phase image of initial wound. $N = 3$ independent experiments, 3–5 technical replicates/experiment. Scale bar, 200 or 45 μm.

F Quantitation of (E). Graph, Relative Wound Density (RWD) at $t_{1/2}$ max (time when DMSO 50% closed). Data, mean (black square) ± SD for 3 independent experiments (large circles), 3–6 technical replicates/experiment (small circles). Exact *P*-value annotated, ANOVA with Tukey's HSD test.

G Spider plots of leader cell movement in the first 19 h of invasion of *Trp53*$^{-/-}$;*Pten*$^{-/-}$ 1.15 ID8 cells treated with DMSO or SecinH3. 10–25 leader cells were tracked per experiment ($n = 2$ set-up using repeated cultures of each subline), across multiple technical replicates/experiment. Representative plots from cells tracked in one independent experiment shown.

H–P *CYTH2* mRNA levels in (H, I) LCM normal ovarian surface epithelium versus HGSOC epithelium or normal ovarian stroma versus ovarian cancer-associated stroma, or (J–P) bulk sequencing of normal ovary versus tumour. Specific data set, sample size (*n*) and *P*-values (Mann–Whitney) annotated, whiskers Min-Max, line at median.

Q–T Overall survival (% patients, months; TCGA OV data set), of patients grouped by low (M1) versus high (M2) levels based on a median split of (Q) *CYTH2* mRNA, (R) *CYTH2* exon 9.1 percentage spliced in ratio (PSI), (S) combination of *ARF6* and *CYTH2* mRNA, or (T) combination of A*RF6* mRNA and *CYTH2* Ex9.1 PSI. Median survival, sample size (*n*) and *P*-value, Log-rank test (Mantel-Cox) annotated.

 © 2023 The Authors

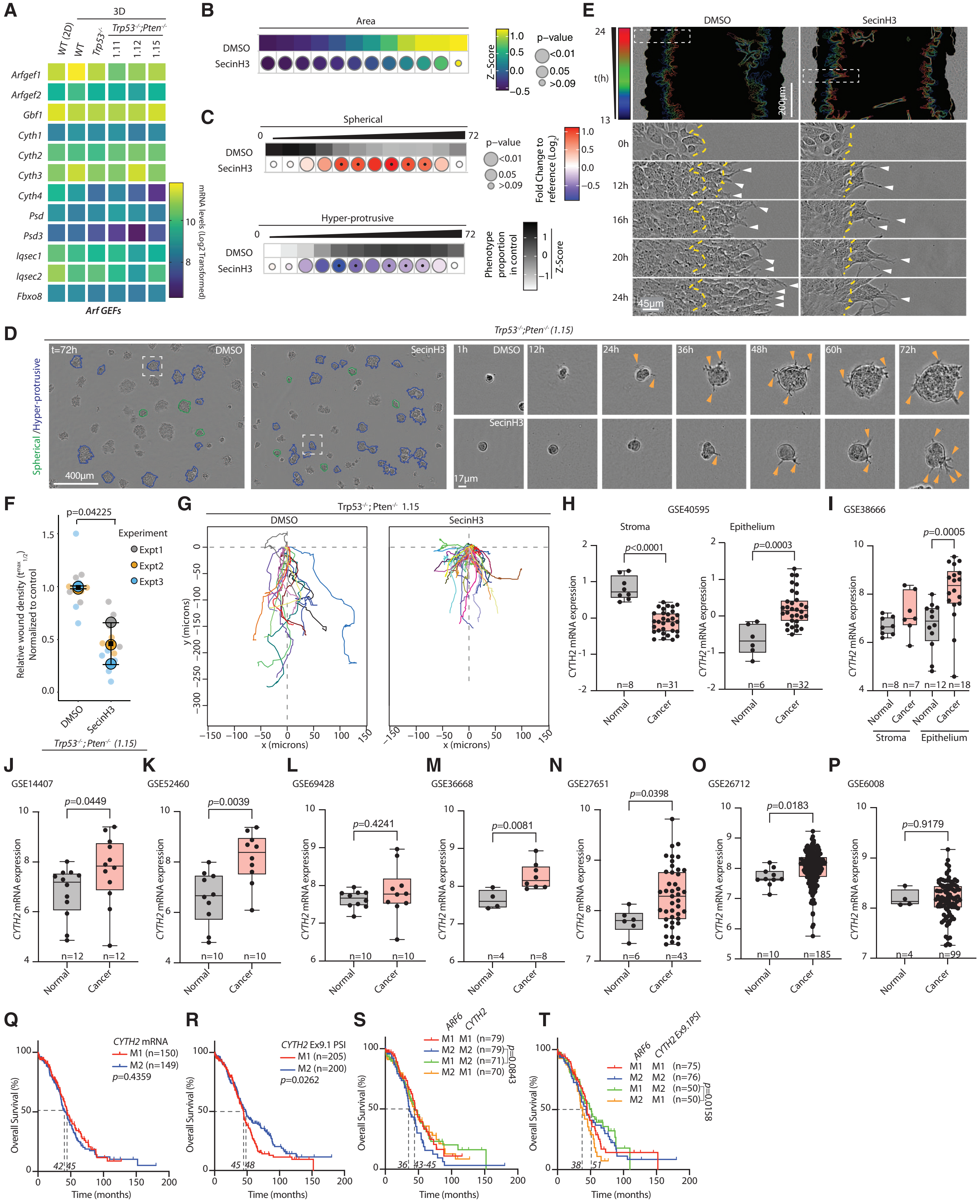

Figure EV5.

PTEN deficiency exposes a requirement for an ARF GTPase module for integrin-dependent invasion in ovarian cancer.

Appendix

Table of Contents

Appendix Figure S1 2
Appendix Figure S2 5
Appendix Figure S3 8

Appendix Figure S1

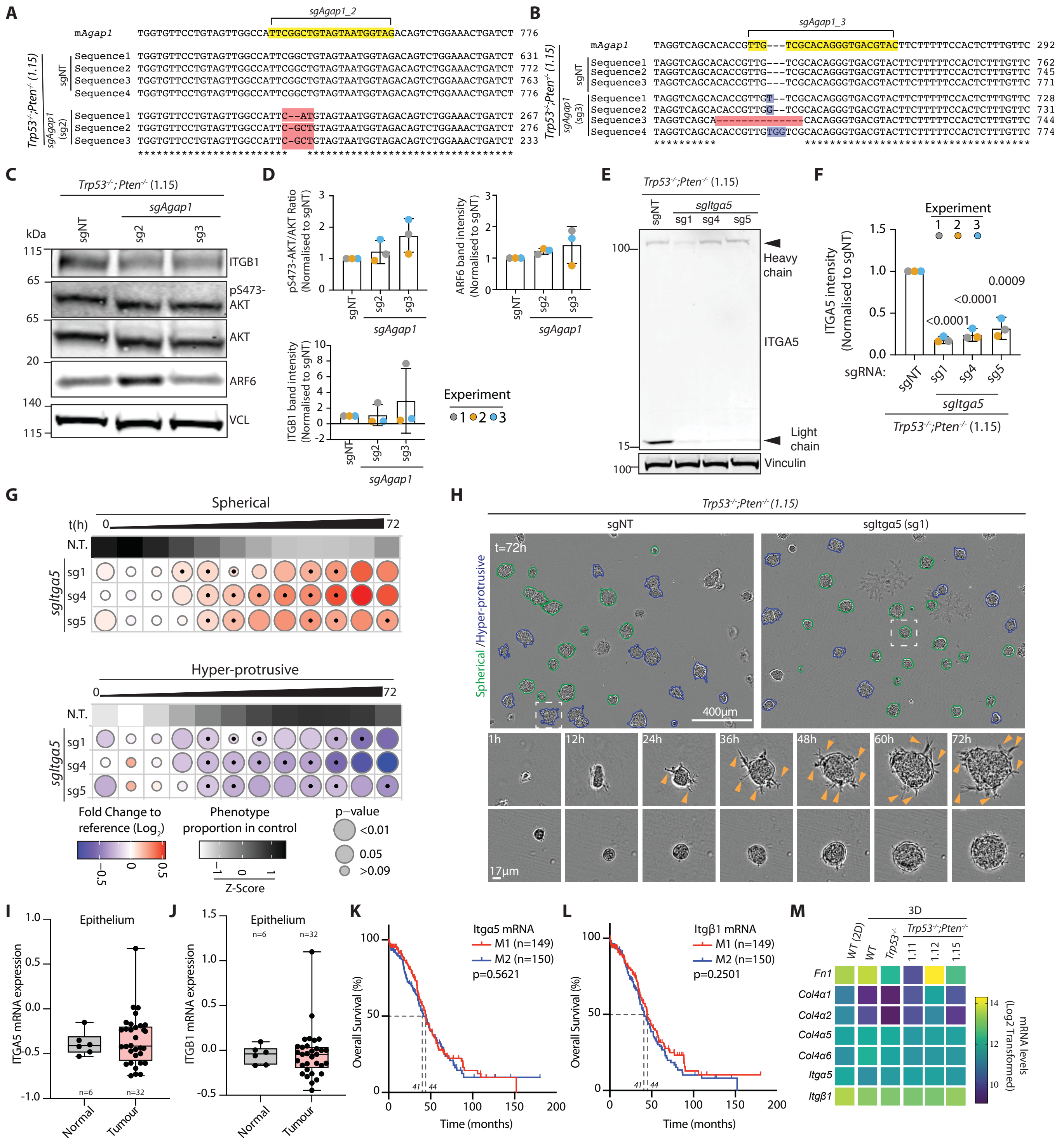

Appendix Figure S1. Itga5 contribution to collective invasion.

A-B. Sequencing of CRISPR editing of the murine *Agap1* locus with sg2 **(A)** or sg3 **(B)** compared to control (sgNT). Yellow, sgRNA sequence; red, deletions; blue, insertions.

C-D. Western blotting **(C)** and quantitation **(D)** of β1-integrin (ITGB1), pS473-AKT, AKT and ARF6 in lysates from *Agap1* KO cells (sgNT versus sg2 or sg3). VCL is loading control for AKT, pS473-AKT and ARF6 and sample integrity control for ITGB1. Representative blots of n=3 independent lysate preparations. **(D)** Data, mean ± SD for pS473-AKT:total AKT band intensity ratio, normalised to control (sgNT). Unpaired, two-tailed t-test, p values not significant (>0.05).

E-F. Western blot **(E)** and quantitation (**F**), α5 integrin (ITGA5) in *ITGA5* KO cells (sg1, sg4, sg5 versus sgNT). VCL, loading control. Representative blots of n=3 independent lysate preparations. **(F)** Data, mean ± SD sum intensity of ITGA5 heavy and light chain bands, normalised to ID8 $Trp53^{-/-}$;$Pten^{-/-}$ 1.15 cells treated with sgNT. Unpaired, two-tailed t-test, p values are annotated.

G. Frequency of Spherical and Hyper-protrusive phenotypes upon CRISPR-mediated KO of *Itga5,* 6 hr time intervals over 72 hrs. Heatmap (grayscale) - phenotype proportion (z-score) in control (sgNT). Heatmap (blue-red) - $\log_2$ fold change from control. P-values, bubble size (Cochran-Mantel-Haenszel test with Bonferroni adjustment). Black dot, homogenous effect across independent experiments (Breslow-Day test, Bonferroni adjustment, non-significant). N=3 independent experiments, 3-6 technical replicates/experiment. Total spheroid number per condition, Table EV 1.

H. Representative phase contrast images of spheroids described in **(G)**. Outlines pseudocoloured for classification (Spherical, green; Hyper-protrusive, blue) at indicated timepoints. Magnified individual spheroids from boxed regions. Arrowheads, protrusions into ECM. Scale bars, 400μm or 17μm, as indicated.

I-J. *Itgα5* **(I)** and *Itgβ1* **(J)** mRNA levels in LCM normal ovarian surface epithelium versus HGSOC epithelium. Dataset, GSE40595, sample size (n) and p-values (Mann-Whitney) annotated, whiskers Min-Max, line at median.

K-L. Overall survival (% patients, months; TCGA OV dataset), of patients grouped by low (M1) versus high (M2) mRNA levels, based on a median split, of **(K)** *ITGA5* or **(L)** *ITGB1*. Median survival, sample size (n) and p-value, Log-rank test (Mantel-Cox) annotated.

M. Heatmap, Log_2-transformed RNA-sequencing read counts of Collagen IV, Fibronectin *Itgα5* and *Itgβ1* in ID8 spheroids and 2D monolayers (Wild Type, WT (2D)) across n=4 independent RNA preparations.

Appendix Figure S2

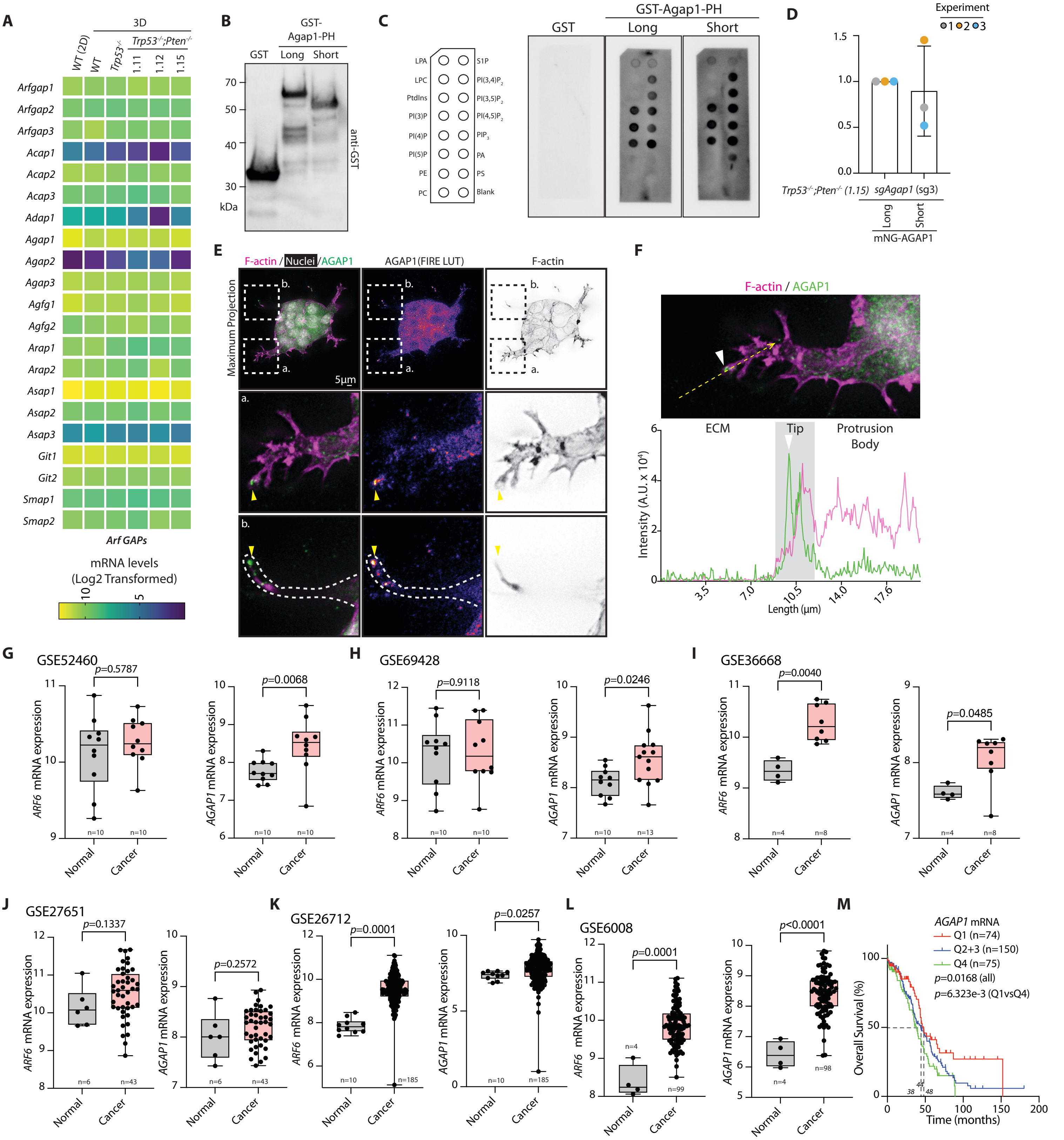

Appendix Figure S2. Further characterization of AGAP1 biological and clinical features.

A. Heatmap, log_2-transformed RNA-sequencing read counts of ARF GAPs in ID8 spheroids and 2D monolayers (Wild Type, WT (2D)) across n=4 independent RNA preparations.

B. Western blot for GST performed on purified recombinant GST alone, or GST-fusion of both isoforms of AGAP1 PH domains. Representative of n=2 blots performed.

C. Binding of the GST (Control), GST-AGAP1 PH Long or GST-AGAP1 PH Short fusion proteins to phospholipids immobilised on a cellulose membrane (cartoon on left denotes lipid position. Probed using a-GST antibody for visualization. Representative of n=3 independent experiments. LPA, Lysophosphatidic acid; LPC, Lysophosphocholine; PE, Phosphatidylethanolamine; PC, Phosphatidylcholine; S1P, Sphingosine-1-phosphate; PA, Phosphatidic Acid; PS, Phosphatidylserine.

D. Quantitation of **(Figure 6C).** Data, mean ± SD of mNG band intensity between mNG-AGAP1-Long and mNG-AGAP1-Short isoforms. Unpaired, two-tailed t-test, p values ns, non-significant (>0.05).

E. Immunofluorescence and confocal imaging of *Trp53*$^{-/-}$;*Pten*$^{-/-}$ 1.15 spheroids stained for AGAP1 (green) and F-actin (magenta). Magnified images from boxed regions, pseudocoloured in inverted grayscale (F-actin) or FIRE LUT (AGAP1). Arrowheads, labelling at protrusion tips. Scale bars = 5 µm. Representative of n=3 spheroids imaged.

F. Intensity profiles for AGAP1 (green) and F-actin (magenta) from spheroid in **(E)**. Tip measured is annotated, ECM to body, yellow arrow, tip, white arrowhead.

G-L. *ARF6 and AGAP1* mRNA levels in normal ovary versus tumour. Specific datasets, sample size (n) and p-values (Mann-Whitney) annotated, whiskers Min-Max, line at median.

M. Overall survival (% patients, months; TCGA OV dataset), of patients grouped by *AGAP1* mRNA levels based on quartile. Median survival, sample size (n) and p-value, Log-rank test (Mantel-Cox) annotated.

Appendix Figure S3

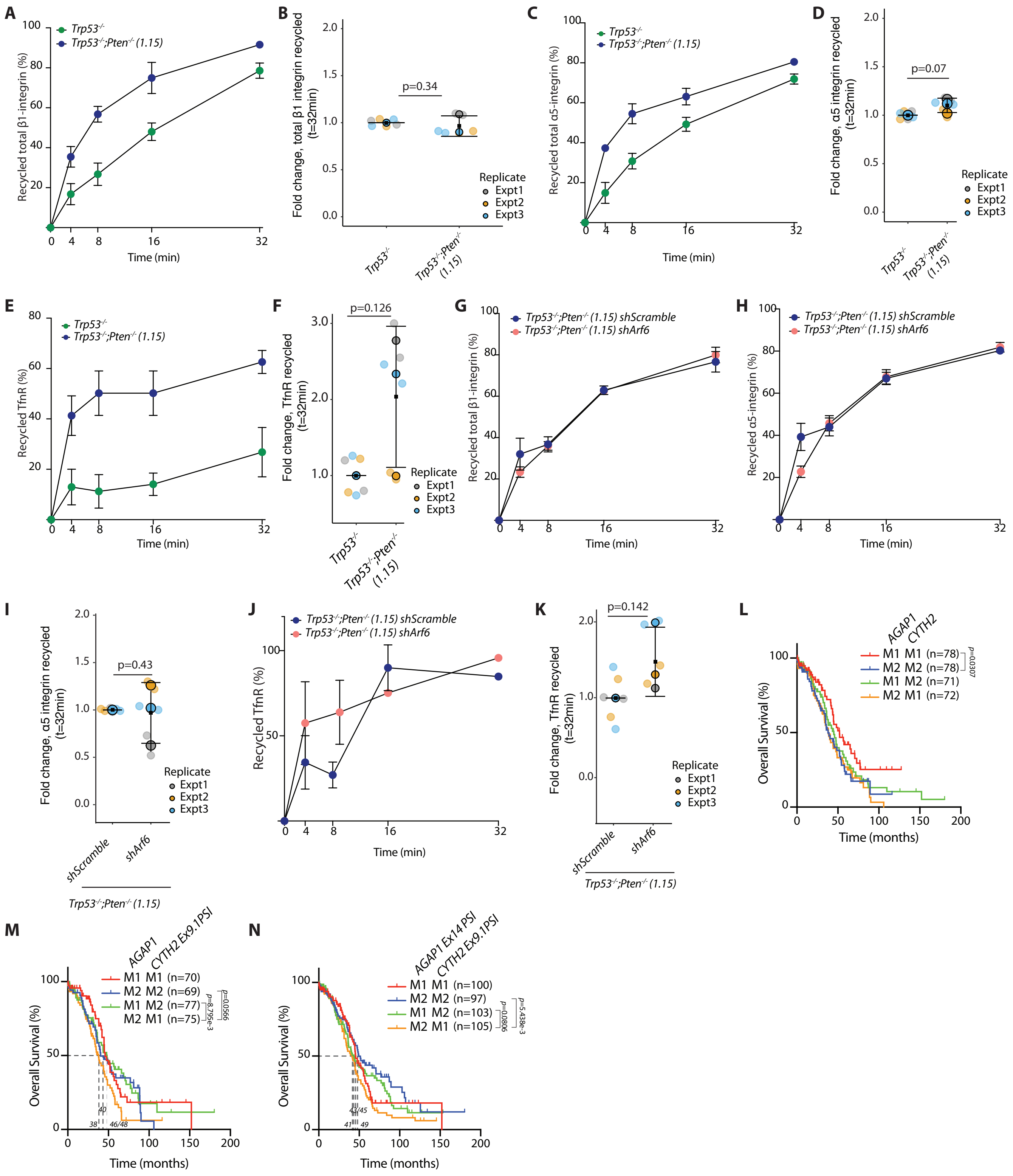

Appendix Figure S3. The ARF6 module does not affect recycling of other cargos.

A-K. Representative capture ELISA graphs (**A,C,E,G,H,J**) and associated quantitation (**B,D,F,I,K**) for recycling of internalized cargoes. *Trp53*$^{-/-}$ versus *Trp53*$^{-/-}$*;Pten*$^{-/-}$ cells (**A-F**) or *Trp53*$^{-/-}$*;Pten*$^{-/-}$ cells expressing sh*Scramble* versus sh*Arf6* (**G-K**). Graphs are shown for total β1 integrin (**A,B,G**), α5 integrin **(C,D,H,I)** or Transferrin receptor (TrnR) **(E,F,J,K)**. Graphs shown are representative of n=3 independent replicates apart from total β1 integrin in *Trp53*$^{-/-}$*;Pten*$^{-/-}$ cells expressing sh*Arf6,* for which n=1. Data, mean (black square) ± SD for 3 repeated experiments (large circles), 1-3 technical replicates/experiment/timepoint (small circles), two-tailed t-test, p-values are annotated.

L-N. Overall survival (% patients, months; TCGA OV dataset) of patients grouped in combinations of a median split of (**L**) *AGAP1* mRNA and *CYTH2* mRNA, (**M**) *AGAP1* mRNA and *CYTH2* Ex9.1 PSI, (**N**) *AGAP1* Ex14 PSI and *CYTH2* Ex9.1 PS. Median survival, sample size (n) and p-value, Log-rank test (Mantel-Cox) annotated.
