# Supplementary figures and images for: PTEN deficiency exposes a requirement for an ARF GTPase module for integrin‐dependent invasion in ovarian cancer (part 1 of 2)
Source: EMBO J. 2023 Aug 14;42(18):e113987. doi: 10.15252/embj.2023113987 (PMC10505920; doi:10.15252/embj.2023113987)

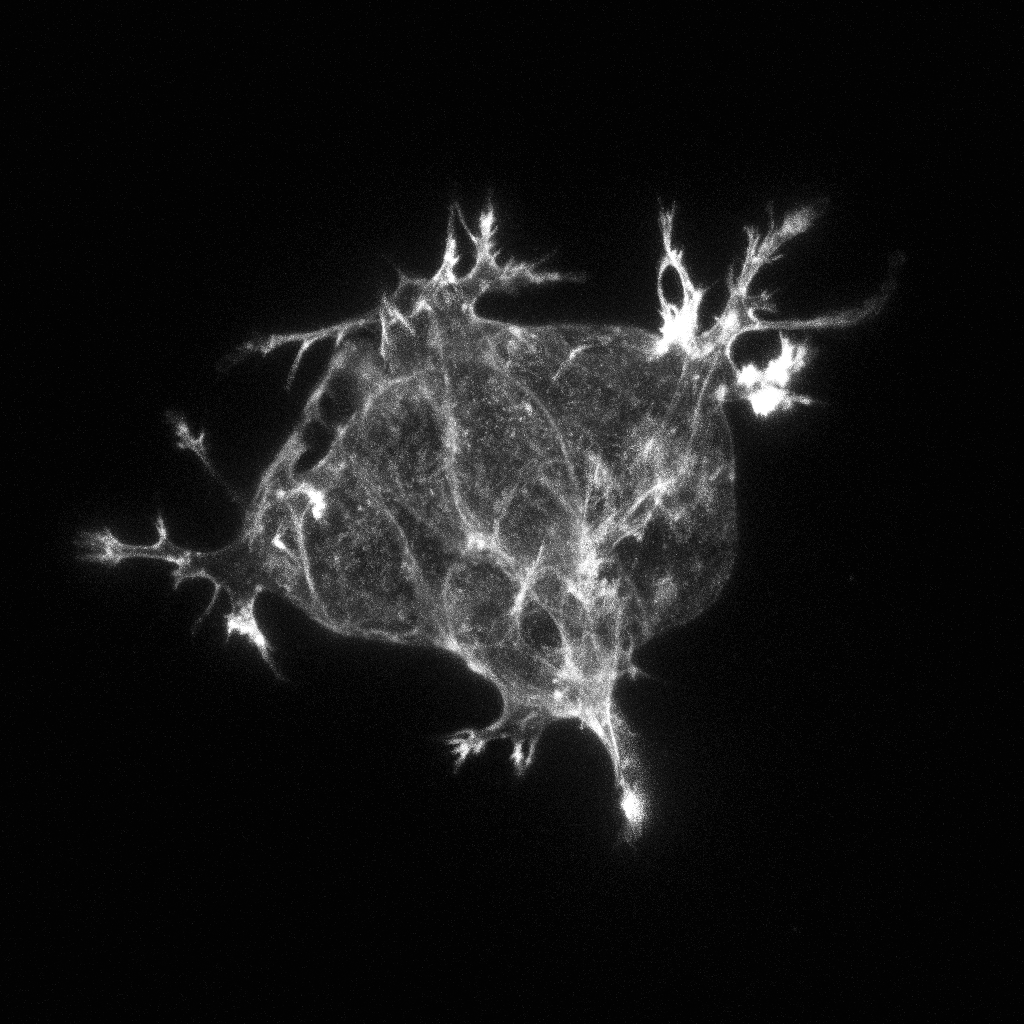

Supplement: Supplementary file 21 — Source Data for Figure 3 [file EMBJ-42-e113987-s002.zip › Figure 3/3K/ACTIN-MAX_1.15 PI3KB 3.tif]

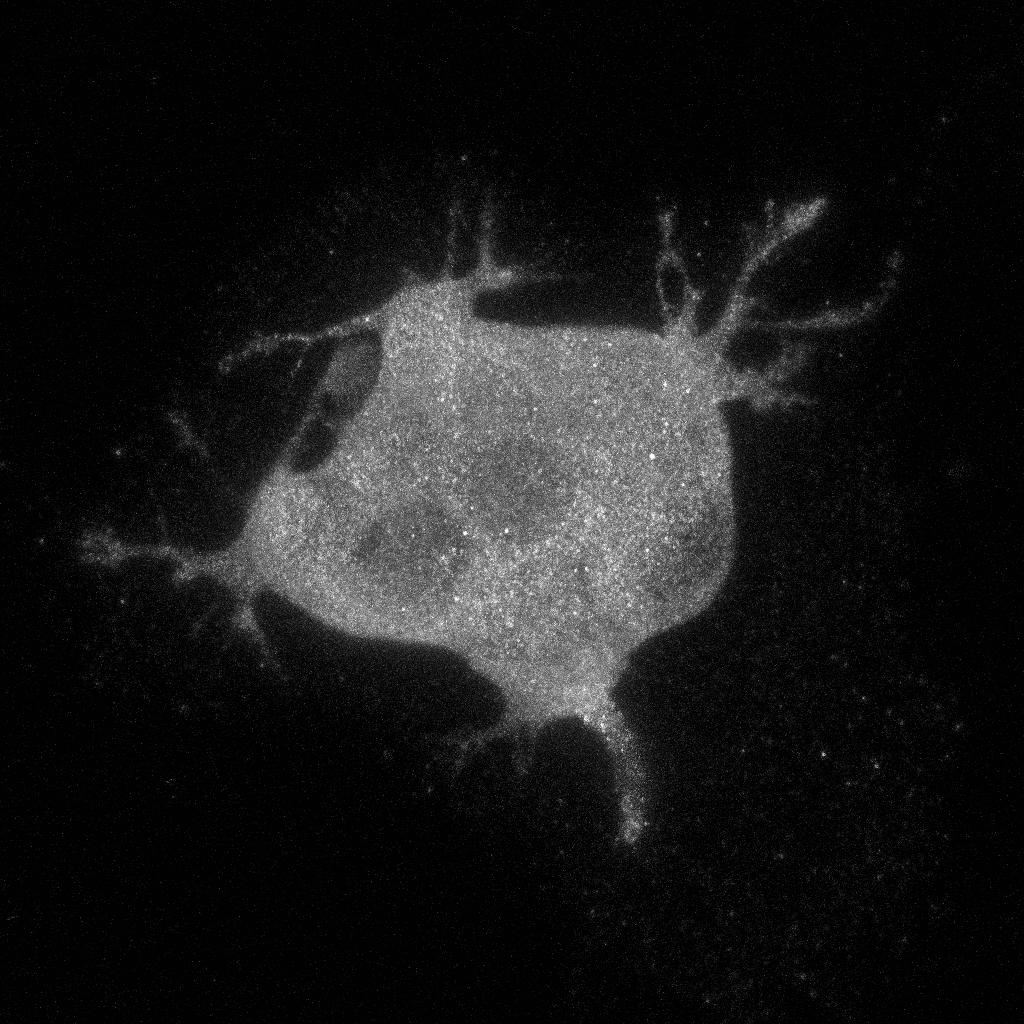

Supplement: Supplementary file 21 — Source Data for Figure 3 [file EMBJ-42-e113987-s002.zip › Figure 3/3K/PI3KB-MAX_1.15 PI3KB 3.tif]

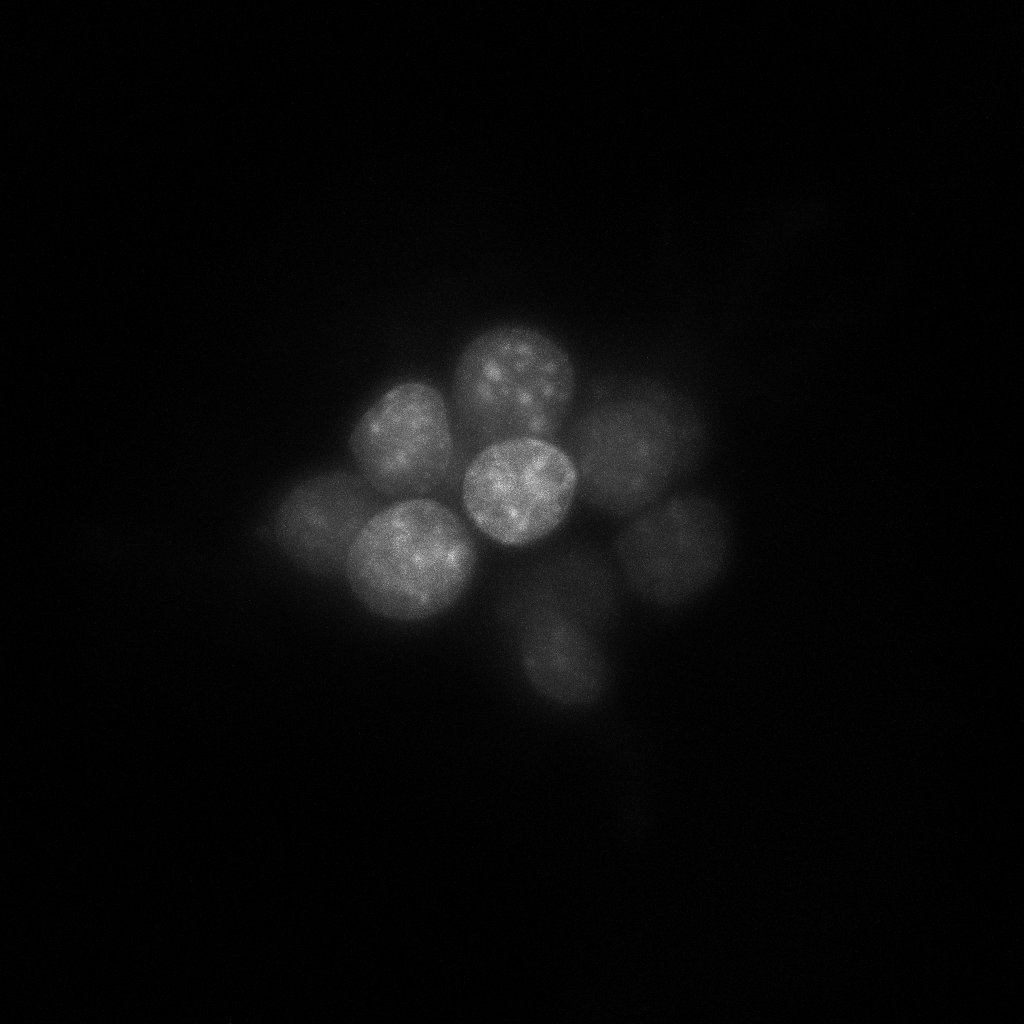

Supplement: Supplementary file 21 — Source Data for Figure 3 [file EMBJ-42-e113987-s002.zip › Figure 3/3K/HOECSHT-MAX_1.15 PI3KB 3.tif]

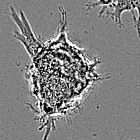

Supplement: Supplementary file 21 — Source Data for Figure 3 [file EMBJ-42-e113987-s002.zip › Figure 3/3J/DMSO t=72h_Full.tif]

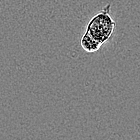

Supplement: Supplementary file 21 — Source Data for Figure 3 [file EMBJ-42-e113987-s002.zip › Figure 3/3J/DMSO t=12h.tif]

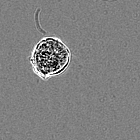

Supplement: Supplementary file 21 — Source Data for Figure 3 [file EMBJ-42-e113987-s002.zip › Figure 3/3J/PI3Kbeta t=60h.tif]

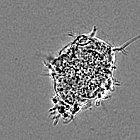

Supplement: Supplementary file 21 — Source Data for Figure 3 [file EMBJ-42-e113987-s002.zip › Figure 3/3J/DMSO t=60h.tif]

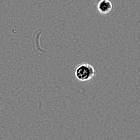

Supplement: Supplementary file 21 — Source Data for Figure 3 [file EMBJ-42-e113987-s002.zip › Figure 3/3J/PI3Kbeta t=1h.tif]

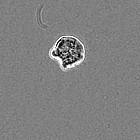

Supplement: Supplementary file 21 — Source Data for Figure 3 [file EMBJ-42-e113987-s002.zip › Figure 3/3J/PI3Kbeta t=36h.tif]

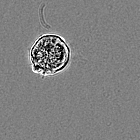

Supplement: Supplementary file 21 — Source Data for Figure 3 [file EMBJ-42-e113987-s002.zip › Figure 3/3J/PI3Kbeta t=72h_Full.tif]

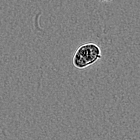

Supplement: Supplementary file 21 — Source Data for Figure 3 [file EMBJ-42-e113987-s002.zip › Figure 3/3J/PI3Kbeta t=12h.tif]

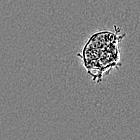

Supplement: Supplementary file 21 — Source Data for Figure 3 [file EMBJ-42-e113987-s002.zip › Figure 3/3J/DMSO t=24h.tif]

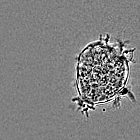

Supplement: Supplementary file 21 — Source Data for Figure 3 [file EMBJ-42-e113987-s002.zip › Figure 3/3J/DMSO t=48.tif]

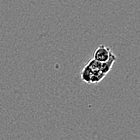

Supplement: Supplementary file 21 — Source Data for Figure 3 [file EMBJ-42-e113987-s002.zip › Figure 3/3J/DMSO t=1h.tif]

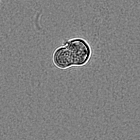

Supplement: Supplementary file 21 — Source Data for Figure 3 [file EMBJ-42-e113987-s002.zip › Figure 3/3J/PI3Kbeta t=24h.tif]

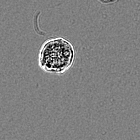

Supplement: Supplementary file 21 — Source Data for Figure 3 [file EMBJ-42-e113987-s002.zip › Figure 3/3J/PI3Kbeta t=48h.tif]

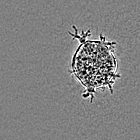

Supplement: Supplementary file 21 — Source Data for Figure 3 [file EMBJ-42-e113987-s002.zip › Figure 3/3J/DMSO t=36.tif]

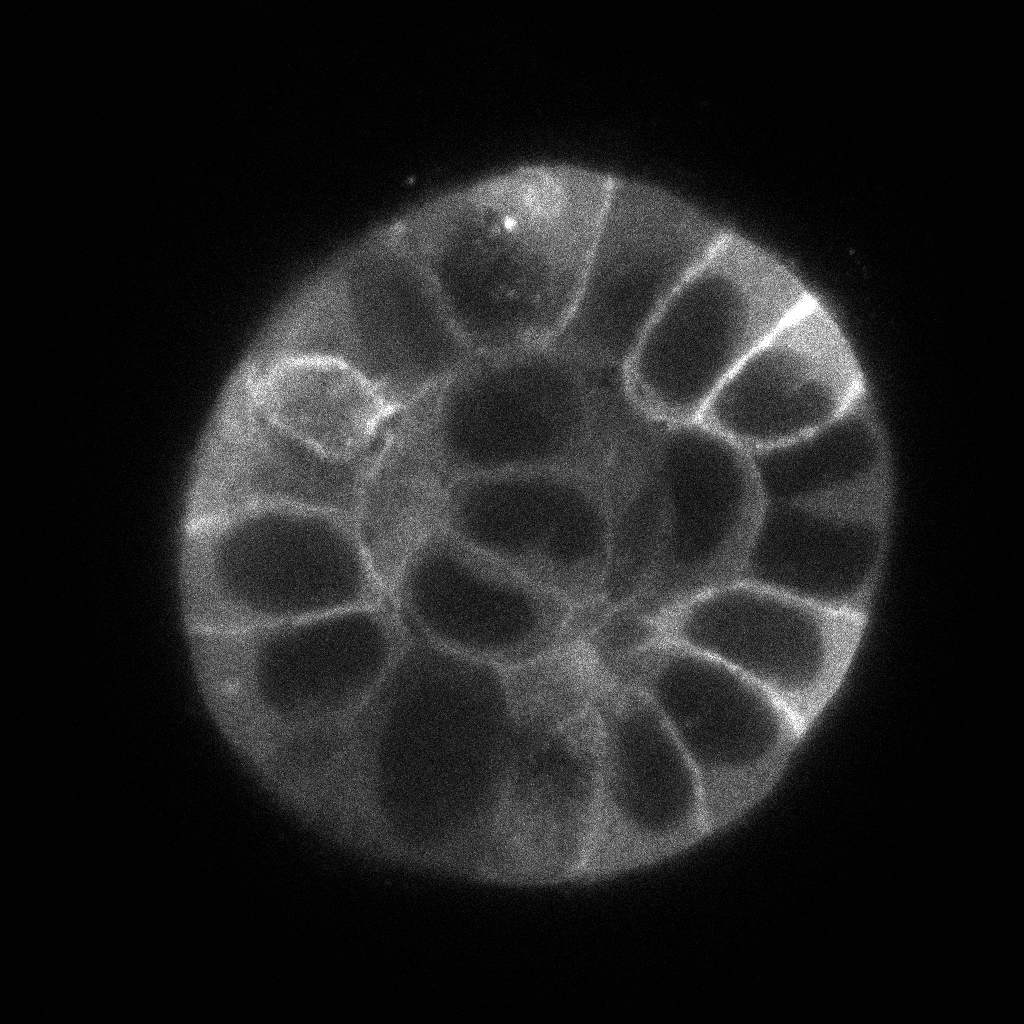

Supplement: Supplementary file 21 — Source Data for Figure 3 [file EMBJ-42-e113987-s002.zip › Figure 3/3A/z=20:34 Trp53-:- mNG PLCdelta1.tif]

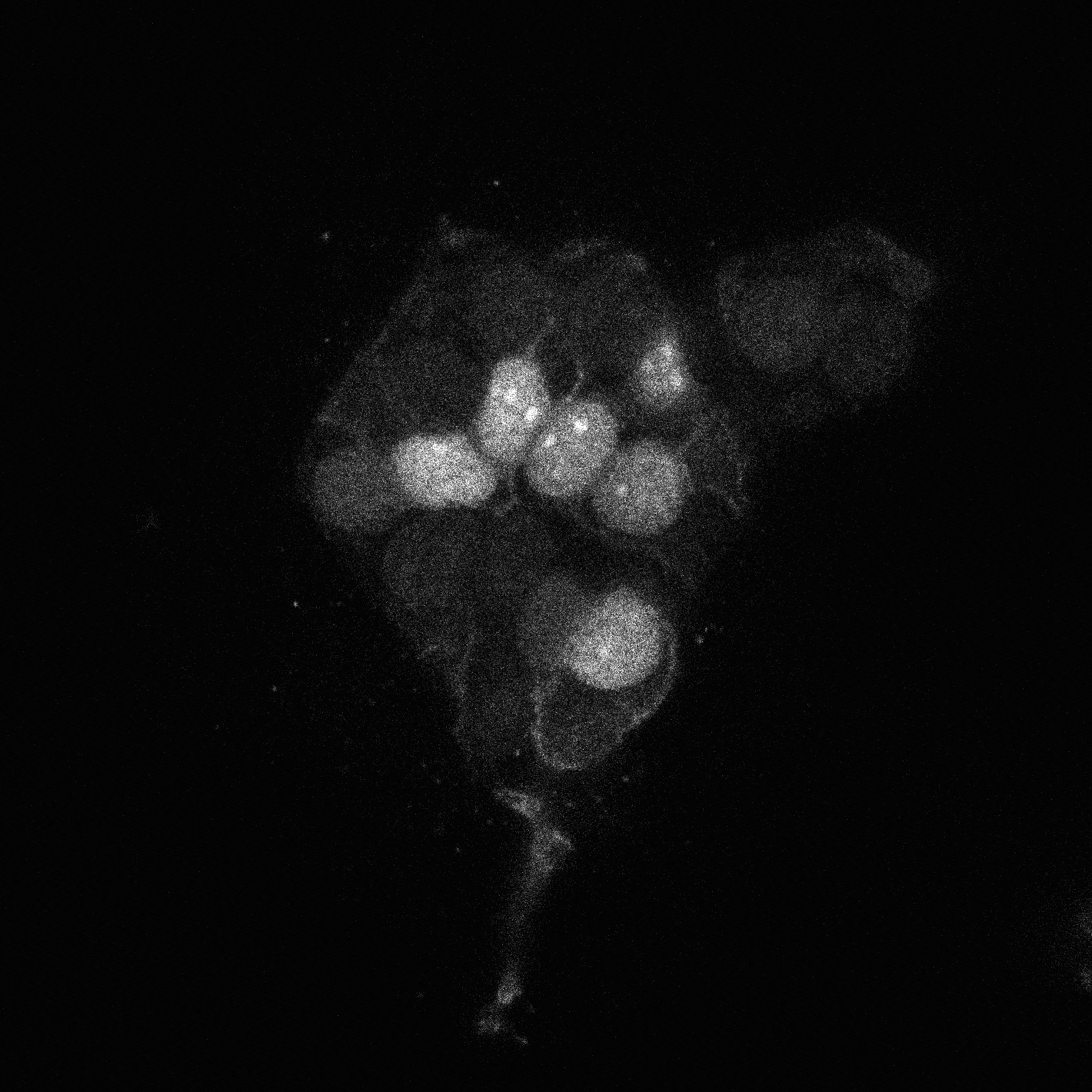

Supplement: Supplementary file 21 — Source Data for Figure 3 [file EMBJ-42-e113987-s002.zip › Figure 3/3A/z=11:52 Trp53-:- Pten-:- mNG PH CYTH3.tif]

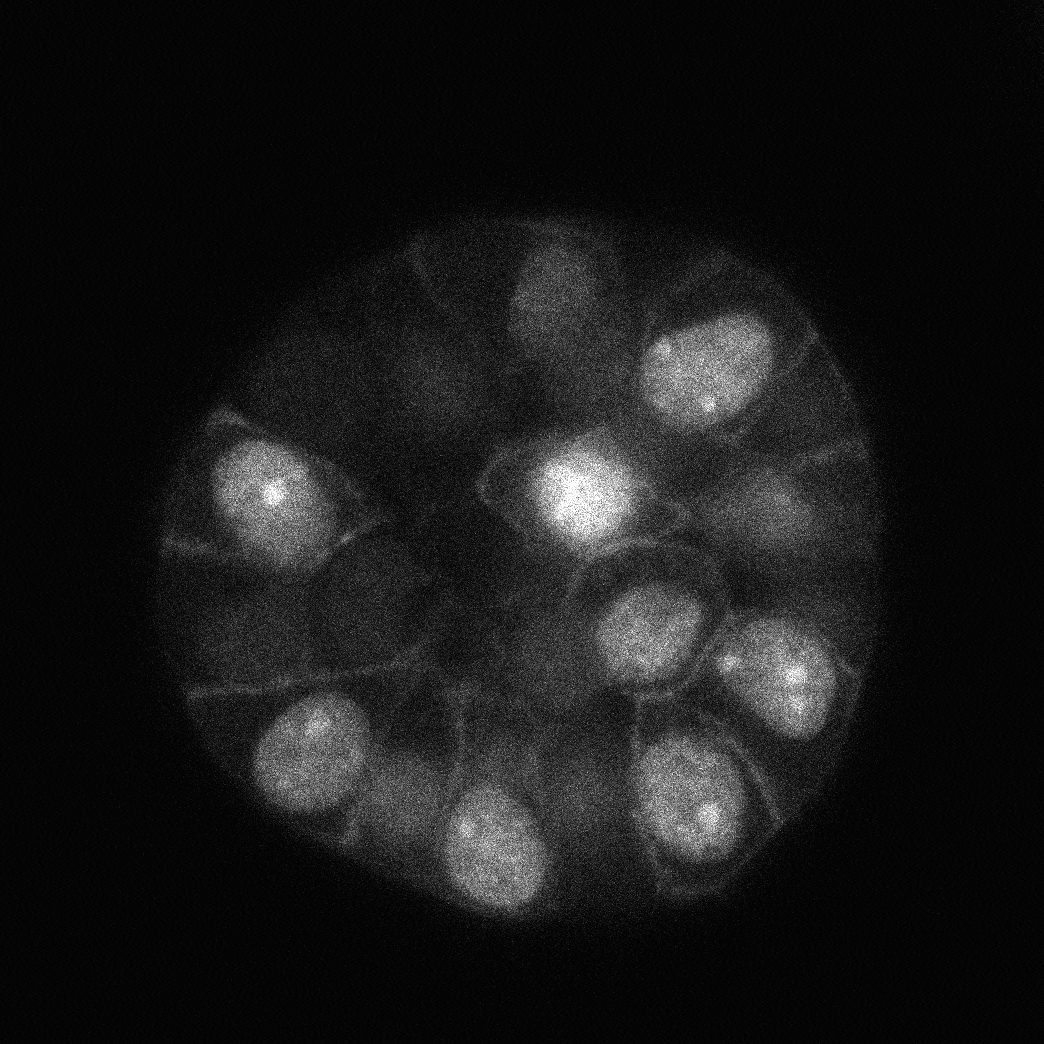

Supplement: Supplementary file 21 — Source Data for Figure 3 [file EMBJ-42-e113987-s002.zip › Figure 3/3A/z=24:35 Trp53-:- mNG PH CYTH3.tif]

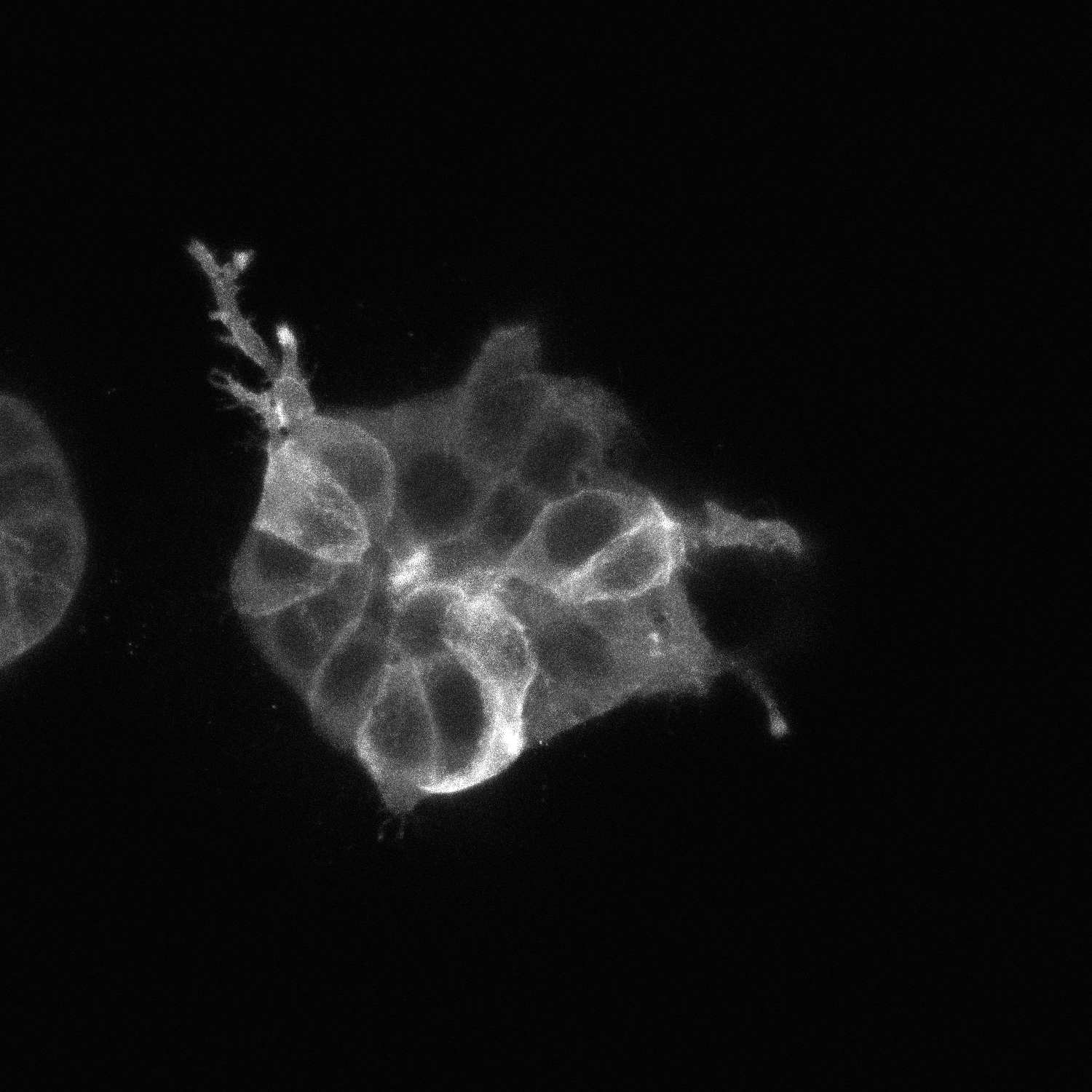

Supplement: Supplementary file 21 — Source Data for Figure 3 [file EMBJ-42-e113987-s002.zip › Figure 3/3A/mNG PLCD1 z=14-17-MAX_Trp53-:- Pten-:-.tif]

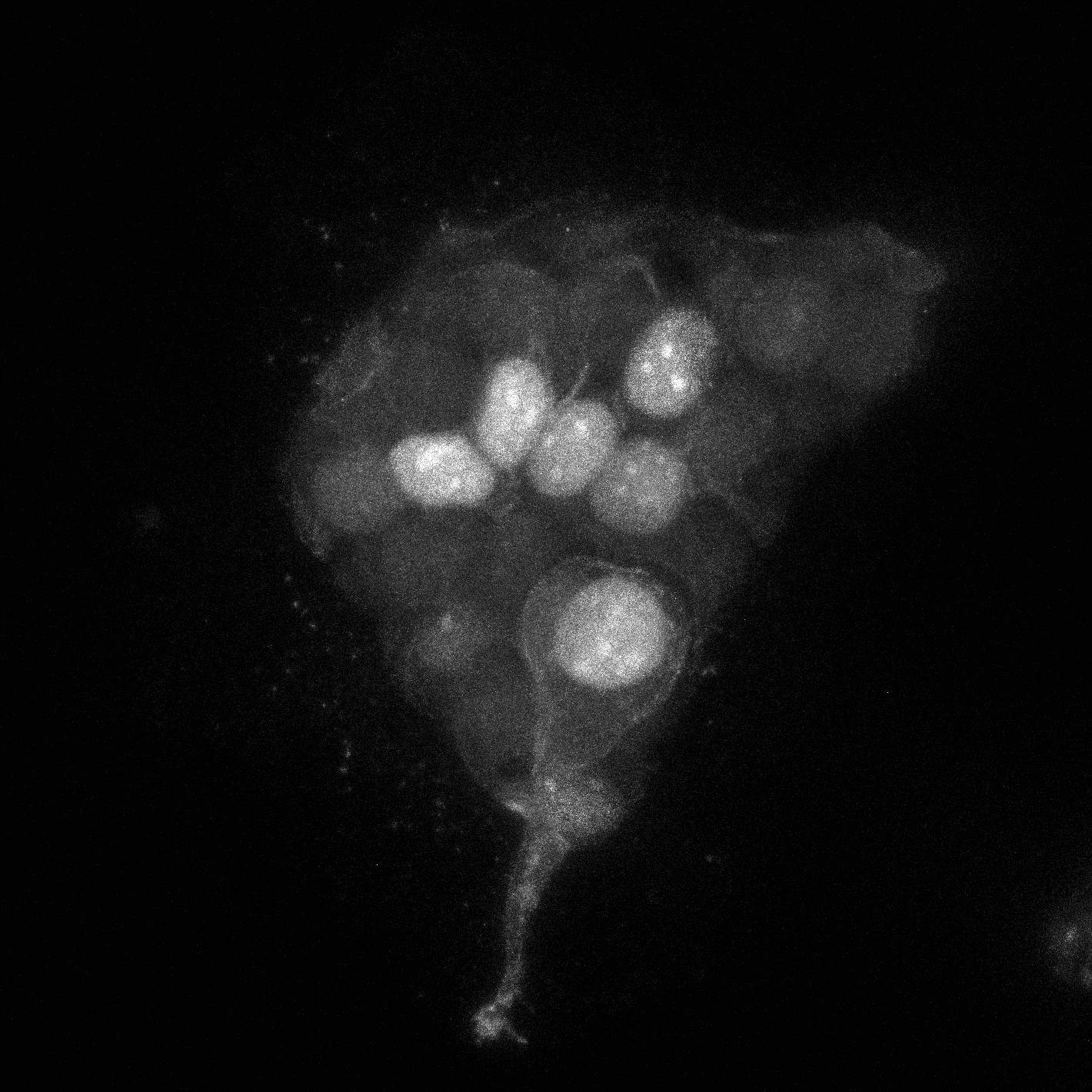

Supplement: Supplementary file 21 — Source Data for Figure 3 [file EMBJ-42-e113987-s002.zip › Figure 3/3A/mNG PH CYTH3 Z=18-16-MAX_ Trp53-:- Pten-:- .tif]

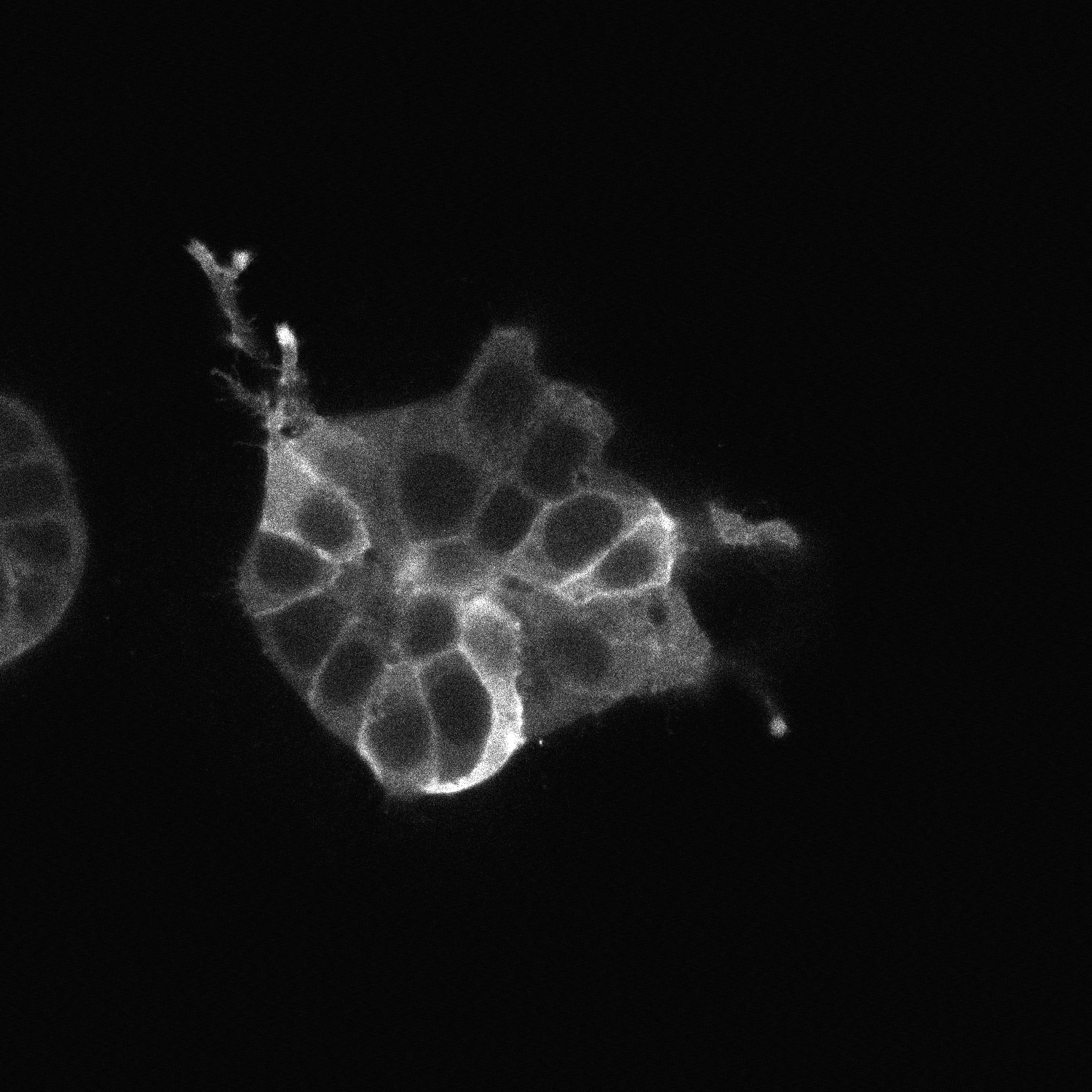

Supplement: Supplementary file 21 — Source Data for Figure 3 [file EMBJ-42-e113987-s002.zip › Figure 3/3A/z=15:34 Trp53-:- Pten-:- mNG PLCdelta1.tif]

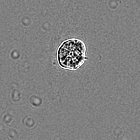

Supplement: Supplementary file 21 — Source Data for Figure 3 [file EMBJ-42-e113987-s002.zip › Figure 3/3G/PI3Ki t=60h.tif]

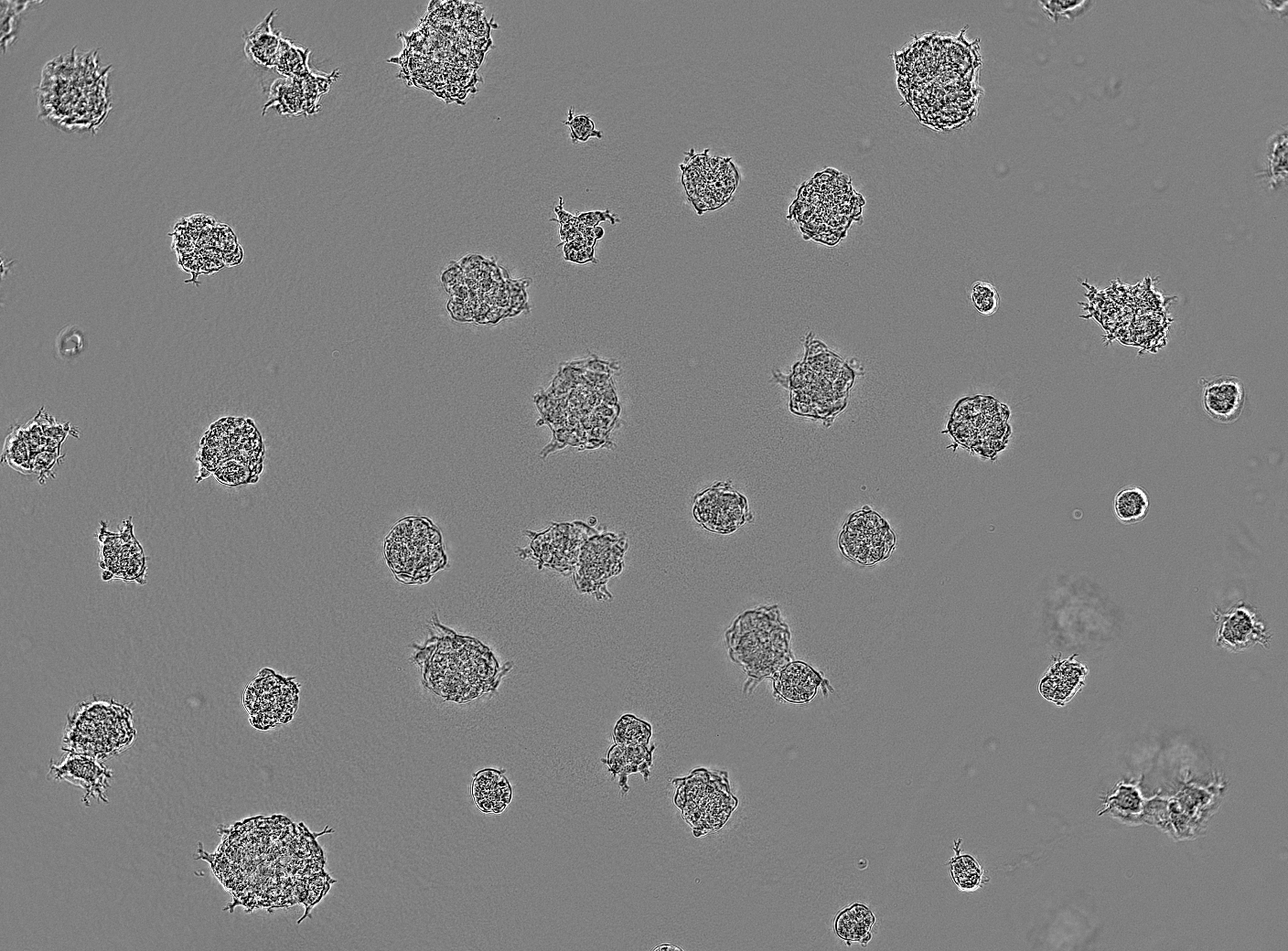

Supplement: Supplementary file 21 — Source Data for Figure 3 [file EMBJ-42-e113987-s002.zip › Figure 3/3G/DMSO t=72h_Full.tif]

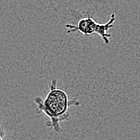

Supplement: Supplementary file 21 — Source Data for Figure 3 [file EMBJ-42-e113987-s002.zip › Figure 3/3G/DMSO t=12h.tif]

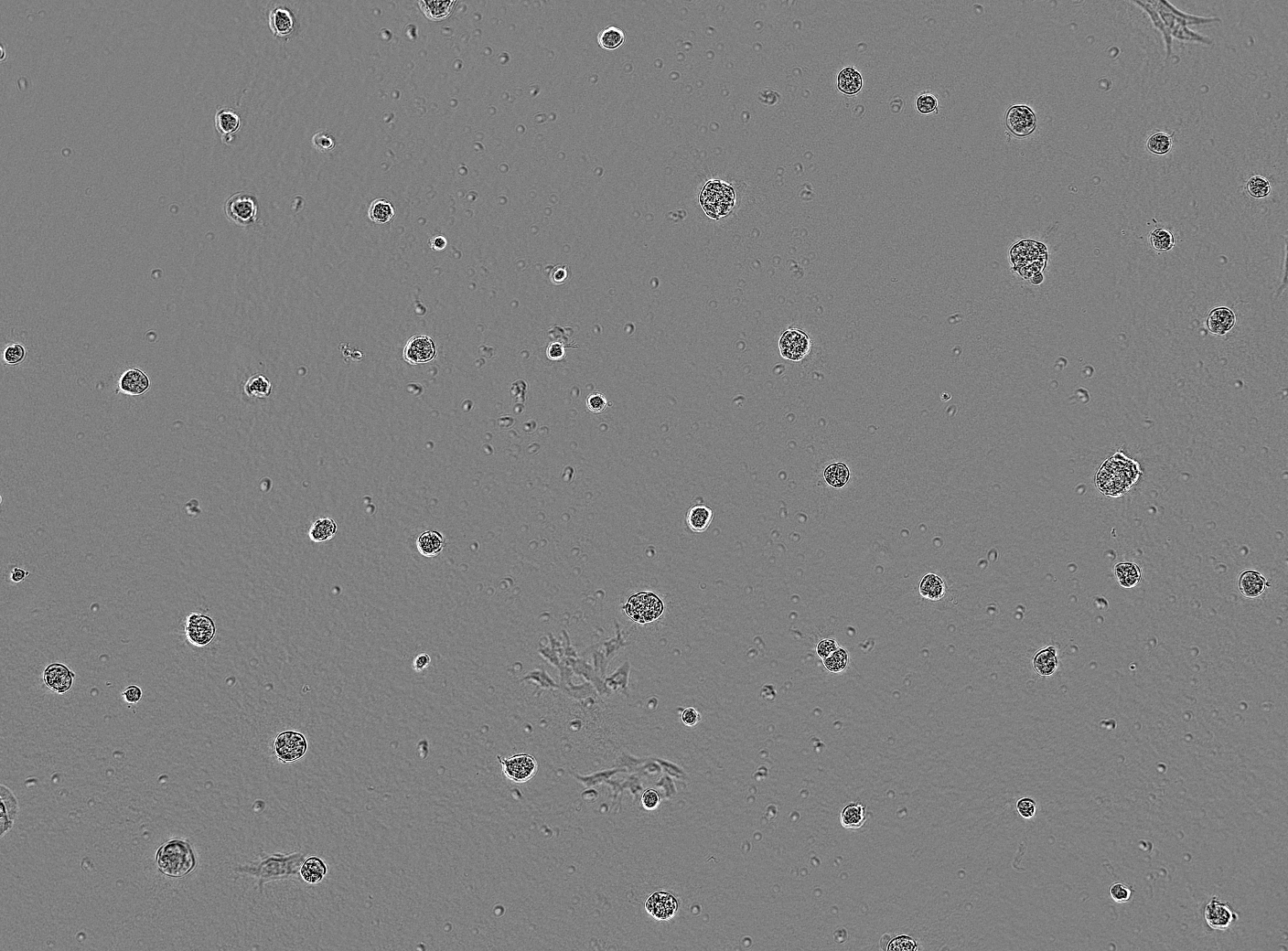

Supplement: Supplementary file 21 — Source Data for Figure 3 [file EMBJ-42-e113987-s002.zip › Figure 3/3G/PI3Ki t=72h_Full.tif]

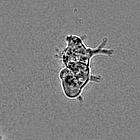

Supplement: Supplementary file 21 — Source Data for Figure 3 [file EMBJ-42-e113987-s002.zip › Figure 3/3G/DMSO t=36h.tif]

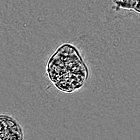

Supplement: Supplementary file 21 — Source Data for Figure 3 [file EMBJ-42-e113987-s002.zip › Figure 3/3G/Akti t=72h.tif]

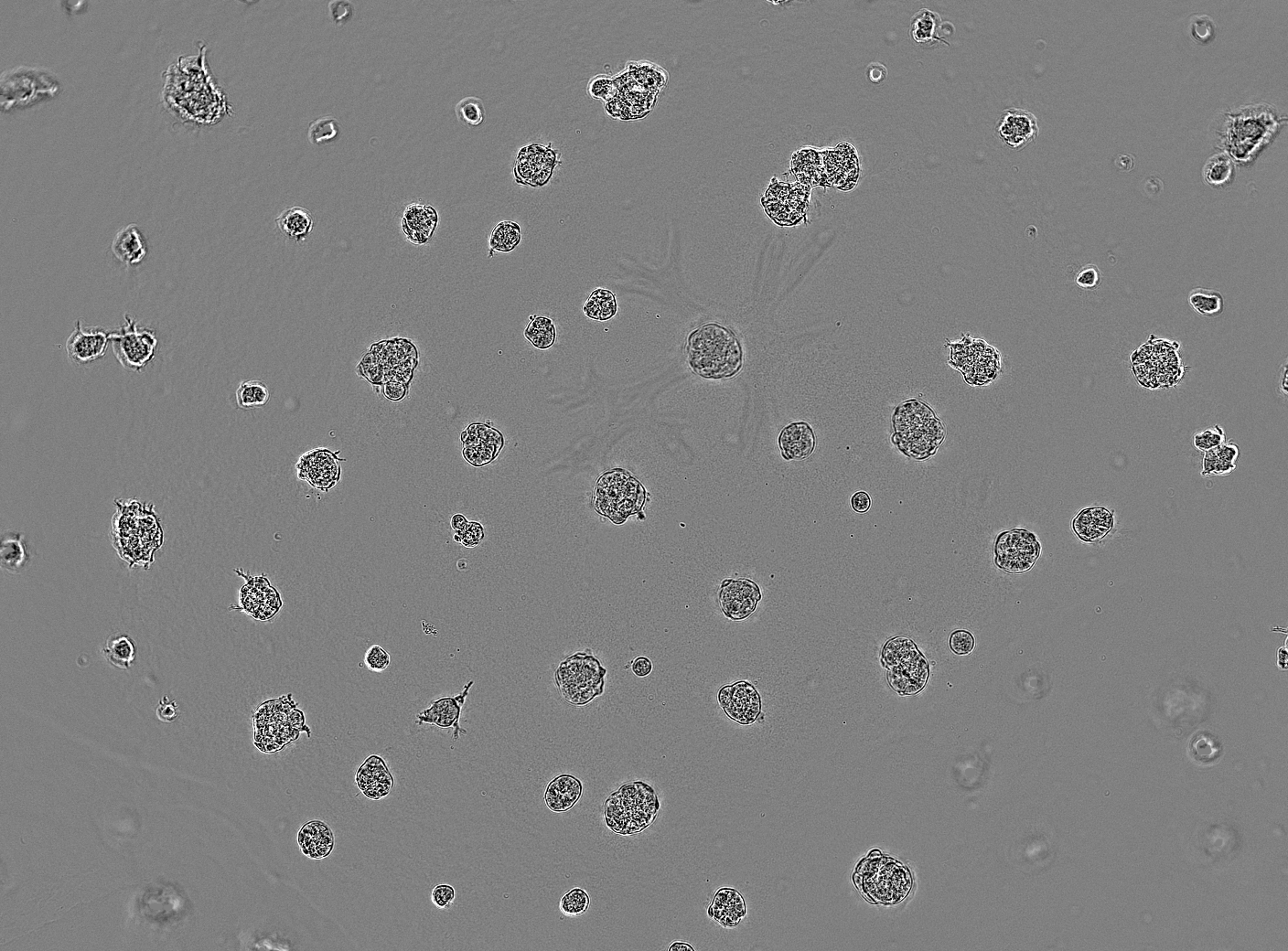

Supplement: Supplementary file 21 — Source Data for Figure 3 [file EMBJ-42-e113987-s002.zip › Figure 3/3G/Akti t=72h_Full.tif]

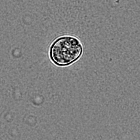

Supplement: Supplementary file 21 — Source Data for Figure 3 [file EMBJ-42-e113987-s002.zip › Figure 3/3G/PI3Ki t=36h.tif]

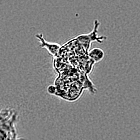

Supplement: Supplementary file 21 — Source Data for Figure 3 [file EMBJ-42-e113987-s002.zip › Figure 3/3G/DMSO t=60h.tif]

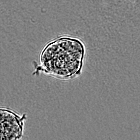

Supplement: Supplementary file 21 — Source Data for Figure 3 [file EMBJ-42-e113987-s002.zip › Figure 3/3G/Akti t=48h.tif]

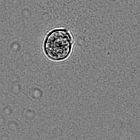

Supplement: Supplementary file 21 — Source Data for Figure 3 [file EMBJ-42-e113987-s002.zip › Figure 3/3G/PI3Ki t=24h.tif]

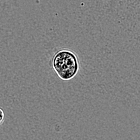

Supplement: Supplementary file 21 — Source Data for Figure 3 [file EMBJ-42-e113987-s002.zip › Figure 3/3G/Akti t=12h.tif]

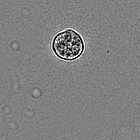

Supplement: Supplementary file 21 — Source Data for Figure 3 [file EMBJ-42-e113987-s002.zip › Figure 3/3G/PI3Ki t=48.tif]

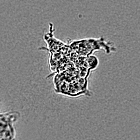

Supplement: Supplementary file 21 — Source Data for Figure 3 [file EMBJ-42-e113987-s002.zip › Figure 3/3G/DMSO t=72h.tif]

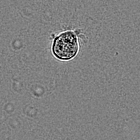

Supplement: Supplementary file 21 — Source Data for Figure 3 [file EMBJ-42-e113987-s002.zip › Figure 3/3G/PI3Ki t=12.tif]

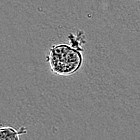

Supplement: Supplementary file 21 — Source Data for Figure 3 [file EMBJ-42-e113987-s002.zip › Figure 3/3G/Akti t=24h.tif]

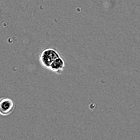

Supplement: Supplementary file 21 — Source Data for Figure 3 [file EMBJ-42-e113987-s002.zip › Figure 3/3G/Akti t=1h.tif]

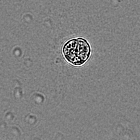

Supplement: Supplementary file 21 — Source Data for Figure 3 [file EMBJ-42-e113987-s002.zip › Figure 3/3G/PI3Ki t=72h.tif]

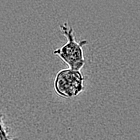

Supplement: Supplementary file 21 — Source Data for Figure 3 [file EMBJ-42-e113987-s002.zip › Figure 3/3G/DMSO t=24h.tif]

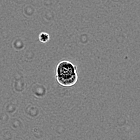

Supplement: Supplementary file 21 — Source Data for Figure 3 [file EMBJ-42-e113987-s002.zip › Figure 3/3G/PI3Ki t=1h.tif]

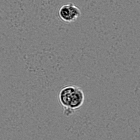

Supplement: Supplementary file 21 — Source Data for Figure 3 [file EMBJ-42-e113987-s002.zip › Figure 3/3G/DMSO t=1h.tif]

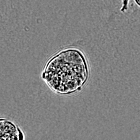

Supplement: Supplementary file 21 — Source Data for Figure 3 [file EMBJ-42-e113987-s002.zip › Figure 3/3G/Akti t=60h.tif]

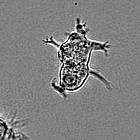

Supplement: Supplementary file 21 — Source Data for Figure 3 [file EMBJ-42-e113987-s002.zip › Figure 3/3G/DMSO t=48h.tif]

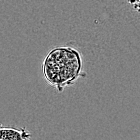

Supplement: Supplementary file 21 — Source Data for Figure 3 [file EMBJ-42-e113987-s002.zip › Figure 3/3G/Akti t=36h.tif]

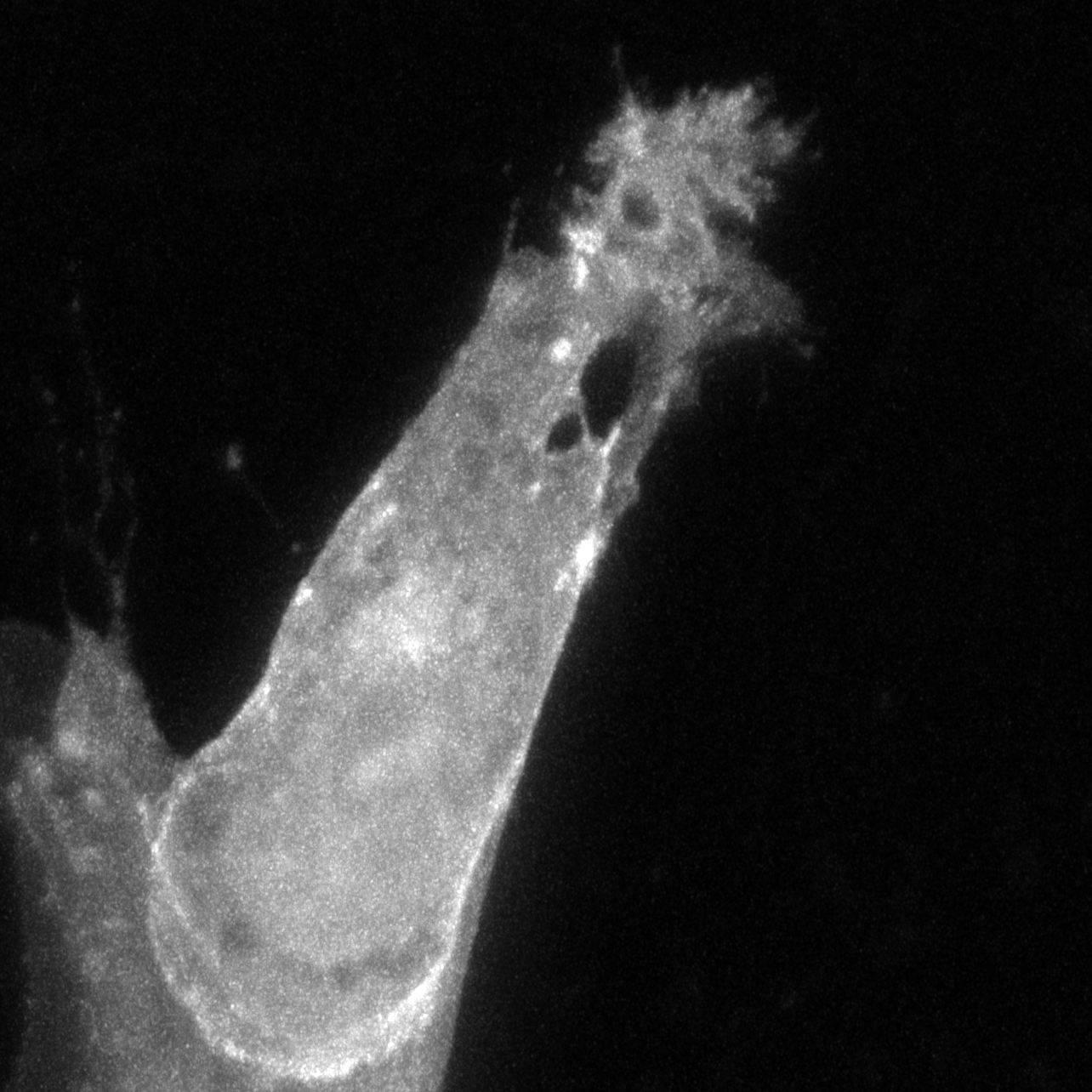

Supplement: Supplementary file 22 — Source Data for Figure 4 [file EMBJ-42-e113987-s026.zip › Figure 4/4D/mNG ARF6-Trp53-:- mNG Arf6.tif]

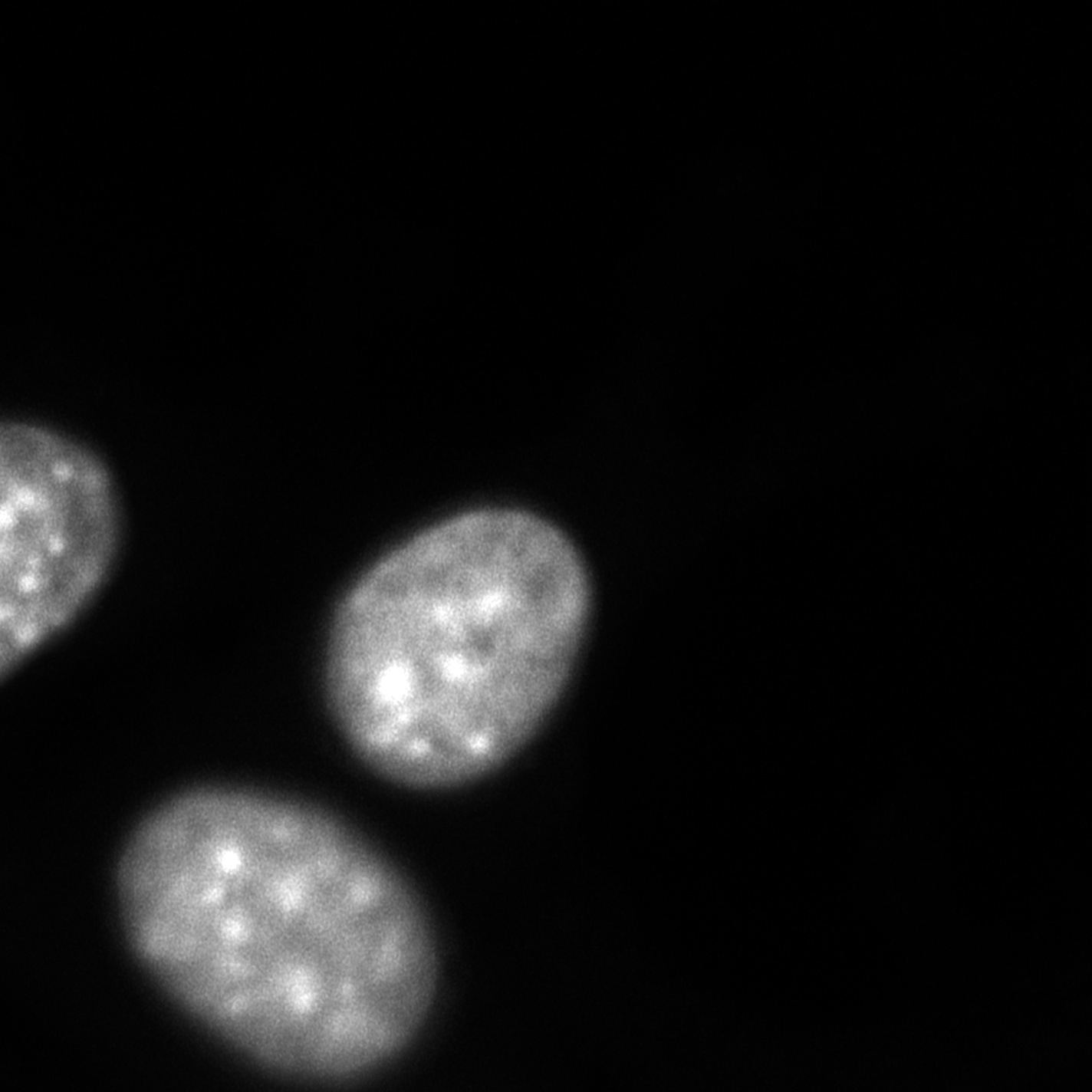

Supplement: Supplementary file 22 — Source Data for Figure 4 [file EMBJ-42-e113987-s026.zip › Figure 4/4D/HOECSHT-MAX_Trp53-:- PTEN-:- mNG Arf6.tif]

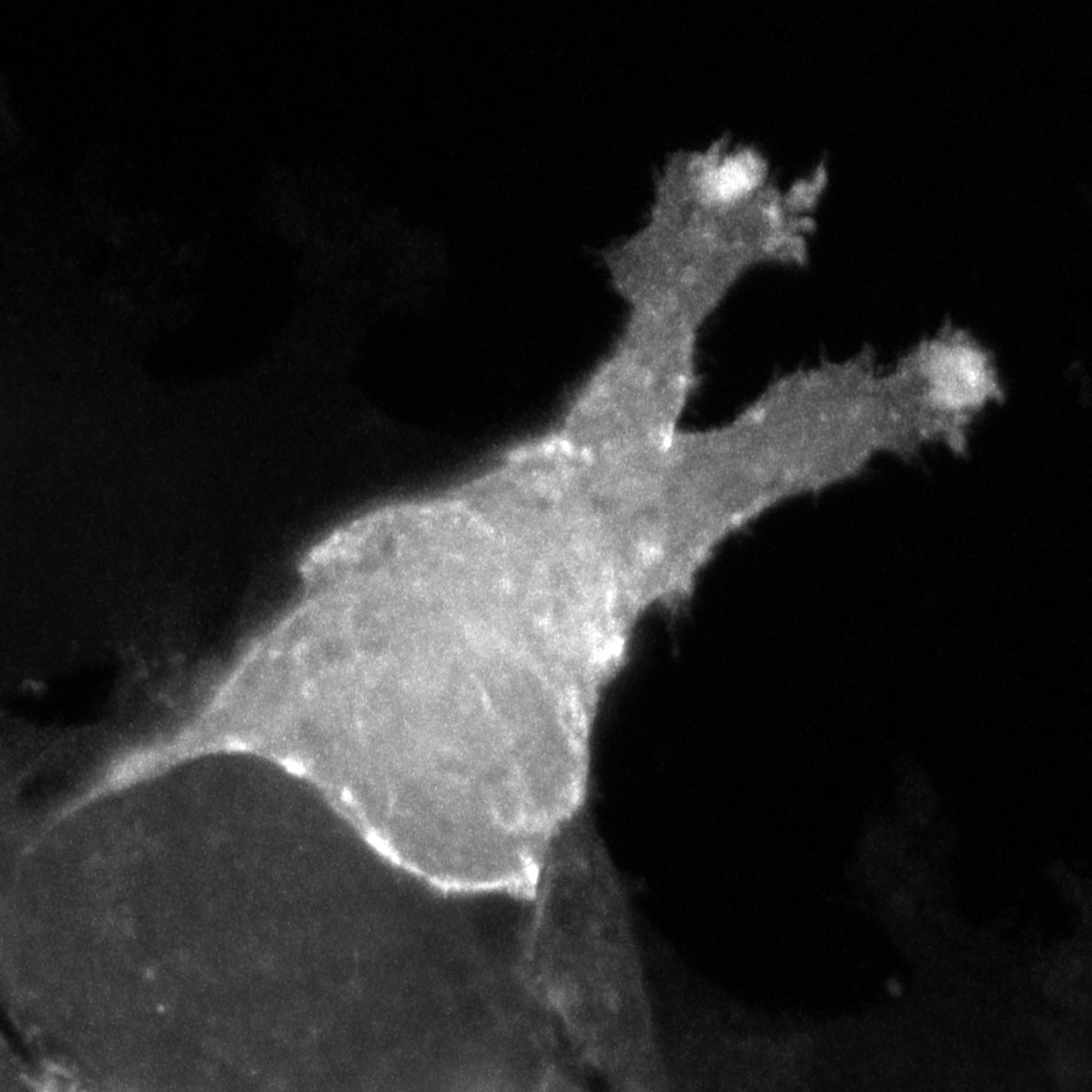

Supplement: Supplementary file 22 — Source Data for Figure 4 [file EMBJ-42-e113987-s026.zip › Figure 4/4D/mNG ARF6-MAX_Trp53-:- PTEN-:- mNG Arf6.tif]

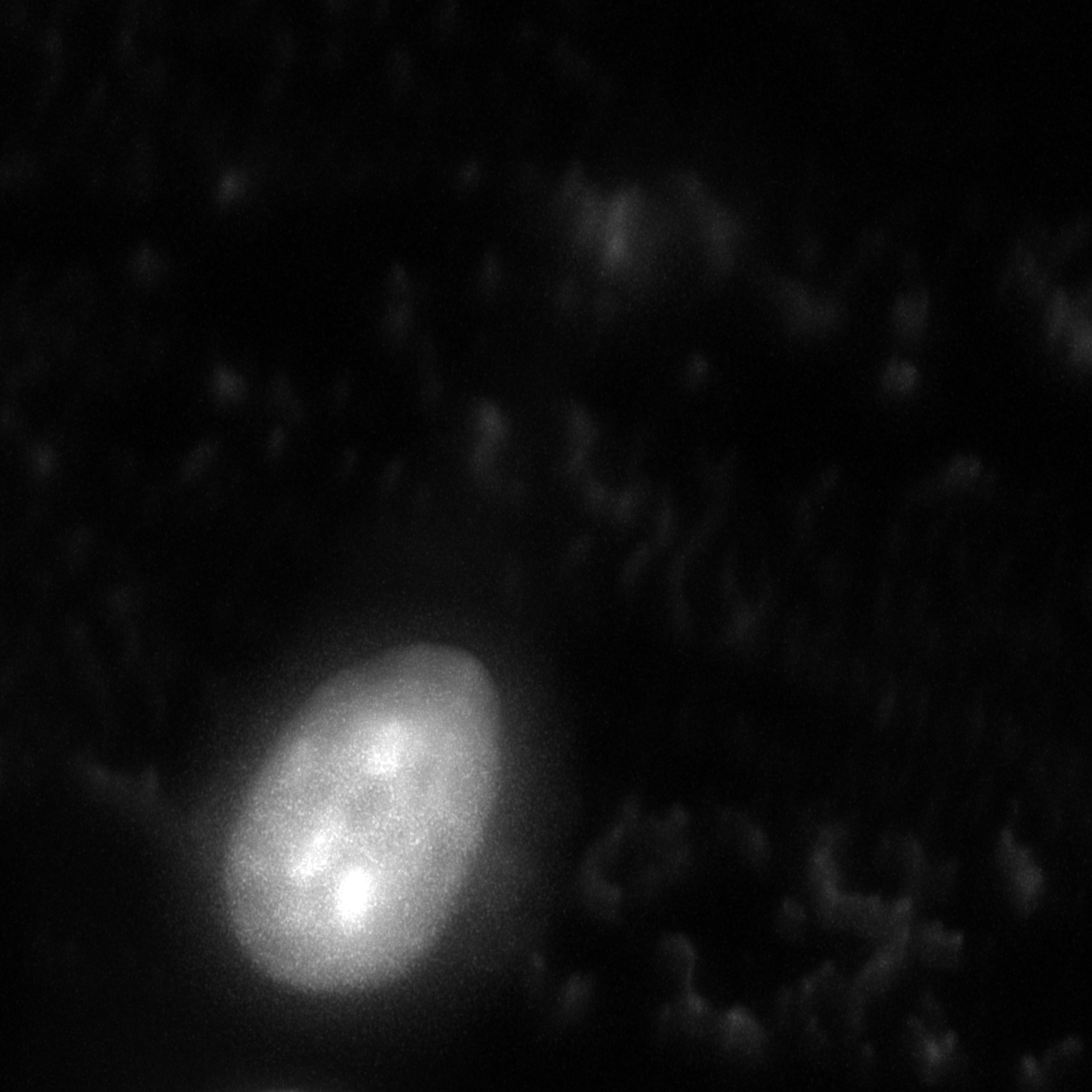

Supplement: Supplementary file 22 — Source Data for Figure 4 [file EMBJ-42-e113987-s026.zip › Figure 4/4D/HOECSHT-MAX_Trp53-:- mNG Arf6.tif]

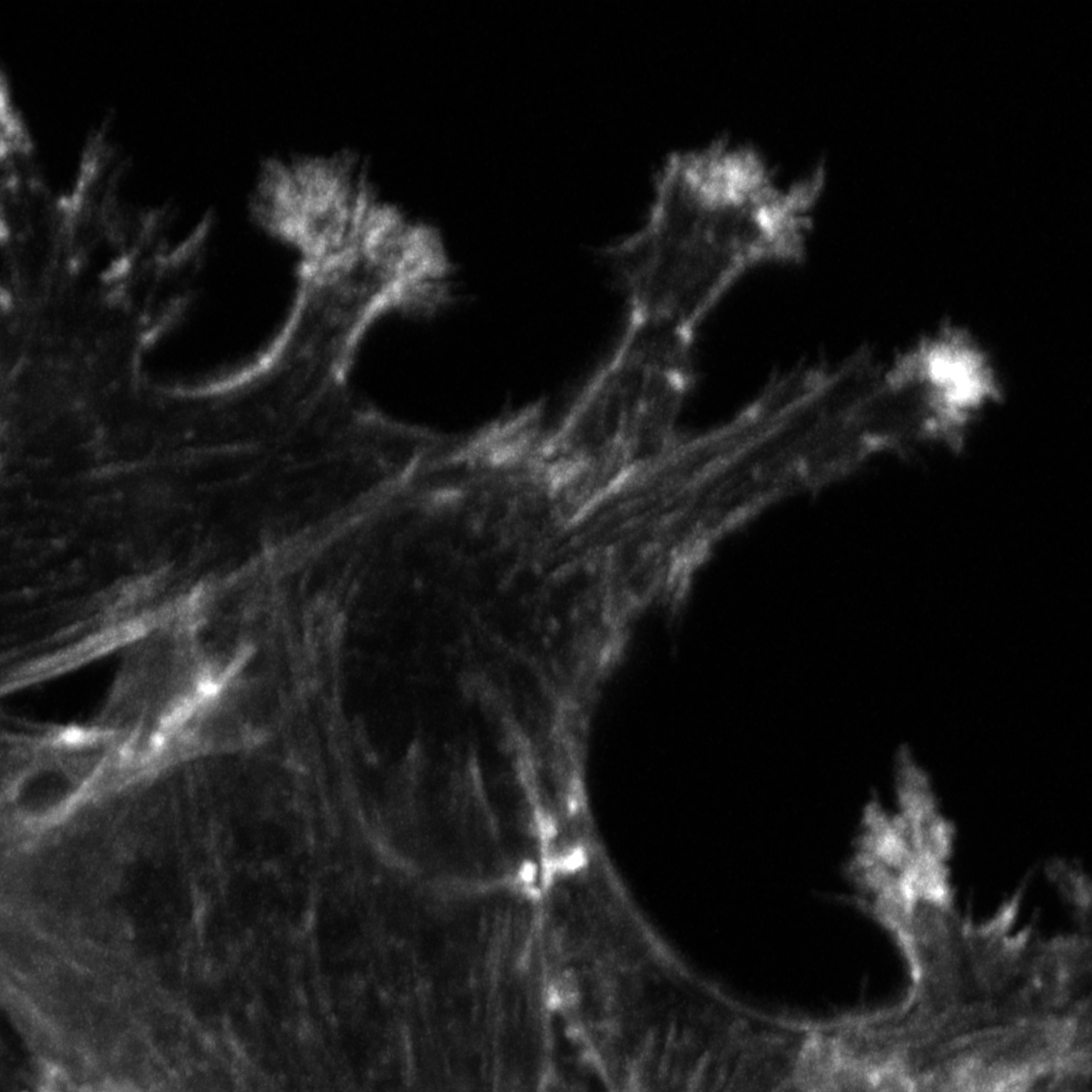

Supplement: Supplementary file 22 — Source Data for Figure 4 [file EMBJ-42-e113987-s026.zip › Figure 4/4D/Actin-MAX_ Trp53-:- PTEN-:- mNG Arf6.tif]

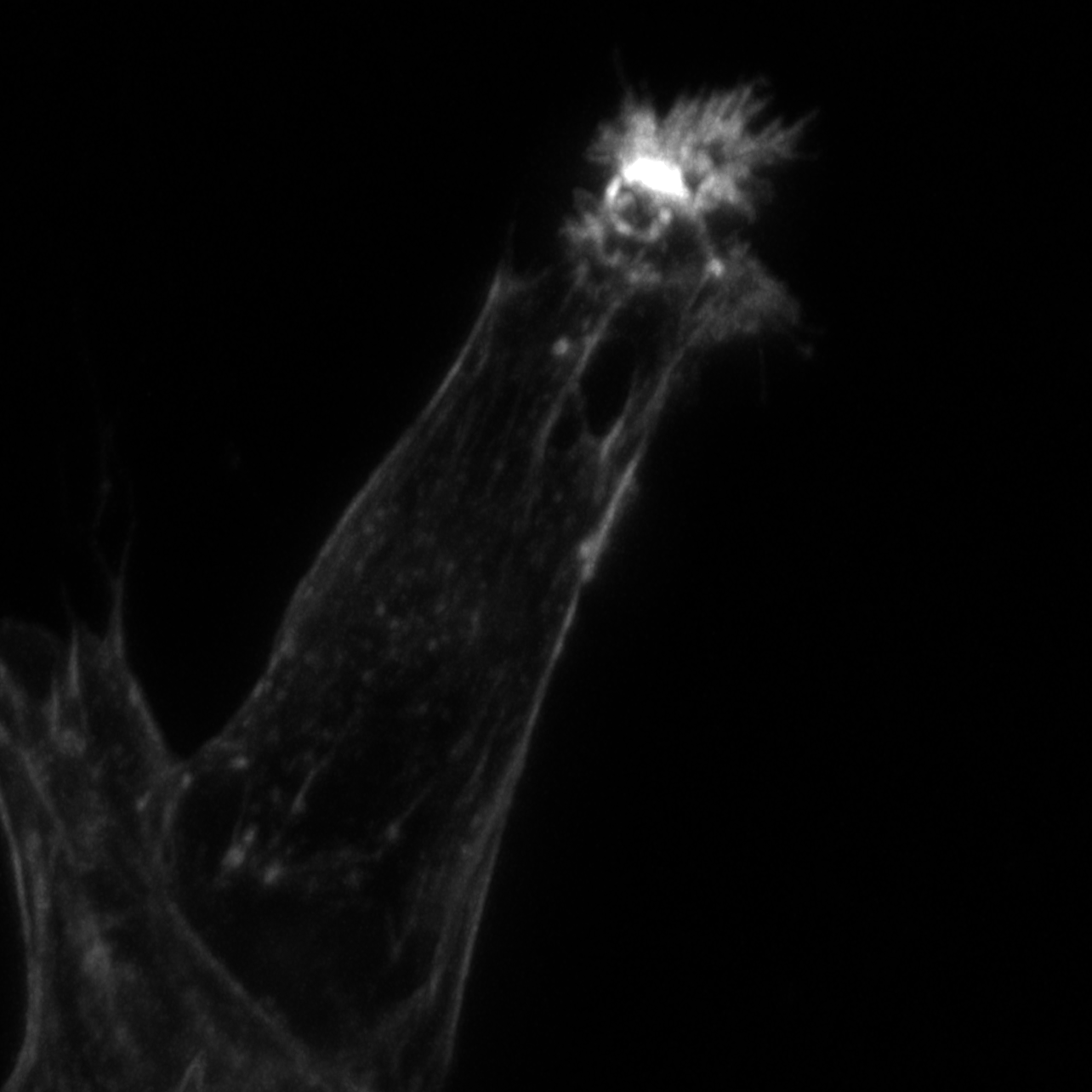

Supplement: Supplementary file 22 — Source Data for Figure 4 [file EMBJ-42-e113987-s026.zip › Figure 4/4D/Actin-MAX_Trp53-:- mNG Arf6.tif]

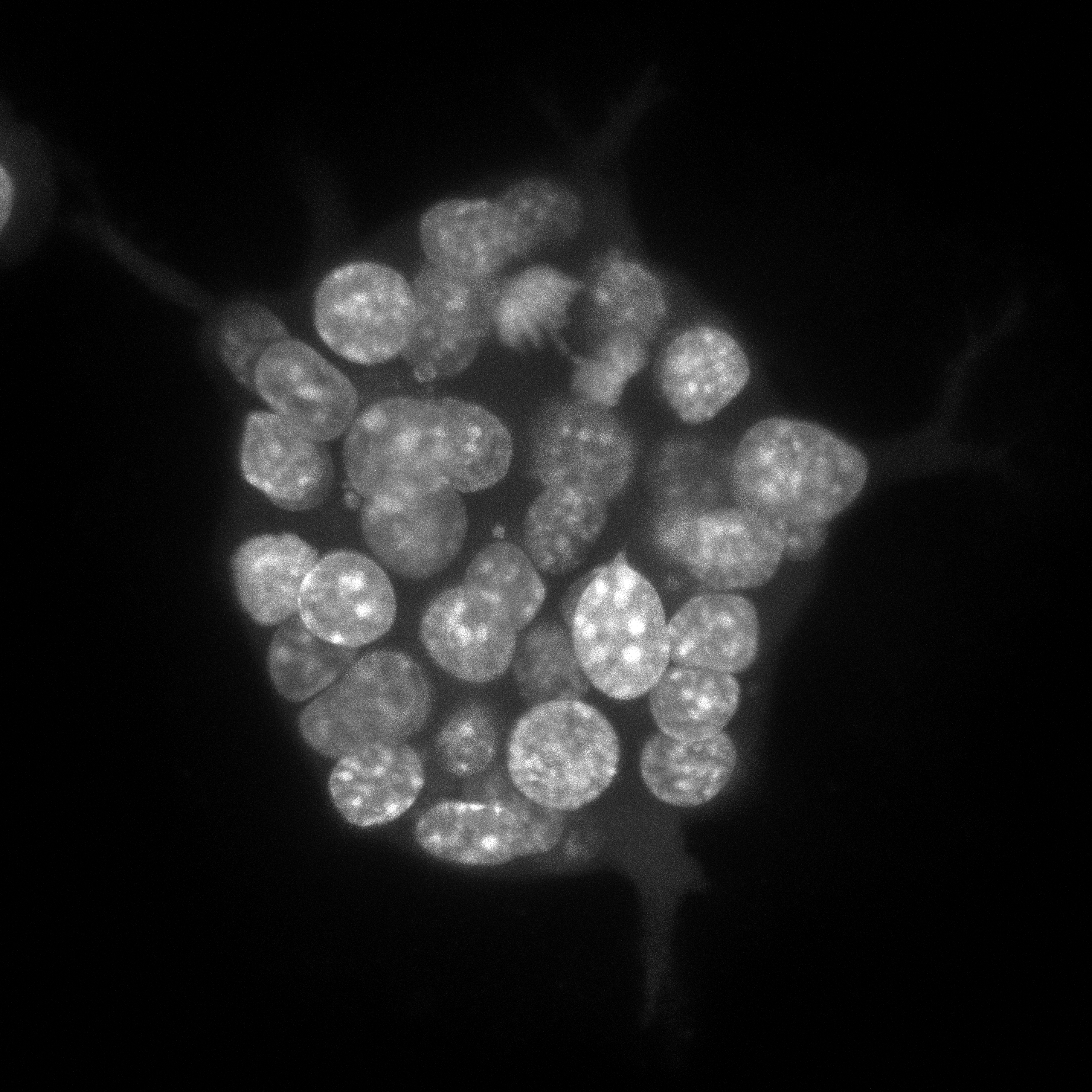

Supplement: Supplementary file 22 — Source Data for Figure 4 [file EMBJ-42-e113987-s026.zip › Figure 4/4F/HOECSHT MAX_Trp53-:- Pten-:- mNG Arf6.tif]

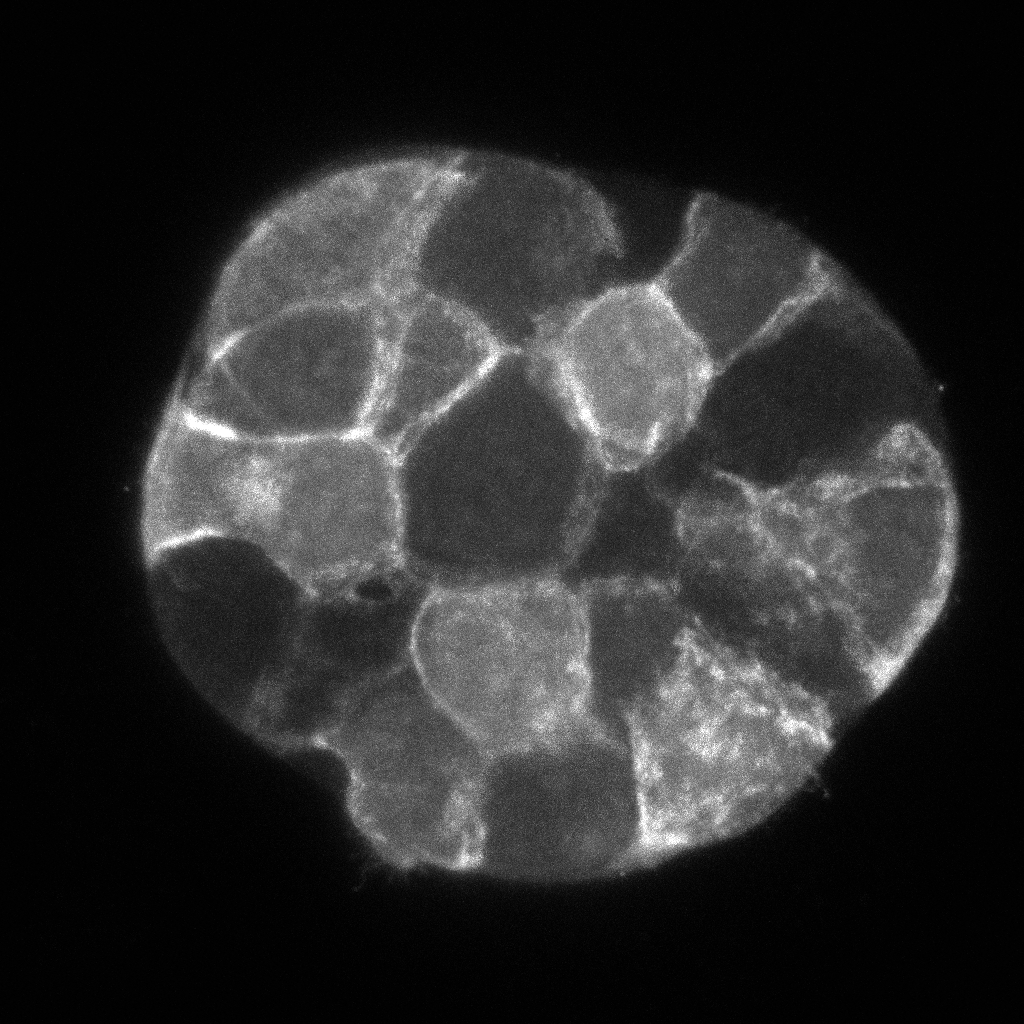

Supplement: Supplementary file 22 — Source Data for Figure 4 [file EMBJ-42-e113987-s026.zip › Figure 4/4F/mNG Arf6-MAX z=40-45 Trp53-:- mNG Arf6.tif]

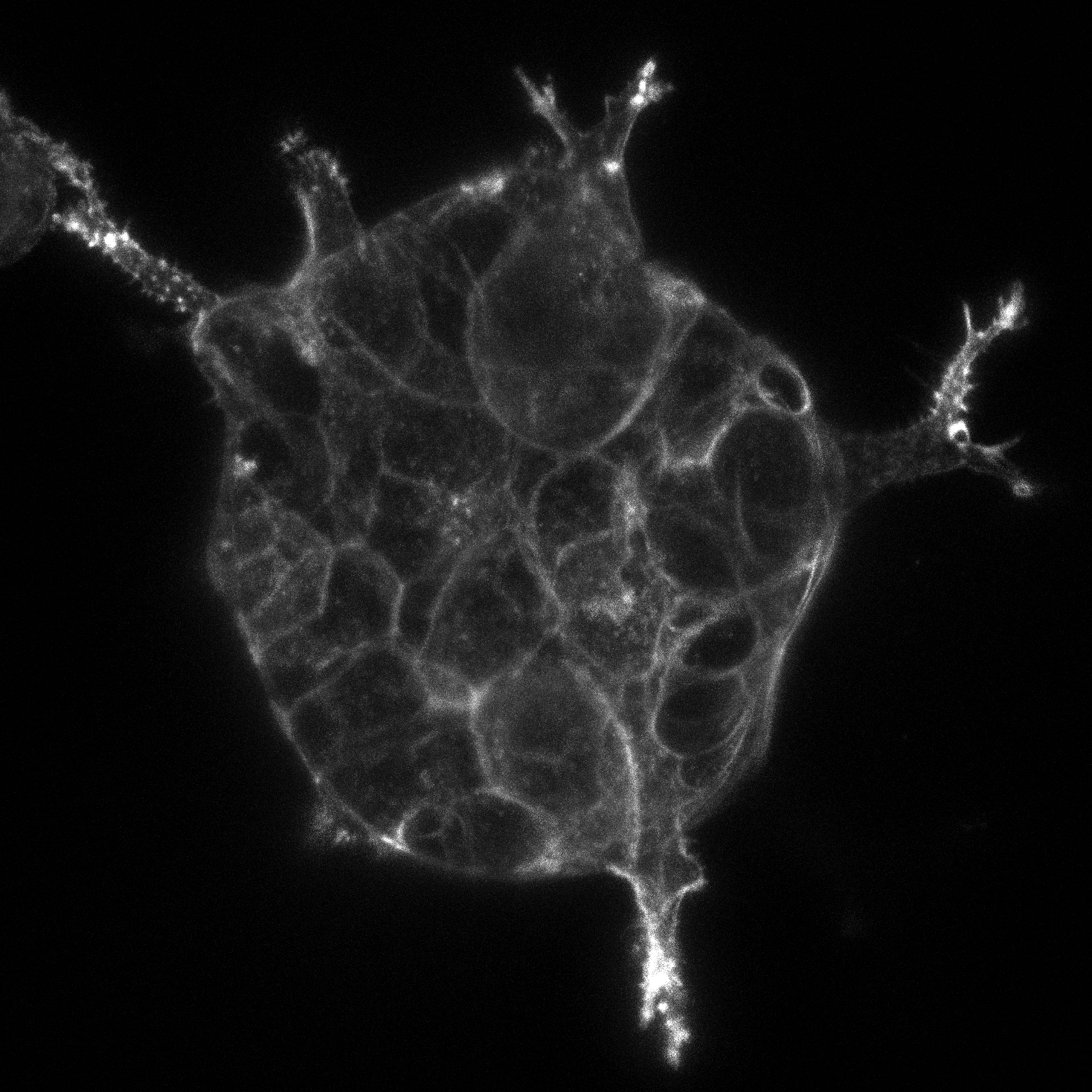

Supplement: Supplementary file 22 — Source Data for Figure 4 [file EMBJ-42-e113987-s026.zip › Figure 4/4F/F-actin-MAX_Trp53-:- Pten-:- mNG Arf6.tif]

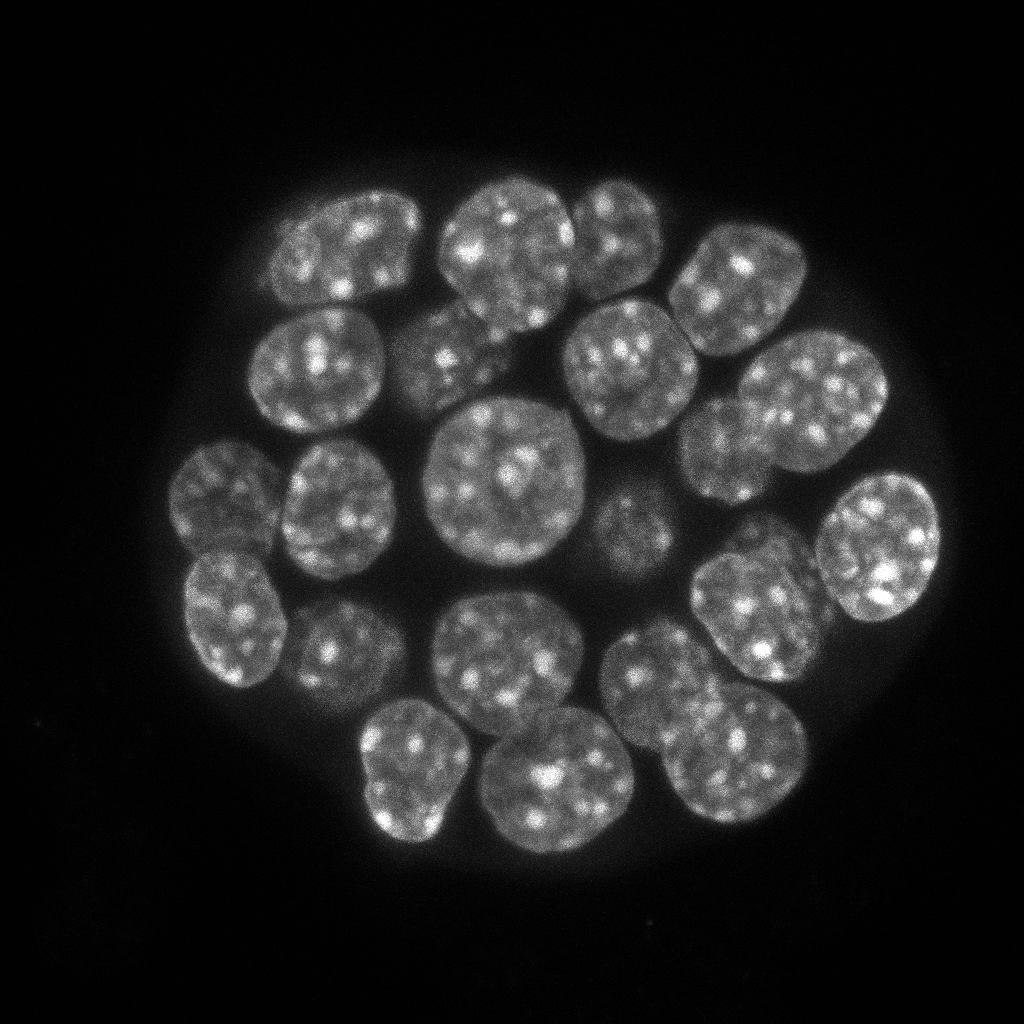

Supplement: Supplementary file 22 — Source Data for Figure 4 [file EMBJ-42-e113987-s026.zip › Figure 4/4F/HOECHST MAX z=40-45MAX Trp53-:- mNG Arf6.tif]

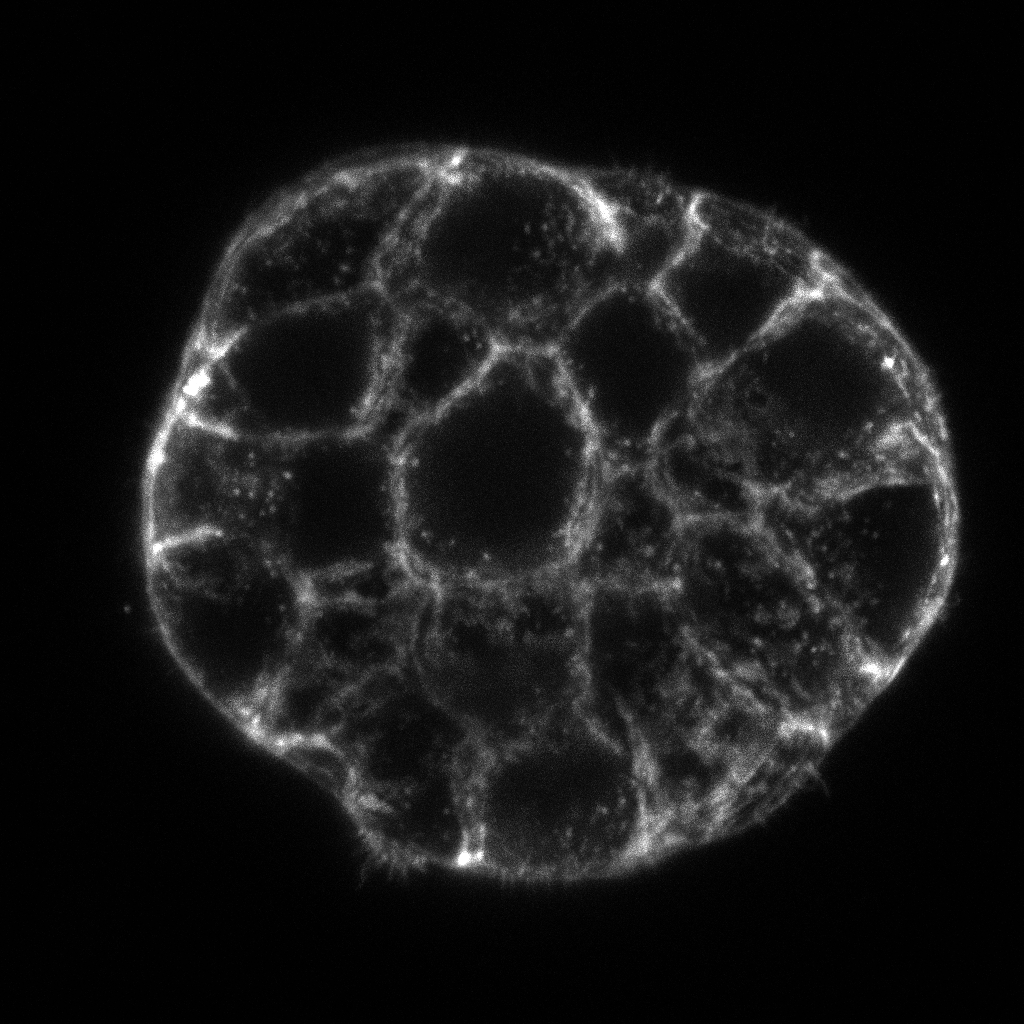

Supplement: Supplementary file 22 — Source Data for Figure 4 [file EMBJ-42-e113987-s026.zip › Figure 4/4F/F-actin-MAX z=40-45 Trp53-:- mNG Arf6.tif]

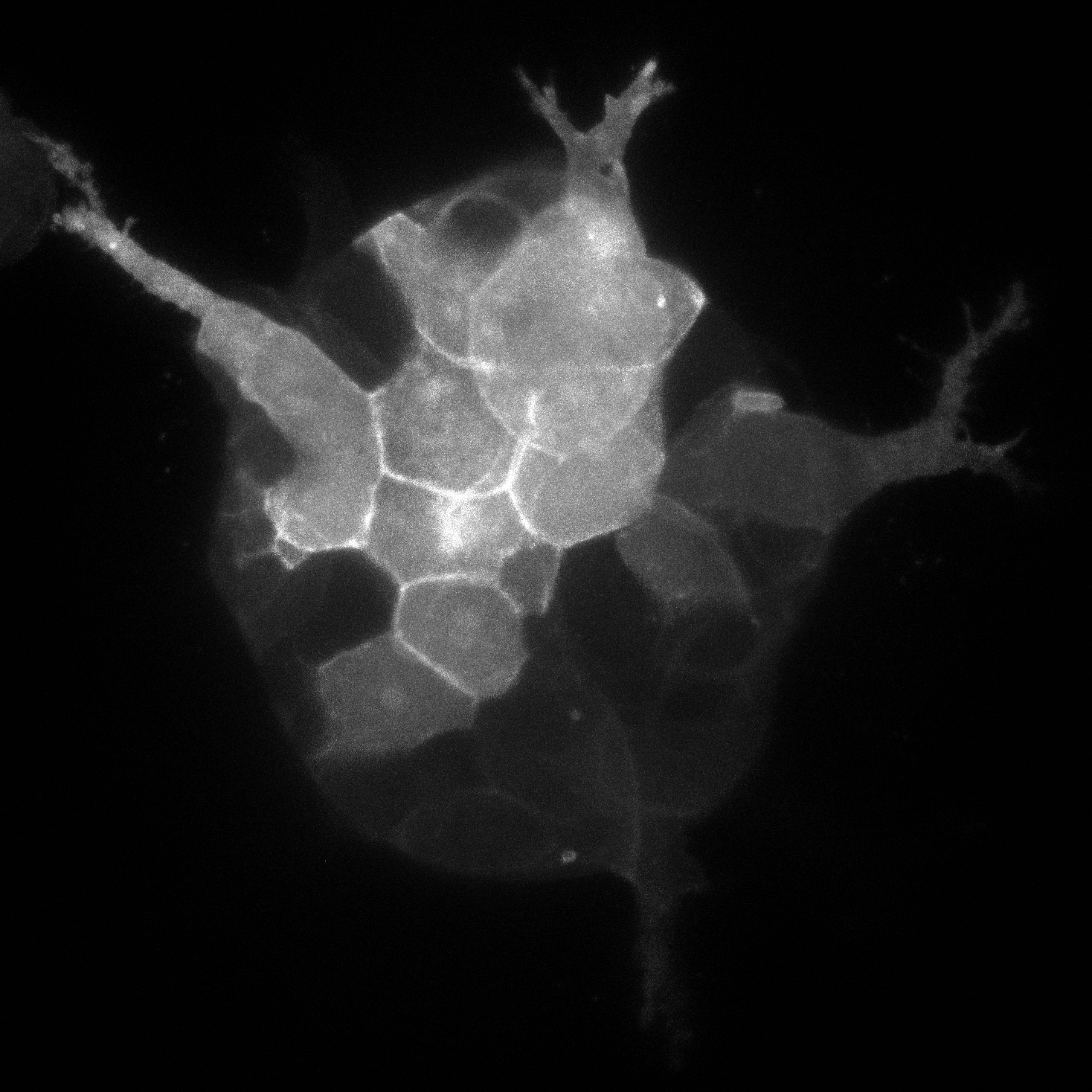

Supplement: Supplementary file 22 — Source Data for Figure 4 [file EMBJ-42-e113987-s026.zip › Figure 4/4F/mNG Arf6 MAX_Trp53-:- Pten-:- mNG Arf6.tif]

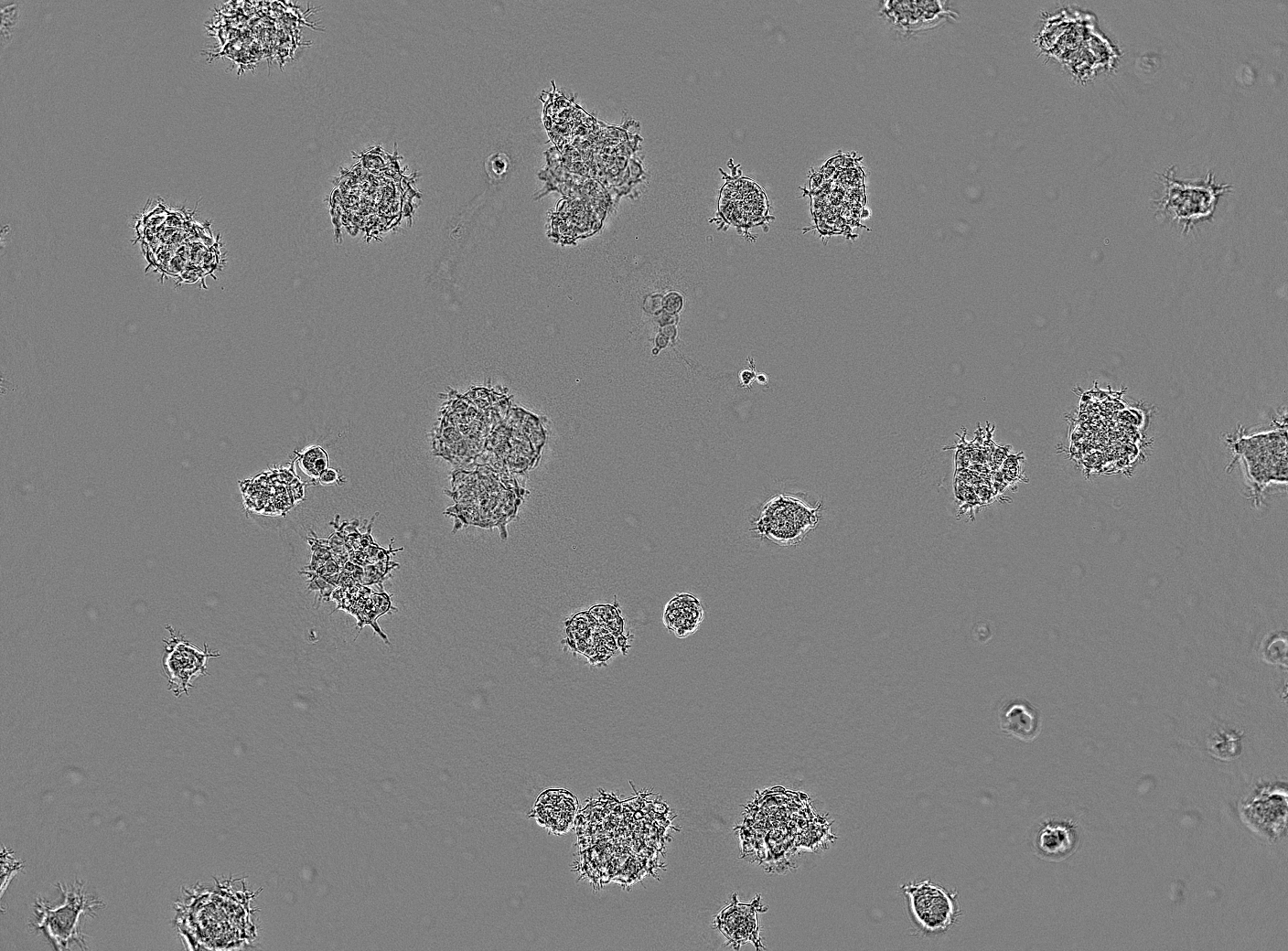

Supplement: Supplementary file 22 — Source Data for Figure 4 [file EMBJ-42-e113987-s026.zip › Figure 4/4C/ARF5 KD t=72h_Full.tif]

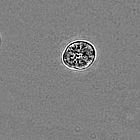

Supplement: Supplementary file 22 — Source Data for Figure 4 [file EMBJ-42-e113987-s026.zip › Figure 4/4C/ARF6 KD t=24h.tif]

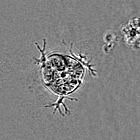

Supplement: Supplementary file 22 — Source Data for Figure 4 [file EMBJ-42-e113987-s026.zip › Figure 4/4C/Scramble t=36h.tif]

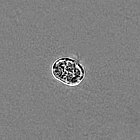

Supplement: Supplementary file 22 — Source Data for Figure 4 [file EMBJ-42-e113987-s026.zip › Figure 4/4C/ARF6 KD t=60h.tif]

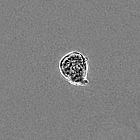

Supplement: Supplementary file 22 — Source Data for Figure 4 [file EMBJ-42-e113987-s026.zip › Figure 4/4C/ARF6 KD t=48h.tif]

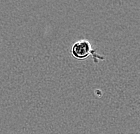

Supplement: Supplementary file 22 — Source Data for Figure 4 [file EMBJ-42-e113987-s026.zip › Figure 4/4C/ARF5 KD t=12h.tif]

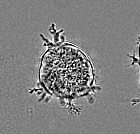

Supplement: Supplementary file 22 — Source Data for Figure 4 [file EMBJ-42-e113987-s026.zip › Figure 4/4C/ARF5 KD t=72.tif]

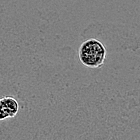

Supplement: Supplementary file 22 — Source Data for Figure 4 [file EMBJ-42-e113987-s026.zip › Figure 4/4C/ARF6 KD t=1h.tif]

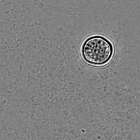

Supplement: Supplementary file 22 — Source Data for Figure 4 [file EMBJ-42-e113987-s026.zip › Figure 4/4C/ARF6 KD t=12h.tif]

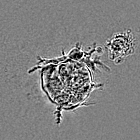

Supplement: Supplementary file 22 — Source Data for Figure 4 [file EMBJ-42-e113987-s026.zip › Figure 4/4C/Scramble t=48h.tif]

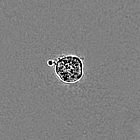

Supplement: Supplementary file 22 — Source Data for Figure 4 [file EMBJ-42-e113987-s026.zip › Figure 4/4C/ARF6 KD t=72h.tif]

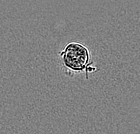

Supplement: Supplementary file 22 — Source Data for Figure 4 [file EMBJ-42-e113987-s026.zip › Figure 4/4C/ARF5 KD t=24h.tif]

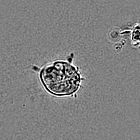

Supplement: Supplementary file 22 — Source Data for Figure 4 [file EMBJ-42-e113987-s026.zip › Figure 4/4C/Scramble t=24h.tif]

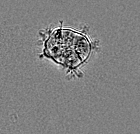

Supplement: Supplementary file 22 — Source Data for Figure 4 [file EMBJ-42-e113987-s026.zip › Figure 4/4C/ARF5 KD t=48h.tif]

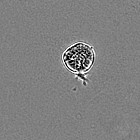

Supplement: Supplementary file 22 — Source Data for Figure 4 [file EMBJ-42-e113987-s026.zip › Figure 4/4C/ARF6 KD t=36h.tif]

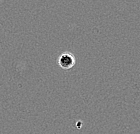

Supplement: Supplementary file 22 — Source Data for Figure 4 [file EMBJ-42-e113987-s026.zip › Figure 4/4C/ARF5 KD t=1h.tif]

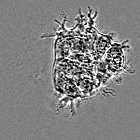

Supplement: Supplementary file 22 — Source Data for Figure 4 [file EMBJ-42-e113987-s026.zip › Figure 4/4C/Scramble t=72h.tif]

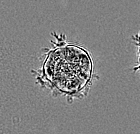

Supplement: Supplementary file 22 — Source Data for Figure 4 [file EMBJ-42-e113987-s026.zip › Figure 4/4C/ARF5 KD t=60h.tif]

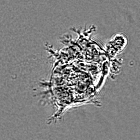

Supplement: Supplementary file 22 — Source Data for Figure 4 [file EMBJ-42-e113987-s026.zip › Figure 4/4C/Scramble t=60h.tif]

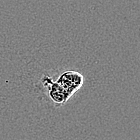

Supplement: Supplementary file 22 — Source Data for Figure 4 [file EMBJ-42-e113987-s026.zip › Figure 4/4C/Scramble t=12h.tif]

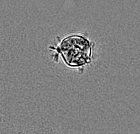

Supplement: Supplementary file 22 — Source Data for Figure 4 [file EMBJ-42-e113987-s026.zip › Figure 4/4C/ARF5 KD t=36h.tif]

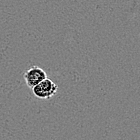

Supplement: Supplementary file 22 — Source Data for Figure 4 [file EMBJ-42-e113987-s026.zip › Figure 4/4C/Scramble t=0h.tif]

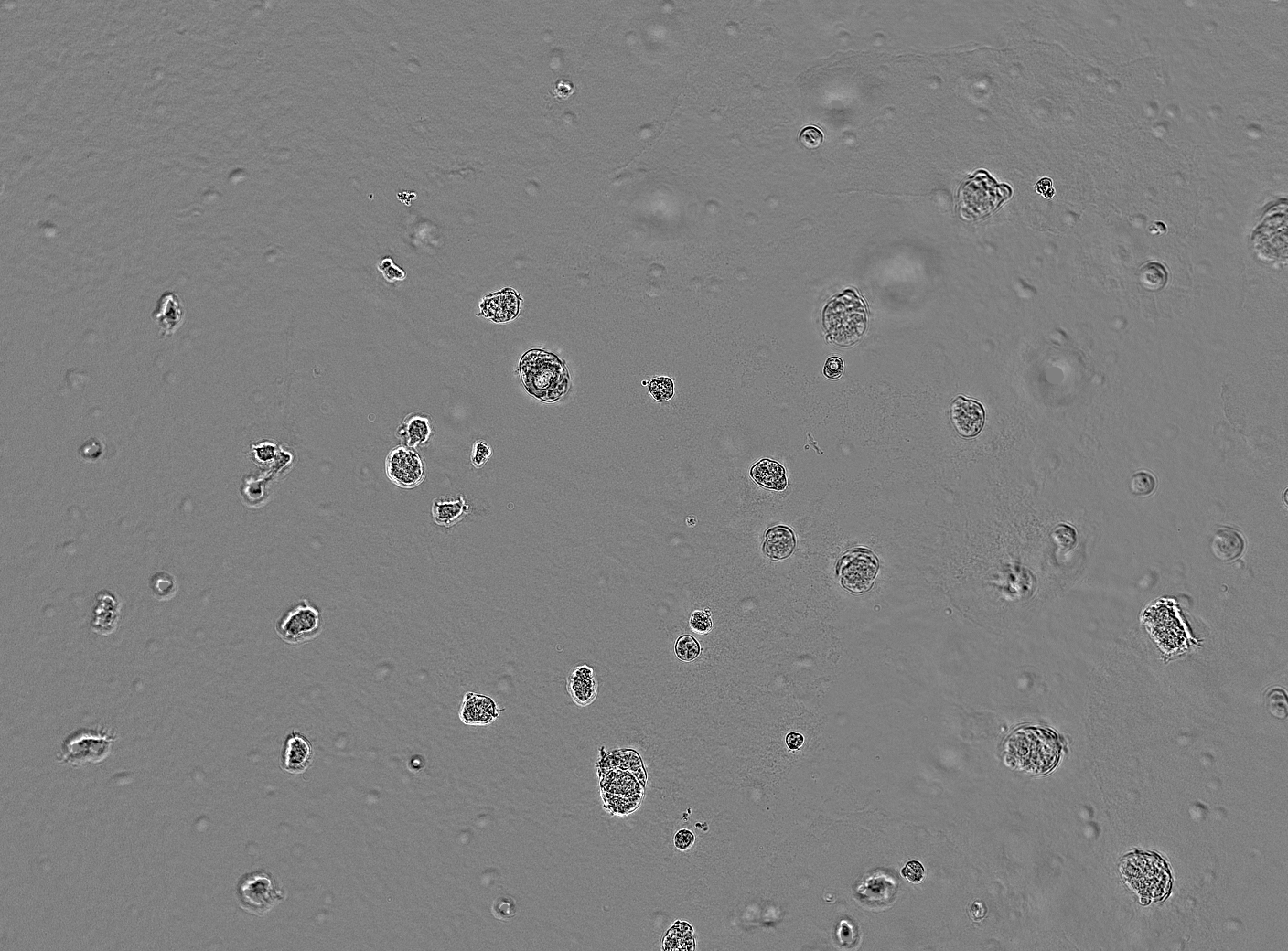

Supplement: Supplementary file 22 — Source Data for Figure 4 [file EMBJ-42-e113987-s026.zip › Figure 4/4C/ARF6 KD t=72h_Full.tif]

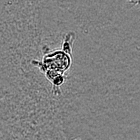

Supplement: Supplementary file 24 — Source Data for Figure 6 [file EMBJ-42-e113987-s009.zip › Figure 6/6G/sgAgap1 + mNG AGAP1_L t=24h.tif]

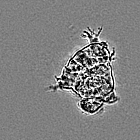

Supplement: Supplementary file 24 — Source Data for Figure 6 [file EMBJ-42-e113987-s009.zip › Figure 6/6G/sgNT + mNG t=72.tif]

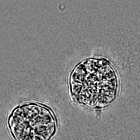

Supplement: Supplementary file 24 — Source Data for Figure 6 [file EMBJ-42-e113987-s009.zip › Figure 6/6G/sgAgap1 + mNG AGAP1_S t=72h.tif]

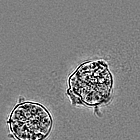

Supplement: Supplementary file 24 — Source Data for Figure 6 [file EMBJ-42-e113987-s009.zip › Figure 6/6G/sgAgap1 + mNG AGAP1_S t=60h.tif]

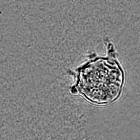

Supplement: Supplementary file 24 — Source Data for Figure 6 [file EMBJ-42-e113987-s009.zip › Figure 6/6G/sgAgap1 + mNG AGAP1_L t=72.tif]

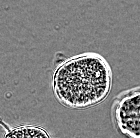

Supplement: Supplementary file 24 — Source Data for Figure 6 [file EMBJ-42-e113987-s009.zip › Figure 6/6G/sgAgap1 + mNG t=60h.tif]

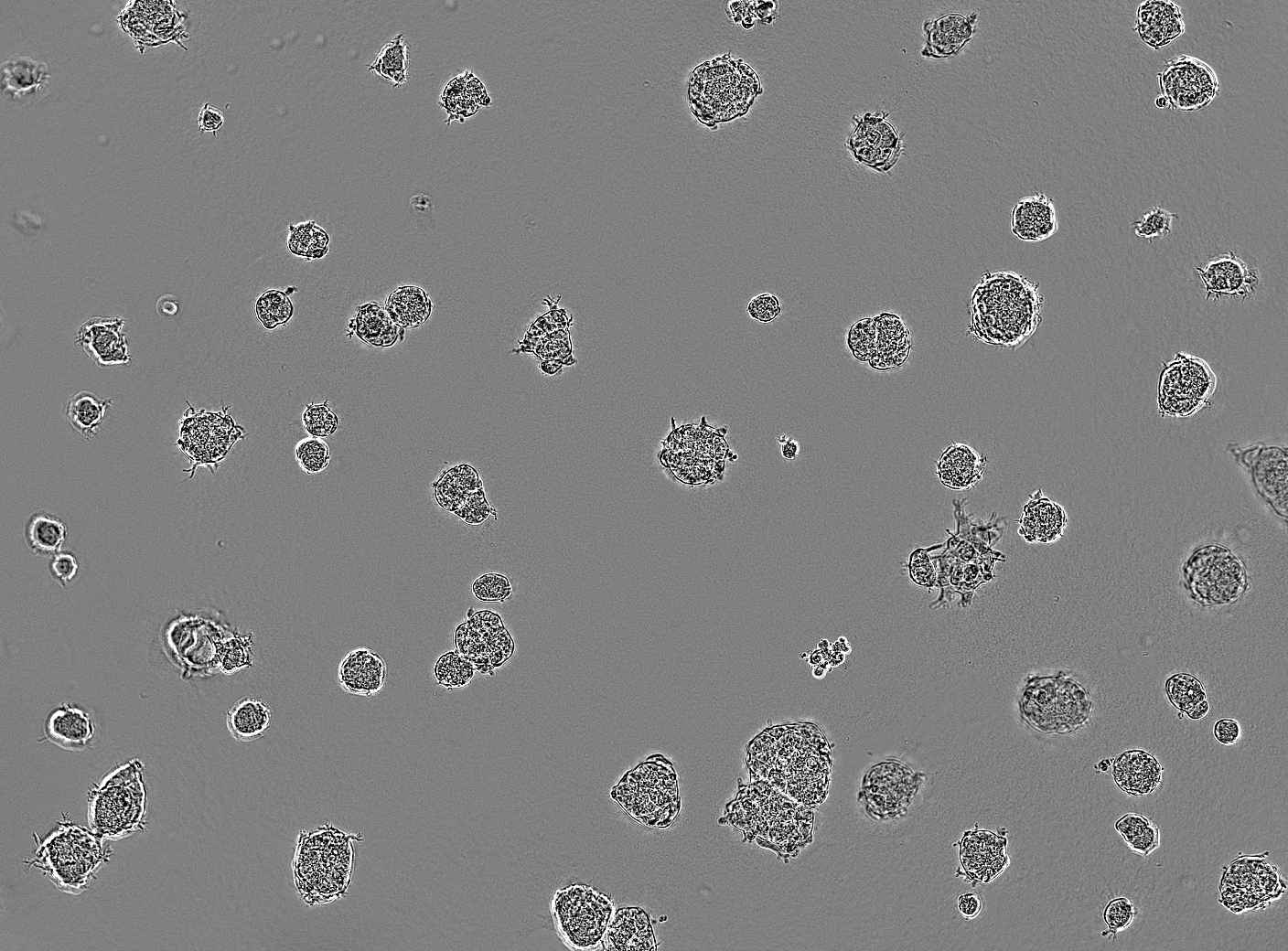

Supplement: Supplementary file 24 — Source Data for Figure 6 [file EMBJ-42-e113987-s009.zip › Figure 6/6G/sgNT + mNG t=72_Full.tif]

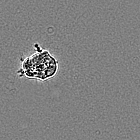

Supplement: Supplementary file 24 — Source Data for Figure 6 [file EMBJ-42-e113987-s009.zip › Figure 6/6G/sgNT + mNG t=24.tif]

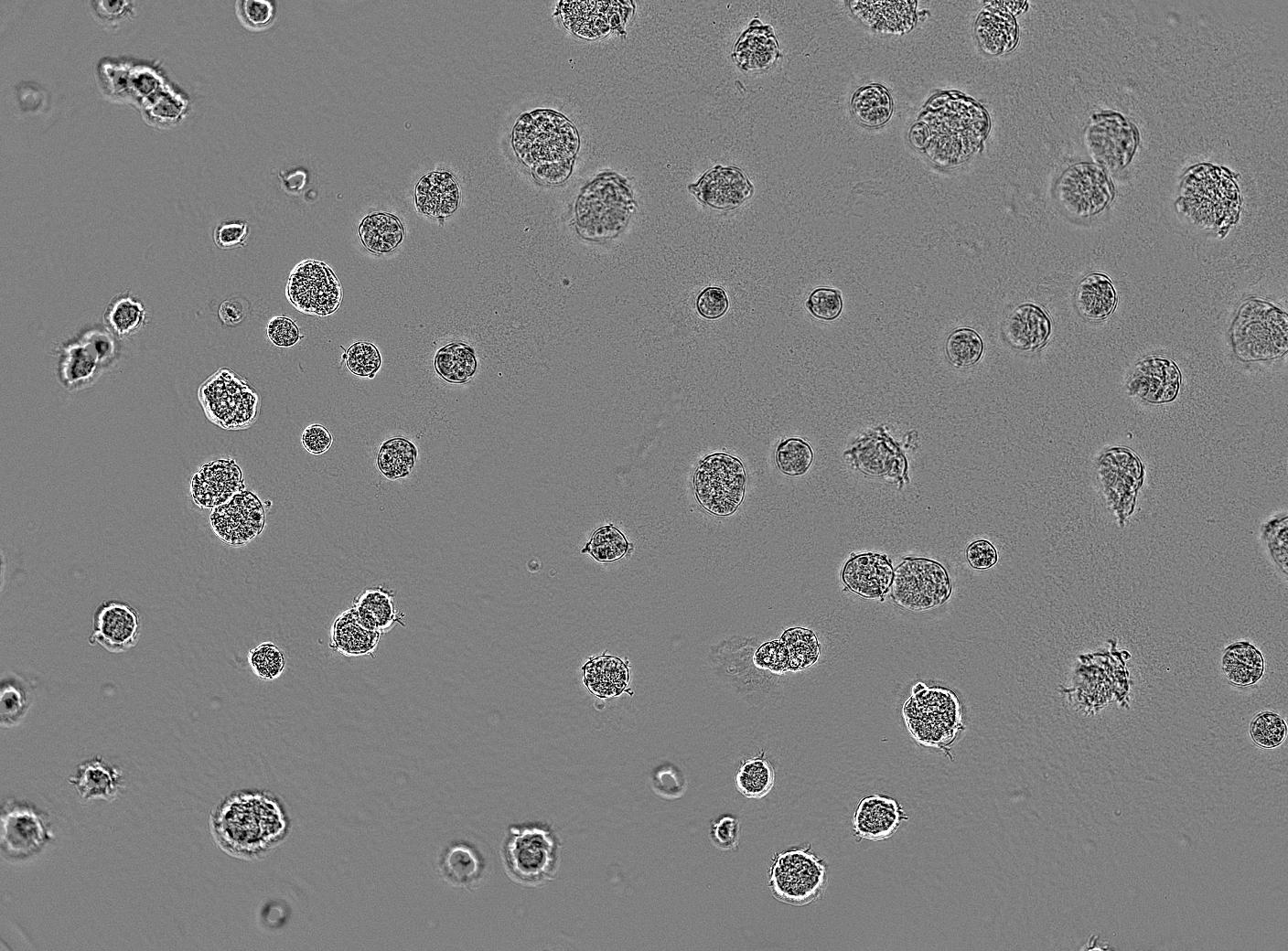

Supplement: Supplementary file 24 — Source Data for Figure 6 [file EMBJ-42-e113987-s009.zip › Figure 6/6G/sgAgap1 + mNG AGAP1_S t=72_Full.tif]

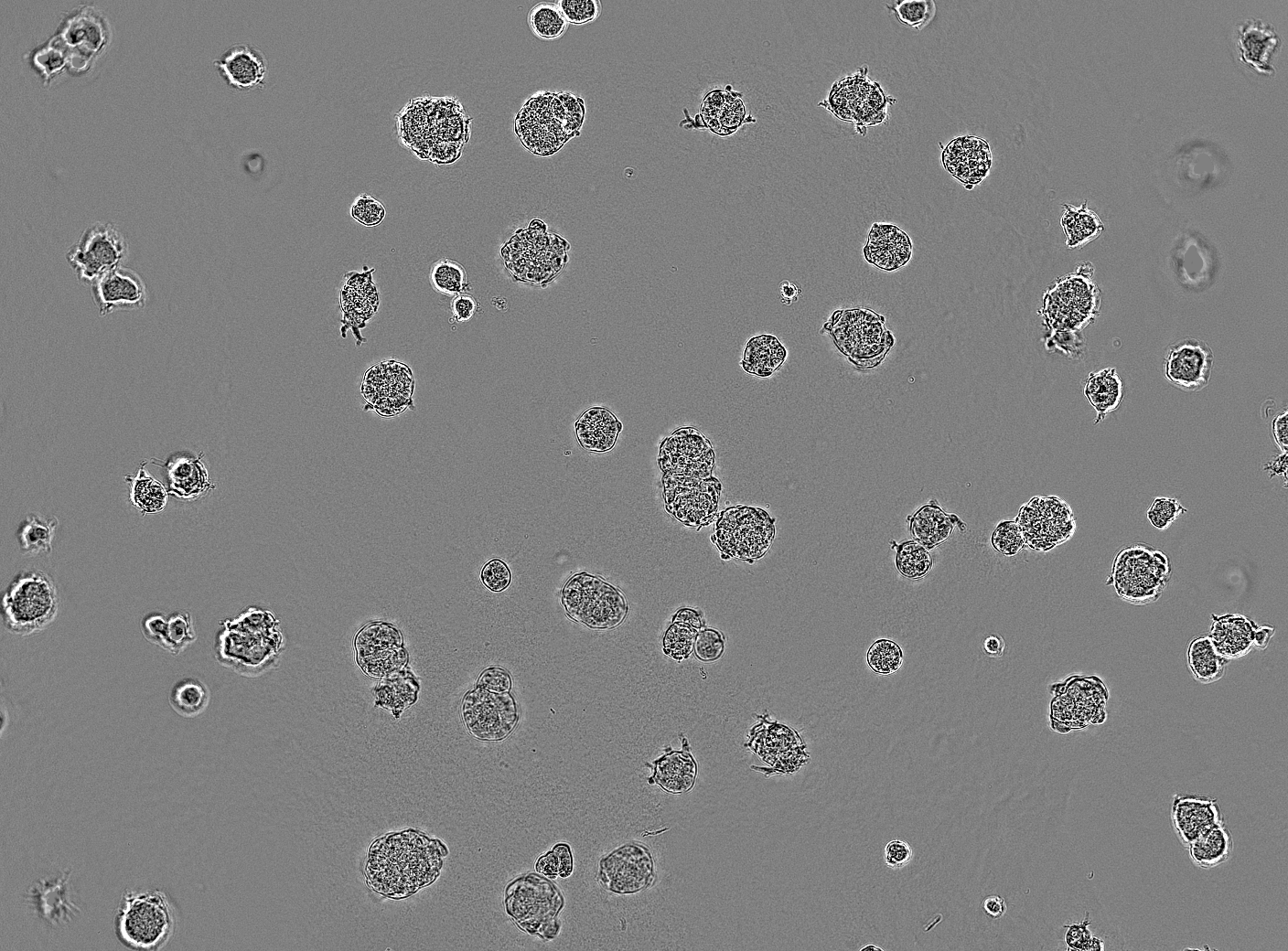

Supplement: Supplementary file 24 — Source Data for Figure 6 [file EMBJ-42-e113987-s009.zip › Figure 6/6G/sgAgap1 + mNG AGAP1_L t=72_Full.tif]

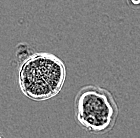

Supplement: Supplementary file 24 — Source Data for Figure 6 [file EMBJ-42-e113987-s009.zip › Figure 6/6G/sgAgap1 + mNG t=48h.tif]

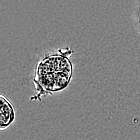

Supplement: Supplementary file 24 — Source Data for Figure 6 [file EMBJ-42-e113987-s009.zip › Figure 6/6G/sgAgap1 + mNG AGAP1_S t=48h.tif]

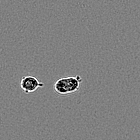

Supplement: Supplementary file 24 — Source Data for Figure 6 [file EMBJ-42-e113987-s009.zip › Figure 6/6G/sgNT + mNG t=1h.tif]

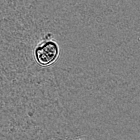

Supplement: Supplementary file 24 — Source Data for Figure 6 [file EMBJ-42-e113987-s009.zip › Figure 6/6G/sgAgap1 + mNG AGAP1_L t=12h.tif]

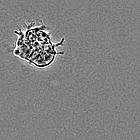

Supplement: Supplementary file 24 — Source Data for Figure 6 [file EMBJ-42-e113987-s009.zip › Figure 6/6G/sgNT + mNG t=36.tif]

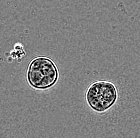

Supplement: Supplementary file 24 — Source Data for Figure 6 [file EMBJ-42-e113987-s009.zip › Figure 6/6G/sgAgap1 + mNG t=24h.tif]

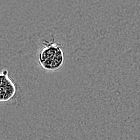

Supplement: Supplementary file 24 — Source Data for Figure 6 [file EMBJ-42-e113987-s009.zip › Figure 6/6G/sgAgap1 + mNG AGAP1_S t=24h.tif]

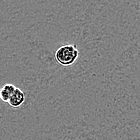

Supplement: Supplementary file 24 — Source Data for Figure 6 [file EMBJ-42-e113987-s009.zip › Figure 6/6G/sgAgap1 + mNG AGAP1_S t=12h.tif]
