# Supplementary figures and images for: PTEN deficiency exposes a requirement for an ARF GTPase module for integrin‐dependent invasion in ovarian cancer (part 2 of 2)
Source: EMBO J. 2023 Aug 14;42(18):e113987. doi: 10.15252/embj.2023113987 (PMC10505920; doi:10.15252/embj.2023113987)

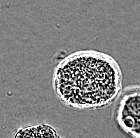

Supplement: Supplementary file 24 — Source Data for Figure 6 [file EMBJ-42-e113987-s009.zip › Figure 6/6G/sgAgap1 + mNG t=72h.tif]

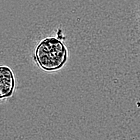

Supplement: Supplementary file 24 — Source Data for Figure 6 [file EMBJ-42-e113987-s009.zip › Figure 6/6G/sgAgap1 + mNG AGAP1_S t=36h.tif]

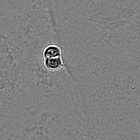

Supplement: Supplementary file 24 — Source Data for Figure 6 [file EMBJ-42-e113987-s009.zip › Figure 6/6G/sgAgap1 + mNG AGAP1_L t=1h.tif]

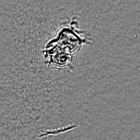

Supplement: Supplementary file 24 — Source Data for Figure 6 [file EMBJ-42-e113987-s009.zip › Figure 6/6G/sgAgap1 + mNG AGAP1_L t=36h.tif]

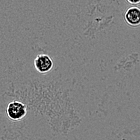

Supplement: Supplementary file 24 — Source Data for Figure 6 [file EMBJ-42-e113987-s009.zip › Figure 6/6G/sgAgap1 + mNG AGAP1_S t=1h.tif]

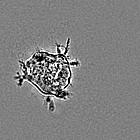

Supplement: Supplementary file 24 — Source Data for Figure 6 [file EMBJ-42-e113987-s009.zip › Figure 6/6G/sgNT + mNG t=48.tif]

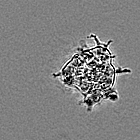

Supplement: Supplementary file 24 — Source Data for Figure 6 [file EMBJ-42-e113987-s009.zip › Figure 6/6G/sgNT + mNG t=60.tif]

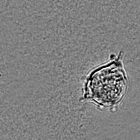

Supplement: Supplementary file 24 — Source Data for Figure 6 [file EMBJ-42-e113987-s009.zip › Figure 6/6G/sgAgap1 + mNG AGAP1_L t=60h.tif]

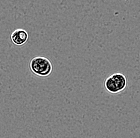

Supplement: Supplementary file 24 — Source Data for Figure 6 [file EMBJ-42-e113987-s009.zip › Figure 6/6G/sgAgap1 + mNG t=1h.tif]

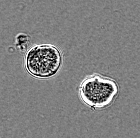

Supplement: Supplementary file 24 — Source Data for Figure 6 [file EMBJ-42-e113987-s009.zip › Figure 6/6G/sgAgap1 + mNG t=36h.tif]

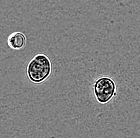

Supplement: Supplementary file 24 — Source Data for Figure 6 [file EMBJ-42-e113987-s009.zip › Figure 6/6G/sgAgap1 + mNG t=12h.tif]

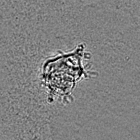

Supplement: Supplementary file 24 — Source Data for Figure 6 [file EMBJ-42-e113987-s009.zip › Figure 6/6G/sgAgap1 + mNG AGAP1_L t=48h.tif]

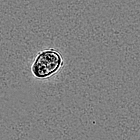

Supplement: Supplementary file 24 — Source Data for Figure 6 [file EMBJ-42-e113987-s009.zip › Figure 6/6G/sgNT + mNG t=12.tif]

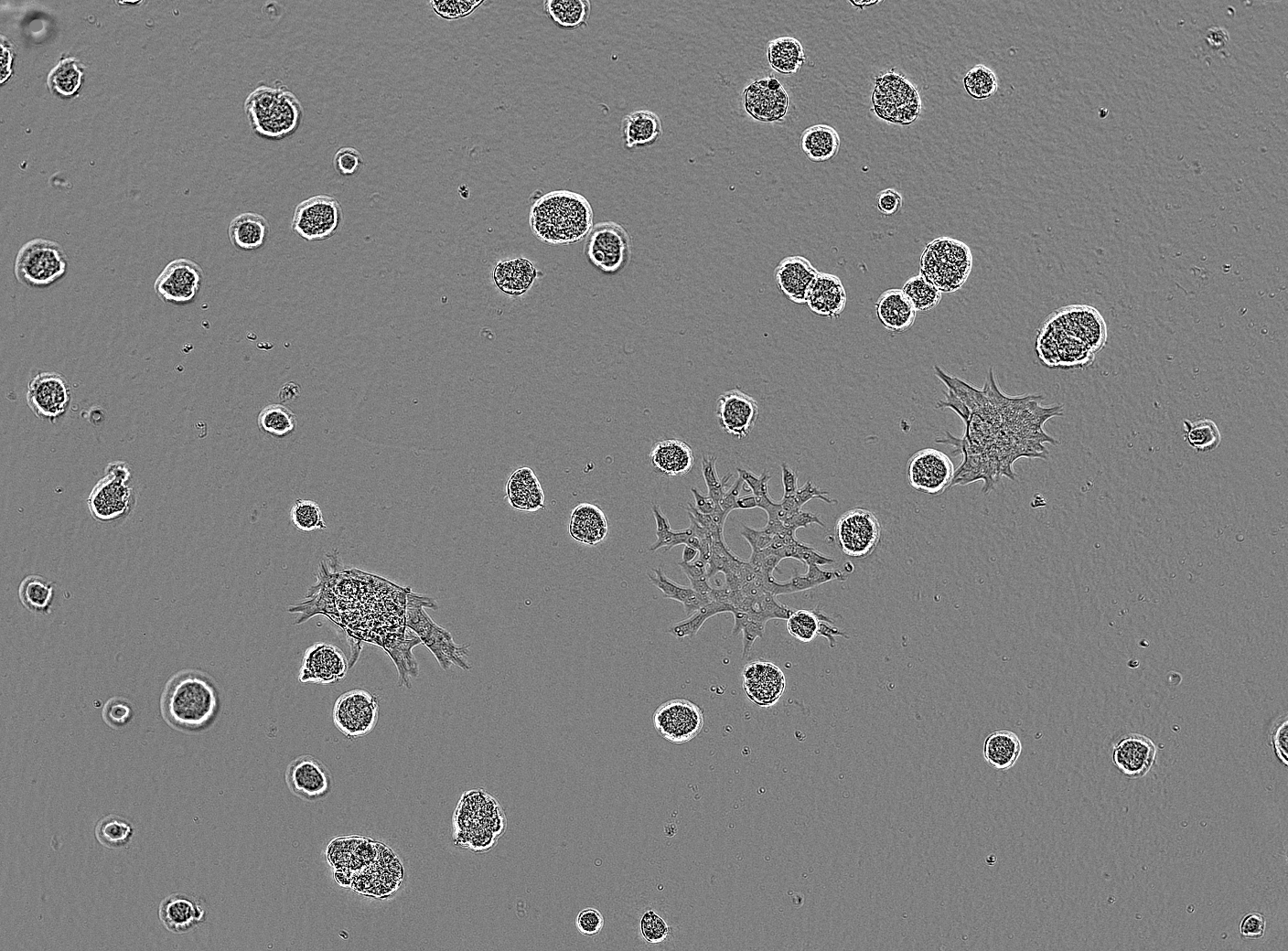

Supplement: Supplementary file 24 — Source Data for Figure 6 [file EMBJ-42-e113987-s009.zip › Figure 6/6G/sgAgap1 + mNG t=72h_Full.tif]

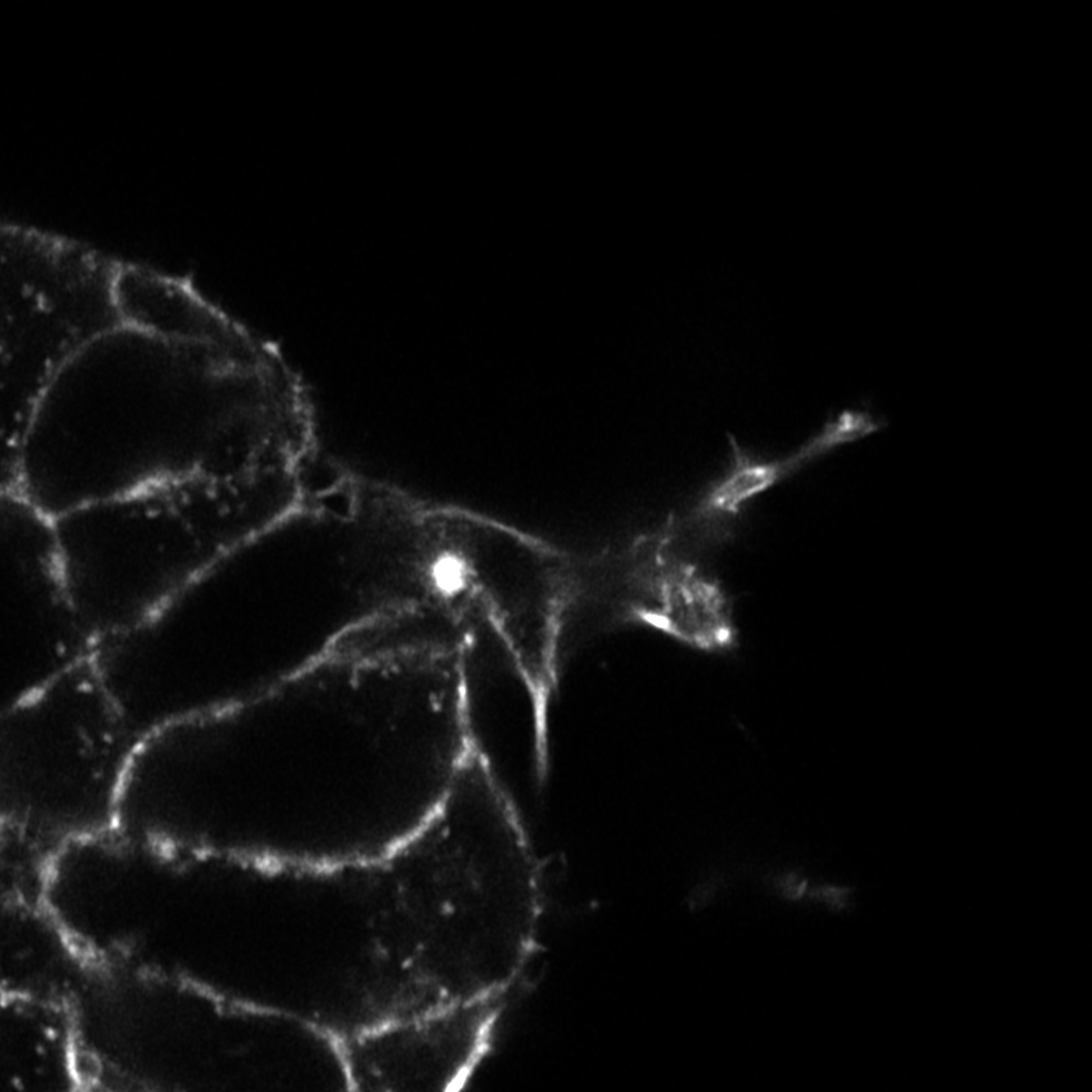

Supplement: Supplementary file 25 — Source Data for Figure 7 [file EMBJ-42-e113987-s018.zip › Figure 7/7C/Z=35 Actin for pFAK.tif]

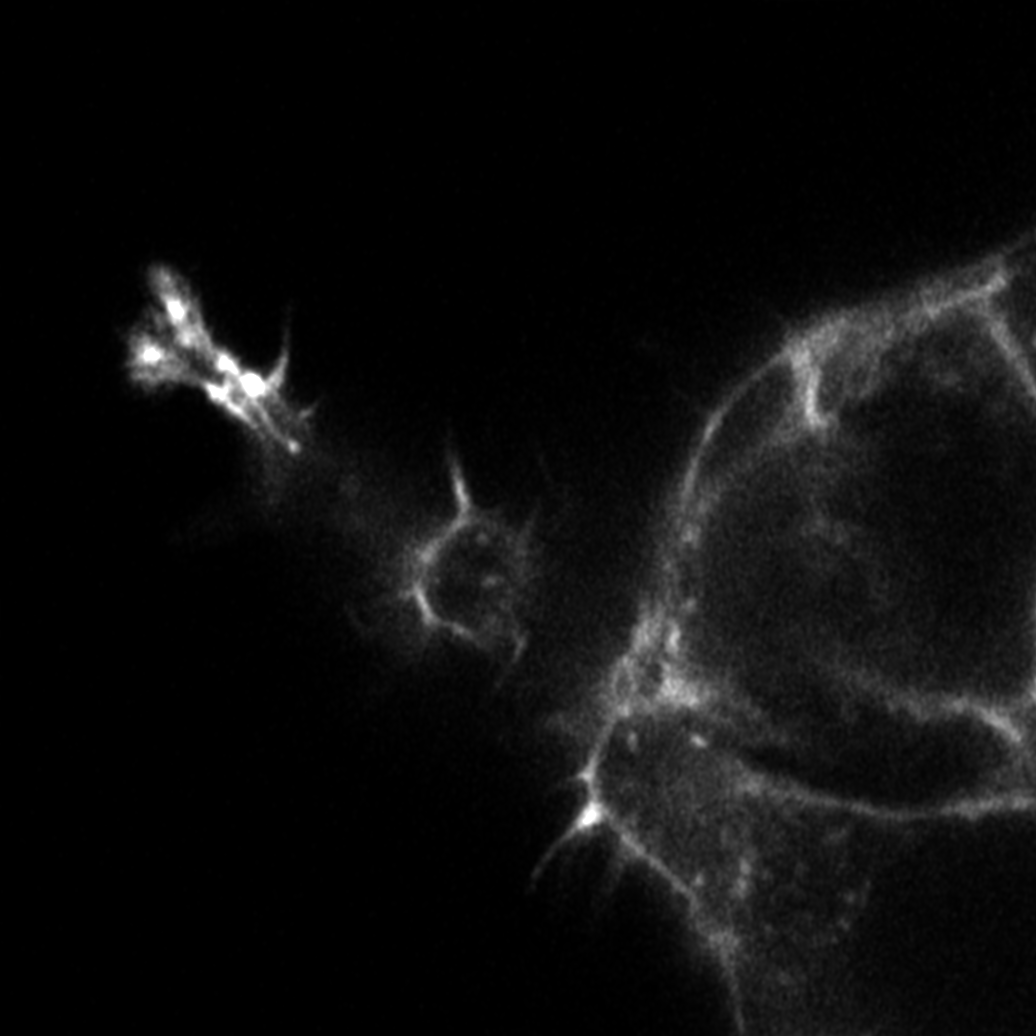

Supplement: Supplementary file 25 — Source Data for Figure 7 [file EMBJ-42-e113987-s018.zip › Figure 7/7C/Z=29 Actin for pSRC.tif]

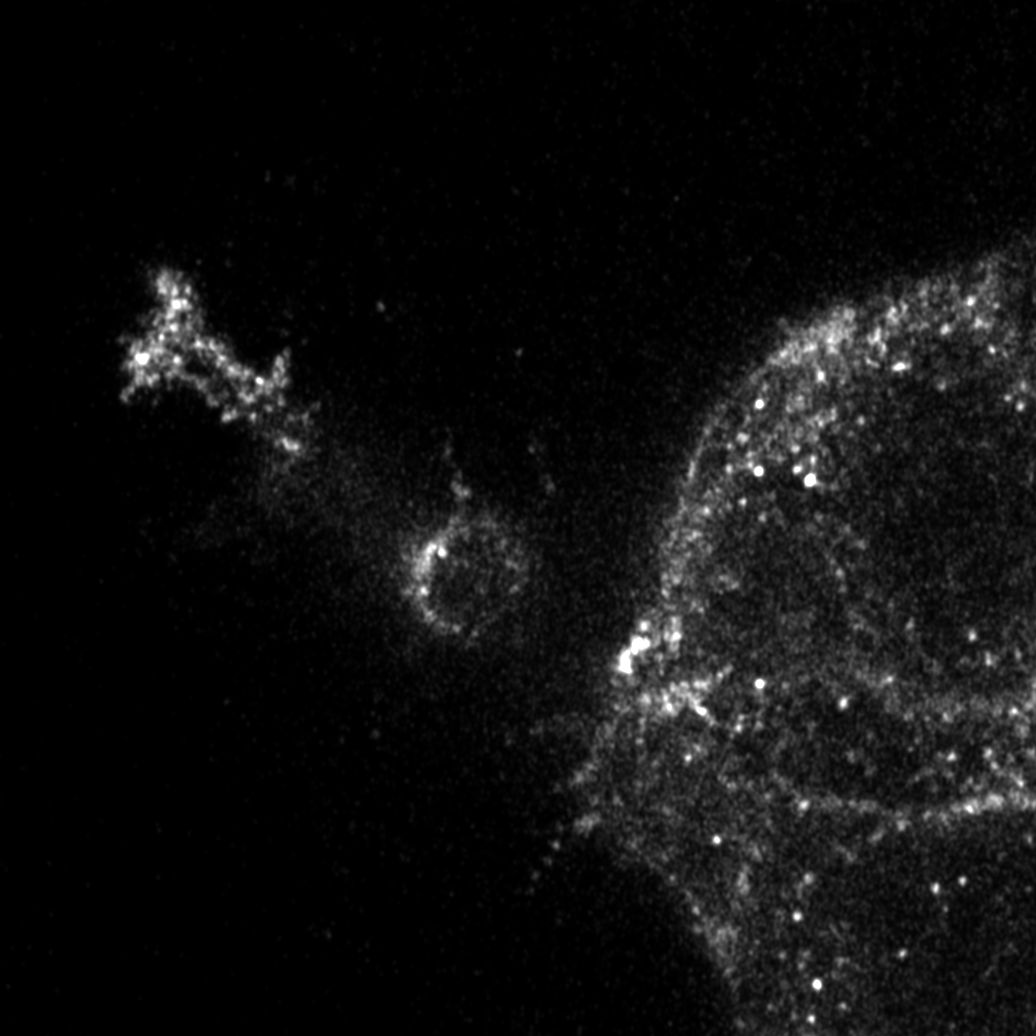

Supplement: Supplementary file 25 — Source Data for Figure 7 [file EMBJ-42-e113987-s018.zip › Figure 7/7C/Z=29 pSRC.tif]

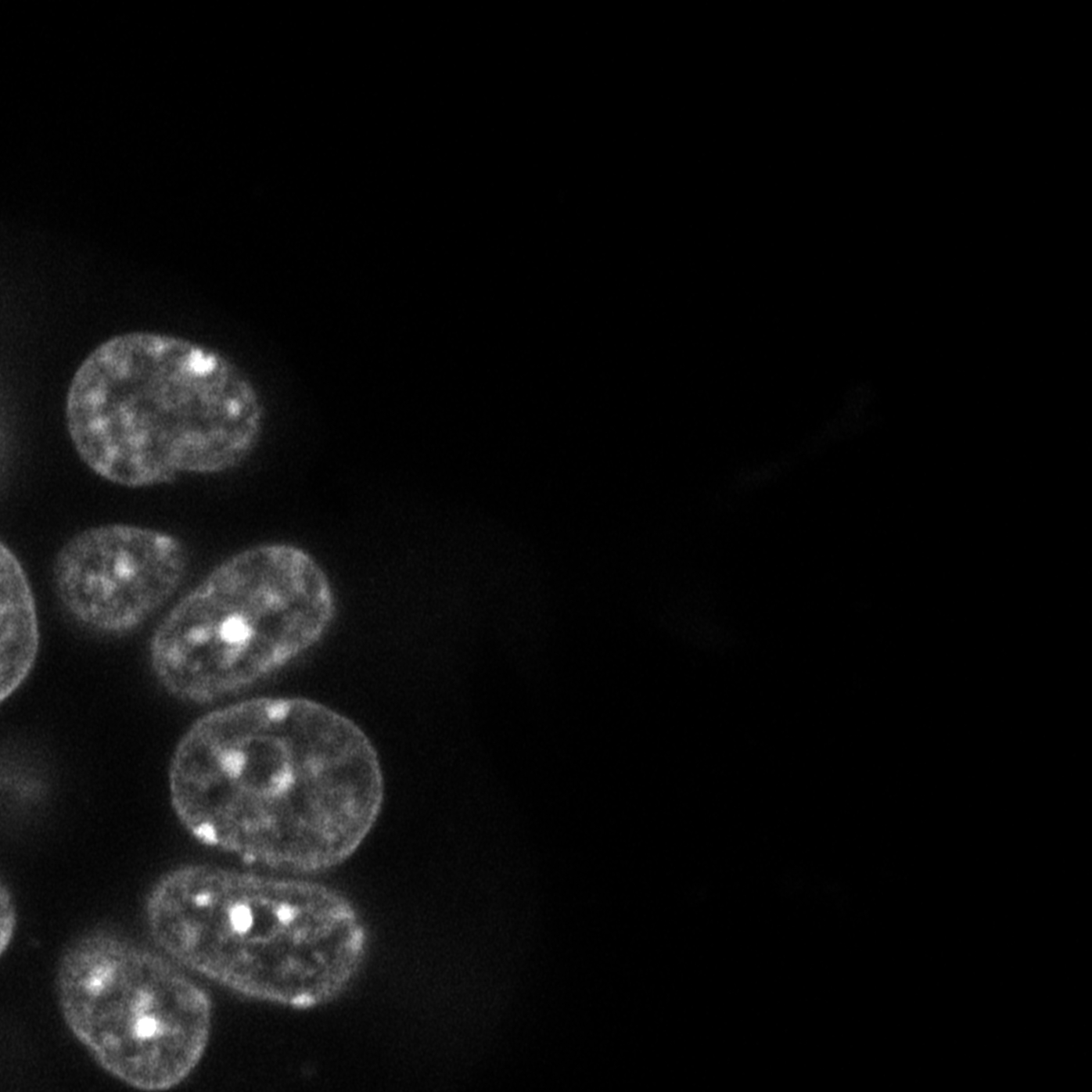

Supplement: Supplementary file 25 — Source Data for Figure 7 [file EMBJ-42-e113987-s018.zip › Figure 7/7C/Z=35 Hoescht for pFAK.tif]

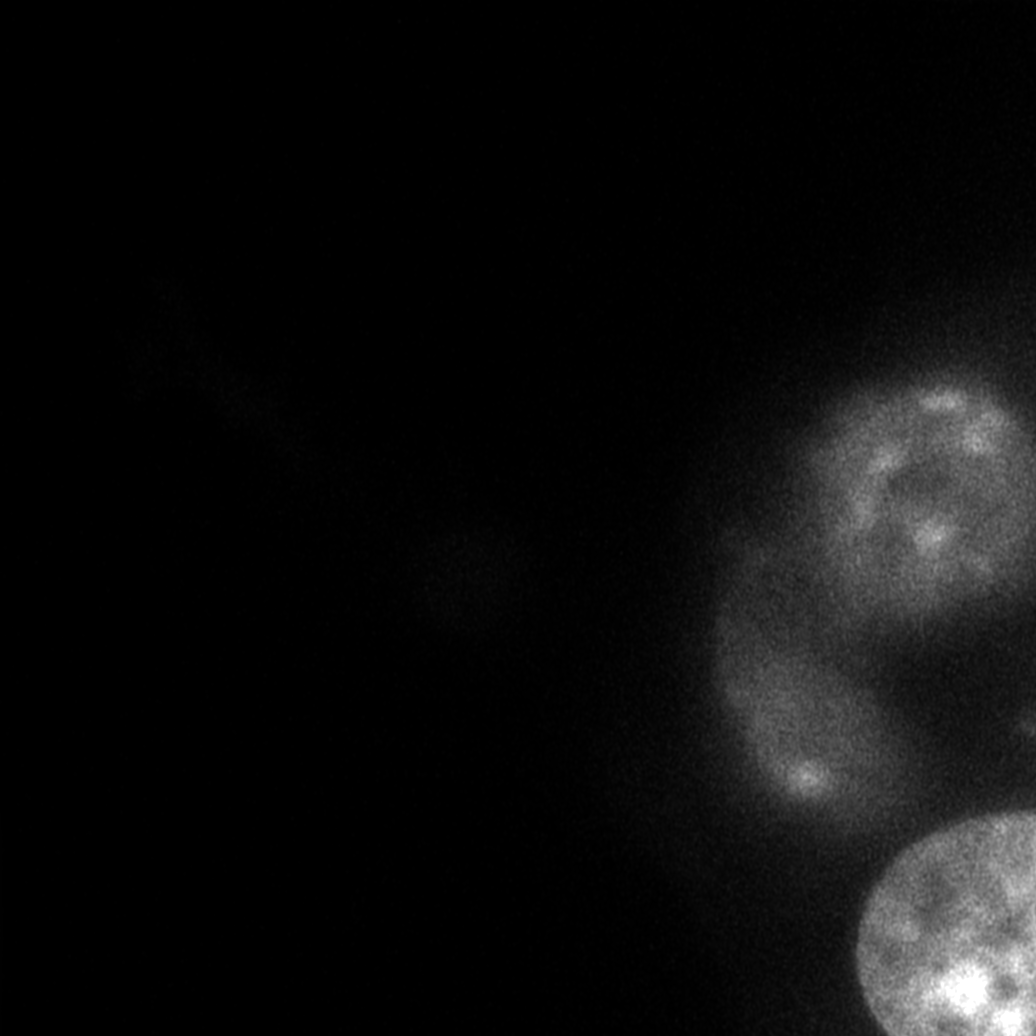

Supplement: Supplementary file 25 — Source Data for Figure 7 [file EMBJ-42-e113987-s018.zip › Figure 7/7C/Z=29 Hoescht for pSRC.tif]

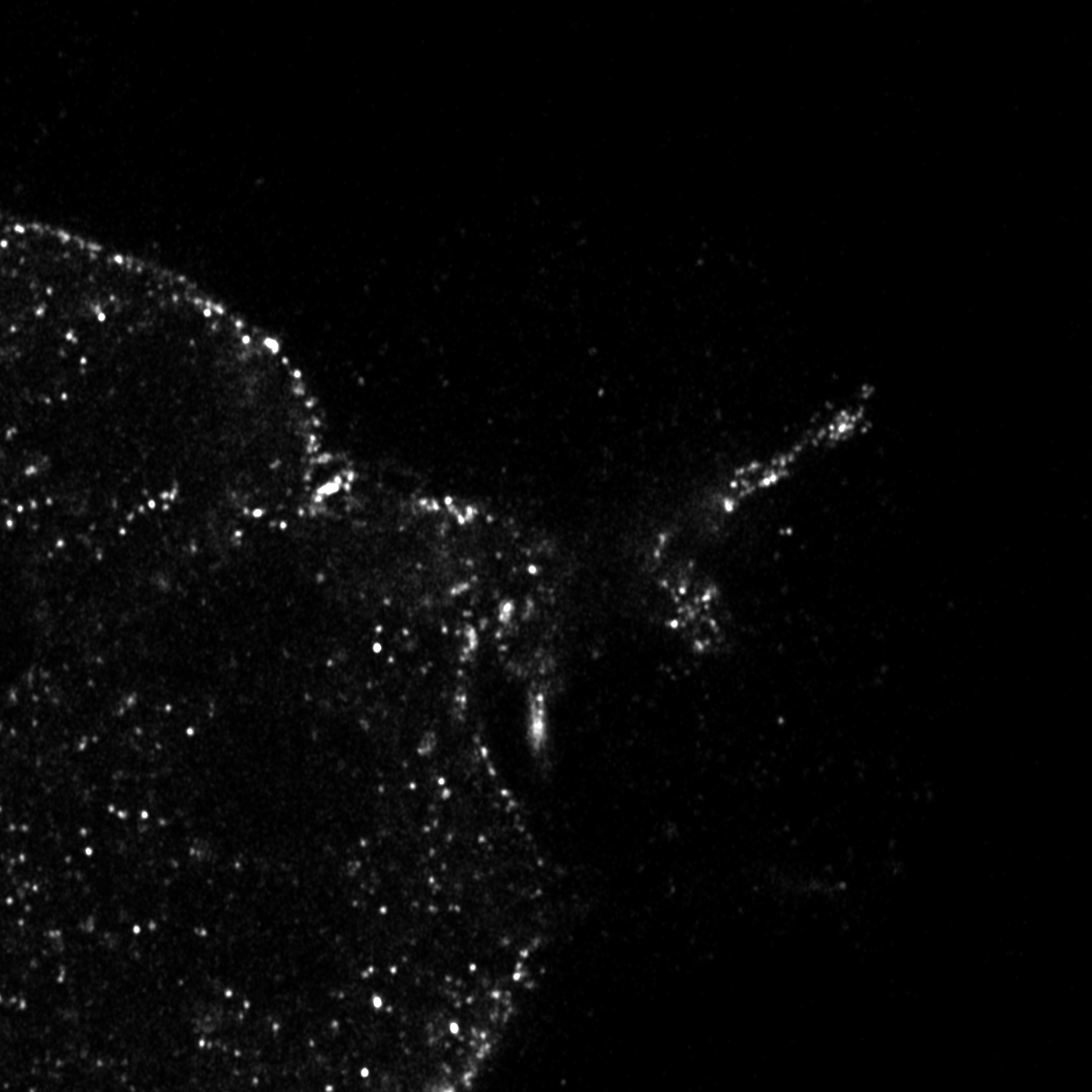

Supplement: Supplementary file 25 — Source Data for Figure 7 [file EMBJ-42-e113987-s018.zip › Figure 7/7C/Z=35 pFAK.tif]

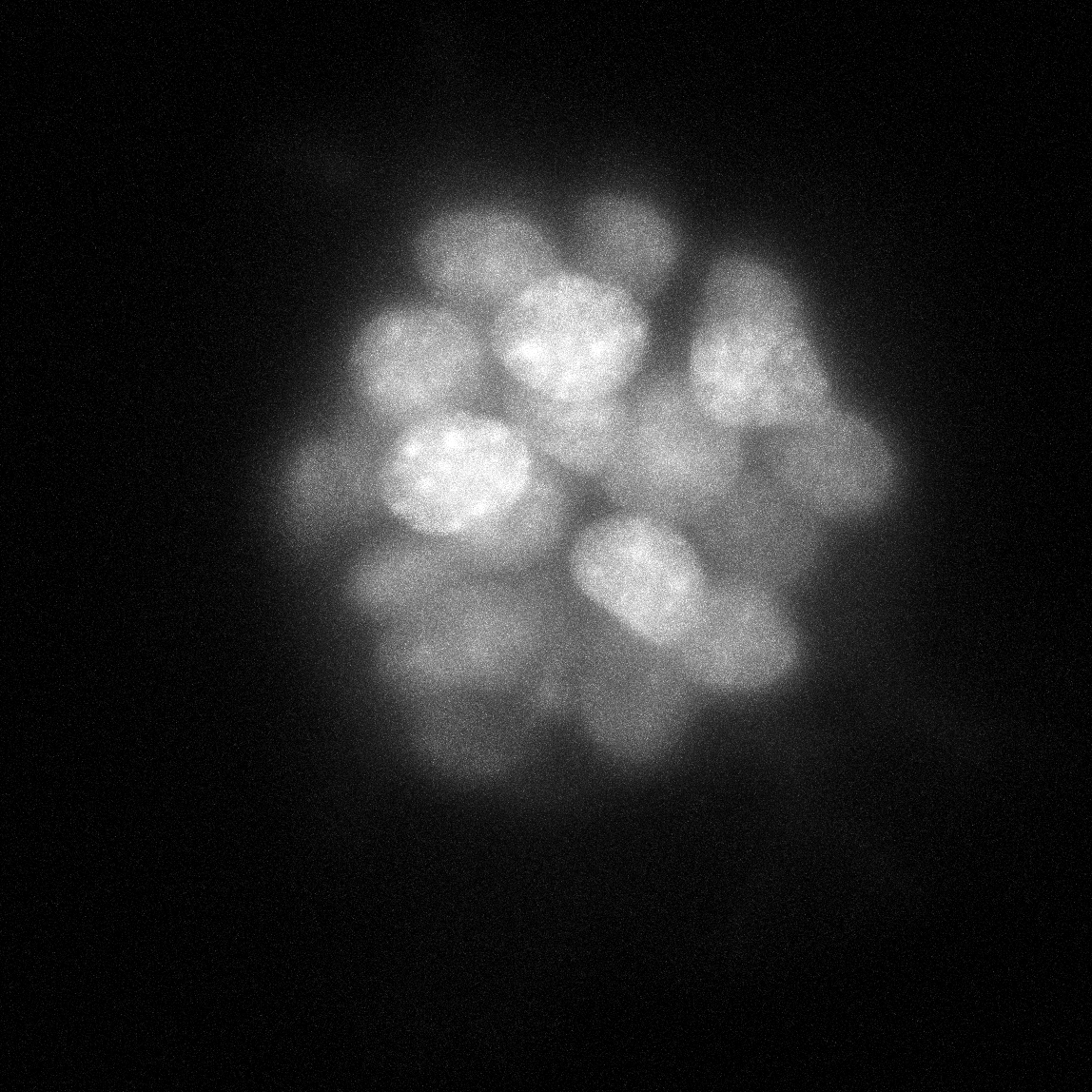

Supplement: Supplementary file 25 — Source Data for Figure 7 [file EMBJ-42-e113987-s018.zip › Figure 7/7A/Hoescht MAX for alpha5 itg.tif]

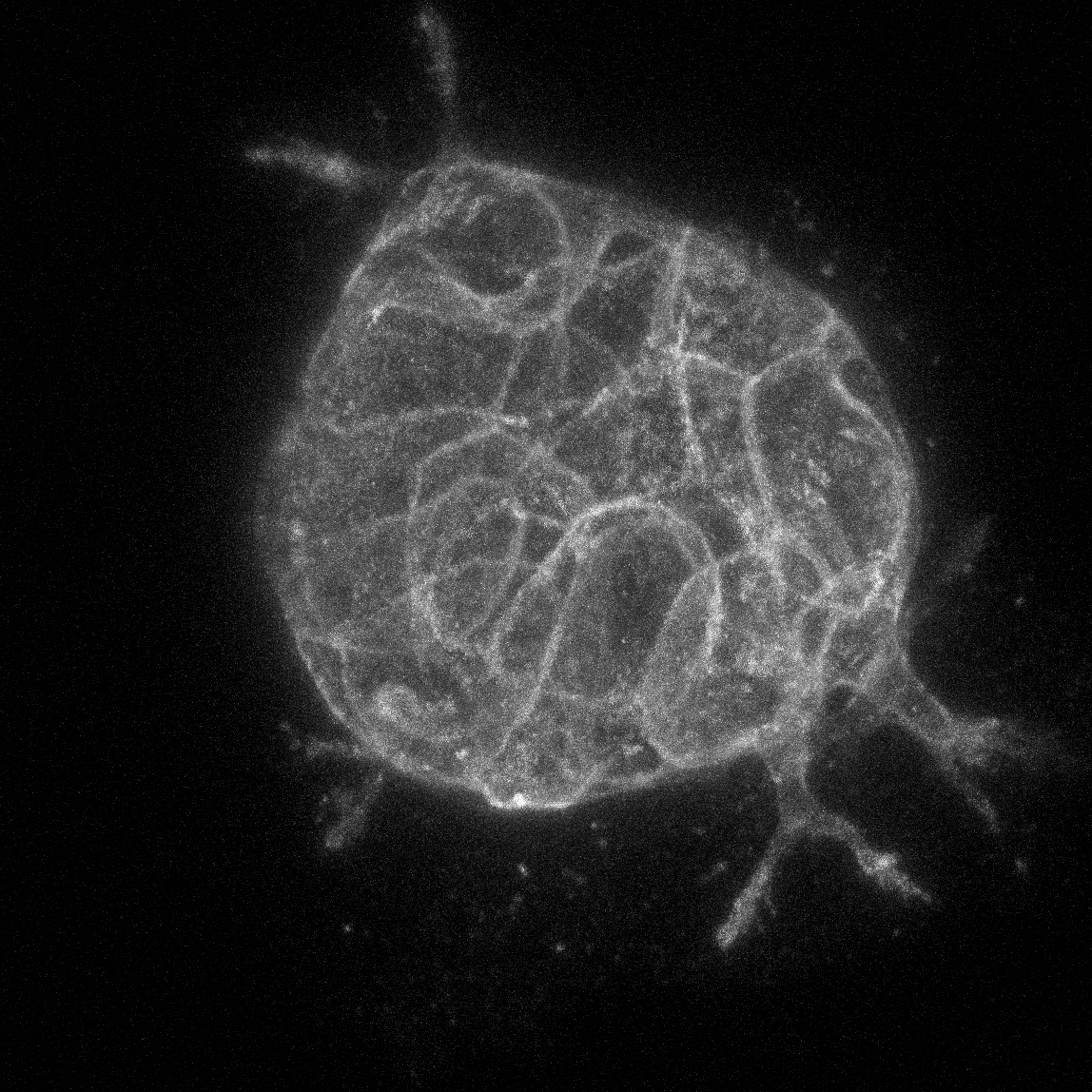

Supplement: Supplementary file 25 — Source Data for Figure 7 [file EMBJ-42-e113987-s018.zip › Figure 7/7A/Alpha5 itg MAX.tif]

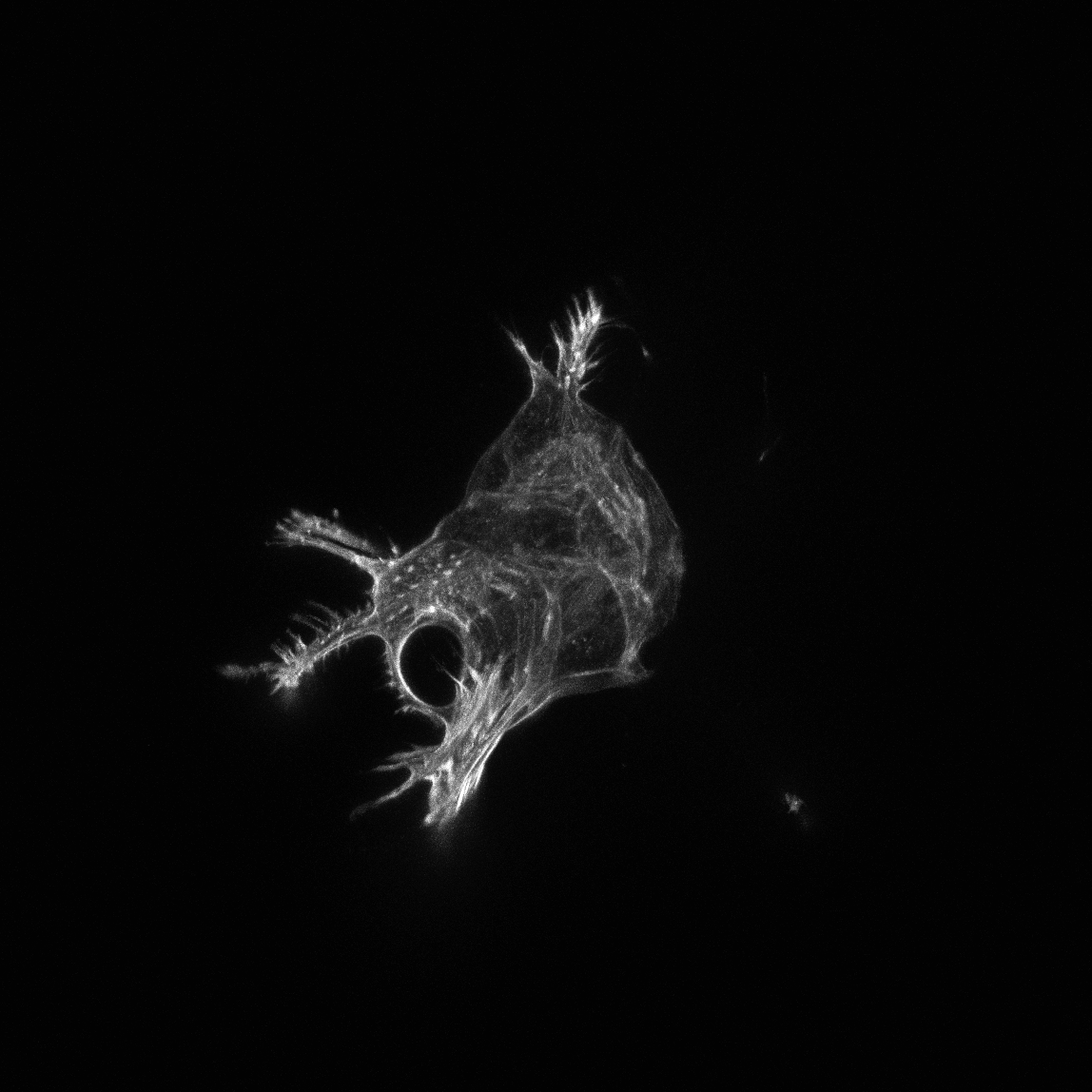

Supplement: Supplementary file 25 — Source Data for Figure 7 [file EMBJ-42-e113987-s018.zip › Figure 7/7A/Actin MAX FOR beta1 itg.tif]

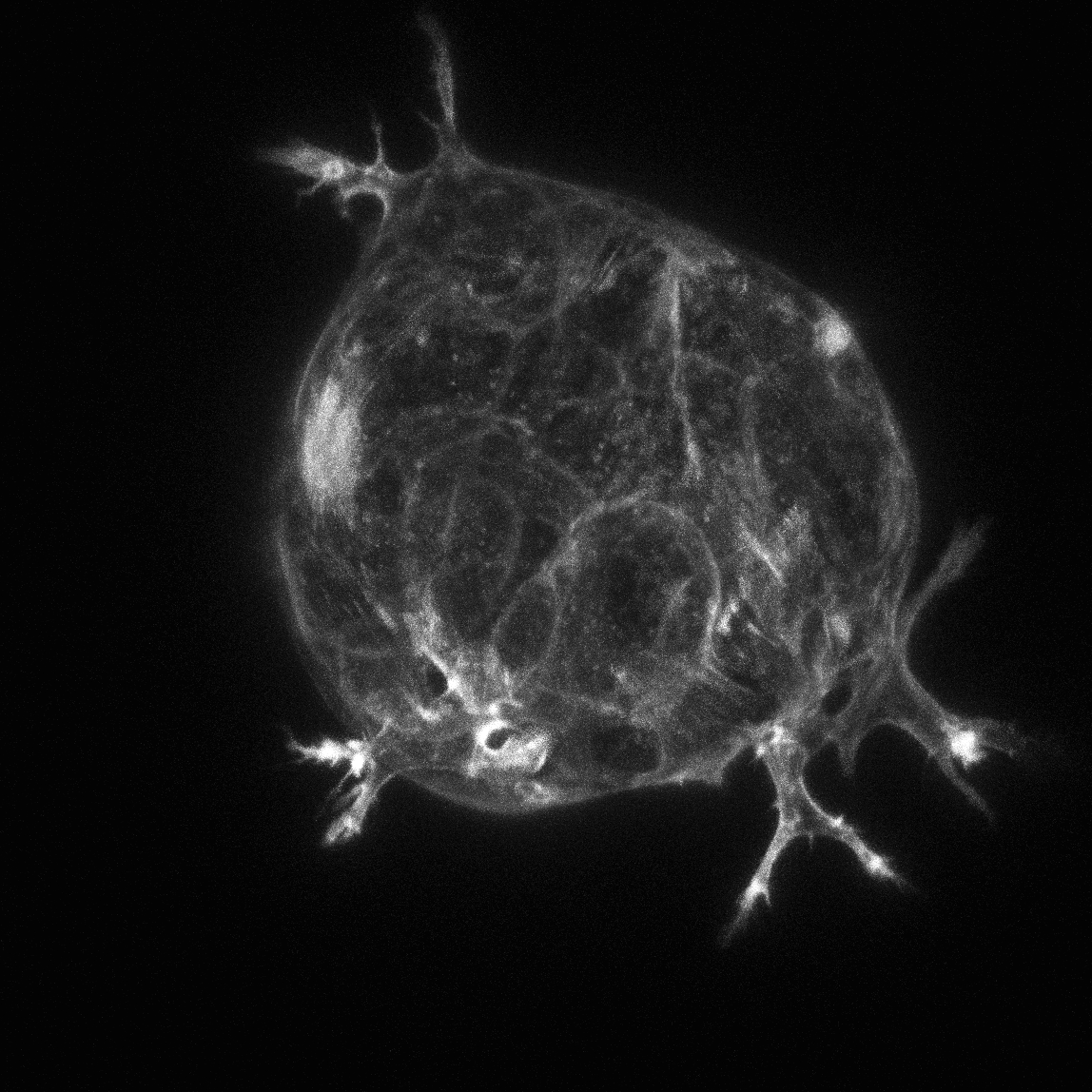

Supplement: Supplementary file 25 — Source Data for Figure 7 [file EMBJ-42-e113987-s018.zip › Figure 7/7A/Actin MAX for alpha5 itg.tif]

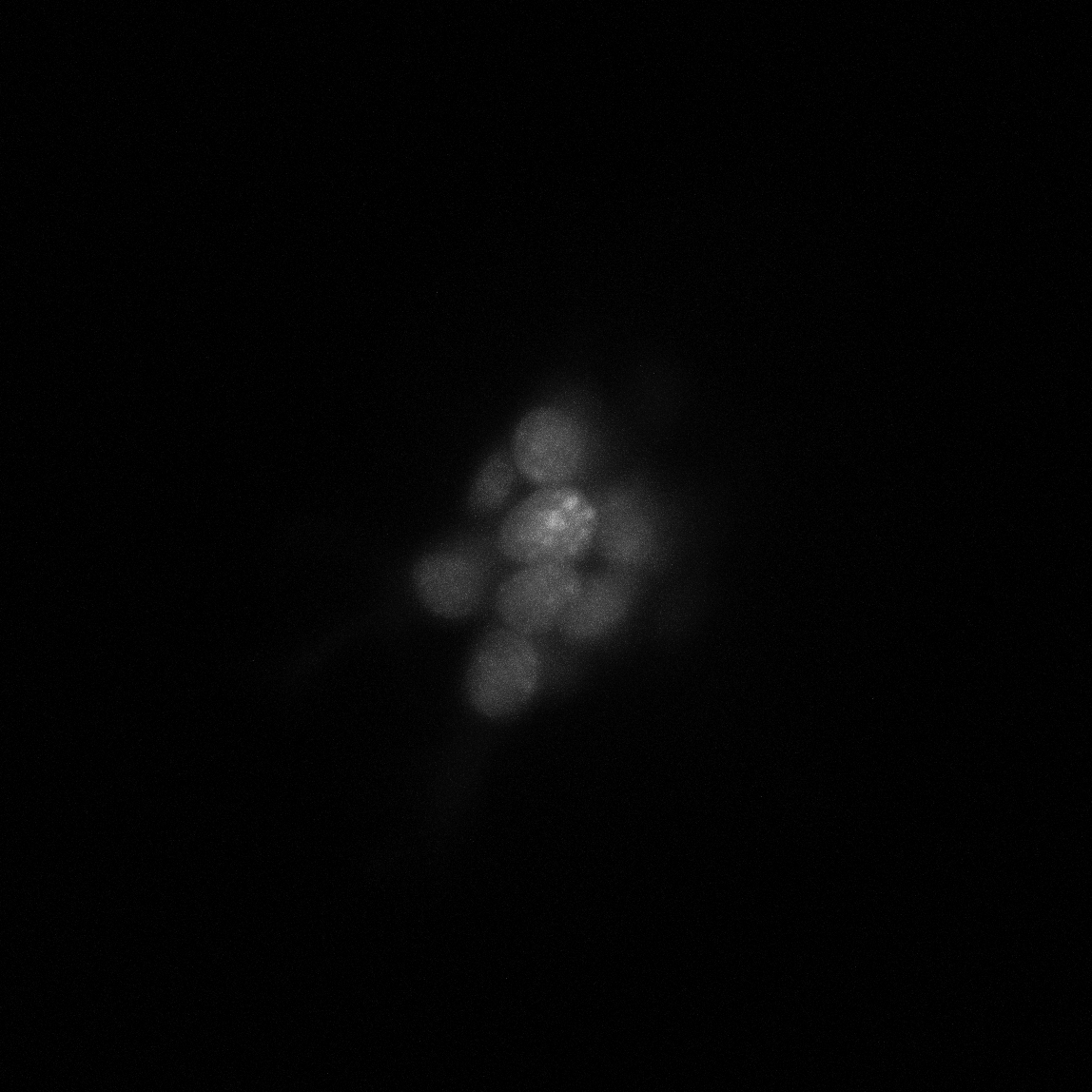

Supplement: Supplementary file 25 — Source Data for Figure 7 [file EMBJ-42-e113987-s018.zip › Figure 7/7A/Beta1 Hoescht MAX.tif]

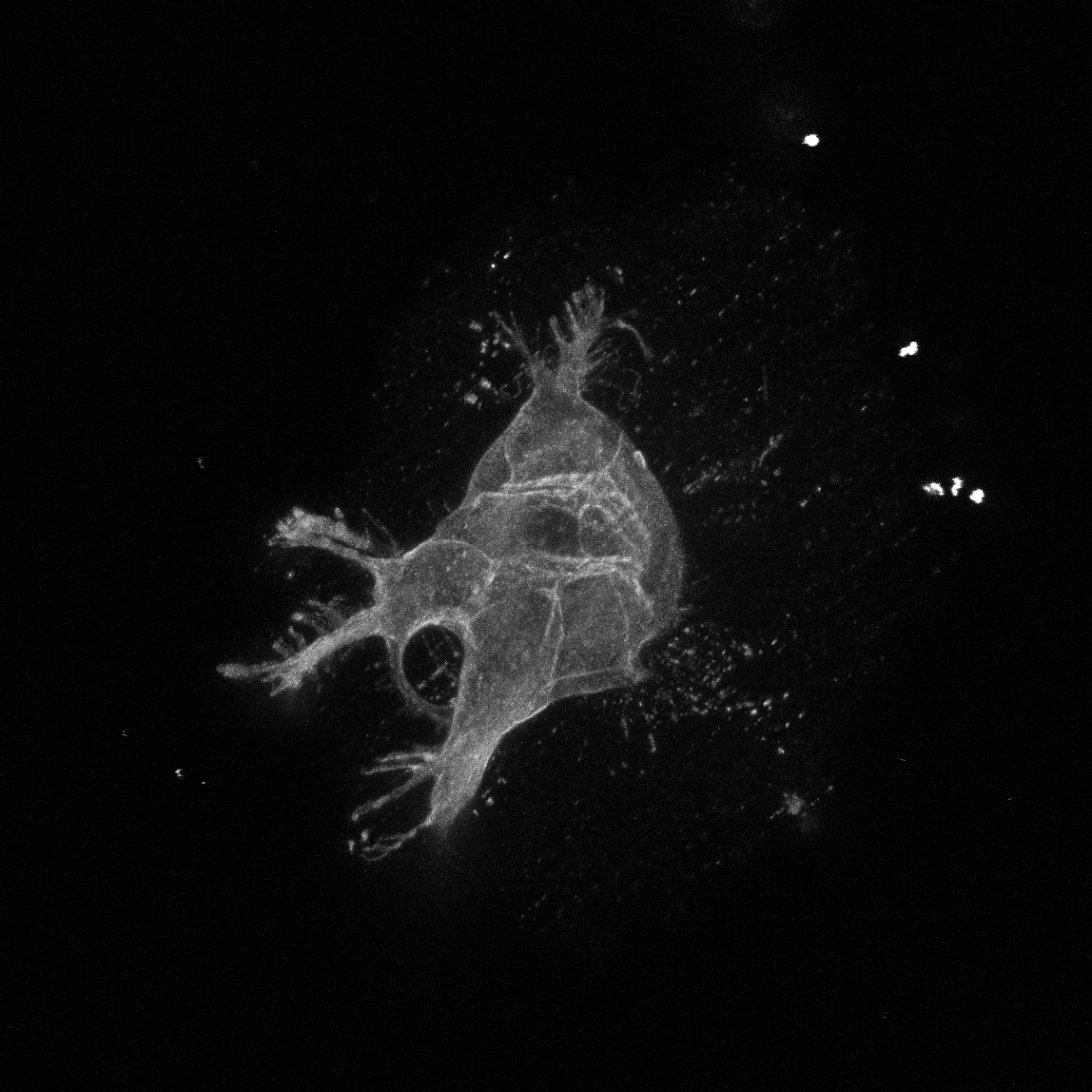

Supplement: Supplementary file 25 — Source Data for Figure 7 [file EMBJ-42-e113987-s018.zip › Figure 7/7A/Beta1 itg MAX.tif]
