# Supplementary material for: PTEN deficiency exposes a requirement for an ARF GTPase module for integrin‐dependent invasion in ovarian cancer
Source: EMBO J. 2023 Aug 14;42(18):e113987. doi: 10.15252/embj.2023113987 (PMC10505920; doi:10.15252/embj.2023113987)
Supplement: Supplementary file 26 [file EMBJ-42-e113987-s016.pdf]

THE EMBO JOURNAL

PTEN deficiency exposes a requirement for an ARF GTPase module for integrin-dependent invasion in ovarian cancer.

Konstantina Nikolatou, Emma Sandilands, Alvaro Roman-Fernandez, Erin Cumming, Eva Freckmann, Sergio Lilla, Lori Buetow, Lynn McGarry, Matthew Neilson, Robin Shaw, David Strachan, Crispin Miller, Danny Huang, Ian McNeish, James C. Norman, Sara Zanivan, and David Bryant
DOI: 10.15252/embj.2023113987

Corresponding author(s): David Bryant (David.Bryant@glasgow.ac.uk)

Review Timeline:

Transfer from Review Commons	9th Mar 23
Editorial Decision:	22nd Mar 23
Revision Received:	19th May 23
Editorial Decision:	7th Jul 23
Revision Received:	13th Jul 23
Accepted:	19th Jul 23

Editor: Daniel Klimmeck

Transaction Report:

(Note: With the exception of the correction of typographical or spelling errors that could be a source of ambiguity, letters and reports are not edited. Depending on transfer agreements, referee reports obtained elsewhere may or may not be included in this compilation. Referee reports are anonymous unless the Referee chooses to sign their reports.)

Review #1

1. Evidence, reproducibility and clarity:

Evidence, reproducibility and clarity (Required)

This paper by Konstantinou et al aims at deciphering the mechanisms by which PTEN loss could be driving poorer prognosis in patients. The authors use their great high-throughput 3D screening method coupled to an unbiased proteomic method and a CRISPR screen to uncover a new pro-invasive axis driving collective invasion of high-grade serous ovarian carcinoma (HGSOC) cells. Overall, this is a very impressive study, very well done and controlled with rigorous statistical analyses that uses sophisticated methods to convincingly show that the CYTH2-ARF6-AGAP1-ITGA6/ITGB1 module is required for the pro-invasive effect of PTEN depletion and discriminates patients with poorest prognosis.

Major comments

Below are listed all the claims that, in my opinion, are not adequately supported by the data.

1. Choice of the cell line: More justification on the use of the ID8 cell line and on the p53 deletion is needed. The authors need to clearly state that most p53 mutations in ovarian cancer are missense mutations that lead to a strong accumulation of a p53 protein devoid of transcriptional activity. Nevertheless, it seems that p53 mutations are not associated to differences in patient survival. Hence the choice of studying PTEN loss in the complete absence of p53, a situation that does not mirror the clinical situation, needs to be explained. Moreover, the in vivo experiments already performed in the literature mentioned in the discussion should be mentioned in the introduction to provide more context and physiological relevance to this study (especially regarding the special focus on the p53 null/ dKO cells throughout the study).
2. "Therefore, PTEN loss in ovarian cancer, particularly at the protein level, occurs in the tumour epithelium and is associated with upregulated AKT signalling and poor overall survival". This claim is an over-interpretation and over-generalisation of the data presented. I appreciate the honesty of the authors in showing all the ovarian datasets that are available and highlight the discrepancies in expression of the proteins they study in stroma and epithelium. I think the way to present these data in the text without over-interpreting and generalizing would be to show that there is a clear epithelial-specific downregulation of PTEN at the mRNA level. Most likely due to the

contribution to other cell types in the stroma, only 3 out of 5 bulk tumour mRNA datasets show a tumour specific downregulation of PTEN and no association with survival based on a median split of PTEN mRNA expression. Nevertheless, although there is no direct correlation between PTEN mRNA and protein levels, patients with low PTEN protein levels have poorer survival that is associated to an upregulation of Akt signalling. This allows to have a clearer conclusion, based solely on the protein data presented and no over-generalisation using the mRNA data. This, to me, makes a stronger case for studying PTEN loss in ovarian cancer and is fully supported by the data presented.
3. PTEN loss induces modest effects in 2D culture. The authors make claims regarding the fact that some of the phenotypes they look at happen after PTEN depletion alone or in combination with p53 loss and are more prominent in 3D vs 2D. Many of these are insufficiently backed up by data. A few key experiments are also only performed in 2D and should be done in 3D. Finally, some clarifications if the role of PTEN is most prominent on either collective, ECM-induced or 3D-dependent invasion.

First, the authors claim that PTEN loss alone (i.e. without p53 deletion) leads to changes in Akt signalling. Supp fig 1H clearly shows that there is no significant increase in Akt activation, although there seems to be one in the Western Blot (WB) presented in supp fig 1G. There is a clear, significant increase in the Akt activation in all the PTEN KO clones when in association with p53 loss though. This claim is hence not backed up by data and the conclusion seems to be that the effect on Akt signalling requires both deletion of p53 and PTEN.

It will be interesting to see a quantification of the pS473-Akt staining (supp fig S1J), as it seems from these images that pAkt is preferentially found on rounded cells. It should also be performed in 3D conditions to see if there is an enrichment at invasive tips and back-up the invasion data.

Arf6 is recruited to the invasive tips of cells invading a 2D wound (fig4D). How do the authors reconcile the fact that all the machinery required for 3D invasion is present but that PTEN loss has a modest effect on cells in 2D? If the wound assay was done on glass, it should be done again on ECM coated glass to see if it recapitulates the effects seen in 3D. This experiment will help deconvolute if the effect of PTEN loss is more linked to collective behaviour than 3D organization or presence of ECM.

The recycling assays are all done in 2D, condition under which the authors claim that the PTEN phenotype is weakest. Although I understand that it is not possible to do this assay in 3D, its contribution to elucidating the mechanism by which integrins participate in the PTEN loss invasive phenotype is not clear. The requirement of

integrins relies on the data showing that ITGB1 KO results in no collagen4-positive basement membrane of the cysts and greatly impaired invasion. Experiments looking at the integrin localisation would be helpful: can an enrichment at the invasive tips can be seen? Are ITGA6 and/or ITGB1 repartitions homogeneous between the cysts membranes and the invasive tips? In my opinion the Src/FAK data is not enough to draw the conclusions of fig7I schematic.

4. Expression of AGAP1 isoforms do not alter ARF6 levels. Data in fig 6C, D show a significant downregulation of Arf6 and Akt signalling after expression of AGAP1S. Can the authors clarify what they mean?
5. Arf6 is not modulated in the different cell lines: data in fig4B (far right graph) and supp fig 4B, J seem to indicate otherwise. Can the authors clarify what they mean?
6. Immunofluorescence panels without quantifications: Quantifications for the different stainings shown in fig3A; 4D, E; 5H; 7B and supp fig S1L, J; S3 need to be included to fully back the conclusions of the authors. Indeed, these images are used to draw conclusions and not only as illustrations.
7. Quantifications of invasion show that WT cysts become hyper-protrusive at around the half experiment mark (around 30-40hrs). Nevertheless, all movies or galleries show spherical cysts, which does not seem representative. Can the authors change this or explain why these images/movies were chosen?
8. Since it seems that the main effect of PTEN is to drive the localisation and intensity of recycling of Arf6 cargoes, it will be helpful to confirm that all the proteins involved in the Arf6 module be shown to be accumulated/present at the pro-invasive tips. Immunofluorescence stainings showing the presence of AGAP1 (could be done with the AGAP1S isoform that is mNeon-tagged), pS473-Akt, ITGB1 (active integrin if possible, otherwise total integrin), ITGA5, PI3K should be included if possible. A quantification comparing signal in the cysts and in the invasive tips should also be included to see if there is an accumulation to PIP3-enriched areas.
9. Data in fig5I convincingly show that PTEN loss induces a fragmented collagen4-positive basement membrane. The authors use this data to claim that this is one of the ways that PTEN could be driving invasion but no correlation between these structures and the hyper-protrusive phenotype is made. This experiment needs to be done to support this claim.

Minor comments

1. Data visualization: I think that the heatmap representation is overkill when only 2 or 3 conditions are presented. A graph showing the evolution of area or spherical/Hyper-protrusive phenotype proportions across time would be easier to read and more impactful: each genotype could be presented with a colour and the

spherical/hyper-protrusive phenotypes as either plain or dashed lanes across time. I understand that this representation allows for the stats to be done at each time points but they are generally pretty clear (especially for the PTEN KO or dKO phenotypes) and do not need to be done for each time point in my opinion. These heatmaps could be put in supplementary figures if the authors feel strongly about putting stats for each time points.

Fig supp S1M, fig 5I should be presented as a stacked histogram to improve readability and merged with fig supp S1K.

Displaying fold change as antilog rather than log values would be easier for the reader to realise the magnitude of the differences.

A bar graph would be easier to read than the matrix representation for fig 6B.

The way Area data is presented throughout to me makes it very difficult to understand what is going on. Could the authors at least give some explanations in figure legends. A curve graph displaying the evolution of the area across time would be easier to read and see the differences between conditions.
2. It is confusing that, in fig supp S1M, there is a significant decrease of the rounded phenotype after PTEN loss that is not associated to a significant change in another of the categories. Could the authors explain how?
3. One of the big differences of the PTEN KO cells seems their ability to invade through the matrigel bed and migration on the glass below (supp movie S2). From what I gather, these cysts would be considered out of focus and excluded from the analysis. Would it be possible that this would minimize some of the results? Would it be possible to include a quantification of this particular phenotype to confirm it is specific to PTEN KO cells?

In the same spirit, could the authors provide the percentage of non-classified cysts, to make sure that the same proportion of cysts is quantified across all different genotypes.
4. Can the authors clarify how a 0 fold change (in log value) in fig 2D can be highly significant?
5. Delta isoform of PI3K seems to have an effect on area in the middle of the experiment, but has no effect at all on invasion. Could the authors comment? Are these smaller cysts still as invasive? There might be an interesting uncoupling between proliferation and invasion there.
6. ITGB1 depletion seems to induce a downregulation of Akt protein. Is that right? Does it change Akt localisation? Is there a dose effect whereby there is not enough Akt protein to mediate invasion?

7. Stats should be added directly on the graphs for the recycling assays, doing a pairwise comparison of the different genotypes for each time points. Can the authors clarify what the t-32min quantification graphs adds (fig7E, supp fig S8G-I)? I would advise to remove them, as this data is already presented in the recycling assay graphs.
8. There is a substantial amount of typos and erroneous references to figures. I listed below the ones that I spotted and I encourage the authors to carefully check.

- a. there are some mistakes in referencing the number of cysts in supp table 1. There is for example no cysts experiments in Figure 1 but yet there are some references to figure 1 in supp table 1. Please correct it. I think it will be easier for the reader if the number of cysts quantified for each conditions was also indicated in the figure legends. Supp table 1 can still be included for readers that want additional details.
- b. comma missing page 3
- c. page 3 and 4: PI(3,4)2 means PI(3,4)P2? Can be shorten to PIP2 for ease of read and specify if it is another PIP2 specie otherwise
- d. define CYTH abbreviation: I suppose this is for cytohesin?
- e. fig1F-I: don't understand why TCGA.OV is specified on some but not all the graphs. It seems to me that all the data are from TCGA.OV? Makes it seems it is nit the case
- f. legend of fig1H, I: y axis is -Log10 values in 1I, not Log10 values
- g. page 6: dKO abbreviation is already specified above and should be used to avoid repetition and for ease of read
- h. supp fig S1D: missing legend for the second bar (after Wild Type)
- i. supp fig S1N: legend of the X-axis should be below the axis
- j. supp fig S1O: the numerotation of the X-axis needs to be below the line of the axis for ease of read, not above it
- k. legend of S2A: clones 1.12 and 1.15 are p53-/-;PTEN-/- and not PTEN-/-
- l. supp figS2C can the authors specify the different stages of matrigel (liquid or gel) that are used for the invasion assay, to make it easier for the non-specialist to understand what is going on. Please confirm that the 50% GFR matrigel makes a gel on top of the cells and fill in the wound to produce the 3D invasion assay setup.
- m. page 7: no parental cells are used in S3A, B only p53 null and p53 null and dKO. Please also specify what cells are being compared in the text
- n. description of arrow heads and colours need to be moved to figure legends and not in main text (page 7)
- o. fig 2D: the signification of the dot in the circles needs to be in the legends (since it is its first apparition in the manuscript). It only appears later on, in supp2A legend. Additional description of the matrices is necessary, as they contain a lot of information to digest to understand fully what is going on
- p. legend of fig3: error in figure reference: area data is D and not E, protrusive phenotypes are E and not F

- q. arrow missing in fig3B
- r. fig 3D,E, G, H: please indicate the cell line studied
- s. fig 3I: the different genotypes need to be stated on the galleries for clarity
- t. page 8: define Arf6-mNG in the text
- u. page 9: "<" symbol should be an alpha symbol
- v. fig 4A: indicate the cell line used on the figure
- w. supp fig S4E: why is it specified mouse-specific for the shArf6?
- x. 4H, I, J: indicate on the figure if these interactors are mostly unchanged, strong interactors or weak interactors for clarity
- y. legend of fig4H: "coloured spots underneath denote the protein complex that each interactor belongs (in J)" should indicate panel G and not J
- z. fig4I, J: are you sure of the legend for the fold change coloring? Log2 of 1 is a 0 fold change, i don't see how these could show any significant difference (i.e. some of the pale red circles are significant)
- aa. page 11: description of the assay (starting with "Machine learning classification of...") is very confusing, please clarify
- bb. page16: figure 4H should be 4I (PTEN-null specific association of Arf6 with ITGA5)
- cc. supp fig S5H-P: choose Tumour or cancer to homogeneise naming across the graphs
- dd. fig 5H: box are difficultly visible in green, change color to yellow or something more visible
- ee. page 13: Fig6E, F should also refer to 6G
- ff. LCM abbreviation on page 10 and 12 refers to LCMD? Otherwise please define it.

Optional suggestions

1. Choice of cell line: There is a high number of patients (around 9% according to (Cole et al. 2016)) that present the R248Q gain-of-function mutation. A recent study has shown that this mutant p53 protein is associated to an activation of Akt signalling and an increase of the intercellular trafficking of EGFR (Lai et al. 2021). Given that EGFR was also a hit in this screen, that is seems to have a central role in Arf6 cargoes (fig 4G), I think it would be a great addition to this study. It could hence cooperate with PTEN loss to drive strong, robust invasion.
2. Are MAPK involved in the PTEN KO pro-invasive phenotype? In particular Erk1/2, since EGFR is one of the PTEN loss induced Arf6 cargoes.

Reference

Cole, Alexander J., Trisha Dwight, Anthony J. Gill, Kristie-Ann Dickson, Ying Zhu, Adele Clarkson, Gregory B. Gard, et al. 2016. « Assessing Mutant P53 in Primary High-Grade Serous Ovarian Cancer Using Immunohistochemistry and Massively Parallel Sequencing ». Scientific Reports 6 (1): 26191. https://doi.org/10.1038/srep26191.

Lai, Zih-Yin, Kai-Yun Tsai, Shing-Jyh Chang, et Yung-Jen Chuang. 2021. « Gain-of-Function Mutant TP53 R248Q Overexpressed in Epithelial Ovarian Carcinoma Alters AKT-Dependent Regulation of Intercellular Trafficking in Responses to EGFR/MDM2 Inhibitor ». International Journal of Molecular Sciences 22 (16): 8784. https://doi.org/10.3390/ijms22168784.

2. Significance:

Significance (Required)

It has only been recently appreciated that PTEN loss is a driver in ovarian cancer (Martins et al. 2020) but no studies to data have aimed at understanding the mechanisms. This study is hence the first to propose one and as such provides a very valuable advance for researchers interested in ovarian cancer. The authors also propose that the CYTH2-ARF6-AGAP1 high mRNA be used as a signature of worsen prognosis. This hence paves the way to better understanding and stratify patients with ovarian cancers.

One of the main difference after PTEN loss is the accumulation of PIP3 in pro-invasive tips that correlates with the recruitment of Arf6 to these tips. The authors have developed a very powerful automated quantification pipeline to follow the behaviour of cysts grown in 3D that they have coupled to an unbiased proteomic method to identify interactors and a CRISPR screen to test their functional relevance. This is clearly the strongest aspect of the paper that allows them to gather very robust data and identify the machinery driving invasion in PTEN KO cells. The authors' model claims that this in turns recruit the Arf6 machinery, composed of CYTH2 2G (the only CYTH2 isoform correlated to a poorer prognosis, preferentially binding PIP3) and AGAP1 that leads to a local increase in active integrin recycling that mediates the more invasive phenotype of PTEN depleted cells. It is rightfully mentioned in the discussion that PTEN depletion only leads to a modest change in Arf6 interactors, and that most likely PTEN loss acts by locally directing the Arf6 machinery to the invasive tips. Indeed, the authors convincingly show that Arf6,

AGAP1 and ITGB1 are required for the formation of these invasive protrusions.

The limitation of this study is the combination of 2D and 3D experiments to drive general conclusions on the mechanism. These are listed in the previous section. Another big limitation, in my opinion, is the choice of the cell model: indeed, nearly all patients present a vast increase in the amount of the p53 protein present due to a very large number of mutations that in most cases prevent its binding to DNA. Throughout this paper the authors have used a p53 null cell line that expresses no p53 protein. This is not compatible with the clinical situation. Moreover, since p53 also present frequent gain-of-function mutations that have been shown to be associated to an increase of Akt signalling and intercellular trafficking of EGFR. Studying the implication of the Arf6 module identified here in a context of p53 WT or mutant protein overexpression would be of great interest.

Reference

Martins, Filipe Correia, Dominique-Laurent Couturier, Anna Paterson, Anthony N. Karnezis, Christine Chow, Tayyebeh M. Nazeran, Adekunle Odunsi, et al. 2020. « Clinical and Pathological Associations of PTEN Expression in Ovarian Cancer: A Multicentre Study from the Ovarian Tumour Tissue Analysis Consortium ». British Journal of Cancer 123 (5): 793-802. https://doi.org/10.1038/s41416-020-0900-0.

3. How much time do you estimate the authors will need to complete the suggested revisions:

Estimated time to Complete Revisions (Required)

(Decision Recommendation)

Between 3 and 6 months

4. *Review Commons* values the work of reviewers and encourages them to get credit for their work. Select 'Yes' below to register your reviewing activity at Web of Science Reviewer Recognition Service (formerly Publons); note that the content of your review will not be visible on Web of Science.

Web of Science Reviewer Recognition

Yes

Review #2

1. Evidence, reproducibility and clarity:

Evidence, reproducibility and clarity (Required)

The authors have conducted a study of the molecular requirements for cancer invasion that is stunning in its thoroughness and depth and breadth of its molecular analysis. The writing is exceptionally precise though also very dense (see below). The molecular model proposed is that PTEN loss (in a p53 null background) leads to reliance upon ARF6 for invasion, with regulation through interactions with AGAP1 and beta1-integrin and it is convincingly demonstrated. They focus on interpreting the consequences of genetic and pharmacologic manipulations in a cell line, using a series of 2D and 3D assays. The phenotypes are more prominent in 3D assays.

Concerns and Suggestions:

1. There is a disconnect between the essentially complete loss of protrusions and invasion in 3D (e.g. 4A) and the reduction in magnitude of protrusive invasion but the continued presence of elongated cells with protrusions in 2D (e.g. S4C). This discrepancy is present in a couple of comparisons and is glossed over in quick callouts to many figure panels.
2. Once a journal has been identified, it would be wise for the editor to allow some flexibility in word limit to enable some very dense sections to be expanded slightly to guide the reader through the experiments and results more clearly. For example, in the section "ARF6 regulates active integrin pools...", there are callouts like (Fig. 7C,E; S8A-C; G-I) and then (Fig. 7D,E; Fig. S8E-F, H-I). It takes a lot of time to unpack these different experimental claims based on a single sentence.
3. The patient data on CYTH2 and its relationship to survival is modestly convincing.
4. Very minor- search on %- there are a few inconsistencies in terms of spaces and commas vs. periods. The Methods also have some inconsistencies in terms of spaces between numbers and units or numbers and degrees Celsius. References are also in a different font. Overall it was extremely carefully written though (just dense).

2. Significance:

Significance (Required)

One limitation of the experimental design is that the depth of molecular analysis in vitro comes at the expense of any in vivo validation, which the authors acknowledge in the Discussion. They attempt to make similar points using analysis of patient survival data from public databases but these analyses generally yielded small magnitude differences. The main audience for this study is likely to be cell biologists interested in cell migration, cell-ECM adhesion, cancer invasion, and GTPases. I don't see any need for new experiments- what can be done has been done and then some. I do think that it would benefit readers if the text could be made less dense.

3. How much time do you estimate the authors will need to complete the suggested revisions:

Estimated time to Complete Revisions (Required)

(Decision Recommendation)

Less than 1 month

4. *Review Commons* values the work of reviewers and encourages them to get credit for their work. Select 'Yes' below to register your reviewing activity at Web of Science Reviewer Recognition Service (formerly Publons); note that the content of your review will not be visible on Web of Science.

Web of Science Reviewer Recognition

Yes

Review #3

1. Evidence, reproducibility and clarity:

Evidence, reproducibility and clarity (Required)

Summary:

Using a murine HGSOC 3D cell model, in combination with analysis of human ovarian cancer datasets, the authors uncover a CYTH2-ARF6-AGAP1 signaling module regulated by PTEN and identify a biomarker for tumor invasion and targeted therapy.

Major comments:

The findings of this study are significant as they reveal a critical signaling module that controls tumor invasion by mediating tumor cell interaction with the extracellular matrix. The experiments are properly designed, and the data are well presented. The conclusions are appropriate and supported by the data. The limitation of the study has also been discussed properly.

One suggestion regarding the survival analysis in Fig. 6 and 7.
The authors noted that the CYTH2-ARF6-AGAP1 module is not specifically or only induced in Pten-null contexts, but rather that Pten-null cells become more dependent on the module for enacting the invasive phenotype. Based on this, it would be interesting to evaluate how the PTEN status impacts the survival difference by integrating the PTEN genomic status (WT versus mutation) or its expression level (protein or mRNA) into the survival analysis of patient cohorts in Fig. 6 and Fig. 7.

Referees cross-commenting

Gene deletion and mutation may elicit different functional outcomes. I therefore agree with Reviewer #1 that "the choice of studying PTEN loss in the complete absence of p53, a situation that does not mirror the clinical situation, needs to be explained".

2. Significance:

Significance (Required)

The model used and data presented in this study are of significance for both scientific discovery and clinical application, which will interest the broad audience in both basic and clinical research.

3. How much time do you estimate the authors will need to complete the suggested revisions:

Estimated time to Complete Revisions (Required)

(Decision Recommendation)

Less than 1 month

4. *Review Commons* values the work of reviewers and encourages them to get credit for their work. Select 'Yes' below to register your reviewing activity at Web of Science Reviewer Recognition Service (formerly Publons); note that the content of your review will not be visible on Web of Science.

Web of Science Reviewer Recognition

Yes

Revision Plan

Manuscript number: RC-2022-01776
Corresponding author(s): David Bryant

1. General Statements [optional]

We describe an ARF6 GTPase module that controls integrin recycling to drive invasion in PTEN-null Ovarian Cancer (OC). We used high-throughput, time-lapse imaging and machine learning to characterise spheroid behaviours from a series of cell lines modelling common genetic lesions in OC patients. We identified that PTEN loss was associated with increased invasion, the formation of invasive protrusions enriched for the PTEN substrate $PI(3,4,5)P_3$, and enhanced recycling of integrins in an ARF6-dependent matter. We utilised Mass Spectrometry proteomics and unbiased labelling to investigate the interactome of ARF6, identifying a single ARF GAP (AGAP1) and a single ARF GEF (CYTH2). Importantly, this ARF6-AGAP1-CYTH2 modality was associated with poor clinical outcome in patients.

We thank all Reviewers for their highly complementary assessment of our manuscript, describing our paper as a *"very impressive study, very well done and controlled with rigorous statistical analyses that uses sophisticated methods"*, a study that is *"stunning in its thoroughness and depth and breadth of its molecular analysis"*, with *"experiments are properly designed, and the data are well presented. The conclusions are appropriate and supported by the data".* Finally, we would like to thank the reviewers for appreciating that our results are ***"of significance for both scientific discovery and clinical application, which will interest the broad audience in both basic and clinical research".***

2. Description of the planned revisions

Insert here a point-by-point reply that explains what revisions, additional experimentations and analyses are <u>*planned*</u> *to address the points raised by the referees.*

Reviewer comments in bold. Our response in non-bold.

Reviewer #1 (Evidence, reproducibility and clarity (Required)):

This paper by Konstantinou et al aims at deciphering the mechanisms by which PTEN loss could be driving poorer prognosis in patients. The authors use their great high-throughput 3D screening method coupled to an unbiased proteomic method and a CRISPR screen to uncover a new pro-invasive axis driving collective invasion of high-grade serous ovarian carcinoma (HGSOC) cells. Overall, this is a very impressive study, very well done and

controlled with rigorous statistical analyses that uses sophisticated methods to convincingly show that the CYTH2-ARF6-AGAP1-ITGA6/ITGB1 module is required for the pro-invasive effect of PTEN depletion and discriminates patients with poorest prognosis.

MAJOR COMMENTS

Below are listed all the claims that, in my opinion, are not adequately supported by the data.

1) Choice of the cell line: More justification on the use of the ID8 cell line and on the p53 deletion is needed. The authors need to clearly state that most p53 mutations in ovarian cancer are missense mutations that lead to a strong accumulation of a p53 protein devoid of transcriptional activity. Nevertheless, it seems that p53 mutations are not associated to differences in patient survival. Hence the choice of studying PTEN loss in the complete absence of p53, a situation that does not mirror the clinical situation, needs to be explained. Moreover, the in vivo experiments already performed in the literature mentioned in the discussion should be mentioned in the introduction to provide more context and physiological relevance to this study (especially regarding the special focus on the p53 null/ dKO cells throughout the study).

We will update the manuscript with a detailed explanation of the cell line of choice. Briefly, while indeed Tp53 is found mutated in HGSOC, approximately 30-35 % of these are classified as null mutations (PMID: 21552211), making models with null Trp53 representative of the clinical situation. Further, there is no difference in patient outcome in HGSOC by Tp53 mutation type (PMID: 20229506), while gene expression data from TCGA suggest that HGSC is marked by loss of wild-type P53 signalling regardless of Tp53 mutation type (PMID 25109877). Thus, we believe our choice of model can faithfully mirror the clinical situation.

2) "Therefore, PTEN loss in ovarian cancer, particularly at the protein level, occurs in the tumour epithelium and is associated with upregulated AKT signalling and poor overall survival". This claim is an over-interpretation and over-generalisation of the data presented. I appreciate the honesty of the authors in showing all the ovarian datasets that are available and highlight the discrepancies in expression of the proteins they study in stroma and epithelium. I think the way to present these data in the text without over-interpreting and generalizing would be to show that there is a clear epithelial-specific downregulation of PTEN at the mRNA level. Most likely due to the contribution to other cell types in the stroma, only 3 out of 5 bulk tumour mRNA datasets show a tumour specific downregulation of PTEN and no association with survival based on a median split of PTEN mRNA expression. Nevertheless, although there is no direct correlation between PTEN mRNA and protein levels, patients with low PTEN protein levels have poorer survival that is associated to an upregulation of Akt signalling. This allows to have a

clearer conclusion, based solely on the protein data presented and no over-generalisation using the mRNA data. This, to me, makes a stronger case for studying PTEN loss in ovarian cancer and is fully supported by the data presented.

We will incorporate this reviewer suggestion into the modified manuscript.

3) PTEN loss induces modest effects in 2D culture. The authors make claims regarding the fact that some of the phenotypes they look at happen after PTEN depletion alone or in combination with p53 loss and are more prominent in 3D vs 2D. Many of these are insufficiently backed up by data. A few key experiments are also only performed in 2D and should be done in 3D. Finally, some clarifications if the role of PTEN is most prominent on either collective, ECM-induced or 3D-dependent invasion.

some clarifications if the role of PTEN is most prominent on either collective, ECM-induced or 3D-dependent invasion

We believe that the reviewer may be confused. Both of our models, either spheroids or invading monolayers, are events occurring inside gels of ECM. Therefore, these are all are 3D, ECM-induced, collective invasion. We have not performed 2D migration assays. We apologise that the this was not clearer in the first submission. We will correct this in the updated manuscript.

First, the authors claim that PTEN loss alone (i.e. without p53 deletion) leads to changes in Akt signalling. Supp fig 1H clearly shows that there is no significant increase in Akt activation, although there seems to be one in the Western Blot (WB) presented in supp fig 1G. There is a clear, significant increase in the Akt activation in all the PTEN KO clones when in association with p53 loss though. This claim is hence not backed up by data and the conclusion seems to be that the effect on Akt signalling requires both deletion of p53 and PTEN.

The reviewer is correct: that the increase to pAKT levels upon PTEN KO is more robust with co-KO of TP53, thereby indicating synergy with p53. We will update the manuscript to note this, accordingly.

It will be interesting to see a quantification of the pS473-Akt staining (supp fig S1J), as it seems from these images that pAkt is preferentially found on rounded cells. It should also be performed in 3D conditions to see if there is an enrichment at invasive tips and back-up the invasion data.

This observation made us realise that the images we had included were giving the wrong impression (that pAkt levels would be highest in round cells). Based on the quantitation in Fig. S1M, PTEN KO cells (which have elevated pAkt levels), show a marked depletion of rounded cells. Therefore, pAkt elevated is not associated with being enriched in rounded cells. We will replace this image with cells mirroring the phenotypes quantified in Fig S1M.

We used 2D for quantitation of pAKT staining, as we perform a like for like comparison. We cannot compare pAkt in 3D protrusions accurately between genotypes because of the frequency of protrusions: in p53 KO protrusion are rare. In 3D, therefore, it is not a situation where protrusions are present in both genotypes and we compare enrichment or depletion in a stable structure. Rather, what we can provide is whether when protrusions form, there is clear pAkt labelling in a protrusion. We will include for the revision a representative image of each phenotype in 3D, including a 3D $Trp53^{-/-}$;$Pten^{-/-}$ spheroid stained for pAKT S473.

Arf6 is recruited to the invasive tips of cells invading a 2D wound (fig4D). How do the authors reconcile the fact that all the machinery required for 3D invasion is present but that PTEN loss has a modest effect on cells in 2D? If the wound assay was done on glass, it should be done again on ECM coated glass to see if it recapitulates the effects seen in 3D. This experiment will help deconvolute if the effect of PTEN loss is more linked to collective behaviour than 3D organization or presence of ECM.

We again apologise for not being clearer in our description. Both the wound assays and the IF of invading monolayer were performed with cell monolayers invading into Matrigel. Monolayers are grown on top of Matrigel, wounded, and then overlayed with Matrigel. Therefore, this is orthogonal to our spheroid assay, and completely 3D. We will address this comment by changing the text in the results section to highlight the 3D nature of the method.

The recycling assays are all done in 2D, condition under which the authors claim that the PTEN phenotype is weakest. Although I understand that it is not possible to do this assay in 3D, its contribution to elucidating the mechanism by which integrins participate in the PTEN loss invasive phenotype is not clear. The requirement of integrins relies on the data showing that ITGB1 KO results in no collagen4-positive basement membrane of the cysts and greatly impaired invasion. Experiments looking at the integrin localisation would be helpful: can an enrichment at the invasive tips can be seen? Are ITGA6 and/or ITGB1 repartitions homogeneous between the cysts membranes and the invasive tips? In my opinion the Src/FAK data is not enough to draw the conclusions of fig7I schematic.

We will endeavour to include images of 3D spheroids of Trp53-/-;Pten-/- cells and stained for β1 integrin (total and active) and α5 integrin to interrogate localisation at the tips.

4) Expression of AGAP1 isoforms do not alter ARF6 levels. Data in fig 6C, D show a significant downregulation of Arf6 and Akt signalling after expression of AGAP1S. Can the authors clarify what they mean?

We thank the reviewer for picking up that discrepancy between the results and the text. We will change the relevant text to highlight that expression of AGAP1S is associated with a statistically significant reduction of roughly 30% in ARF6 levels and 10% in p:t AKT. We do not know why AGAP1s may enact such an effect.

5) Arf6 is not modulated in the different cell lines: data in fig4B (far right graph) and supp fig 4B, J seem to indicate otherwise. Can the authors clarify what they mean?

It is not clear exactly what the reviewer is referring to here. If the reviewer is referring to Supplementary Figure 4B, this is an experiment examining the levels of ARF5 or ARF6 upon knockdown, so levels would be expected to vary. Fig S4B does not correspond to the experiment performed in S4J. Our interpretation is that loss of p53 alone or in combination with Pten does not seem to be consistently be accompanied with an increase in either the levels of total or bulk GTP-bound ARF6 that could explain the dependency of $Trp53^{-/-}$;$Pten^{-/-}$ on the GTPase for the invasive phenotype. We will make our interpretation clearer in the text

6) Immunofluorescence panels without quantifications: Quantifications for the different stainings shown in fig3A; 4D, E; 5H; 7B and supp fig S1L, J; S3 need to be included to fully back the conclusions of the authors. Indeed, these images are used to draw conclusions and not only as illustrations.

It is not possible to do a direct comparison between protrusion vs no protrusion (see our response above). We will include a line scan to show clear enrichment at the end of the tip for image shown. Quantitation for Figure S1L is already included (S1K and M), quantitation for Figure S1J is presented in Fig S1I and for Fig 5H quantitation of the phenotype is present in Fig 5I.

7) Quantifications of invasion show that WT cysts become hyper-protrusive at around the half experiment mark (around 30-40hrs). Nevertheless, all movies or galleries show spherical cysts, which does not seem representative. Can the authors change this or explain why these images/movies were chosen?

We present the fold change at each time point because that is intuitively easier to understand rather than the raw number. The quantitation does not show that the cysts necessarily become hyper-protrusive at the specific timepoint, but rather that the proportion of hyper-protrusive cysts observed in this genotype peaks at the specific timepoint. This phenotype may still be in the minority of behaviours. As an example, something that occurs 5% of the time in the control, with a two-fold increase in behaviours, might still only be 10% of the population. Therefore, adding in a picture that may be representative of a small proportion of the population may not be a realistic depiction of what is happening across the entire population. We will provide the reviewer with the exact percentage of spheroids that are classified as hyper-protrusive at the specific cell line across timepoints, to make this clearer.

8) Since it seems that the main effect of PTEN is to drive the localisation and intensity of recycling of Arf6 cargoes, it will be helpful to confirm that all the proteins involved in the Arf6 module be shown to be accumulated/present at the pro-invasive tips. Immunofluorescence stainings showing the presence of AGAP1 (could be done with the AGAP1S isoform that is mNeon-tagged), pS473-Akt, ITGB1 (active integrin if possible,

otherwise total integrin), ITGA5, PI3K should be included if possible. A quantification comparing signal in the cysts and in the invasive tips should also be included to see if there is an accumulation to PIP3-enriched areas.

We will endeavour to include the requested images.

9) Data in fig5I convincingly show that PTEN loss induces a fragmented collagen4-positive basement membrane. The authors use this data to claim that this is one of the ways that PTEN could be driving invasion but no correlation between these structures and the hyper-protrusive phenotype is made. This experiment needs to be done to support this claim.

This comment made us realise that in an attempt to make images simpler (displayed nuclei and COL4 only), we omitted a staining for where protrusions were moving through gaps in the ECM. We will update these times to demonstrate such events.

MINOR COMMENTS

1) Data visualization: I think that the heatmap representation is overkill when only 2 or 3 conditions are presented. A graph showing the evolution of area or spherical/Hyper-protrusive phenotype proportions across time would be easier to read and more impactful: each genotype could be presented with a colour and the spherical/hyper-protrusive phenotypes as either plain or dashed lanes across time. I understand that this representation allows for the stats to be done at each time points but they are generally pretty clear (especially for the PTEN KO or dKO phenotypes) and do not need to be done for each time point in my opinion. These heatmaps could be put in supplementary figures if the authors feel strongly about putting stats for each time points.

We thank their reviewer for their suggestion. We believe that our approach, while complex, is the best visualisation to reflect both the changes across time but also between conditions while allowing appreciation of the statistical significance. This visualisation has been optimised by our lab over years of working with this type of data and we would prefer that they remain consistent with the accepted standard of our other publications. We are, however, happy to expand the explanation in the text on how to interpret the bubble heatmaps.

Fig supp S1M, fig 5I should be presented as a stacked histogram to improve readability and merged with fig supp S1K.

We will merge Figures S1M and S1K. We believe that Figure 5I is easier to read as is.

Displaying fold change as antilog rather than log values would be easier for the reader to realise the magnitude of the differences.

We disagree with the reviewer.

A bar graph would be easier to read than the matrix representation for fig 6B.

We disagree with the reviewer as we feel it makes it easier to directly compare each lipid between the two cell lines.

The way Area data is presented throughout to me makes it very difficult to understand what is going on. Could the authors at least give some explanations in figure legends. A curve graph displaying the evolution of the area across time would be easier to read and see the differences between conditions.

Please see our response to Minor point 1

2) It is confusing that, in fig supp S1M, there is a significant decrease of the rounded phenotype after PTEN loss that is not associated to a significant change in another of the categories. Could the authors explain how?

This can be simply explained from our data: while the rounded phenotype was reduced in a consistent way across replicate experiments (therefore resulting in significance), the effect on the other two phenotypes was not consistent (not set in magnitude and directionality). This therefore does not lead to a significant (i.e. consistent) effect on the latter two phenotypes. PTEN loss therefore seems to allow cells to undergo – at the expense of being round - a range of shape changes, rather than a set phenotype.

3) One of the big differences of the PTEN KO cells seems their ability to invade through the matrigel bed and migration on the glass below (supp movie S2). From what I gather, these cysts would be considered out of focus and excluded from the analysis. Would it be possible that this would minimize some of the results? Would it be possible to include a quantification of this particular phenotype to confirm it is specific to PTEN KO cells?

In the same spirit, could the authors provide the percentage of non-classified cysts, to make sure that the same proportion of cysts is quantified across all different genotypes.

Indeed, we cannot exclude that we under-estimate the magnitude of the effect on the PTEN null. We will include this point in the discussion. We can include a reviewer-only figure showing the proportion of cysts and levels of the 'OutOfFocus' objects across cell lines.

4) Can the authors clarify how a 0 fold change (in log value) in fig 2D can be highly significant?

We believe that the reviewer is equating statistical significance with something being biologically meaningful. Statistical analysis does not indicate *a priori* whether something is biologically meaningful. Rather, it assesses the likelihood that an observed result is occurring by

chance (or not). For instance, if a small change (e.g 0.04 in a log2 fold change) occurred repeatedly across experimental replicates this is unlikely to be a result of chance, and therefore could be statistically significant. Yet, such a small magnitude of effect is probably biologically minor. This is why our heatmaps provide both statistical significance, fold change, and consistency in magnitude of effect.

5) Delta isoform of PI3K seems to have an effect on area in the middle of the experiment, but has no effect at all on invasion. Could the authors comment? Are these smaller cysts still as invasive? There might be an interesting uncoupling between proliferation and invasion there.

The cysts are actually slightly larger with PI3Kδ inhibition and there is no change in invasion. We will expand our comments in text as well to account for this observation.

6) ITGB1 depletion seems to induce a downregulation of Akt protein. Is that right? Does it change Akt localisation? Is there a dose effect whereby there is not enough Akt protein to mediate invasion?

The p:t AKT ratio does not change consistently across all gRNAs (Figure 5C) but we can look at Akt (total) protein levels and include this information if needed.

7) Stats should be added directly on the graphs for the recycling assays, doing a pairwise comparison of the different genotypes for each time points. Can the authors clarify what the t-32min quantification graphs adds (fig7E, supp fig S8G-I)? I would advise to remove them, as this data is already presented in the recycling assay graphs.

We don't include these because although they are technical replicates, they are demonstrative of a single experiment. What we include instead is the quantitation across independent biological experiments (which each have their own internal multiple technical replicates), where it is appropriate to include statistical analysis.

8) There is a substantial amount of typos and erroneous references to figures. I listed below the ones that I spotted and I encourage the authors to carefully check.

a. there are some mistakes in referencing the number of cysts in supp table 1. There is for example no cysts experiments in Figure 1 but yet there are some references to figure 1 in supp table 1. Please correct it. I think it will be easier for the reader if the number of cysts quantified for each conditions was also indicated in the figure legends. Supp table 1 can still be included for readers that want additional details.

b. comma missing page 3

c. page 3 and 4: PI(3,4)2 means PI(3,4)P2? Can be shorten to PIP2 for ease of read and specify if it is another PIP2 specie otherwise

d. define CYTH abbreviation: I suppose this is for cytohesin?

e. fig1F-I: don't understand why TCGA.OV is specified on some but not all the graphs. It seems to me that all the data are from TCGA.OV? Makes it seems it is nit the case

f. legend of fig1H, I: y axis is -Log10 values in 1I, not Log10 values

g. page 6: dKO abbreviation is already specified above and should be used to avoid repetition and for ease of read

h. supp fig S1D: missing legend for the second bar (after Wild Type)

i. supp fig S1N: legend of the X-axis should be below the axis

j. supp fig S1O: the numerotation of the X-axis needs to be below the line of the axis for ease of read, not above it

k. legend of S2A: clones 1.12 and 1.15 are p53-/-;PTEN-/- and not PTEN-/-

l. supp figS2C can the authors specify the different stages of matrigel (liquid or gel) that are used for the invasion assay, to make it easier for the non-specialist to understand what is going on. Please confirm that the 50% GFR matrigel makes a gel on top of the cells and fill in the wound to produce the 3D invasion assay setup.

m. page 7: no parental cells are used in S3A, B only p53 null and p53 null and dKO. Please also specify what cells are being compared in the text

n. description of arrow heads and colours need to be moved to figure legends and not in main text (page 7)

o. fig 2D: the signification of the dot in the circles needs to be in the legends (since it is its first apparition in the manuscript). It only appears later on, in supp2A legend. Additional description of the matrices is necessary, as they contain a lot of information to digest to understand fully what is going on

p. legend of fig3: error in figure reference: area data is D and not E, protrusive phenotypes are E and not F

q. arrow missing in fig3B

r. fig 3D,E, G, H: please indicate the cell line studied

s. fig 3I: the different genotypes need to be stated on the galleries for clarity

t. page 8: define Arf6-mNG in the text

u. page 9: "<" symbol should be an alpha symbol

v. fig 4A: indicate the cell line used on the figure

w. supp fig S4E: why is it specified mouse-specific for the shArf6?

x. 4H, I, J: indicate on the figure if these interactors are mostly unchanged, strong interactors or weak interactors for clarity

y. legend of fig4H: "coloured spots underneath denote the protein complex that each interactor belongs (in J)" should indicate panel G and not J

z. fig4I, J: are you sure of the legend for the fold change coloring? Log2 of 1 is a 0 fold change, i don't see how these could show any significant difference (i.e. some of the pale red circles are significant)

aa. page 11: description of the assay (starting with "Machine learning classification of...") is very confusing, please clarify

bb. page16: figure 4H should be 4I (PTEN-null specific association of Arf6 with ITGA5)

cc. supp fig S5H-P: choose Tumour or cancer to homogeneise naming across the graphs

dd. fig 5H: box are difficultly visible in green, change color to yellow or something more visible

ee. page 13: Fig6E, F should also refer to 6G

ff. LCM abbreviation on page 10 and 12 refers to LCMD? Otherwise please define it.

We thank the reviewer for their careful examination of the manuscript. We will go through all above points and make the corresponding careful adjustments to the manuscript.

OPTIONAL SUGGESTIONS

Revision Plan

1) Choice of cell line: There is a high number of patients (around 9% according to (Cole et al. 2016)) that present the R248Q gain-of-function mutation. A recent study has shown that this mutant p53 protein is associated to an activation of Akt signalling and an increase of the intercellular trafficking of EGFR (Lai et al. 2021). Given that EGFR was also a hit in this screen, that is seems to have a central role in Arf6 cargoes (fig 4G), I think it would be a great addition to this study. It could hence cooperate with PTEN loss to drive strong, robust invasion.

This is an excellent observation and one we will likely follow-up in an independent study.

2) Are MAPK involved in the PTEN KO pro-invasive phenotype? In particular Erk1/2, since EGFR is one of the PTEN loss induced Arf6 cargoes.

This is an excellent observation and one we will likely follow-up in an independent study.

REFERENCE

Cole, Alexander J., Trisha Dwight, Anthony J. Gill, Kristie-Ann Dickson, Ying Zhu, Adele Clarkson, Gregory B. Gard, et al. 2016. « Assessing Mutant P53 in Primary High-Grade Serous Ovarian Cancer Using Immunohistochemistry and Massively Parallel Sequencing ». Scientific Reports 6 (1): 26191. https://doi.org/10.1038/srep26191.

Lai, Zih-Yin, Kai-Yun Tsai, Shing-Jyh Chang, et Yung-Jen Chuang. 2021. « Gain-of-Function Mutant TP53 R248Q Overexpressed in Epithelial Ovarian Carcinoma Alters AKT-Dependent Regulation of Intercellular Trafficking in Responses to EGFR/MDM2 Inhibitor ». International Journal of Molecular Sciences 22 (16): 8784. https://doi.org/10.3390/ijms22168784.

Reviewer #2 (Evidence, reproducibility and clarity (Required)):

The authors have conducted a study of the molecular requirements for cancer invasion that is stunning in its thoroughness and depth and breadth of its molecular analysis. The writing is exceptionally precise though also very dense (see below). The molecular model proposed is that PTEN loss (in a p53 null background) leads to reliance upon ARF6 for invasion, with regulation through interactions with AGAP1 and beta1-integrin and it is convincingly demonstrated. They focus on interpreting the consequences of genetic and pharmacologic manipulations in a cell line, using a series of 2D and 3D assays. The phenotypes are more prominent in 3D assays.

Concerns and Suggestions:
1. There is a disconnect between the essentially complete loss of protrusions and invasion in 3D (e.g. 4A) and the reduction in magnitude of protrusive invasion but the continued presence of elongated cells with protrusions in 2D (e.g. S4C). This discrepancy is present in a couple of comparisons and is glossed over in quick callouts to many figure panels.

We thank the reviewer for mentioning this as this comment was very helpful in determining that we needed to clarify our description of the role of ARF6 to protrusion formation vs maturation. In the $Trp53^{-/-}$ genotype, protrusions can form, but they rapidly retract, failing to mature into structures that drive invasion through ECM (e.g. Figure S2E). This protrusion maturation occurs upon PTEN KO. When ARF6 depleted, PTEN-null cells can form protrusions, but now again lack the ability to mature into invasion-inducing structures.

This concept of needing ARF6 for protrusion maturation and maintenance is underpinned by our model of ARF6 regulating recycling of active integrin back to the protrusion front. Indeed, we have observed ARF6 being required not for protrusion initiation, but rather ensuring protrusions are not retracted in other contexts (i.e. upon loss of the ARF6 GEF protein IQSEC1 in invading 3D culture of PC3 cells; PMID: 33712589).

We also note that, as responded to Reviewer 1, the assay is a 3D invasion rather than 2D migration assay, with cells sandwiched between Matrigel.

We will update the relevant sections of the results and discussion with the point above.

2. Once a journal has been identified, it would be wise for the editor to allow some flexibility in word limit to enable some very dense sections to be expanded slightly to guide the reader through the experiments and results more clearly. For example, in the section "ARF6 regulates active integrin pools...", there are callouts like (Fig. 7C,E; S8A-C; G-I) and then (Fig. 7D,E; Fig. S8E-F, H-I). It takes a lot of time to unpack these different experimental claims based on a single sentence.

We greatly appreciate the refreshing comments of this reviewer to advocate for actions to improve clarity in our reporting. We would take glad advantage of such a possibility.

3. The patient data on CYTH2 and its relationship to survival is modestly convincing.

In Ovarian Cancer, effects on survival are often minor. This is not a disease where one often sees large shifts in survival, which is why we are so excited about the large shifts that we do see with the ARF GTPase module we identified. However, we concede that the effects on CYTH2, although significant, are not vast changes. We will point this out and tone down our language.

4. Very minor- search on %- there are a few inconsistencies in terms of spaces and commas vs. periods. The Methods also have some inconsistencies in terms of spaces between numbers and units or numbers and degrees Celsius. References are also in a different font. Overall it was extremely carefully written though (just dense).

We thank the reviewer for their careful inspection of our manuscript. We will carefully go over the sections flagged before resubmission

Reviewer #2 (Significance (Required)):

One limitation of the experimental design is that the depth of molecular analysis in vitro comes at the expense of any in vivo validation, which the authors acknowledge in the Discussion. They attempt to make similar points using analysis of patient survival data from public databases but these analyses generally yielded small magnitude differences. The main audience for this study is likely to be cell biologists interested in cell migration, cell-ECM adhesion, cancer invasion, and GTPases. I don't see any need for new experiments- what can be done has been done and then some. I do think that it would benefit readers if the text could be made less dense.

Reviewer #3 (Evidence, reproducibility and clarity (Required)):

Summary:
Using a murine HGSOC 3D cell model, in combination with analysis of human ovarian cancer datasets, the authors uncover a CYTH2-ARF6-AGAP1 signaling module regulated by PTEN and identify a biomarker for tumor invasion and targeted therapy.

Major comments:
The findings of this study are significant as they reveal a critical signaling module that controls tumor invasion by mediating tumor cell interaction with the extracellular matrix. The experiments are properly designed, and the data are well presented. The conclusions are appropriate and supported by the data. The limitation of the study has also been discussed properly.

One suggestion regarding the survival analysis in Fig. 6 and 7.
The authors noted that the CYTH2-ARF6-AGAP1 module is not specifically or only induced in Pten-null contexts, but rather that Pten-null cells become more dependent on the module for enacting the invasive phenotype. Based on this, it would be interesting to evaluate how the PTEN status impacts the survival difference by integrating the PTEN genomic status (WT versus mutation) or its expression level (protein or mRNA) into the survival analysis of patient cohorts in Fig. 6 and Fig. 7.

We thank the reviewer for this excellent point. We will include such analysis, where possible. One consideration will be that extensive division of patients based on these molecular characteristics may results in patient numbers too low to draw conclusions of significance.

****Referees cross-commenting****

Gene deletion and mutation may elicit different functional outcomes. I therefore agree with Reviewer #1 that "the choice of studying PTEN loss in the complete absence of p53, a situation that does not mirror the clinical situation, needs to be explained".

We will make our reasons for this choice clear in the text before submission. Please refer to response to Reviewer 1, Major comment 1.

Reviewer #3 (Significance (Required)):

The model used and data presented in this study are of significance for both scientific discovery and clinical application, which will interest the broad audience in both basic and clinical research.

Revision Plan

3. Description of the revisions that have already been incorporated in the transferred manuscript

4. Description of analyses that authors prefer not to carry out

Please include a point-by-point response explaining why some of the requested data or additional analyses might not be necessary or cannot be provided within the scope of a revision. This can be due to time or resource limitations or in case of disagreement about the necessity of such additional data given the scope of the study. Please leave empty if not applicable.

Dear Dr Bryant,

Thank you for submitting your work for consideration by the EMBO Journal and transferring your manuscript from Review Commons, now listed as EMBOJ-2023-113987. My apologies for getting back to you with unusual protraction due to the high current load of submissions to the journal.

We have now carefully assessed your manuscript together with the referee reports and your point-by-point response to their concerns. I am happy to say that we find the results to be of interest for the EMBO Journal, and thus are positive to have a revised study re-evaluated by the referees.

Given the referees' positive recommendations and based on your detailed response, I would thus like to invite you to submit a revised version of the manuscript, addressing the issues raised along the lines of your response. I should add that it is EMBO Journal policy to allow only a single round of revision, and acceptance of your manuscript will therefore depend on the completeness of your responses in this revised version.

We generally allow three months as standard revision time. As a matter of policy, competing manuscripts published during this period will not negatively impact on our assessment of the conceptual advance presented by your study. However, we request that you contact the editor as soon as possible upon publication of any related work, to discuss how to proceed. Should you foresee a problem in meeting this three-month deadline, please let us know in advance and we may be able to grant an extension.

When submitting your revised manuscript, please carefully review the instructions below.

Please feel free to approach me any time should you have additional questions related to this.

Thank you for the opportunity to consider your work for publication.

I look forward to your revision.

Kind regards,

Daniel Klimmeck

Daniel Klimmeck, PhD
Senior Editor
The EMBO Journal

Instruction for the preparation of your revised manuscript:

1) a .docx formatted version of the manuscript text (including legends for main figures, EV figures and tables). Please make sure that the changes are highlighted to be clearly visible.

2) individual production quality figure files as .eps, .tif, .jpg (one file per figure).

3) a .docx formatted letter INCLUDING the reviewers' reports and your detailed point-by-point response to their comments. As part of the EMBO Press transparent editorial process, the point-by-point response is part of the Review Process File (RPF), which will be published alongside your paper.

4) a complete author checklist, which you can download from our author guidelines (https://wol-prod-cdn.literatumonline.com/pb-assets/embo-site/Author Checklist%20-%20EMBO%20J-1561436015657.xlsx). Please insert information in the checklist that is also reflected in the manuscript. The completed author checklist will also be part of the RPF.

5) Please note that all corresponding authors are required to supply an ORCID ID for their name upon submission of a revised manuscript.

6) It is mandatory to include a 'Data Availability' section after the Materials and Methods. Before submitting your revision, primary datasets produced in this study need to be deposited in an appropriate public database, and the accession numbers and

database listed under 'Data Availability'. Please remember to provide a reviewer password if the datasets are not yet public (see https://www.embopress.org/page/journal/14602075/authorguide#datadeposition).
In case you have no data that requires deposition in a public database, please state so in this section. Note that the Data Availability Section is restricted to new primary data that are part of this study.
*** Note - All links should resolve to a page where the data can be accessed. ***

7) Our journal encourages inclusion of *data citations in the reference list* to directly cite datasets that were re-used and obtained from public databases. Data citations in the article text are distinct from normal bibliographical citations and should directly link to the database records from which the data can be accessed. In the main text, data citations are formatted as follows: "Data ref: Smith et al, 2001" or "Data ref: NCBI Sequence Read Archive PRJNA342805, 2017". In the Reference list, data citations must be labeled with "[DATASET]". A data reference must provide the database name, accession number/identifiers and a resolvable link to the landing page from which the data can be accessed at the end of the reference. Further instructions are available at .

8) At EMBO Press we ask authors to provide source data for the main and EV figures. Our source data coordinator will contact you to discuss which figure panels we would need source data for and will also provide you with helpful tips on how to upload and organize the files.

Numerical data can be provided as individual .xls or .csv files (including a tab describing the data). For 'blots' or microscopy, uncropped images should be submitted (using a zip archive or a single pdf per main figure if multiple images need to be supplied for one panel). Additional information on source data and instruction on how to label the files are available at .

9) We replaced Supplementary Information with Expanded View (EV) Figures and Tables that are collapsible/expandable online (see examples in https://www.embopress.org/doi/10.15252/embj.201695874). A maximum of 5 EV Figures can be typeset. EV Figures should be cited as 'Figure EV1, Figure EV2" etc. in the text and their respective legends should be included in the main text after the legends of regular figures.

- For the figures that you do NOT wish to display as Expanded View figures, they should be bundled together with their legends in a single PDF file called *Appendix*, which should start with a short Table of Content. Appendix figures should be referred to in the main text as: "Appendix Figure S1, Appendix Figure S2" etc. See detailed instructions regarding expanded view here: .

- Additional Tables/Datasets should be labelled and referred to as Table EV1, Dataset EV1, etc. Legends have to be provided in a separate tab in case of .xls files. Alternatively, the legend can be supplied as a separate text file (README) and zipped together with the Table/Dataset file.

10) When assembling figures, please refer to our figure preparation guideline in order to ensure proper formatting and readability in print as well as on screen:
http://bit.ly/EMBOPressFigurePreparationGuideline

Please remember: Digital image enhancement is acceptable practice, as long as it accurately represents the original data and conforms to community standards. If a figure has been subjected to significant electronic manipulation, this must be noted in the figure legend or in the 'Materials and Methods' section. The editors reserve the right to request original versions of figures and the original images that were used to assemble the figure.

11) For data quantification: please specify the name of the statistical test used to generate error bars and P values, the number (n) of independent experiments (specify technical or biological replicates) underlying each data point and the test used to calculate p-values in each figure legend. The figure legends should contain a basic description of n, P and the test applied. Graphs must include a description of the bars and the error bars (s.d., s.e.m.).

Further information is available in our Guide For Authors: https://www.embopress.org/page/journal/14602075/authorguide

We realize that it is difficult to revise to a specific deadline. In the interest of protecting the conceptual advance provided by the work, we recommend a revision within 3 months (20th Jun 2023). Please discuss the revision progress ahead of this time with the editor if you require more time to complete the revisions. Use the link below to submit your revision:

Link Not Available

***

Rev_Com_number: RC-2022-01776
New_manu_number: EMBOJ-2023-113987
Corr_author: Bryant
Title: PTEN deficiency exposes a requirement for an ARF GTPase module in integrin-dependent invasion in ovarian cancer.

EMBOJ-2023-113987
Response to reviewers

Reviewer comments in bold. Our response in non-bold with changes in text highlighted in blue.

Reviewer #1 (Evidence, reproducibility and clarity (Required)):

This paper by Konstantinou et al aims at deciphering the mechanisms by which PTEN loss could be driving poorer prognosis in patients. The authors use their great high-throughput 3D screening method coupled to an unbiased proteomic method and a CRISPR screen to uncover a new pro-invasive axis driving collective invasion of high-grade serous ovarian carcinoma (HGSOC) cells. Overall, this is a very impressive study, very well done and controlled with rigorous statistical analyses that uses sophisticated methods to convincingly show that the CYTH2-ARF6-AGAP1-ITGA6/ITGB1 module is required for the pro-invasive effect of PTEN depletion and discriminates patients with poorest prognosis.

MAJOR COMMENTS

Below are listed all the claims that, in my opinion, are not adequately supported by the data.

1) Choice of the cell line: More justification on the use of the ID8 cell line and on the p53 deletion is needed. The authors need to clearly state that most p53 mutations in ovarian cancer are missense mutations that lead to a strong accumulation of a p53 protein devoid of transcriptional activity. Nevertheless, it seems that p53 mutations are not associated to differences in patient survival. Hence the choice of studying PTEN loss in the complete absence of p53, a situation that does not mirror the clinical situation, needs to be explained. Moreover, the in vivo experiments already performed in the literature mentioned in the discussion should be mentioned in the introduction to provide more context and physiological relevance to this study (especially regarding the special focus on the p53 null/ dKO cells throughout the study).

We updated the manuscript with a detailed explanation of the cell line of choice. Briefly, while indeed Tp53 is found mutated in HGSOC, approximately 30-35 % of these are classified as null mutations (PMID: 21552211), making models with null Trp53 representative of the clinical situation. Further, there is no difference in patient outcome in HGSOC by Tp53 mutation type (PMID: 20229506), while gene expression data from TCGA suggest that HGSC is marked by loss of wild-type P53 signalling regardless of Tp53 mutation type (PMID 25109877). Thus, we believe our choice of model can faithfully mirror the clinical situation.

The following has been added in pages 6-7:

" An approximate 30-35% of the observed *TP53* mutations are classified as null (Yemelyanova et al., 2011) with loss of wild-type P53 signalling observed regardless of

mutation type (Hoadley et al., 2014). As such *TP53*-null models of HGSOC constitute good representations of the clinical situation. Based on the above and the fact that patient outcome is not stratified based on TP53 mutation type (Ahmed et al., 2010), we utilised ID8 ovarian cancer cells knocked out (KO) for *Trp53* and *Pten*, alone or in combination(…)"

2) "Therefore, PTEN loss in ovarian cancer, particularly at the protein level, occurs in the tumour epithelium and is associated with upregulated AKT signalling and poor overall survival". This claim is an over-interpretation and over-generalisation of the data presented. I appreciate the honesty of the authors in showing all the ovarian datasets that are available and highlight the discrepancies in expression of the proteins they study in stroma and epithelium. I think the way to present these data in the text without over-interpreting and generalizing would be to show that there is a clear epithelial-specific downregulation of PTEN at the mRNA level. Most likely due to the contribution to other cell types in the stroma, only 3 out of 5 bulk tumour mRNA datasets show a tumour specific downregulation of PTEN and no association with survival based on a median split of PTEN mRNA expression. Nevertheless, although there is no direct correlation between PTEN mRNA and protein levels, patients with low PTEN protein levels have poorer survival that is associated to an upregulation of Akt signalling. This allows to have a clearer conclusion, based solely on the protein data presented and no over-generalisation using the mRNA data. This, to me, makes a stronger case for studying PTEN loss in ovarian cancer and is fully supported by the data presented.

We incorporated this reviewer suggestion into the modified manuscript. The first Result's paragraph now reads:

"(…) As such, an epithelial-specific downregulation of PTEN at the mRNA level is evident in those datasets. (…) Therefore, low *PTEN* protein levels in ovarian cancer are associated with upregulated AKT signalling and poor overall survival."

3) PTEN loss induces modest effects in 2D culture. The authors make claims regarding the fact that some of the phenotypes they look at happen after PTEN depletion alone or in combination with p53 loss and are more prominent in 3D vs 2D. Many of these are insufficiently backed up by data. A few key experiments are also only performed in 2D and should be done in 3D. Finally, some clarifications if the role of PTEN is most prominent on either collective, ECM-induced or 3D-dependent invasion.

some clarifications if the role of PTEN is most prominent on either collective, ECM-induced or 3D-dependent invasion

We believe that the reviewer may be confused. Both of our models, either spheroids or invading monolayers, are events occurring inside gels of ECM. Therefore, these are all are 3D, ECM-induced, collective invasion. We have not performed 2D migration assays. We apologise that the this was not clearer in the first submission.

In the updated manuscript we have made our description clearer and changed our wording to:

"Confirmation of this increased activity upon *Pten* KO occurred in orthogonal 3D invasion assays with monolayers plated on ECM, wounded, and then further overlaid with more ECM (Fig. EV 2B-D, Supplementary Movie 4). Tracking of the directionality of invasive front of the wound edge revealed an increase in additional depth and persistence occurred upon co-loss of *Pten* compared to *Trp53* alone (Fig. EV 2E). (…)"

Changes have also been made in figure legends to reflect the fact that we are observing invasion rather than migration using this assay.

First, the authors claim that PTEN loss alone (i.e. without p53 deletion) leads to changes in Akt signalling. Supp fig 1H clearly shows that there is no significant increase in Akt activation, although there seems to be one in the Western Blot (WB) presented in supp fig 1G. There is a clear, significant increase in the Akt activation in all the PTEN KO clones when in association with p53 loss though. This claim is hence not backed up by data and the conclusion seems to be that the effect on Akt signalling requires both deletion of p53 and PTEN.

The reviewer is correct: that the increase to pAKT levels upon PTEN KO is more robust with co-KO of TP53, thereby indicating synergy with p53.

We have update the manuscript to note this, accordingly.

"While *Pten* KO alone resulted in a trend towards increased AKT activation (pS473) this was not statistically significant. However, *Pten* KO in combination with *Trp53* KO, significantly increased AKT activation (Fig. EV 1C-H), indicating synergy between Pten and p53 depletion in stabilising pAKT."

It will be interesting to see a quantification of the pS473-Akt staining (supp fig S1J), as it seems from these images that pAkt is preferentially found on rounded cells. It should also be performed in 3D conditions to see if there is an enrichment at invasive tips and back-up the invasion data.

This observation made us realise that the images we had included were giving the wrong impression (that pAkt levels would be highest in round cells). Based on the quantitation in Fig. S1K, PTEN KO cells (which have elevated pAkt levels), show a marked depletion of rounded cells. Therefore, pAkt elevated is not associated with being enriched in rounded cells. We replaced this image with cells mirroring the phenotypes quantified in Fig S1M (now Fig EV 1K).

We used 2D for quantitation of pAKT staining, as we perform a like for like comparison. We cannot compare pAkt in 3D protrusions accurately between genotypes because of the frequency of protrusions: in p53 KO protrusion are rare. In 3D, therefore, it is not a situation

where protrusions are present in both genotypes and we compare enrichment or depletion in a stable structure. Rather, what we can provide is whether when protrusions form, there is clear pAkt labelling in a protrusion. We have included a representative image of 3D Trp53$^{-/-}$;Pten$^{-/-}$ spheroid stained for pAKT S473 and an accompanying line plot showing pAKT S473 enrichment at the very tip of the invasive protrusion (Figure EV 3E-F).

The following text has been added in the manuscript, page 9:

"Accordingly, the tips of the invasive protrusions in the *Trp53$^{-/-}$;Pten$^{-/-}$* spheroids were highly enriched for the PIP_3 effector pAKT (S473), prior to F-actin enrichment (Figure EV E,F, arrowheads)."

Arf6 is recruited to the invasive tips of cells invading a 2D wound (fig4D). How do the authors reconcile the fact that all the machinery required for 3D invasion is present but that PTEN loss has a modest effect on cells in 2D? If the wound assay was done on glass, it should be done again on ECM coated glass to see if it recapitulates the effects seen in 3D. This experiment will help deconvolute if the effect of PTEN loss is more linked to collective behaviour than 3D organization or presence of ECM.

We again apologise for not being clearer in our description. Both the wound assays and the IF of invading monolayer were performed with cell monolayers invading into Matrigel. Monolayers are grown on top of Matrigel, wounded, and then overlayed with Matrigel. Therefore, this is orthogonal to our spheroid assay, and completely 3D.

We changed the text in the results section pages 8 and 9 to highlight the 3D nature of the method:

"(..)no effect on invasion for ECM-embedded, wounded monolayers (Fig S4C-D). (…)We examined whether ARF6 localisation was modulated by PTEN loss. In *Trp53$^{-/-}$* spheroids and leader cells of invasive, ECM-embedded monolayers, ARF6-mNeonGreen (mNG) localised prominently at cell-cell contacts (white arrowheads), cell-ECM contacts (blue arrowheads), as well as to intracellular pools (green arrowheads) (Fig. 4D,F)."

The recycling assays are all done in 2D, condition under which the authors claim that the PTEN phenotype is weakest. Although I understand that it is not possible to do this assay in 3D, its contribution to elucidating the mechanism by which integrins participate in the PTEN loss invasive phenotype is not clear. The requirement of integrins relies on the data showing that ITGB1 KO results in no collagen4-positive basement membrane of the cysts and greatly impaired invasion. Experiments looking at the integrin localisation would be helpful: can an enrichment at the invasive tips can be seen? Are ITGA6 and/or ITGB1 repartitions homogeneous between the cysts membranes and the invasive tips? In my opinion the Src/FAK data is not enough to draw the conclusions of fig7I schematic.

We have included images of 3D spheroids of Trp53$^{-/-;}$Pten$^{-/-}$ cells stained for β1 integrin (total) and α5 integrin to interrogate localisation at the tips (Figure 7A-B). A line scan analysis of a representative example for each is also included. Both antibodies showed cortical localisation and pronounced enrichment at the tips of invasive protrusions. Unfortunately, we could not stain with an antibody specific for the active β1 integrin.

The following has been added in page 16:

"(…) In addition to cortical localisation, both a5-integrin and β1-integrin showed localisation to the extreme tips of invasive protrusions in *Trp53$^{-/-}$;Pten$^{-/-}$* spheroids, occurring prior to F-actin enrichment and mirroring the localisation observed for both pAKT (S473) and PIP3 (Fig. EV 7A-B). (…)"

4) Expression of AGAP1 isoforms do not alter ARF6 levels. Data in fig 6C, D show a significant downregulation of Arf6 and Akt signalling after expression of AGAP1S. Can the authors clarify what they mean?

We thank the reviewer for picking up that discrepancy between the results and the text.

We have changed the relevant text to highlight that expression of AGAP1S is associated with a statistically significant reduction of roughly 30% in ARF6 levels and 10% in p:t AKT. We do not know why AGAP1s may enact such an effect.

The text has been amended to: "(…)The AGAP1-S isoform modelstly decreased ARF6 levels (~ 30% reduction) and AKT activation (~ 10% reduction) (Fig. 6C, D). Accordingly, mNG-AGAP1-S-expressing spheroids were (…)"

5) Arf6 is not modulated in the different cell lines: data in fig4B (far right graph) and supp fig 4B, J seem to indicate otherwise. Can the authors clarify what they mean?

It is not clear exactly what the reviewer is referring to here. If the reviewer is referring to <u>Supplementary</u> Figure 4B, this is an experiment examining the levels of ARF5 or ARF6 upon knockdown, so levels would be expected to vary. Fig S4B does not correspond to the experiment performed in S4J.

Our interpretation from experiments described in S4H-J is that loss of p53 alone or in combination with Pten does not seem to be consistently accompanied with an increase in either the levels of total or bulk GTP-bound ARF6 that could explain the dependency of Trp53$^{-/-}$;Pten$^{-/-}$ on the GTPase for the invasive phenotype. Please refer to the relevant Results section in page 11:

"We observed no consistent alteration in global levels of *Arf6* mRNA (Fig EV 4H), protein (Fig EV 4I), or GTP-loading (Fig EV 4J) in *Trp53$^{-/-}$ or Trp53$^{-/-}$;Pten$^{-/-}$* cells compared to parental cell (WT), including multiple clones of the latter genotype.."

6) Immunofluorescence panels without quantifications: Quantifications for the different stainings shown in fig3A; 4D, E; 5H; 7B and supp fig S1L, J; S3 need to be included to fully back the conclusions of the authors. Indeed, these images are used to draw conclusions and not only as illustrations.

It is not possible to do a direct comparison between protrusion vs no protrusion (see our response above). We have now included a line scan to show clear enrichment at the end of the tip for image shown, see added panels in Figures: 3, S3, 4, 7, S7.

Quantitation for Figure S1L is already included (S1K and M in the pre-revisions manuscript, EV 1K in the revised manuscript), quantitation for Figure S1J is presented in Fig S1I (EV 1I) and for Fig 5H quantitation of the phenotype is present in Fig 5I.

7) Quantifications of invasion show that WT cysts become hyper-protrusive at around the half experiment mark (around 30-40hrs). Nevertheless, all movies or galleries show spherical cysts, which does not seem representative. Can the authors change this or explain why these images/movies were chosen?

The quantitation does not show that the cysts necessarily become hyper-protrusive at the specific timepoint in the Wild Type spheroids, but rather that the proportion of hyper-protrusive spheroids observed in this genotype peaks at the specific timepoint. This phenotype may still be in the minority of behaviours. As an example, something that occurs 10% of the time in the control genotype at maximum, still represents a minority of the population. Therefore, adding in a picture that may be representative of a small proportion of the population may not be a realistic depiction of what is happening across the entire population.

We have included a reviewer-only file with raw counts of the spheroids in each phenotype from the experiment presented in Figure 2D-E. In this it is clearly shown that at the time point of ~48h, where the Wild Type spheroids are classified as Hyperprotrusive the most, it is actually a small percentage of spheroids that does so (11.4% at Experiment 1, 5.95% at Experiment 2 and 19.7% for Experiment 3). In comparison PTEN null spheroids of the 1.15 sub-clone, which are actually mainly classified as Hyperprotrusive, are classified as such at 88.6% , 54,8% and 89.6% in each of the experiments respectively.

8) Since it seems that the main effect of PTEN is to drive the localisation and intensity of recycling of Arf6 cargoes, it will be helpful to confirm that all the proteins involved in the Arf6 module be shown to be accumulated/present at the pro-invasive tips. Immunofluorescence stainings showing the presence of AGAP1 (could be done with the AGAP1S isoform that is mNeon-tagged), pS473-Akt, ITGB1 (active integrin if possible, otherwise total integrin), ITGA5, PI3K should be included if possible. A quantification comparing signal in the cysts and in the invasive tips should also be included to see if there is an accumulation to PIP3-enriched areas.

The requested images and accompanying Line Scan analyses have now been included in the updated figures.

AGAP1 – Fig. EV 7E-F
PS473-Akt- Fig. EV 3A-B
ITGB1- Fig. 7A-B
ITGA5 – Fig. 7A-B
PI3Kβ- Fig. 3K-L

9) Data in fig5I convincingly show that PTEN loss induces a fragmented collagen4-positive basement membrane. The authors use this data to claim that this is one of the ways that PTEN could be driving invasion but no correlation between these structures and the hyper-protrusive phenotype is made. This experiment needs to be done to support this claim.

This comment made us realise that in an attempt to make images simpler (displayed nuclei and COL4 only), we omitted a staining for where protrusions were moving through gaps in the ECM. We have updated these images to demonstrate such events.

MINOR COMMENTS

1) Data visualization: I think that the heatmap representation is overkill when only 2 or 3 conditions are presented. A graph showing the evolution of area or spherical/Hyper-protrusive phenotype proportions across time would be easier to read and more impactful: each genotype could be presented with a colour and the spherical/hyper-protrusive phenotypes as either plain or dashed lanes across time. I understand that this representation allows for the stats to be done at each time points but they are generally pretty clear (especially for the PTEN KO or dKO phenotypes) and do not need to be done for each time point in my opinion. These heatmaps could be put in supplementary figures if the authors feel strongly about putting stats for each time points.

We thank their reviewer for their suggestion. We believe that our approach, while complex, is the best visualisation to reflect both the changes across time but also between conditions while allowing appreciation of the statistical significance. This visualisation has been optimised by our lab over years of working with this type of data and we would prefer that they remain consistent with the accepted standard of our other publications. We include an explanation on how to interpret the bubble heatmaps in all relevant figure legends. The following has also been added in the results section (Page 7):

"We used bubble heatmaps for size (Area) of the spheroids and the proportion of objects across genotypes classified as either Spherical or Hyper-protrusive. This allows simultaneous presentation of 1) the magnitude of change in phenotypes, 2) the statistical significance of each comparison and 3) whether the magnitude of the effect was reproducible across independent experiments(Freckmann et al., 2022). In the bubble heatmaps experiments are presented in 6-hour time chunks, representing the average value of phenotype proportion

during each interval. The control condition is presented as z-scored normalised values at each time period (Fig. 2D). For treatments, compared to the control condition, the colour of the circle corresponds to Log2 Fold change to control and the circle is scaled according to the statistical significance of the comparison (Cochran-Mantel Haenszel test with Bonferroni adjustment), with larger circle sizes corresponding to smaller p-values. Additionally, the presence of a black dot in the centre corresponds to effect magnitude, demonstrating homogeneity across strata (biological replicates) as determined by a non-significant p value using the Breslow-Day statistical test (with Bonferroni adjustment).

Fig supp S1M, fig 5I should be presented as a stacked histogram to improve readability and merged with fig supp S1K.

We merged Figures S1M and S1K (EV 1K). We believe that Figure 5I is easier to read as is.

Displaying fold change as antilog rather than log values would be easier for the reader to realise the magnitude of the differences.

We disagree with the reviewer.

A bar graph would be easier to read than the matrix representation for fig 6B.

We disagree with the reviewer as we feel it makes it easier to directly compare each lipid between the two cell lines.

The way Area data is presented throughout to me makes it very difficult to understand what is going on. Could the authors at least give some explanations in figure legends. A curve graph displaying the evolution of the area across time would be easier to read and see the differences between conditions.

Please see our response to Minor point 1

2) It is confusing that, in fig supp S1M, there is a significant decrease of the rounded phenotype after PTEN loss that is not associated to a significant change in another of the categories. Could the authors explain how?

This can be simply explained from our data: while the rounded phenotype was reduced in a consistent way across replicate experiments (therefore resulting in significance), the effect on the other two phenotypes was not consistent (not set in magnitude and directionality). This therefore does not lead to a significant (i.e. consistent) effect on the latter two phenotypes. PTEN loss therefore seems to allow cells to undergo – at the expense of being round - a range of shape changes, rather than a set phenotype.

3) One of the big differences of the PTEN KO cells seems their ability to invade through the matrigel bed and migration on the glass below (supp movie S2). From what I gather, these cysts would be considered out of focus and excluded from the analysis. Would it be possible that this would minimize some of the results? Would it be possible to include a quantification of this particular phenotype to confirm it is specific to PTEN KO cells?

In the same spirit, could the authors provide the percentage of non-classified cysts, to make sure that the same proportion of cysts is quantified across all different genotypes.

We have retrieved the information on the proportion of InFocus and OutOfFocus spheroids for the experiment comparing the following ID8 genotypes: Wild Type, Trp53$^{-/-}$ and Trp53$^{-/-}$;Pten$^{-/-}$ 1.11 and 1.15. In the specific experiment it seems that there were indeed more spheroids classified as Out of Focus in the PTEN-depleted sublines, particularly in the late time points. This is in line to what we were expecting as the longer the spheroids are in culture the most likely it is that they will have invaded to the plate bottom. Notably, the oppossite effect was observed for earlier timepoints.

Indeed, we cannot exclude that to some extent we under-estimate the magnitude of the effect on the PTEN null. This may be particularly true in later time points. Given that even under those circumstances the effect of PTEN depletion is clear, we believe our method is robust enough to accurately describe the emerging phenotype.

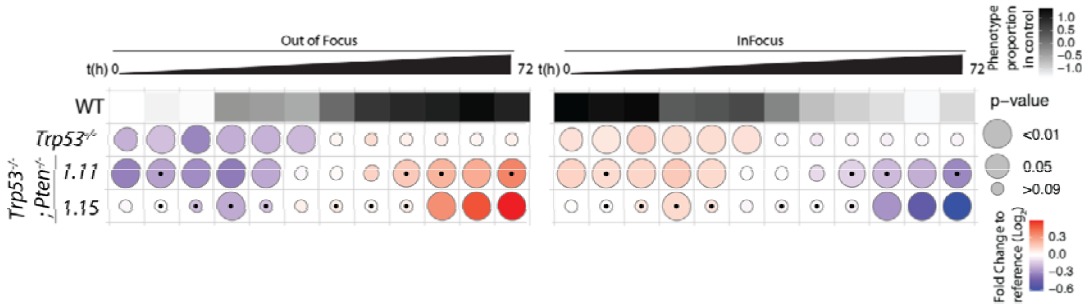

Our discussion now includes the following text:

"For consistency and clarity, we excluded from our analyses objects that were either out of focus during imaging or had completely invaded to the bottom of the dish. This ensured that objects that could not be imaged properly would not be improperly segmented and thus erroneously classified as either Spherical or Hyper-protrusive. Further, the highly invasive cells that sometimes invaded from early time points were almost exclusively found in the *Trp53$^{-/-}$;Pten$^{-/-}$* spheroids (across all clones). While we cannot exclude the possibility that this exclusion may lead to some under-estimation on the magnitude of the effect of PTEN loss, we felt that since the effect was clear even upon exclusion, this was a more honest way of performing our analyses as it allowed for more accurate classification of 3D structures."

4) Can the authors clarify how a 0 fold change (in log value) in fig 2D can be highly significant?

We believe that the reviewer is equating statistical significance with something being biologically meaningful. Statistical analysis does not indicate *a priori* whether something is biologically meaningful. Rather, it assesses the likelihood that an observed result is occurring by chance (or not). For instance, if a small change (e.g 0.04 in a log2 fold change) occurred repeatedly across experimental replicates this is unlikely to be a result of chance, and therefore could be statistically significant. Yet, such a small magnitude of effect is probably biologically minor. This is why our heatmaps provide both statistical significance, fold change, and consistency in magnitude of effect.

5) Delta isoform of PI3K seems to have an effect on area in the middle of the experiment, but has no effect at all on invasion. Could the authors comment? Are these smaller cysts still as invasive? There might be an interesting uncoupling between proliferation and invasion there.

The cysts are actually slightly larger with PI3Kδ inhibition and there is no change in invasion. We have expanded our comments in text to account for this observation:

The text now reads: " Interestingly, PI3Kd inhibition resulted in a transient elevation of spheroid size that nonetheless did not change the Hyper-protrusive behaviour, suggesting an uncoupling between proliferation and invasiveness under these conditions. Notably, PI3Kβ was found localised at the tips of the invasive protrusions (white/black arrowheads), prior to F-actin (yellow arrowheads), in *Trp53*$^{-/-}$;*Pten*$^{-/-}$ spheroids (Fig. 3K,L). This mirrors pAKT (S473) and PIP_3 localisation to the immediate tip of protrusions (Fig. 3A,B; Fig. EV 3E,F)."

6) ITGB1 depletion seems to induce a downregulation of Akt protein. Is that right? Does it change Akt localisation? Is there a dose effect whereby there is not enough Akt protein to mediate invasion?

The p:t AKT ratio does not change consistently across all gRNAs. When the total AKT intensity is quantified, we see a statistically significant but very minor (~13%) reduction in one of the KO cell lines. We have included this information in the updated manuscript but do not believe that such a minor change, as determined by Western blot, and in only one of the KO lines, will have any biological meaning.

7) Stats should be added directly on the graphs for the recycling assays, doing a pairwise comparison of the different genotypes for each time points. Can the authors clarify what the t-32min quantification graphs adds (fig7E, supp fig S8G-I)? I would advise to remove them, as this data is already presented in the recycling assay graphs.

We don't include these because although they are technical replicates, they are demonstrative of a single experiment. What we include instead is the quantitation across independent biological experiments (which each have their own internal multiple technical replicates), where it is appropriate to include statistical analysis.

8) There is a substantial amount of typos and erroneous references to figures. I listed below the ones that I spotted and I encourage the authors to carefully check.

a. there are some mistakes in referencing the number of cysts in supp table 1. There is for example no cysts experiments in Figure 1 but yet there are some references to figure 1 in supp table 1. Please correct it. I think it will be easier for the reader if the number of cysts quantified for each conditions was also indicated in the figure legends. Supp table 1 can still be included for readers that want additional details.

b. comma missing page 3

c. page 3 and 4: PI(3,4)2 means PI(3,4)P2? Can be shorten to PIP2 for ease of read and specify if it is another PIP2 specie otherwise

This has now been corrected to PI(3,4)P2.

d. define CYTH abbreviation: I suppose this is for cytohesin?

This has now been corrected

e. fig1F-I: don't understand why TCGA.OV is specified on some but not all the graphs. It seems to me that all the data are from TCGA.OV? Makes it seems it is nit the case

This has now been corrected

f. legend of fig1H, I: y axis is -Log10 values in 1I, not Log10 values

This has now been corrected

g. page 6: dKO abbreviation is already specified above and should be used to avoid repetition and for ease of read

This has now been corrected

h. supp fig S1D: missing legend for the second bar (after Wild Type)

This has now been corrected

i. supp fig S1N: legend of the X-axis should be below the axis

This has now been corrected

j. supp fig S1O: the numerotation of the X-axis needs to be below the line of the axis for ease of read, not above it

This has now been corrected

k. legend of S2A: clones 1.12 and 1.15 are p53-/-;PTEN-/- and not PTEN-/-

This has now been corrected

l. supp figS2C can the authors specify the different stages of matrigel (liquid or gel) that are used for the invasion assay, to make it easier for the non-specialist to understand what is going on. Please confirm that the 50% GFR matrigel makes a gel on top of the cells and fill in the wound to produce the 3D invasion assay setup.

This has now been corrected.

m. page 7: no parental cells are used in S3A, B only p53 null and p53 null and dKO. Please also specify what cells are being compared in the text

(...) In poorly protrusive *Trp53*$^{-/-}$ spheroids, probes for PI(4,5)P_2 (mNeonGreen [mNG]-tagged PH-PLCδ1) and PIP_3 (mNG-PH-CYTH3^{2G}) localised cortically, as well as in the nucleus in the case of PIP_3 (Fig. 3A). In wounded invasive monolayers of *Trp53*$^{-/-}$ cells, PI(4,5)P_2 and PIP_3 were not obviously enriched at protrusion tips (Fig. EV3A-D, arrowheads). However, in *Trp53*$^{-/-}$*;Pten*$^{-/-}$ dKO cells, a pool of PIP_3 was prominently located to the tips of protrusions in both spheroids and invasive monolayers (Fig. 3A-B, EV 3C-D, arrowheads). Accordingly, the tips of the invasive protrusions in the *Trp53*$^{-/-}$*;Pten*$^{-/-}$ spheroids were highly enriched for the PIP_3 effector pAKT (S473), prior to F-actin enrichment (Figure 3V E,F, arrowheads). (...)

n. description of arrow heads and colours need to be moved to figure legends and not in main text (page 7)

This has now been corrected.

o. fig 2D: the signification of the dot in the circles needs to be in the legends (since it is its first apparition in the manuscript). It only appears later on, in supp2A legend. Additional description of the matrices is necessary, as they contain a lot of information to digest to understand fully what is going on

The description has now been moved from S2A to 2D.

p. legend of fig3: error in figure reference: area data is D and not E, protrusive phenotypes are E and not F

This has now been corrected.

q. arrow missing in fig3B

This has now been corrected.

r. fig 3D,E, G, H: please indicate the cell line studied

This has now been corrected.

s. fig 3I: the different genotypes need to be stated on the galleries for clarity

This has now been corrected.

t. page 8: define Arf6-mNG in the text

This has now been corrected.

u. page 9: "<" symbol should be an alpha symbol

This has now been corrected.

v. fig 4A: indicate the cell line used on the figure

This has now been corrected.

w. supp fig S4E: why is it specified mouse-specific for the shArf6?

This was for historical reasons, as the shRNAs used in the specific figure panel were targeting the mouse Arf6 transcript, whereas the shRNA described in S4A and S4B can target both human and mouse. This information has now been removed from Figure EV 4E to avoid confusion.

x. 4H, I, J: indicate on the figure if these interactors are mostly unchanged, strong interactors or weak interactors for clarity

This has now been corrected.

y. legend of fig4H: "coloured spots underneath denote the protein complex that each interactor belongs (in J)" should indicate panel G and not J

This has now been corrected according to the new order of figure panels.

z. fig4I, J: are you sure of the legend for the fold change coloring? Log2 of 1 is a 0 fold change, i don't see how these could show any significant difference (i.e. some of the pale red circles are significant)

We apologise for the error in our label. The values correspond to the difference in the Log2 transformed LFQ value compared to TurboID alone (Fig4H) or WT ARF6-Turbo ID (Fig4I and J). The labelling has been corrected.

aa. page 11: description of the assay (starting with "Machine learning classification of...") is very confusing, please clarify

The figure legend for panel 2C has been changed in the revised manuscript to now read:

" (…)((2) Based on the measurements obtained from live imaging for each individual spheroid, we used CellProfiler Analyst and user-supervised machine learning (FastGentle Boosting algorithm) to construct rules based on which the objects would be classified as 'In Focus' or 'Out-of-focus' (3). The later were filtered out of the dataset. (4) (…)"

We have avoided to include additional information as this method is more extensively described in our Materials and Methods section and our lab's recent paper which we have cited in the Results section.

bb. page16: figure 4H should be 4I (PTEN-null specific association of Arf6 with ITGA5)

This has been corrected.

cc. supp fig S5H-P: choose Tumour or cancer to homogeneise naming across the graphs

This has been homogenised to Cancer.

dd. fig 5H: box are difficultly visible in green, change color to yellow or something more visible

The boxes are now in red.

ee. page 13: Fig6E, F should also refer to 6G

This has been corrected.

ff. LCM abbreviation on page 10 and 12 refers to LCMD? Otherwise please define it.

This has been changed.

OPTIONAL SUGGESTIONS

1) Choice of cell line: There is a high number of patients (around 9% according to (Cole et al. 2016)) that present the R248Q gain-of-function mutation. A recent study has shown that this mutant p53 protein is associated to an activation of Akt signalling and an increase of the intercellular trafficking of EGFR (Lai et al. 2021). Given that EGFR was also a hit in this screen, that is seems to have a central role in Arf6 cargoes (fig 4G), I think it would be a great addition to this study. It could hence cooperate with PTEN loss to drive strong, robust invasion.

This is an excellent observation and one we will likely follow-up in an independent study.

2) Are MAPK involved in the PTEN KO pro-invasive phenotype? In particular Erk1/2, since EGFR is one of the PTEN loss induced Arf6 cargoes.

This is an excellent observation and one we will likely follow-up in an independent study.

REFERENCE

Cole, Alexander J., Trisha Dwight, Anthony J. Gill, Kristie-Ann Dickson, Ying Zhu, Adele Clarkson, Gregory B. Gard, et al. 2016. « Assessing Mutant P53 in Primary High-Grade Serous Ovarian Cancer Using Immunohistochemistry and Massively Parallel Sequencing ». Scientific Reports 6 (1): 26191. https://doi.org/10.1038/srep26191.

Lai, Zih-Yin, Kai-Yun Tsai, Shing-Jyh Chang, et Yung-Jen Chuang. 2021. « Gain-of-Function Mutant TP53 R248Q Overexpressed in Epithelial Ovarian Carcinoma Alters AKT-Dependent Regulation of Intercellular Trafficking in Responses to EGFR/MDM2 Inhibitor ». International Journal of Molecular Sciences 22 (16): 8784. https://doi.org/10.3390/ijms22168784.

Reviewer #2 (Evidence, reproducibility and clarity (Required)):

The authors have conducted a study of the molecular requirements for cancer invasion that is stunning in its thoroughness and depth and breadth of its molecular analysis. The writing is exceptionally precise though also very dense (see below). The molecular model proposed is that PTEN loss (in a p53 null background) leads to reliance upon ARF6 for invasion, with regulation through interactions with AGAP1 and beta1-integrin and it is convincingly demonstrated. They focus on interpreting the consequences of genetic and pharmacologic manipulations in a cell line, using a series of 2D and 3D assays. The phenotypes are more prominent in 3D assays.

Concerns and Suggestions:

1. There is a disconnect between the essentially complete loss of protrusions and invasion in 3D (e.g. 4A) and the reduction in magnitude of protrusive invasion but the continued presence of elongated cells with protrusions in 2D (e.g. S4C). This discrepancy is present in a couple of comparisons and is glossed over in quick callouts to many figure panels.

We thank the reviewer for mentioning this as this comment was very helpful in determining that we needed to clarify our description of the role of ARF6 to protrusion formation vs maturation. In the Trp53$^{-/-}$ genotype, protrusions can form, but they rapidly retract, failing to mature into structures that drive invasion through ECM (e.g. Figure EV 2E). This protrusion maturation occurs upon PTEN KO. When ARF6 depleted, PTEN-null cells can form protrusions, but now again lack the ability to mature into invasion-inducing structures.

This concept of needing ARF6 for protrusion maturation and maintenance is underpinned by our model of ARF6 regulating recycling of active integrin back to the protrusion front. Indeed, we have observed ARF6 being required not for protrusion initiation, but rather ensuring protrusions are not retracted in other contexts (i.e. upon loss of the ARF6 GEF protein IQSEC1 in invading 3D culture of PC3 cells; PMID: 33712589).

We also note that, as responded to Reviewer 1, the assay is a 3D invasion rather than 2D migration assay, with cells sandwiched between Matrigel.

The following has been added in the Discussion section of our manuscript:

"It should be highlighted that our observations herein and the model we are suggesting do not imply that ARF6 is required for invasion initiation, but rather for invasion maturation and persistence. Indeed, upon ARF6 depletion, PTEN-null spheroids often exhibited the formation of fine, transient protrusions. In most cases, however, these were not enough to lead to the formation of a stable invasion structure. Similarly, ECM-embedded *Trp53*$^{-/-}$;*Pten*$^{-/-}$ ARF6 KD monolayers were still able to invade and eventually close the monolayer wound albeit with reduced efficiency compared to their ARF6-proficient counterparts. This mirrored the behaviour of both WT and *Trp53*$^{-/-}$ spheroids and monolayers. We reported a similar

function of ARF6 being regulating protrusion maturation rather than initiation in conjunction with the ARF GEF protein IQSEC1 in invading 3D cultures of prostate cancer cells (PC3)(Nacke et al., 2021)."

2. Once a journal has been identified, it would be wise for the editor to allow some flexibility in word limit to enable some very dense sections to be expanded slightly to guide the reader through the experiments and results more clearly. For example, in the section "ARF6 regulates active integrin pools...", there are callouts like (Fig. 7C,E; S8A-C; G-I) and then (Fig. 7D,E; Fig. S8E-F, H-I). It takes a lot of time to unpack these different experimental claims based on a single sentence.

We greatly appreciate the refreshing comments of this reviewer to advocate for actions to improve clarity in our reporting.

We have know updated the relevant sections in an attempt to make our explanation more clear. The new section now reads:

"ARF6 regulates active integrin pools to produce invasive protrusions.

Our data thus far indicate that a CYTH2-ARF6-AGAP1 module is required for invasion in *Pten*-null cells, and that α5-integrin and β1-integrin are two ARF6-promixal proteins essential for this phenotype. Although the mRNA levels of these two integrins are not altered in normal versus tumour epithelium, nor do they stratify patient survival based on a medium split (Fig. EV 6K-L), we explored whether the ARF6 module may act by regulating distribution of ECM-adhesion complexes to the tips of protrusions to drive invasion. In addition to cortical localisation, both a5-integrin and β1-integrin showed localisation to the extreme tips of invasive protrusions in *Trp53*$^{-/-}$;*Pten*$^{-/-}$ spheroids, occurring prior to F-actin enrichment and mirroring the localisation observed for both pAKT (S473) and PIP3 (Fig. 7A-B). Two markers of ECM-signalling hubs, pY397-FAK and pY416-Src family kinases (SFK), also localised prominently to the tips of protrusions (Fig. 7C-D), in addition to the cell-ECM interface. This observation suggests a pool of integrin signalling complexes localise to protrusion tips.

In ovarian cancer patients with low levels of PTEN protein, pY416-SFK protein levels were elevated (Fig. 1I). To interrogate whether loss of PTEN may be associated with alterations in membrane trafficking of these integrins, we used a captured-based ELISA approach(Roberts et al., 2001). In *Trp53*$^{-/-}$;*Pten*$^{-/-}$ cells, the recycling of internalised total a5-

or β1-integrin, the active form of β1-integrin, or a control cargo of Transferrin Receptor (TfnR), was increased at all timepoints examined compared to *Trp53*$^{-/-}$ cells. This increase reached statistical significance ($p<0.05$) at t=32min for active β1 integrin (Fig. 7E-F; Fig. EV 8A-F).

To determine whether ARF6 regulates this enhanced recycling in *Trp53*$^{-/-}$*;Pten*$^{-/-}$ cells we depleted *Arf6*. *Arf6* depletion in *Trp53*$^{-/-}$*;Pten*$^{-/-}$ cells specifically blunted recycling of active form of β1-integrin, but not of total a5-integrin, β1-integrin or TfnR (Fig. 7G-H; Fig.EV 8G-K). This suggests that the CYTH2-ARF6-AGAP1 module specifically regulates recycling of the active β1-integrin, whilst trafficking of inactive b1-integrins and TfnR is controlled by other signalling modules downstream of PIP_3."

3. The patient data on CYTH2 and its relationship to survival is modestly convincing.

In Ovarian Cancer, effects on survival are often minor. This is not a disease where one often sees large shifts in survival, which is why we are so excited about the large shifts that we do see with the ARF GTPase module we identified. However, we concede that the effects on CYTH2, although significant, are not vast changes.

The following has now been added in the Discussion section:

"(…) however CYTH2 contribution is complicated by poor outcome being specifically conferred by the PIP_3-associating CYTH2^{2G} isoform. While the effect of CYTH2^{2G} (PIP_3-binding variant on survival is modest (3-month decrease when CYTH2^{2G} is high), combining this with high ARF6 levels allows identification of a 10-month decrease in survival."

4. Very minor- search on %- there are a few inconsistencies in terms of spaces and commas vs. periods. The Methods also have some inconsistencies in terms of spaces between numbers and units or numbers and degrees Celsius. References are also in a different font. Overall it was extremely carefully written though (just dense).

This has now been corrected.

Reviewer #2 (Significance (Required)):

One limitation of the experimental design is that the depth of molecular analysis in vitro comes at the expense of any in vivo validation, which the authors acknowledge in the Discussion. They attempt to make similar points using analysis of patient survival data from public databases but these analyses generally yielded small magnitude differences. The main audience for this study is likely to be cell biologists interested in cell migration, cell-ECM adhesion, cancer invasion, and GTPases. I don't see any need for new

experiments- what can be done has been done and then some. I do think that it would benefit readers if the text could be made less dense.

Reviewer #3 (Evidence, reproducibility and clarity (Required)):

Summary:
Using a murine HGSOC 3D cell model, in combination with analysis of human ovarian cancer datasets, the authors uncover a CYTH2-ARF6-AGAP1 signaling module regulated by PTEN and identify a biomarker for tumor invasion and targeted therapy.

Major comments:
The findings of this study are significant as they reveal a critical signaling module that controls tumor invasion by mediating tumor cell interaction with the extracellular matrix. The experiments are properly designed, and the data are well presented. The conclusions are appropriate and supported by the data. The limitation of the study has also been discussed properly.

One suggestion regarding the survival analysis in Fig. 6 and 7.
The authors noted that the CYTH2-ARF6-AGAP1 module is not specifically or only induced in Pten-null contexts, but rather that Pten-null cells become more dependent on the module for enacting the invasive phenotype. Based on this, it would be interesting to evaluate how the PTEN status impacts the survival difference by integrating the PTEN genomic status (WT versus mutation) or its expression level (protein or mRNA) into the survival analysis of patient cohorts in Fig. 6 and Fig. 7.

We thank the reviewer for this excellent point. We now add the exciting additional finding (new data Fig. 7K) that the poorer overall survival outcome observed for patients with low PTEN protein levels (Fig. 1G) requires simultaneous high levels of each of CYTH2-ARF6-AGAP1 mRNA. Conversely, patients with low PTEN protein levels and low simultaneous levels of each of CYTH2-ARF6-AGAP1 mRNA (new data Fig. 7K) had indistinguishable survival from patients with high PTEN protein levels.

Individually, or in pairs, each of ARF6 mRNA, CYTH2 (mRNA or Exon 9.1 splicing), or AGAP1 (mRNA or splicing) did not significantly alter the survival of PTEN low patients. This emphasises a role for this co-acting GEF-ARF-GAP module in PTEN-low phenotypes.

In the TCGA cohort, only 7 patients possessed mutations in PTEN, while there are 578 non-PTEN mutant patients. This low number of mutant PTEN cases demonstrated no difference in patient survival, clinical features, or mRNA expression of ARF module members being significant between WT vs mutant PTEN patients.

We only include the significant new data (new data Fig. 7K) in the revised manuscript. We do not include the many comparisons which resulted in non-significant changes due to not wishing to further add to the density of the manuscript (as described by Reviewer 2).

The final paragraph of the Results section in page 15 now reads:

"(..) Crucially, the poor outcome associated with patients with low PTEN protein levels (Fig 1G) requires simultaneous high mRNA levels of each of the three ARF module components

(CYTH2-ARF6-AGAP1) as patients with low PTEN protein levels and low mRNA levels of each of CYTH2-ARF6-AGAP1 had indistinguishable outcome to high PTEN protein patients (Figure 7K). This resulted in a 23-month difference in survival in patients with low PTEN protein and high ARF module (blue line) compared to high PTEN levels (green line) (Fig. 7K). (…)”

Referees cross-commenting

Gene deletion and mutation may elicit different functional outcomes. I therefore agree with Reviewer #1 that "the choice of studying PTEN loss in the complete absence of p53, a situation that does not mirror the clinical situation, needs to be explained".

We will make our reasons for this choice clear in the text before submission. Please refer to response to Reviewer 1, Major comment 1.

Reviewer #3 (Significance (Required)):

The model used and data presented in this study are of significance for both scientific discovery and clinical application, which will interest the broad audience in both basic and clinical research.

Reviewer-only file, percentage of Hyper-protrusive and Spherical spheroids across time points in Wild Type and Trp53-/-;Pten-/- 1.15 spheroids

Hyperprotrusive:	Genotype	Time Interval	Count	Percentage of phenotype	Spherical:	Genotype	Time Interval	Count	Percentage of phenotype
Expt1	WILD TYPE	6	26	7.12328767		WILD TYPE	6	339	92.87671233
	WILD TYPE	12	24	4.46096654		WILD TYPE	12	514	95.53903346
	WILD TYPE	18	46	7.37179487		WILD TYPE	18	578	92.62820513
	WILD TYPE	24	39	6.28019324		WILD TYPE	24	582	93.71980676
	WILD TYPE	30	52	8.68113523		WILD TYPE	30	547	91.31886477
	WILD TYPE	36	64	10		WILD TYPE	36	576	90
	WILD TYPE	42	66	10.8731466		WILD TYPE	42	541	89.12685338
	WILD TYPE	48	64	11.4285714		WILD TYPE	48	496	88.57142857
	WILD TYPE	54	49	8.16666667		WILD TYPE	54	551	91.83333333
	WILD TYPE	60	31	4.96		WILD TYPE	60	594	95.04
	WILD TYPE	66	34	5.34591195		WILD TYPE	66	602	94.65408805
	WILD TYPE	72	31	4.79134467		WILD TYPE	72	616	95.20865533
Expt2	WILD TYPE	6	15	4.80769231		WILD TYPE	6	297	95.19230769
	WILD TYPE	12	39	7.87878788		WILD TYPE	12	456	92.12121212
	WILD TYPE	18	49	9.5703125		WILD TYPE	18	463	90.4296875
	WILD TYPE	24	20	4.33839479		WILD TYPE	24	441	95.66160521
	WILD TYPE	30	27	5.41082164		WILD TYPE	30	472	94.58917836
	WILD TYPE	36	46	9.48453608		WILD TYPE	36	439	90.51546392
	WILD TYPE	42	30	6.3559322		WILD TYPE	42	442	93.6440678
	WILD TYPE	48	28	5.95744681		WILD TYPE	48	442	94.04255319
	WILD TYPE	54	15	3.17124736		WILD TYPE	54	458	96.82875264
	WILD TYPE	60	17	3.76940133		WILD TYPE	60	434	96.23059867
	WILD TYPE	66	19	3.83064516		WILD TYPE	66	477	96.16935484
	WILD TYPE	72	23	4.74226804		WILD TYPE	72	462	95.25773196
Expt3	WILD TYPE	6	84	5.72207085		WILD TYPE	6	1384	94.27792916
	WILD TYPE	12	95	7.31331794		WILD TYPE	12	1204	92.68668206
	WILD TYPE	18	78	5.98618573		WILD TYPE	18	1225	94.01381427
	WILD TYPE	24	128	9.9921936		WILD TYPE	24	1153	90.0078064
	WILD TYPE	30	140	11.2540193		WILD TYPE	30	1104	88.74598071
	WILD TYPE	36	143	11.3402062		WILD TYPE	36	1118	88.65979381
	WILD TYPE	42	188	15.7322176		WILD TYPE	42	1007	84.26778243
	WILD TYPE	48	222	19.7684773		WILD TYPE	48	901	80.23152271
	WILD TYPE	54	226	21.0624418		WILD TYPE	54	847	78.93755825
	WILD TYPE	60	215	21.037182		WILD TYPE	60	807	78.962818
	WILD TYPE	66	214	21.8813906		WILD TYPE	66	764	78.11860941
	WILD TYPE	72	203	20.4225352		WILD TYPE	72	791	79.57746479

Hyperprotrusive:	Genotype	Time Interval	Count	Percentage of phenotype	Spherica:l	Genotype	Time Interval	Count	Percentage of phenotype
Expt1	PTEN 115	6	13	4.81481482		PTEN 115	6	257	95.18518519
	PTEN 115	12	21	8.53658537		PTEN 115	12	225	91.46341463
	PTEN 115	18	39	14.2857143		PTEN 115	18	234	85.71428571
	PTEN 115	24	84	30.7692308		PTEN 115	24	189	69.23076923
	PTEN 115	30	143	58.1300813		PTEN 115	30	103	41.8699187
	PTEN 115	36	150	70.754717		PTEN 115	36	62	29.24528302

	PTEN 115	42	171	84.6534654		PTEN 115	42	31	15.34653465
	PTEN 115	48	179	88.6138614		PTEN 115	48	23	11.38613861
	PTEN 115	54	129	82.1656051		PTEN 115	54	28	17.8343949
	PTEN 115	60	73	78.4946237		PTEN 115	60	20	21.50537634
	PTEN 115	66	56	73.6842105		PTEN 115	66	20	26.31578947
	PTEN 115	72	56	74.6666667		PTEN 115	72	19	25.33333333
Expt2	PTEN 115	6	21	7.1672355		PTEN 115	6	272	92.83276451
	PTEN 115	12	48	15.3846154		PTEN 115	12	264	84.61538462
	PTEN 115	18	75	24.5901639		PTEN 115	18	230	75.40983607
	PTEN 115	24	115	36.3924051		PTEN 115	24	201	63.60759494
	PTEN 115	30	132	41.9047619		PTEN 115	30	183	58.0952381
	PTEN 115	36	171	54.6325879		PTEN 115	36	142	45.36741214
	PTEN 115	42	174	61.2676056		PTEN 115	42	110	38.73239437
	PTEN 115	48	152	54.8736462		PTEN 115	48	125	45.12635379
	PTEN 115	54	178	68.9922481		PTEN 115	54	80	31.00775194
	PTEN 115	60	185	70.610687		PTEN 115	60	77	29.38931298
	PTEN 115	66	169	67.6		PTEN 115	66	81	32.4
	PTEN 115	72	164	73.5426009		PTEN 115	72	59	26.4573991
Expt3	PTEN 115	6	136	15.5963303		PTEN 115	6	736	84.40366972
	PTEN 115	12	171	19.9533256		PTEN 115	12	686	80.04667445
	PTEN 115	18	260	31.6686967		PTEN 115	18	561	68.33130329
	PTEN 115	24	376	45.8536585		PTEN 115	24	444	54.14634146
	PTEN 115	30	452	57.287706		PTEN 115	30	337	42.71229404
	PTEN 115	36	421	63.3082707		PTEN 115	36	244	36.69172932
	PTEN 115	42	479	77.0096463		PTEN 115	42	143	22.9903537
	PTEN 115	48	415	89.6328294		PTEN 115	48	48	10.36717063
	PTEN 115	54	357	91.7737789		PTEN 115	54	32	8.22622108
	PTEN 115	60	222	96.9432314		PTEN 115	60	7	3.056768559
	PTEN 115	66	125	96.8992248		PTEN 115	66	4	3.100775194
	PTEN 115	72	83	96.5116279		PTEN 115	72	3	3.488372093

Dear Dr David Bryant,

Thank you for submitting your revised manuscript (EMBOJ-2023-113987R) to The EMBO Journal. Your amended study was sent back to the three referees for re-evaluation, and we have received comments from two of them, which I enclose below. Please note that while reviewer #1 was at this time not able to give additional input, we have analyzed your response to his/her concerns editorially and found that the raised issues to be satisfactorily addressed. As you will see, the other experts stated that the work has been substantially improved by the revisions and they are now broadly in favour of publication.

Thus, we are pleased to inform you that your manuscript has been accepted in principle for publication in The EMBO Journal.

We now still need you to take care of a number of issues related to formatting and data representation as detailed below, which should be addressed at re-submission.

Please contact me at any time if you have additional questions related to below points.

As you might have seen on our web page, every paper at the EMBO Journal now includes a 'Synopsis', displayed on the html and freely accessible to all readers. The synopsis includes a 'model' figure as well as 2-5 one-short-sentence bullet points that summarize the article. I would appreciate if you could provide this figure and the bullet points.

Thank you for giving us the chance to consider your manuscript for The EMBO Journal. I look forward to your final revision.

Again, please contact me at any time if you need any help or have further questions.

Best regards,

Daniel Klimmeck

Daniel Klimmeck PhD
Senior Editor
The EMBO Journal

Formatting changes required for the revised version of the manuscript:

>> Please add up to five keywords to your manuscript.

>> Adjust the title of the 'Competing Interests statement' section to 'Disclosure and Competing Interests Statement'.

>> Author Contributions: Remove the author contributions information from the manuscript text. Note that CRediT has replaced the traditional author contributions section as of now because it offers a systematic machine-readable author contributions format that allows for more effective research assessment. and use the free text boxes beneath each contributing author's name to add specific details on the author's contribution.

>> Figure callouts: Fig. 4E should be called out before Fig. 4F, Fig. 5F should be called out before Fig. 5G; should it be Fig. 5B-C instead of Fig. 5C-B? please clarify.

>> The introduction section should be labelled with a separate header in the manuscript.

>> Manuscript order: please adjust the order of the sections in the manuscript: abstract, introduction, results, discussion, materials & methods, data availability section, acknowledgments, disclosure statement and competing interests, references, main figure legends, tables, expanded figure legends .

>> Funding information: please complement our online manuscript system with the complete funding details; currently missing: Essen Bioscience.

>> Reference format: please add 'et al' for the entries that have more than 10 authors.

>> Dataset EV legends: legends should be removed from manuscript file, and uploaded as separate tabs in each Excel file.

>> Author Checklist: please complete the author checklist by adding information regarding corresponding author name, journal and manuscript ID.

>> 'Data availability statement' section: adjust the title to 'Data Availability' and add a hyperlink to the SRA and PRIDE database entries; make sure to release privacy from the PRIDE data.

>> We only allow 5 EV Figures; the rest should be provided in an Appendix .pdf file (with a ToC on its first page) and renamed to "Appendix Figure S1", etc. their corresponding legends should also be in the Appendix file and the callouts in the manuscript should be updated accordingly .

>>Change the 'Experimental design' paragraph to a separate 'Statistical analysis' section, detailing the algorithms applied.

>>Movies: movie legends should be removed from the main manuscript file and zipped with each movie file using the nomenclature "Movie EV1..." .

>> Consider additional changes and comments from our production team as indicated by the .doc file enclosed and leave changes in track mode.

****

Further information is available in our Guide For Authors: https://www.embopress.org/page/journal/14602075/authorguide

----

Referee #2:

My concerns, and those of the other reviewers, were addressed by the authors in a comprehensive fashion. I am fully supportive of publication in EMBO Journal.

Referee #3:

The authors have fully addressed my comments and suggestions, which led to new findings. I am glad to see such outcome of the review process. The manuscript is now acceptable for publication.

The authors have addressed all minor editorial requests.

2nd Revision - Editorial Decision 19th Jul 2023

Dear Dr David Bryant,

Thank you for submitting the revised version of your manuscript. I have now evaluated your amended manuscript and concluded that the remaining minor concerns have been sufficiently addressed.

Thus, I am pleased to inform you that your manuscript has been accepted for publication in the EMBO Journal.

Please note that it is EMBO Journal policy for the transcript of the editorial process (containing referee reports and your response letter) to be published as an online supplement to each paper. I would thus like to ask for your consent on keeping the additional referee figure included in this file.

Also, in case you might NOT want the transparent process file published at all, you will also need to inform us via email immediately. More information is available here:
https://www.embopress.org/page/journal/14602075/authorguide#transparentprocess

----

Please note that in order to be able to start the production process, our publisher will need and contact you shortly regarding the page charge authorisation and licence to publish forms.

Authors of accepted peer-reviewed original research articles may choose to pay a fee in order for their published article to be made freely accessible to all online immediately upon publication. The EMBO Open Access fee is fixed at $6,540 USD / £5,310 GBP / €5,900 EUR (+ VAT where applicable).

We offer two licenses for Open Access papers, CC-BY and CC-BY-NC-ND.
For more information on these licenses, please visit:
http://creativecommons.org/licenses/by-nc-nd/3.0/deed.en_US

Should you be planning a Press Release on your article, please get in contact with embojournal@wiley.com as early as possible, in order to coordinate publication and release dates.

On a different note, I would like to alert you that EMBO Press is currently developing a new format for a video-synopsis of work published with us, which essentially is a short, author-generated film explaining the core findings in hand drawings, and, as we believe, can be very useful to increase visibility of the work. This has proven to offer a nice opportunity for exposure i.p. for the first author(s) of the study. Please see the following link for representative examples and their integration into the article web page:
https://www.embopress.org/video_synopses
https://www.embopress.org/doi/full/10.15252/embj.2019103932

Please let me know, should you be interested to engage in commissioning a similar video synopsis for your work. According operation instructions are available and intuitive.

Finally, we have noted that the submitted version of your article is also posted on the preprint platform bioRxiv. We would appreciate if you could alert bioRxiv on the acceptance of this manuscript at The EMBO Journal in order to allow for an update of the entry status. Thank you in advance!

If you have any questions, please do not hesitate to call or email the Editorial Office.

Thank you again for this contribution to The EMBO Journal and congratulations on a successful publication! Please consider us again in the future for your most exciting work.

Best regards,

Daniel Klimmeck

Daniel Klimmeck, PhD
Senior Editor

The EMBO Journal
EMBO
Postfach 1022-40
Meyerhofstrasse 1
D-69117 Heidelberg
contact@embojournal.org

EMBO Press Author Checklist

Corresponding Author Name: David M. Bryant
Journal Submitted to: The EMBO Journal
Manuscript Number: EMBOJ-2023-113987R

USEFUL LINKS FOR COMPLETING THIS FORM
The EMBO Journal - Author Guidelines
EMBO Reports - Author Guidelines
Molecular Systems Biology - Author Guidelines
EMBO Molecular Medicine - Author Guidelines

Reporting Checklist for Life Science Articles (updated January
This checklist is adapted from Materials Design Analysis Reporting (MDAR) Checklist for Authors. MDAR establishes a minimum set of requirements in transparent reporting in the life sciences (see Statement of Task: 10.31222/osf.io/9sm4x). Please follow the journal's guidelines in preparing your manuscript.
Please note that a copy of this checklist will be published alongside your article.

Abridged guidelines for figures
1. Data
The data shown in figures should satisfy the following conditions:
- the data were obtained and processed according to the field's best practice and are presented to reflect the results of the experiments in an accurate and unbiased manner.
- ideally, figure panels should include only measurements that are directly comparable to each other and obtained with the same assay.
- plots include clearly labeled error bars for independent experiments and sample sizes. Unless justified, error bars should not be shown for technical
- if n<5, the individual data points from each experiment should be plotted. Any statistical test employed should be justified.
- Source Data should be included to report the data underlying figures according to the guidelines set out in the authorship guidelines on Data

2. Captions
Each figure caption should contain the following information, for each panel where they are relevant:
- a specification of the experimental system investigated (eg cell line, species name).
- the assay(s) and method(s) used to carry out the reported observations and measurements.
- an explicit mention of the biological and chemical entity(ies) that are being measured.
- an explicit mention of the biological and chemical entity(ies) that are altered/varied/perturbed in a controlled manner.
- the exact sample size (n) for each experimental group/condition, given as a number, not a range;
- a description of the sample collection allowing the reader to understand whether the samples represent technical or biological replicates (including how many animals, litters, cultures, etc.).
- a statement of how many times the experiment shown was independently replicated in the laboratory.
- definitions of statistical methods and measures:
  - common tests, such as t-test (please specify whether paired vs. unpaired), simple χ2 tests, Wilcoxon and Mann-Whitney tests, can be unambiguously identified by name only, but more complex techniques should be described in the methods section;
  - are tests one-sided or two-sided?
  - are there adjustments for multiple comparisons?
  - exact statistical test results, e.g., P values = x but not P values < x;
  - definition of 'center values' as median or average;
  - definition of error bars as s.d. or s.e.m.

Please complete ALL of the questions below.
Select "Not Applicable" only when the requested information is not relevant for your study.

Materials

Newly Created Materials	Information included in the manuscript?	In which section is the information available? (Reagents and Tools Table, Materials and Methods, Figures, Data Availability Section)
New materials and reagents need to be available; do any restrictions apply?	Yes	Materials and Methods/ Supplementry Tables

Antibodies	Information included in the manuscript?	In which section is the information available? (Reagents and Tools Table, Materials and Methods, Figures, Data Availability Section)
For **antibodies** provide the following information:<br>- Commercial antibodies: RRID (if possible) or supplier name, catalogue number and or/clone number<br>- Non-commercial: RRID or citation	Yes	Materials and Methods

DNA and RNA sequences	Information included in the manuscript?	In which section is the information available? (Reagents and Tools Table, Materials and Methods, Figures, Data Availability Section)
**Short novel DNA or RNA including primers, probes**: provide the sequences.	Yes	Supplementary Information and Materials and Methods

Cell materials	Information included in the manuscript?	In which section is the information available? (Reagents and Tools Table, Materials and Methods, Figures, Data Availability Section)
**Cell lines:** Provide species information, strain. Provide accession number in repository **OR** supplier name, catalog number, clone number, and/**OR** RRID.	Yes	Materials and Methods
**Primary cultures:** Provide species, strain, sex of origin, genetic modification status.	Not Applicable	
Report if the cell lines were recently **authenticated** (e.g., by STR profiling) and tested for mycoplasma contamination.	Yes	Materials and Methods

Experimental animals	Information included in the manuscript?	In which section is the information available? (Reagents and Tools Table, Materials and Methods, Figures, Data Availability Section)
**Laboratory animals or Model organisms:** Provide species, strain, sex, age, genetic modification status. Provide accession number in repository **OR** supplier name, catalog number, clone number, **OR** RRID.	Not Applicable	
**Animal observed in or captured from the field:** Provide species, sex, and age where possible.	Not Applicable	
Please detail **housing and husbandry conditions**.	Not Applicable	

Plants and microbes	Information included in the manuscript?	In which section is the information available? (Reagents and Tools Table, Materials and Methods, Figures, Data Availability Section)
**Plants:** provide species and strain, ecotype and cultivar where relevant, unique accession number if available, and source (including location for collected wild specimens).	Not Applicable	
**Microbes:** provide species and strain, unique accession number if available, and source.	Not Applicable	

Human research participants	Information included in the manuscript?	In which section is the information available? (Reagents and Tools Table, Materials and Methods, Figures, Data Availability Section)
If collected and within the bounds of privacy constraints report on age, sex and gender or ethnicity for all study participants.	Not Applicable	

Core facilities	Information included in the manuscript?	In which section is the information available? (Reagents and Tools Table, Materials and Methods, Figures, Data Availability Section)
If your work benefited from core facilities, was their service mentioned in the acknowledgments section?	Yes	Acknowledgments

Design

Study protocol	Information included in the manuscript?	In which section is the information available? (Reagents and Tools Table, Materials and Methods, Figures, Data Availability Section)
If study protocol has been **pre-registered, provide DOI in the manuscript**. For clinical trials, provide the trial registration number **OR** cite DOI.	Not Applicable	
Report the **clinical trial registration number** (at ClinicalTrials.gov or equivalent), where applicable.	Not Applicable	

Laboratory protocol	Information included in the manuscript?	In which section is the information available? (Reagents and Tools Table, Materials and Methods, Figures, Data Availability Section)
Provide DOI OR other citation details if **external detailed step-by-step protocols** are available.	Not Applicable	

Experimental study design and statistics	Information included in the manuscript?	In which section is the information available? (Reagents and Tools Table, Materials and Methods, Figures, Data Availability Section)
Include a statement about **sample size** estimate even if no statistical methods were used.	Yes	Materials and Methods
Were any steps taken to minimize the effects of subjective bias when allocating animals/samples to treatment (e.g. **randomization procedure**)? If yes, have they been described?	Not Applicable	
Include a statement about **blinding** even if no blinding was done.	Yes	Materials and Methods
Describe **inclusion/exclusion criteria** if samples or animals were excluded from the analysis. Were the criteria pre-established?<br><br>If sample or data points were omitted from analysis, report if this was due to attrition or intentional exclusion and provide justification.	Yes	Materials and Methods/ Results/ Discussion
For every figure, are **statistical tests** justified as appropriate? Do the data meet the assumptions of the tests (e.g., normal distribution)? Describe any methods used to assess it. Is there an estimate of variation within each group of data? Is the variance similar between the groups that are being statistically compared?	Yes	Materials and Methods/ Figure Legends

Sample definition and in-laboratory replication	Information included in the manuscript?	In which section is the information available? (Reagents and Tools Table, Materials and Methods, Figures, Data Availability Section)
In the figure legends: state number of times the experiment was **replicated** in laboratory.	Yes	Figure legends
In the figure legends: define whether data describe **technical or biological replicates**.	Yes	Figure Legends

Ethics

Ethics	Information included in the manuscript?	In which section is the information available? (Reagents and Tools Table, Materials and Methods, Figures, Data Availability Section)
Studies involving **human participants**: State details of **authority granting ethics approval** (IRB or equivalent committee(s), provide reference number for approval.	Not Applicable	
Studies involving **human participants**: Include a statement confirming that **informed consent** was obtained from all subjects and that the experiments conformed to the principles set out in the WMA Declaration of Helsinki and the Department of Health and Human Services Belmont Report.	Not Applicable	
Studies involving **human participants:** For publication of **patient photos**, include a statement confirming that consent to publish was obtained.	Not Applicable	
Studies involving experimental **animals**: State details of **authority granting ethics approval** (IRB or equivalent committee(s), provide reference number for approval. Include a statement of compliance with ethical regulations.	Not Applicable	
Studies involving **specimen and field samples:** State if relevant **permits** obtained, provide details of authority approving study; if none were required, explain why.	Not Applicable	

Dual Use Research of Concern (DURC)	Information included in the manuscript?	In which section is the information available? (Reagents and Tools Table, Materials and Methods, Figures, Data Availability Section)
Could your study fall under dual use research restrictions? Please check biosecurity documents and list of **select agents and toxins** (CDC): https://www.selectagents.gov/sat/list.htm	Not Applicable	
If you used a select agent, is the security level of the lab appropriate and reported in the manuscript?	Not Applicable	
If a study is subject to dual use research of concern regulations, is the name of the **authority granting approval and reference number** for the regulatory approval provided in the manuscript?	Not Applicable	

Reporting

The MDAR framework recommends adoption of discipline-specific guidelines, established and endorsed through community initiatives. Journals have their own policy about requiring specific guidelines and recommendations to complement MDAR.

Adherence to community standards	Information included in the manuscript?	In which section is the information available? (Reagents and Tools Table, Materials and Methods, Figures, Data Availability Section)
State if relevant guidelines or checklists (e.g., **ICMJE, MIBBI, ARRIVE, PRISMA**) have been followed or provided.	Not Applicable	
For **tumor marker prognostic studies,** we recommend that you follow the **REMARK** reporting guidelines (see link list at top right). See author guidelines, under 'Reporting Guidelines'. Please confirm you have followed these guidelines.	Not Applicable	
For **phase II and III randomized controlled trials**, please refer to the **CONSORT** flow diagram (see link list at top right) and submit the CONSORT checklist (see link list at top right) with your submission. See author guidelines, under 'Reporting Guidelines'. Please confirm you have submitted this list.	Not Applicable	

Data Availability

Data availability	Information included in the manuscript?	In which section is the information available? (Reagents and Tools Table, Materials and Methods, Figures, Data Availability Section)
Have **primary datasets** been deposited according to the journal's guidelines (see 'Data Deposition' section) and the respective accession numbers provided in the Data Availability Section?	Yes	Data Availability Section
Were **human clinical and genomic datasets** deposited in a public access-controlled repository in accordance to ethical obligations to the patients and to the applicable consent agreement?	Not Applicable	
Are **computational models** that are central and integral to a study available without restrictions in a machine-readable form? Were the relevant accession numbers or links provided?	Not Applicable	
If publicly available data were reused, provide the respective **data citations in the reference list**.	Yes	Results/ Reference List
